# Simultaneous Installation of 1,6-Stereocenters by Asymmetric Allylation of Resorcinol Boronates: Application to the Synthesis of Merocyclophane A

**DOI:** 10.1021/jacs.6c14045

**Published:** 2026-10-22

**Authors:** Ze-Shu Wang, Joshua D. Mitchell, Jasper L. Tyler, Laura J. McCormick McPherson, Simon J. Coles, Varinder K. Aggarwal

**Affiliations:** † School of Chemistry, University of Bristol, Cantock’s Close, Bristol BS8 1TS, U.K.; ‡ UK National Crystallography Service, Department of Chemistry, 7423 University of Southampton, Highfield Campus, Southampton SO17 1BJ, U.K.

## Abstract

Installing multiple noncontiguous stereocenters in a single catalytic event offers a powerful platform for constructing complex three-dimensional molecular architectures, yet asymmetric methodologies capable of selectively forging remote 1,*n*-stereochemical relationships remain limited. Here we disclose a strategy for the simultaneous installation of 1,6-stereocenters via *para*-selective activation of chiral resorcinol boronates with π-allyl iridium complexes. Upon oxidation-induced rearomatization, the resulting dearomatized intermediates furnish the allylated products in an overall transformation that exhibits excellent regio- and stereoselectivity. Crucially, independent variation of the ligand and boronic ester configuration provides predictable access to all four stereoisomers with no observable match–mismatch effects. The synthetic potential of the methodology is exemplified by a concise total synthesis of the cyanobacteria-derived [7.7]­paracyclophane merocyclophane A, in which all stereocenters are installed simultaneously on the resorcinol core in a single step.

## Graphical abstract

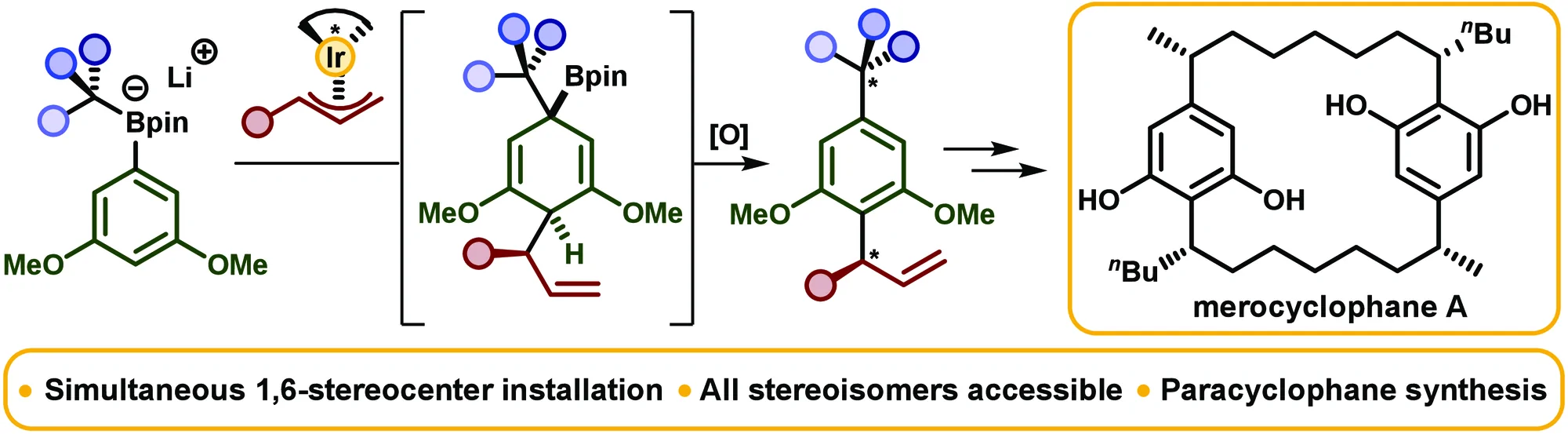

The simultaneous installation of multiple, noncontiguous chiral centers in a single transformation provides the potential to efficiently construct complex 3D molecular architectures. These strategies can also unlock new retrosynthetic disconnections that streamline the synthesis of structurally intricate bioactive compounds. However, as was highlighted in a recent review,^1^ developing methodologies for the catalytic asymmetric construction of 1,*n*-stereocenters (especially when *n* > 3) remains a considerable challenge.^2,3^

Nevertheless, the synthetic advantages offered by this approach have spurred chemists to devise creative solutions to facilitate the construction of distal chiral centers with high levels of stereoselectivity,^1,4–7^ for example through implementing highly tailored bifunctional catalysts (Figure 1a).^8–10^ However, control over asymmetric induction becomes increasingly difficult as the distance between stereogenic centers increases, owing to the greater level of flexibility and reduced energetic differentiation between competing transition states. An alternative solution is to employ two distinct catalysts that work synergistically to construct both stereogenic centers,^11–13^ either by simultaneously activating the coupling partners^14,15^ or by installing the stereocenters sequentially in a cascade process (Figure 1a).^16–20^ A noticeable advantage of this approach is that the judicious combination of the various catalyst enantiomers provides the potential to access different diastereoisomers of the targeted product.^14,15,21^ That being said, few examples of this strategy have been successfully demonstrated and are largely limited by the narrow window of multicatalyst compatibility and the existence of match–mismatch effects.^22–24^

**Figure 1:**
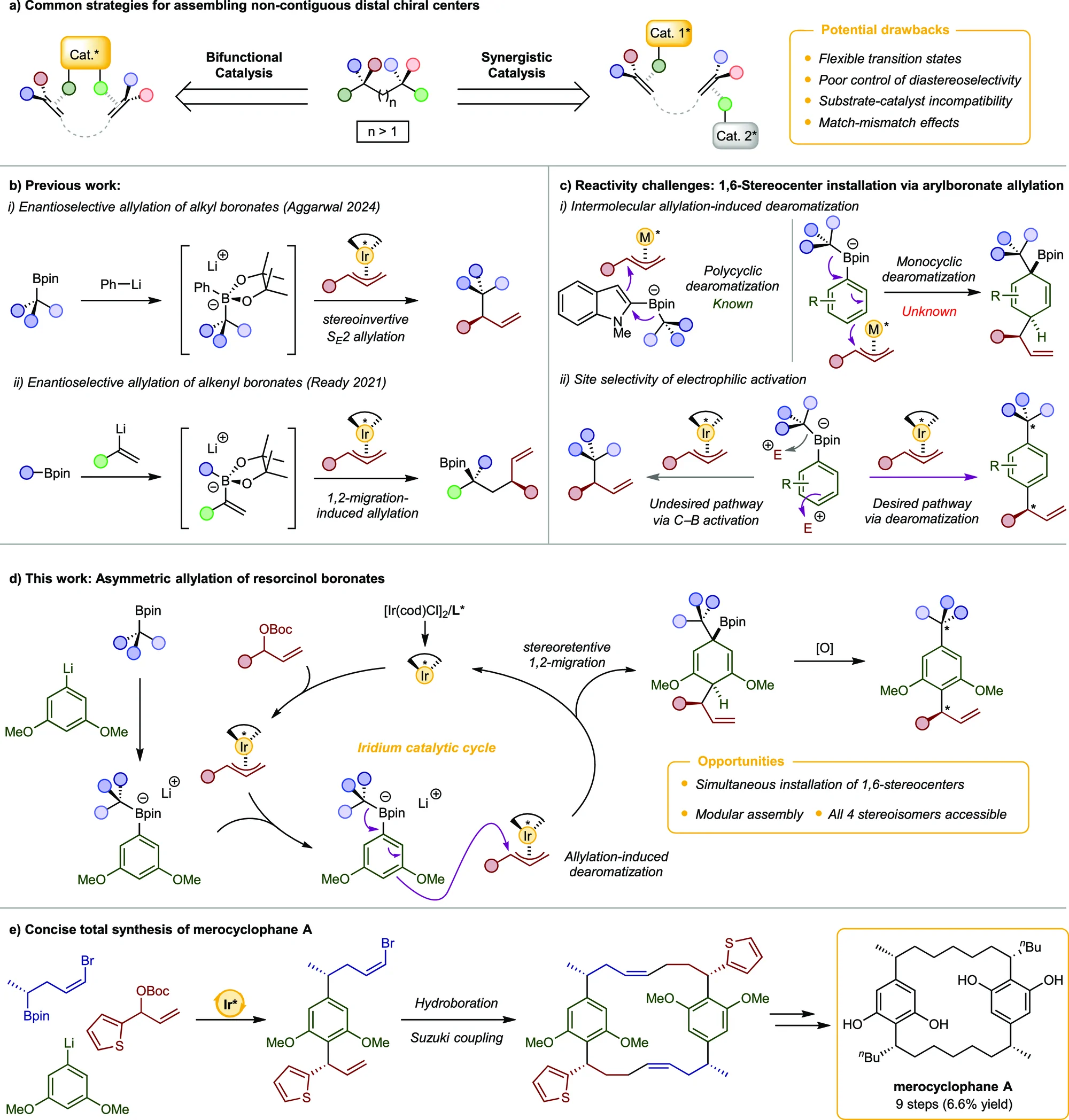
**Asymmetric allylation of resorcinol boronates for the simultaneous installation of 1,6-stereocenters.** (a) Common strategies for assembling noncontiguous stereocenters. (b) Previous work: Enantioselective allylation of alkyl/alkenylboronates. (c) Reactivity challenges of 1,6-stereocenter installation via arylboronate allylation. (d) This work: asymmetric allylation of resorcinol boronates for the simultaneous installation of 1,6-stereocenters. (e) Application to the total synthesis of merocyclophane A.

To circumvent these inherent challenges in the design of a new approach for the selective assembly of 1,*n*-stereocenters (*n* > 3), we considered strategies in which each stereocenter is independently controlled, as these approaches enhance stereochemical fidelity and broaden synthetic applicability. Inspiration came from our recent report on the simultaneous stereoinvertive and stereoselective (SimSS) C­(sp^3^)–C­(sp^3^) cross-coupling of chiral alkylboronates and π-allyl iridium complexes (Figure 1b­(i)).^25,26^ Here, the configuration around each stereocenter could be independently controlled by varying either the enantiomer of the boronic ester or the catalyst, providing access to all four possible stereoisomers of the product. In addition, Ready and Gao have demonstrated that the asymmetric allylation of alkenylboronate complexes can promote 1,2-metalate rearrangements to set 1,3-stereochemical relationships (Figure 1b­(ii)).^27–30^

Motivated by the potential of these reactivity modes, and informed by our group’s studies on enantiospecific C­(sp^2^)–C­(sp^3^) coupling of boronic esters,^31,32^ we conceived a system capable of delivering 1,6-stereocontrol via *para*-selective activation of an arylboronate with a chiral π-allyl iridium complex, thereby triggering a distal, stereoretentive 1,2-boronate migration. However, achieving such a transformation was expected to be highly challenging since it requires the dearomatization of a monocyclic aromatic hydrocarbon (Figure 1c­(i)). While polycyclic aromatic and heteroaromatic systems have been reported to react with π-allyl metal complexes leading to partially dearomatized intermediates,^33^ intermolecular allylation reactions of monocyclic aromatics have not, presumably due to the high energetic penalty that would result from full loss of aromaticity.^34,35^ Furthermore, to achieve the desired reactivity, electrophilic activation must occur regioselectively at the *para* position of the aromatic ring over the C–B bond (Figure 1c­(ii)).^25^

Herein, we report the regio- and stereocontrolled simultaneous installation of 1,6-chiral centers via the activation of monocyclic resorcinol boronates, derived from the corresponding aryllithium and chiral boronic ester, with π-allyl iridium complexes (Figure 1d). We propose that the reaction proceeds through electrophile-induced dearomatization of the resorcinol boronate accompanied by 1,2-boronate migration to generate a cyclohexadiene intermediate. This transformation may occur through a concerted dearomatization/1,2-migration pathway, consistent with mechanistic studies of related boronate rearrangements,^32^ or alternatively through a stepwise process via an oxonium intermediate. Upon oxidation of the resulting cyclohexadiene, rearomatization delivers the desired products which can be accessed as all four possible stereoisomers through variation of the ligand and boronic ester configuration. The unique potential of this reactivity is highlighted by its application to the concise total synthesis of the cyanobacteria-derived [7.7]­paracyclophane merocyclophane A,^36^ where, unlike in previous syntheses, all stereocenters are installed simultaneously in a single step (Figure 1e).

We began our studies by investigating the reaction of cyclohexyl boronic ester-derived arylboronate complexes with allyl carbonate **3**, in the presence of [Ir­(COD)­Cl]_2_ and (*S*)-**L**,^37^ followed by oxidation (Table 1). Interestingly, only resorcinol derivative **2a** displayed the desired reactivity, whereas less electron-rich aryllithium species were unable to deliver more than trace amounts of product **5** (Table 1, entries 1 and 2), highlighting that electron-rich aromatics are required to overcome the penalty of complete dearomatization. Pleasingly, however, exclusive *para* selectivity was observed and the resulting allylic stereocenter was formed in high enantiomeric excess (ee). Consistent with numerous reports of related asymmetric allylation reactions,^25–28,38^ we hypothesize that enantioselectivity arises from a kinetic resolution of the racemic allyl carbonate upon oxidative addition with the chiral catalyst.^38–40^ Notably, dearomatized intermediate **4** could also be isolated prior to oxidation in 55% yield,^41^ supporting our mechanistic proposal for the reaction.

**Table 1:**
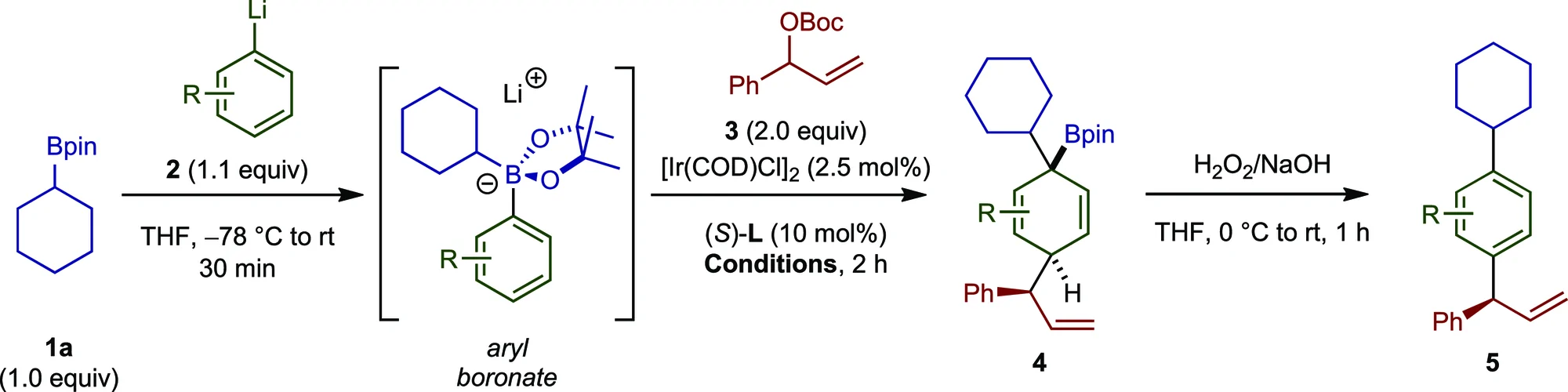

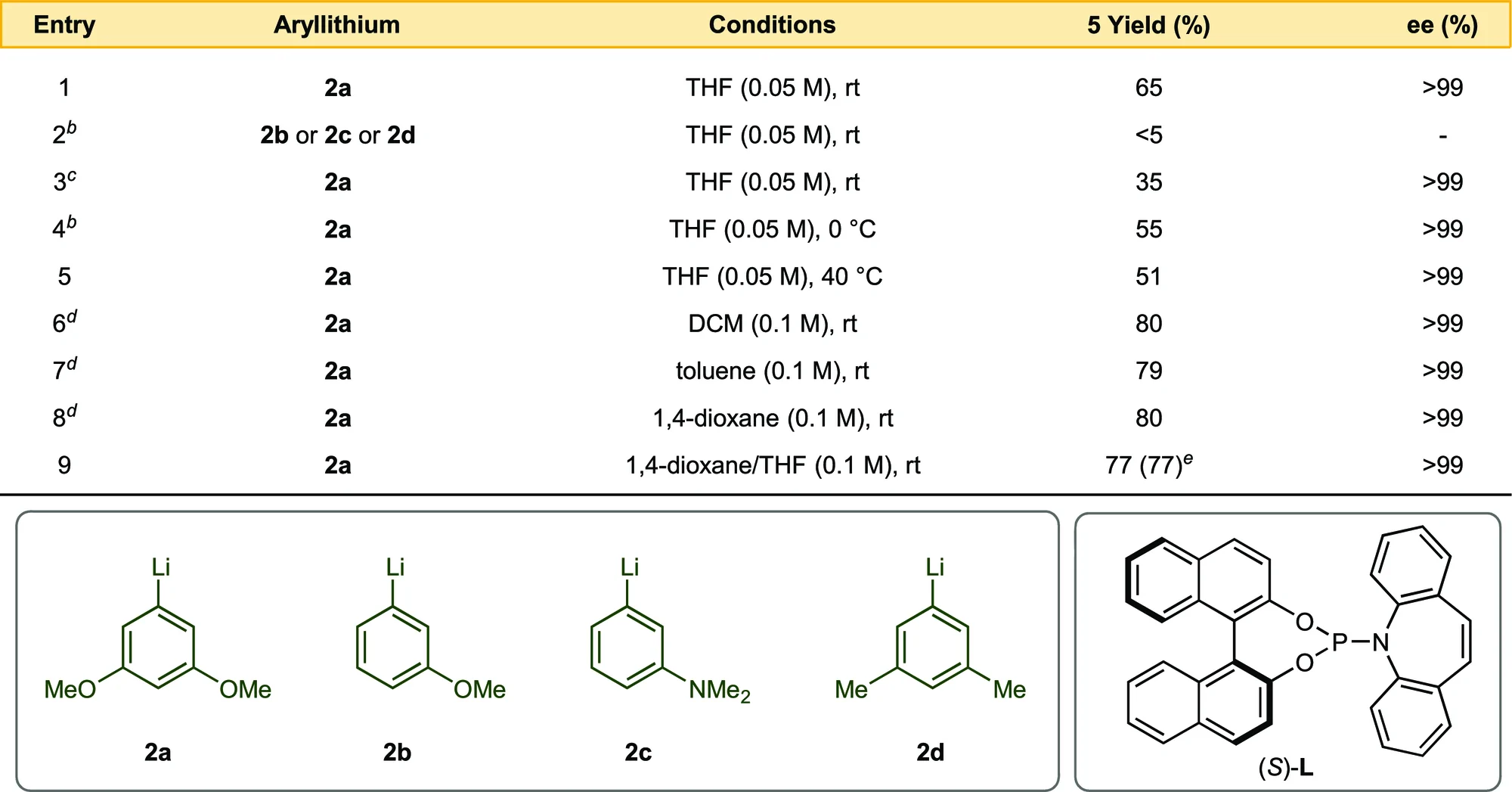
Reaction Optimization^b^

b The allylation step was run for 16 h.

c Using 1.0 equiv of **3**.

d A solvent switch was performed after boronate complex formation.

e Isolated yield.

a Reactions were conducted on a 0.2 mmol scale. Yields were determined by ^1^H NMR spectroscopy using 1,3,5-trimethoxybenzene as an internal standard. Product ee values were determined by HPLC analysis after hydroboration/oxidation.

During optimization, we found that an excess of allyl carbonate **3** and performing the reaction at room temperature was optimum (entries 3–5). Implementing a solvent switch following boronate complex formation was demonstrated to enhance reaction efficiency with a range of solvents (entries 6–8), albeit at the expense of practical convenience. However, comparable product yields could be achieved upon employing a 1,4-dioxane/THF solvent mixture, enabling access to **5** in 77% isolated yield and >99% ee (entry 9).

Having identified optimal conditions, the substrate scope of the reaction was evaluated (Table 2). To simplify our analysis of the stereoselectivity of the asymmetric allylation, we employed achiral 2-cyclohexyl-4,4,5,5-tetramethyl-1,3,2-dioxaborolane (**1a**) as the model substrate for exploring the allylic electrophile scope. A wide range of (hetero)­aryl-substituted allyl carbonates (**3b**–**3k**) bearing both electron-donating and electron-withdrawing groups at the *para*, *meta*, or *ortho* position were all successfully employed, delivering **5b**–**5k** in good yield and excellent enantioselectivity. In agreement with prior reports on related systems,^26,27,29^ alkyl-substituted allylic carbonates were unsuccessful in promoting the desired asymmetric allylation (**5l**), likely due to the lower reactivity of the resulting π-allyl iridium complex. Notably, the corresponding 3-pyridyl-substituted allyl carbonate was unsuccessful under the standard conditions (see the Supporting Information for unsuccessful substrates). Indeed, there are very few examples of Lewis basic heterocycles being used in iridium-catalyzed asymmetric allylic substitution reactions.^42^

**Table 2:**
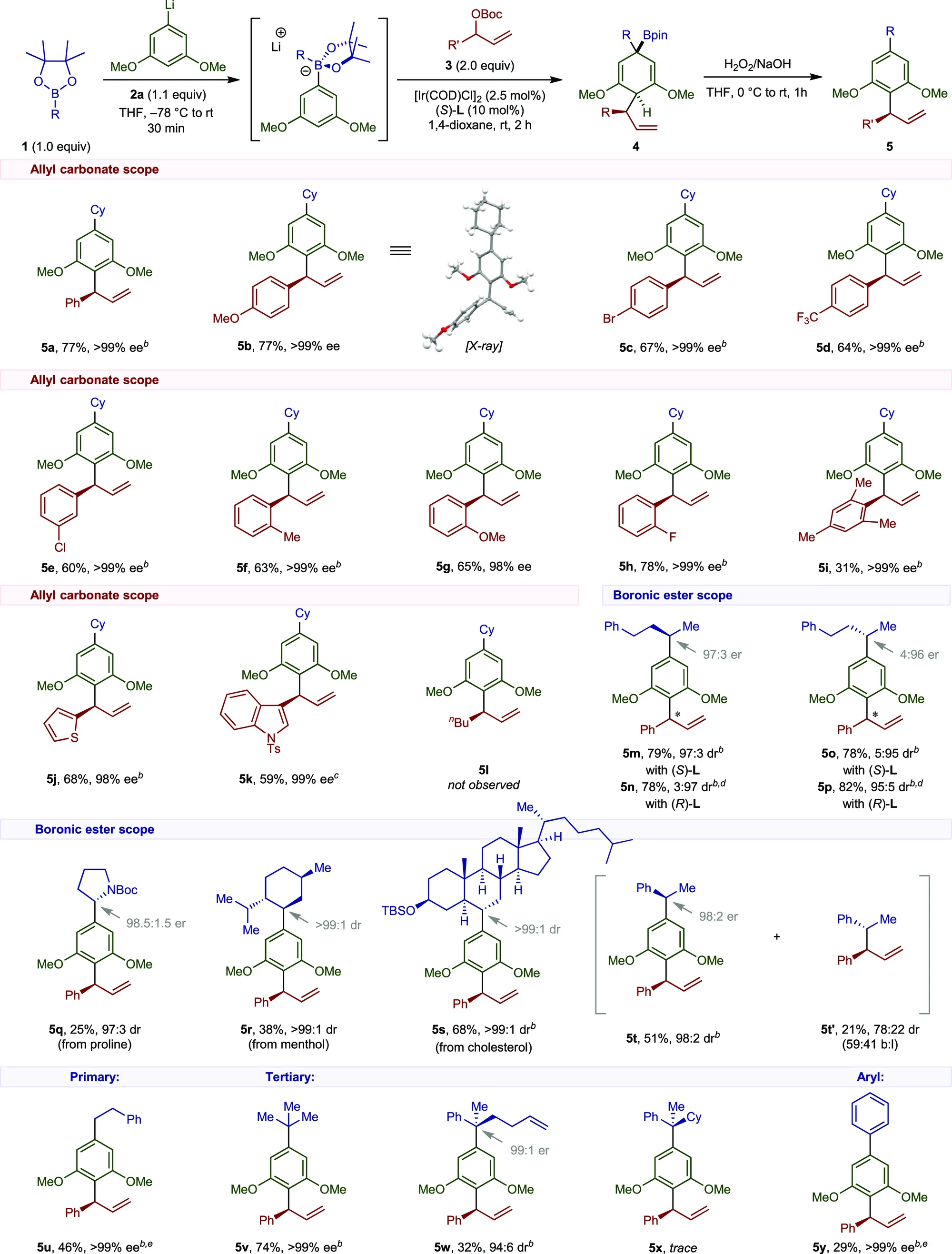
Substrate Scope for the Asymmetric Allylation of Resorcinol Boronates^*a*^

b The ee value was determined after hydroboration/oxidation.

c A solvent switch to 1,4-dioxane was performed.

d (*R*)-**L** was used instead of (*S*)-**L**.

e A solvent switch to DCM was performed.

a Reactions were conducted on a 0.2 mmol scale. Isolated yields are given. Product ee values were determined by HPLC analysis; er/dr values for stereocenters labeled with a gray arrow indicate the stereoisomeric ratios for the corresponding starting boronic esters.

Given that this study was conceived as a strategy to simultaneously and selectively install 1,6-chiral centers, we then explored the employment of enantioenriched boronic esters. Indeed, we found that product **5m** could be obtained in high yield and with complete enantiospecificity (es). In addition, employing the *R* and *S* enantiomers of both the starting boronic ester and chiral ligand allowed access to all four possible product stereoisomers (**5m**–**5p**) with no observable match–mismatch effects, as expected for remote stereocenters. The exploitation of chiral pool molecules was subsequently achieved through the synthesis of resorcinol adducts derived from proline, menthol, and cholesterol (**5q**–**5s**). To probe the regioselectivity of this transformation, we employed enantioenriched benzylic boronates, species which are known to readily react with π-allyl iridium complexes through a stereoinvertive interaction with the C–B bond.^25^ Interestingly, although **5t′** was indeed detected in this reaction (21% yield; 59:41 branched to linear ratio), formation of the desired product represents the major pathway, with **5t** isolated in 51% yield (98:2 dr). Continued exploration of the boronic ester scope demonstrated that primary and tertiary groups also undergo 1,2-migration to provide **5u**–**5w** in moderate yield and excellent enantioselectivity, although employing highly congested tertiary boronates severely compromises reactivity (**5x**; see the Supporting Information for further details of unsuccessful reactions). Finally, phenyl 1,2-migration was achieved (**5y**), albeit in reduced yields due to the lower reactivity of the boronate complex, demonstrating that the electrophile preferentially reacts on the more electron-rich aryl ring.

We then turned our attention toward applying this methodology to the synthesis of merocyclophane A,^36^ a member of the [7.7]­paracyclophane family of natural products which exhibit a broad spectrum of biological activity,^43,44^ and feature fascinating biosyntheses.^45–47^ Whereas previous syntheses of related natural products require multiple steps to independently assemble the chiral side chains around an aromatic core,^48–54^ we postulated that the installation of the 1,6-benzylic stereocenters could be achieved in a single step using our strategy. Moreover, the stereodivergent nature of our approach provides the potential to access all four stereoisomers using a common reaction platform, enabling systematic investigations of stereochemistry–activity relationships in a manner not readily accessible through existing routes.

Retrosynthetic analysis of this molecule identified that the installation of the exocyclic ^*n*^Bu chains would necessitate the employment of an alkyl allylic carbonate. However, such reagents proved incompatible with our transformation, consistent with prior reports of related systems (see **5l**, Table 2).^26,27,29^ Therefore, we proposed to use thiophene as a masked alkyl chain that could be unveiled upon reduction (Figure 2). Being *C*_2_-symmetric, molecules of this substructure are generally assembled through the dimerization of a monomeric unit via a variety of C–C bond-forming methods.^48–54^ We were initially drawn to utilizing a cross metathesis/ring-closing metathesis (CM/RCM) dimerization strategy using monomeric intermediate **8** that was prepared in 50% yield (95:5 dr) using our asymmetric resorcinol allylation protocol. However, the subsequent CM/RCM dimerization was unsuccessful, returning the dimer of the less hindered alkene together with truncated isomers (see the Supporting Information). This represented a surprising outcome, especially as this strategy has been successfully employed on a closely related substrate.^48^ We therefore considered an alternative dimerization strategy in which the macrocycle (**10**) is assembled from halogenated fragment **11** via alkene hydroboration followed by a double *B*-alkyl Suzuki–Miyaura coupling. Although Suzuki–Miyaura reactions have been widely employed in the formation of macrocycles,^55^ to the best of our knowledge, double cross-coupling involving an intermolecular followed by an intramolecular Suzuki–Miyaura reaction, has yet to be reported.

**Figure 2:**
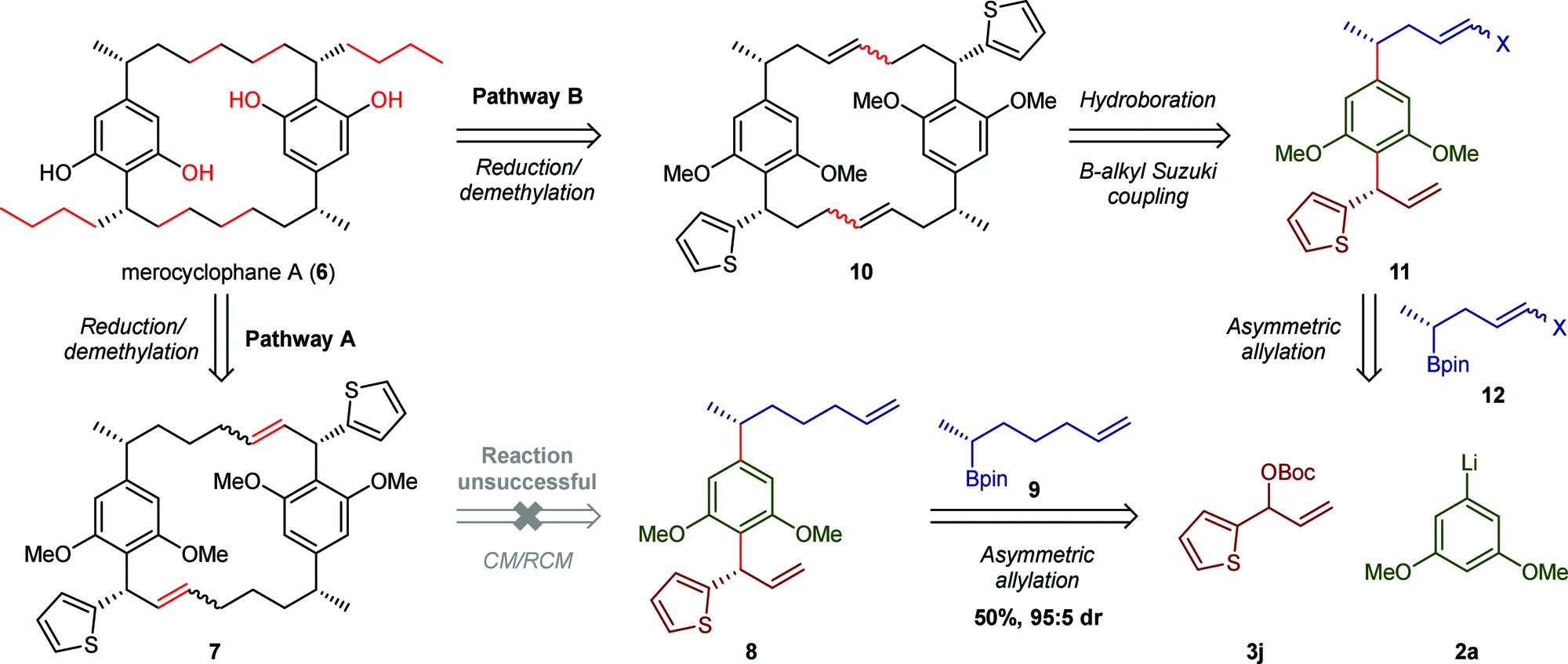
Retrosynthetic analysis of merocyclophane A.

Our synthesis began with the preparation of secondary boronic ester **12** (Figure 3a). Deprotonation of allyltrimethylsilane **13** followed by borylation gave boronic ester **14** ^56^ which was stereoselectively homologated to provide boronic ester **15** in 84% yield.^57^ Conversion to either (*E*)-vinyl bromide **12a** or iodide **12b** was achieved through the employment of the corresponding *N*-halosuccinimide.^58^ Pleasingly, both halides were tolerated in the asymmetric allylation reaction, allowing the simultaneous installation of the 1,6-benzylic stereocenters with high levels of selectivity (***E*-11a**/**b**). However, the desired hydroboration–Suzuki–Miyaura dimerization reaction delivered disappointingly low yields of product (Figure 3b, entries 1–4), potentially due to the formation of oligomers, even under high dilution conditions. We therefore considered utilizing the corresponding *Z* isomer as the change in reactive site geometry was expected to favor cyclization. Gratifyingly, (*Z*)-vinyl bromide **11c**, synthesized from ***Z*-12c** ^59–62^ in 60% yield (97.5:2.5 dr) on 1.4 mmol scale using our asymmetric allylation protocol, significantly enhanced dimerization efficiency (entries 5–6). After optimization, macrocycle **10** was obtained in 46% yield (see the Supporting Information), with X-ray crystallography confirming its structure and stereochemistry.^63^

**Figure 3:**
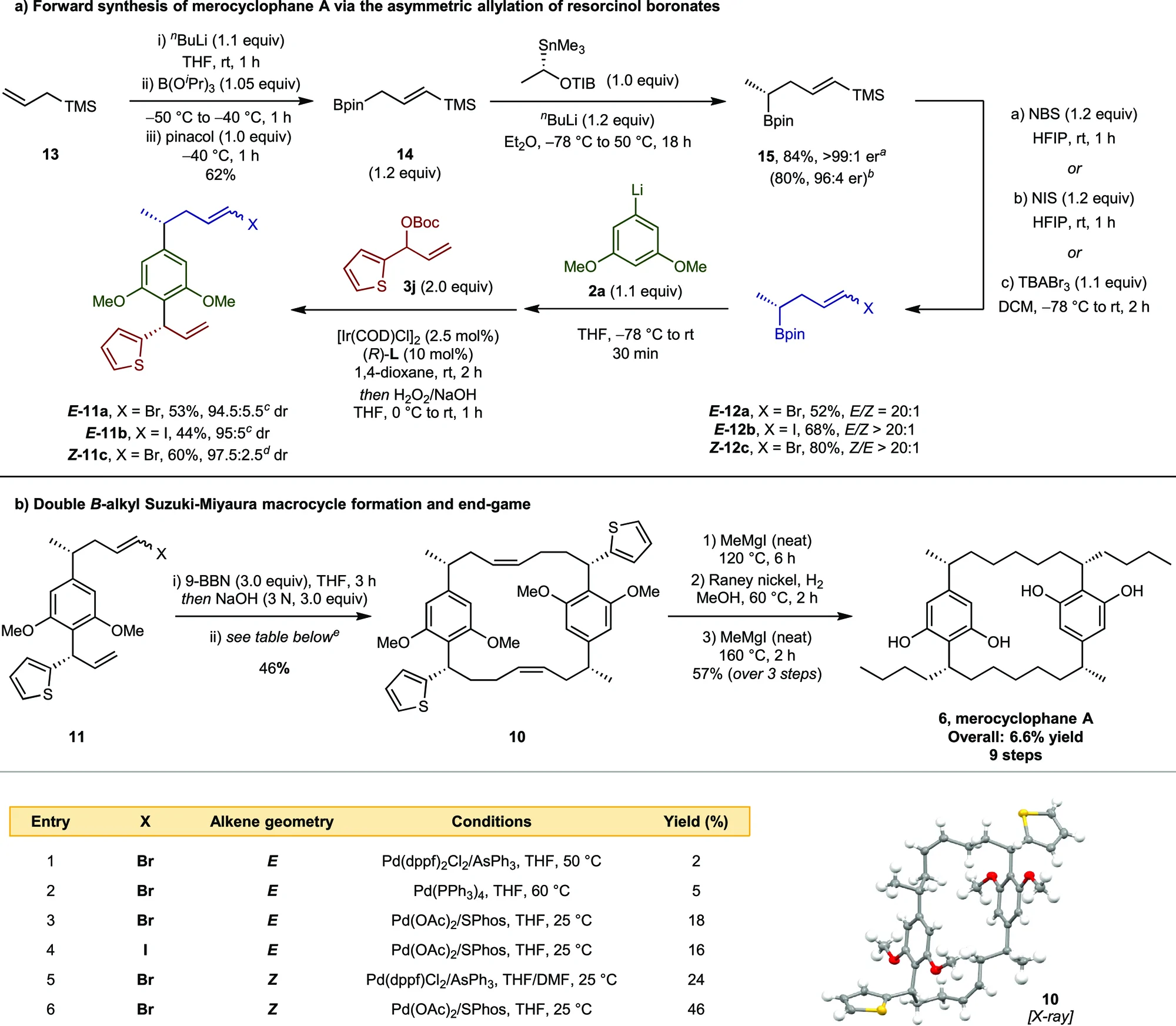
**Total synthesis of merocyclophane A.** (a) Forward synthesis of merocyclophane A via the asymmetric allylation of resorcinol boronates. (b) Macrocycle formation and endgame. ^*a*^Synthesis of **15** using the corresponding stannane (>99:1 er) as shown. ^*b*^Synthesis of **15** using sparteine-mediated deprotonation of EtOTIB (see the Supporting Information). ^*c*^The synthesis of ***E*-11a**/**b** was performed using the corresponding boronic esters with 96:4 er. ^*d*^The synthesis of **Z** **-** **11c** was performed using the corresponding boronic ester with >99:1 er. ^*e*^For reaction conditions, see the Supporting Information.

Focus then turned to demethylation and thiophene reduction, which initially proved challenging. Exhaustive demethylation using BBr_3_, thiophenol, or MeMgI^51^ led to substantial levels of decomposition, whereas Raney nickel reduction led to poor mass recovery of **10** ^64–66^ due to limited methanol solubility. However, partial demethylation with MeMgI yielded a methanol soluble mixture of mono-, di-, and tridemethylated products. This was subjected to Raney nickel reduction before treatment of the crude mixture with MeMgI, affording merocyclophane A (**6**) in 57% yield over three steps. Overall, this route represents a concise nine-step synthesis of merocyclophane A, where both stereogenic centers of the monomeric intermediate were simultaneously installed through our resorcinol boronate asymmetric allylation protocol.

In summary, we have developed an iridium-catalyzed regio- and stereocontrolled asymmetric allylation of resorcinol boronates. This transformation occurs via a 1,2-migration triggered through *para* activation of the arylboronate with a π-allyl iridium complex. Most notably, employing chiral boronic esters allows for the simultaneous installation of distal 1,6-stereocenters, with judicious choice of starting material and catalyst enantiomer providing access to all four possible stereoisomers of the product. The unique potential of this strategy was demonstrated through its application to the concise total synthesis of the [7.7]­paracyclophane merocyclophane A, where all stereocenters were installed simultaneously in a single step.

## Supplementary Material



## Notes

Experimental procedures and characterization data for new compounds (PDF)

## References

[1] Ji C.-L., Zou X.-Z., Gao D.-W. (2025). Catalytic Asymmetric Construction
of Nonadjacent Stereoelements. Angew. Chem.,
Int. Ed..

[2] Clayden J. (2009). Transmission
of Stereochemical Information over Nanometre Distances in Chemical
Reactions. Chem. Soc. Rev..

[3] Sailes H., Whiting A. (2000). The Control of Remote
Asymmetric Centres via Reduction
of Acyclic Carbonyl Functions. J. Chem. Soc.,
Perkin Trans. 1.

[4] Nicolaou K. C., Edmonds D. J., Bulger P. G. (2006). Cascade
Reactions in Total Synthesis. Angew. Chem.,
Int. Ed..

[5] Enders D., Grondal C., Hüttl M. R. M. (2007). Asymmetric Organocatalytic Domino
Reactions. Angew. Chem., Int. Ed..

[6] Moser D., Schmidt T. A., Sparr C. (2023). Diastereodivergent
Catalysis. JACS Au.

[7] Sun H., Ma Y., Xiao G., Kong D. (2024). Stereodivergent Dual Catalysis In
Organic Synthesis. Trends Chem..

[8] Paull D. H., Abraham C. J., Scerba M. T., Alden-Danforth E., Lectka T. (2008). Bifunctional Asymmetric Catalysis:
Cooperative Lewis
Acid/Base Systems. Acc. Chem. Res..

[9] Kong M., Tan Y., Zhao X., Qiao B., Tan C.-H., Cao S., Jiang Z. (2021). Catalytic
Reductive Cross Coupling and Enantioselective Protonation
of Olefins to Construct Remote Stereocenters for Azaarenes. J. Am. Chem. Soc..

[10] Deng Y., Shi X., Shi G., Lu X., Luo J., Deng L. (2022). One-Step Asymmetric
Construction of 1,4-Stereocenters via Tandem Mannich-Isomerization
Reactions Mediated by a Dual-Functional Betaine Catalyst. JACS Au.

[11] Huo X., Li G., Wang X., Zhang W. (2022). Bimetallic Catalysis in Stereodivergent
Synthesis. Angew. Chem., Int. Ed..

[12] Wu Y., Huo X., Zhang W. (2020). Synergistic
Pd/Cu Catalysis in Organic Synthesis. Chem.—Eur.
J..

[13] Kim U., Jung D. J., Jeon H. J., Rathwell K., Lee S. G. (2020). Synergistic
Dual Transition Metal Catalysis. Chem. Rev..

[14] Wang H., Zhang R., Zi W. (2024). Synergistic
Palladium/Copper-Catalyzed
1,4-Difunctionalization of 1,3-Dienes for Stereodivergent Construction
of 1,5-Nonadjacent Stereocenters. Angew. Chem.,
Int. Ed..

[15] Li P., Liu Z., Huo X., Zhang W. (2024). Stereodivergent Construction of 1,5/1,7-Nonadjacent
Tetrasubstituted Stereocenters Enabled by Pd/Cu-Cocatalyzed Asymmetric
Heck Cascade Reaction. Angew. Chem., Int. Ed..

[16] Masson-Makdissi J., Prieto L., Abel-Snape X., Lautens M. (2021). Enantio- and Diastereodivergent
Sequential Catalysis Featuring Two Transition-Metal-Catalyzed Asymmetric
Reactions. Angew. Chem., Int. Ed..

[17] Yang H.-R., Cheng X., Chang X., Wang Z.-F., Dong X.-Q., Wang C.-J. (2024). Copper/Ruthenium
Relay Catalysis Enables 1,6-Double
Chiral Inductions with Stereodivergence. Chem.
Sci..

[18] Tian K., Jin Z., Liu X.-L., He L., Liu H.-F., Yu P.-K., Chang X., Dong X.-Q., Wang C.-J. (2025). Stereodivergent
Assembly of δ-Valerolactones with an Azaarene-Containing Quaternary
Stereocenter Enabled by Cu/Ru Relay Catalysis. Chem. Sci..

[19] Fu C., He L., Chang X., Cheng X., Wang Z.-F., Zhang Z., Larionov V. A., Dong X.-Q., Wang C.-J. (2024). Copper/Ruthenium
Relay Catalysis for Stereodivergent Access to δ-Hydroxy α-Amino
Acids and Small Peptides. Angew. Chem., Int.
Ed..

[20] Chang X., Cheng X., Liu X.-T., Fu C., Wang W.-Y., Wang C.-J. (2022). Stereodivergent Construction of 1,4-Nonadjacent
Stereocenters
via Hydroalkylation of Racemic Allylic Alcohols Enabled by Copper/Ruthenium
Relay Catalysis. Angew. Chem., Int. Ed..

[21] Zhang J., Luo Y., Zheng E., Huo X., Ma S., Zhang W. (2024). Synergistic
Pd/Cu-Catalyzed 1,5-Double Chiral Inductions. J. Am. Chem. Soc..

[22] Martínez S., Veth L., Lainer B., Dydio P. (2021). Challenges and Opportunities
in Multicatalysis. ACS Catal..

[23] Allen A. E., MacMillan D. W. C. (2012). Synergistic
Catalysis: a Powerful Synthetic Strategy
for New Reaction Development. Chem. Sci..

[24] Du Z., Shao Z. (2013). Combining Transition
Metal Catalysis and Organocatalysis - an Update. Chem. Soc. Rev..

[25] Shen H.-C., Wang Z.-S., Noble A., Aggarwal V. K. (2024). Simultaneous Stereoinvertive
and Stereoselective C­(sp^3^)–C­(sp^3^) Cross-Coupling
of Boronic Esters and Allylic Carbonates. J.
Am. Chem. Soc..

[26] Shen H.-C., Aggarwal V. K. (2025). Iridium-Catalyzed Stereocontrolled C­(sp^3^)–C­(sp^3^) Cross-Coupling of Boronic Esters and Allylic
Carbonates Enabled by Boron-to-Zinc Transmetalation. J. Am. Chem. Soc..

[27] Davis C. R., Luvaga I. K., Ready J. M. (2021). Enantioselective Allylation of Alkenyl
Boronates Promotes a 1,2-Metalate Rearrangement with 1,3-Diastereocontrol. J. Am. Chem. Soc..

[28] Davis C. R., Fu Y., Liu P., Ready J. M. (2022). Mechanistic Basis for the Iridium-Catalyzed
Enantioselective Allylation of Alkenyl Boronates. J. Am. Chem. Soc..

[29] Tran M.-K., Ready J. M. (2024). Chemoselective and Stereoselective Allylation of Bis­(alkenyl)­boronates. Angew. Chem., Int. Ed..

[30] Jiang X.-M., Ji C.-L., Ge J.-F., Zhao J.-H., Zhu X.-Y., Gao D.-W. (2024). Asymmetric Synthesis of Chiral 1,2-Bis­(Boronic) Esters
Featuring Acyclic, Non-Adjacent 1,3-Stereocenters. Angew. Chem., Int. Ed..

[31] Bonet A., Odachowski M., Leonori D., Essafi S., Aggarwal V. K. (2014). Enantiospecific
sp^2^-sp^3^ Coupling of Secondary and Tertiary Boronic
Esters. Nat. Chem..

[32] Odachowski M., Bonet A., Essafi S., Conti-Ramsden P., Harvey J. N., Leonori D., Aggarwal V. K. (2016). Development
of Enantiospecific
Coupling of Secondary and Tertiary Boronic Esters with Aromatic Compounds. J. Am. Chem. Soc..

[33] Panda S., Ready J. M. (2017). Palladium Catalyzed Asymmetric Three-Component Coupling
of Boronic Esters, Indoles, and Allylic Acetates. J. Am. Chem. Soc..

[34] Wu Q.-F., Liu W.-B., Zhuo C.-X., Rong Z.-Q., Ye K.-Y., You S.-L. (2011). Iridium-Catalyzed Intramolecular Asymmetric Allylic
Dearomatization of Phenols. Angew. Chem., Int.
Ed..

[35] Xu Q.-L., Dai L.-X., You S.-L. (2012). Enantioselective Synthesis of Tetrahydroisoquinolines
via Iridium-Catalyzed Intramolecular Friedel–Crafts-Type Allylic
Alkylation of Phenols. Org. Lett..

[36] Kang H.-S., Santarsiero B. D., Kim H. J., Krunic A., Shen Q., Swanson S. M., Chai H.-B., Kinghorn A. D., Orjala J. (2012). Merocyclophanes
A and B, Antiproliferative Cyclophanes from the Cultured Terrestrial *Cyanobacterium nostoc* sp. Phytochemistry.

[37] Hoffman T. J., Carreira E. M. (2011). Catalytic Asymmetric
Intramolecular Hydroacylation
with Rhodium/Phosphoramidite-Alkene Ligand Complexes. Angew. Chem., Int. Ed..

[38] Tu H.-F., Yang P., Lin Z.-H., Zheng C., You S.-L. (2020). Time-Dependent
Enantiodivergent Synthesis via Sequential Kinetic Resolution. Nat. Chem..

[39] Rössler S. L., Petrone D. A., Carreira E. M. (2019). Iridium-Catalyzed
Asymmetric Synthesis
of Functionally Rich Molecules Enabled by (Phosphoramidite, Olefin)
Ligands. Acc. Chem. Res..

[40] Cheng Q., Tu H.-F., Zheng C., Qu J.-P., Helmchen G., You S.-L. (2019). Iridium-Catalyzed
Asymmetric Allylic Substitution Reactions. Chem.
Rev..

[41] Compound **4** was isolated as a 1.2:1 mixture of 1,4 *trans:cis* diastereoisomers. See the Supporting Information.

[42] Chen D., Zhang M., Zhang D., Zhang Z., Shao X., Xu X., Li Z., Yang W.-L. (2024). Iridium/Acid
Dual-Catalyzed Enantioselective
Aza-ene-type Allylic Alkylation of Nitro Ketene Aminals with Racemic
Allylic Alcohols. Org. Lett..

[43] Moore B. S., Chen J. L., Patterson G. M., Moore R. E., Brinen L. S., Kato Y., Clardy J. (1990). [7.7]­Paracyclophanes
from Blue-Green
Algae. J. Am. Chem. Soc..

[44] Martins T. P., Rouger C., Glasser N. R., Freitas S., de Fraissinette N. B., Balskus E. P., Tasdemir D., Leão P. N. (2019). Chemistry,
Bioactivity and Biosynthesis of Cyanobacterial Alkylresorcinols. Nat. Prod. Rep..

[45] Nakamura H., Schultz E. E., Balskus E. P. (2017). A New Strategy for Aromatic Ring
Alkylation in Cylindrocyclophane Biosynthesis. Nat. Chem. Biol..

[46] Nakamura H., Hamer H. A., Sirasani G., Balskus E. P. (2012). Cylindrocyclophane
Biosynthesis Involves Functionalization of an Unactivated Carbon Center. J. Am. Chem. Soc..

[47] Braffman N. R., Ruskoski T. B., Davis K. M., Glasser N. R., Johnson C., Okafor C. D., Boal A. K., Balskus E. P. (2022). Structural Basis
for an Unprecedented Enzymatic Alkylation in Cylindrocyclophane Biosynthesis. eLife.

[48] Berthold D., Breit B. (2018). Total Synthesis of
(−)-Cylindrocyclophane F: A Yardstick for
Probing New Catalytic C–C Bond-Forming Methodologies. Chem.—Eur. J..

[49] Yamakoshi H., Ikarashi F., Minami M., Shibuya M., Sugahara T., Kanoh N., Ohori H., Shibata H., Iwabuchi Y. (2009). Syntheses
of Naturally Occurring Cytotoxic [7.7]­Paracyclophanes, (−)-Cylindrocyclophane
A and Its Enantiomer, and Implications for Biological Activity. Org. Biomol. Chem..

[50] Smith A. B., Kozmin S. A., Adams C. M., Paone D. V. (2000). Assembly of (−)-Cylindrocyclophanes
A and F via Remarkable Olefin Metathesis Dimerizations. J. Am. Chem. Soc..

[51] Hoye T. R., Humpal P. E., Moon B. (2000). Total Synthesis of (−)-Cylindrocyclophane
A via a Double Horner–Emmons Macrocyclic Dimerization Event. J. Am. Chem. Soc..

[52] Nicolaou K. C., Sun Y.-P., Korman H., Sarlah D. (2010). Asymmetric Total Synthesis
of Cylindrocyclophanes A and F through Cyclodimerization and a Ramberg-Bäcklund
Reaction. Angew. Chem., Int. Ed..

[53] Chen K.-Y., Wang H.-Q., Yuan Y., Mou S.-B., Xiang Z. (2023). Chemoenzymatic
Synthesis of Cylindrocyclophanes A and F and Merocyclophanes A and
D. Angew. Chem., Int. Ed..

[54] Bosse A. T., Hunt L. R., Suarez C. A., Casselman T. D., Goldstein E. L., Wright A. C., Park H., Virgil S. C., Yu J.-Q., Stoltz B. M., Davies H. M. L. (2024). Total Synthesis
of (−)-Cylindrocyclophane A Facilitated by C-H Functionalization. Science.

[55] Chemler S. R., Trauner D., Danishefsky S. J. (2001). The B-Alkyl Suzuki–Miyaura
Cross-Coupling Reaction: Development, Mechanistic Study, and Applications
in Natural Product Synthesis. Angew. Chem.,
Int. Ed..

[56] Adam, J.-M. ; Abel, M. ; Jayaraman, S. Process for Preparation of Cyclosporin A Analogs Having a Terminal Diene Group. WO 2004089960 A2, 2004.

[57] Aiken S. G., Bateman J. M., Liao H.-H., Fawcett A., Bootwicha T., Vincetti P., Myers E. L., Noble A., Aggarwal V. K. (2023). Iterative
Synthesis of 1,3-Polyboronic Esters with High Stereocontrol and Application
to the Synthesis of Bahamaolide A. Nat. Chem..

[58] Ilardi E. A., Stivala C. E., Zakarian A. (2008). Hexafluoroisopropanol
as a Unique
Solvent for Stereoselective Iododesilylation of Vinylsilanes. Org. Lett..

[59] Ogawa N., Katagiri K., Haimoto Y., Kobayashi Y. (2022). Total Synthesis
of Resolvin D3. Org. Biomol. Chem..

[60] Li L., Ye X., Wu Y., Gao L., Song Z., Yin Z., Xu J. (2013). Sakurai Reaction of
3,3-Bis­(silyl) Silyl Enol Ethers with Acetals
Involving Selective Desilylation of the Geminal Bis­(silane). Concise
Synthesis of Nematocidal Oxylipid. Org. Lett..

[61] Kobayashi Y., Yoshida S., Asano M., Takeuchi A., Acharya H. P. (2007). Nickel-Catalyzed
Coupling Producing (2*Z*)-2,4-Alkadien-1-ols, Conversion
to (*E*)-3-Alkene-1,2,5-triol Derivatives, and Synthesis
of Decarestrictine D. J. Org. Chem..

[62] The bromination of (*E*)-vinyl silane using Br_2_ used in refs 58–60 gave 7.8:1 *Z*/*E*. When TBABr_3_ was used instead, the *Z*/*E* ratio was improved to >20:1.

[63] Coles S. J., Allan D. R., Beavers C. M., Teat S. J., Holgate S. J. W., Tovee C. A. (2020). Leading Edge Chemical Crystallography Service Provision
and Its Impact on Crystallographic Data Science in the Twenty-First
Century. Struct. Bonding (Berlin).

[64] Högberg H. E., Hedenström E., Fägerhag J., Servi S. (1992). Bakers’ Yeast
Reduction of Thiophenepropaenals. Enantioselective Synthesis of (S)-2-Methyl-1-alkanols
via Bakers’ Yeast Mediated Reduction of 2-Methyl-3-(2-thiophene)­propenals. J. Org. Chem..

[65] Radha
Krishna P., Lavanya B., Ilangovan A., Sharma G. V. M. (2000). Stereoselective Synthesis of *C*-Alkyl
and Functionalized *C*-Alkyl Glycosides Using ‘Thiophene’
as a Masked C-4 Synthon. Tetrahedron: Asymmetry.

[66] Hoye T. R., Ye Z., Yao L. J., North J. T. (1996). Synthesis of the *C*
_2_-Symmetric,
Macrocyclic Alkaloid, (+)-Xestospongin A
and Its C(9)-Epimer, (−)-Xestospongin C: Impact of Substrate
Rigidity and Reaction Conditions on the Efficiency of the Macrocyclic
Dimerization Reaction. J. Am. Chem. Soc..

