# Supplementary material for: Simultaneous Installation of 1,6-Stereocenters by Asymmetric Allylation of Resorcinol Boronates: Application to the Synthesis of Merocyclophane A
Source: J Am Chem Soc. 2026 Oct 22;148(43):46485–93. doi: 10.1021/jacs.6c14045 (PMC13741844; doi:10.1021/jacs.6c14045)
Supplement: Supplementary file 1 [file ja6c14045_si_001.pdf]

Supporting Information for

Simultaneous Installation of 1,6-Stereocentres by Asymmetric Allylation of Resorcinol Boronates: Application to the Synthesis of Merocyclophane A

Ze-Shu Wang[a], Joshua D. Mitchell[a], Jasper L. Tyler[a], Laura J. McCormick McPherson[b], Simon J. Coles[b] and Varinder K. Aggarwal[a,*]

[a]School of Chemistry, University of Bristol, Cantock's Close, Bristol, BS8 1TS, U.K.

[b]School of Chemistry and Chemical Engineering, University of Southampton, Southampton, SO17 1BJ, U.K.

*Correspondence to: v.aggarwal@bristol.ac.uk

Contents

1. Materials and General Methods .... - 4 -

1.1. Glassware, Solvents and Reagents .... - 4 -

1.2. Chromatography and Instrumentation .... - 4 -

1.3. Naming of Compounds .... - 5 -

2. Experimental Data .... - 6 -

2.1. Reaction Optimization .... - 6 -

2.1.2. Reaction temperature screening .... - 6 -

2.1.3. Reaction stoichiometry screening .... - 7 -

2.1.4. Reaction solvent screening .... - 8 -

2.2. Synthesis of Starting Materials .... - 9 -

2.2.1. Synthesis of boronic esters .... - 9 -

2.2.2. Synthesis of allyl carbonates .... - 9 -

2.2.3 Synthesis of ligands .... - 10 -

2.3. General Procedures .... - 10 -

2.3.1. General procedure A: asymmetric allylation of resorcinol boronates .... - 10 -

2.3.2. General procedure B: hydroboration-oxidation to measure enantiomeric excess- 12 -

2.3.3. General procedure C: Suzuki coupling to measure enantiomeric ratio .... - 12 -

2.4. Unsuccessful Substrates .... - 13 -

2.5. Isolation of Cyclohexadiene Intermediate .... - 13 -

2.6. Characterization Data of Reaction Products .... - 15 -

3. The Total Synthesis of Merocyclophane A .... - 40 -

3.1. Forward Synthesis of Merocyclophane A .... - 40 -

3.2. Synthesis of *E*-Vinyl Halides (**11a**/**b**) .... - 49 -

3.3. Optimization of the Suzuki Cyclodimerization Reaction .... - 56 -

3.4. Optimization of the Demethylation Reaction .... - 57 -

3.5. Attempted Cross Metathesis/Ring-Closing Metathesis Cyclodimerization .... - 58 -

3.5.1. Synthesis of compound **8** .... - 58 -

3.5.2. Cross metathesis/ring-closing metathesis cyclodimerization optimization ...... - 61 -

3.6. Comparison of Spectroscopic Data for Synthetic and Isolated Merocyclophane A .... - 62 -

3.6.1. ^{1}H NMR of merocyclophane A from isolation paper .... - 62 -

3.6.2. ^{1}H and ^{13}C NMR of previously synthesised merocyclophane A .... - 63 -

3.6.3. ^{1}H and ^{13}C NMR of merocyclophane A synthesised in this study .... - 64 -

4. X-ray Crystallography .... - 65 -

4.1. Crystallographic Data for Compound **5b** (CCDC 2539493) .... - 65 -

4.2. Crystallographic Data for Compound **10** (CCDC 2539492) .... - 65 -

5. NMR Spectra .... - 69 -

6. References .... - 108 -

1. Materials and General Methods

1.1. Glassware, Solvents and Reagents

All manipulations were performed with oven-dried (130 °C for a minimum of 12 h) or flame-dried glassware using standard Schlenk techniques under an atmosphere of nitrogen, unless otherwise stated.

All anhydrous solvents were commercially supplied or dried using an Anhydrous Engineering alumina column drying system (THF, toluene, Et_2O, MeCN, CH_2Cl_2). These solvents were transferred, stored, and used under a positive pressure of nitrogen in a Young's valve-sealed, flame-dried Strauss flask containing 3 Å molecular sieves, activated by microwave irradiation (150 °C, 10 × 30 s cycles). All reactions were carried out using dry solvents unless stated in the procedure.

Reagents were purchased from commercial sources and used as received. **Exceptions**: *N*-bromosuccinimide (NBS) was recrystallised from boiling MeCN. All organolithium reagents were titrated against *N*-benzylbenzamide.[1]

1.2. Chromatography and Instrumentation

Thin layer chromatography (TLC) was performed using Merck Kieselgel 60 F254 fluorescent treated silica and visualised under UV light, or by staining with either aqueous basic potassium permanganate or vanillin solution followed by heating.

Preparative thin-layer chromatography was performed using Merck preparative thin-layer chromatography plates (20 cm × 20 cm; 500 μm of layer thickness; 20–40 μm of particle size; 60 Å medium pore diameter).

Flash column chromatography was conducted using Sigma-Aldrich silica gel (60 Å, 230–400 mesh, 40–63 μm).

NMR spectra were recorded at various field strengths, as indicated, using Bruker 400 MHz, Varian VNMR 400 MHz, Varian VNMR 500 MHz, or Bruker Cryo 500 MHz for ^{1}H and ^{13}C. All NMR spectra were recorded at 25 °C unless otherwise stated. Chemical shifts (δ) are reported in parts per million (ppm) and referenced to $CDCl_3$ (^{1}H: 7.26 ppm; ^{13}C: 77.0 ppm) or CD_3OD (^{1}H: 3.31 ppm; ^{13}C: 49.0 ppm). Coupling constants (*J*) are given in Hertz (Hz) and refer to apparent multiplicities (s = singlet, d = doublet, t = triplet, q = quartet, quin = quintet, hex = hextet, hept = heptet, m = multiplet, br = broad signal, dd = doublet of doublets, etc.).

The ^{1}H NMR spectral data are reported as follows: chemical shift (multiplicity, coupling constants, number of protons). NMR yields were determined, unless otherwise stated, using 1,3,5-trimethoxybenzene as the internal standard.

Gas chromatography spectra (GC) were recorded on an Agilent 7890 GC equipped with a HP-5MS column (15 m × 0.25 mm, 0.25 μm film thickness) and a split/splitless injection port (split ratio: 35:1, flow rate: 42 mL/min).

High resolution mass spectra (HRMS) were recorded on a Bruker Daltonics MicrOTOF II by Electrospray Ionisation (ESI); a Thermo Scientific QExactive by Electron Ionisation (EI); or a Bruker UltrafleXtreme by Matrix-assisted Laser Desorption/Ionisation (MALDI).

Optical rotations ($[\alpha]_D^T$) were measured in CH_2Cl_2 (unless otherwise indicated) using a Bellingham & Stanley ADP 220 Polarimeter.

1.3. Naming of Compounds

Compound names are those generated by ChemDraw Professional 20.0 software (PerkinElmer), following the IUPAC nomenclature.

2. Experimental Data

2.1. Reaction Optimization

The procedures of reaction optimization were performed according to **General Procedure A** with modifications.

2.1.2. Reaction temperature screening

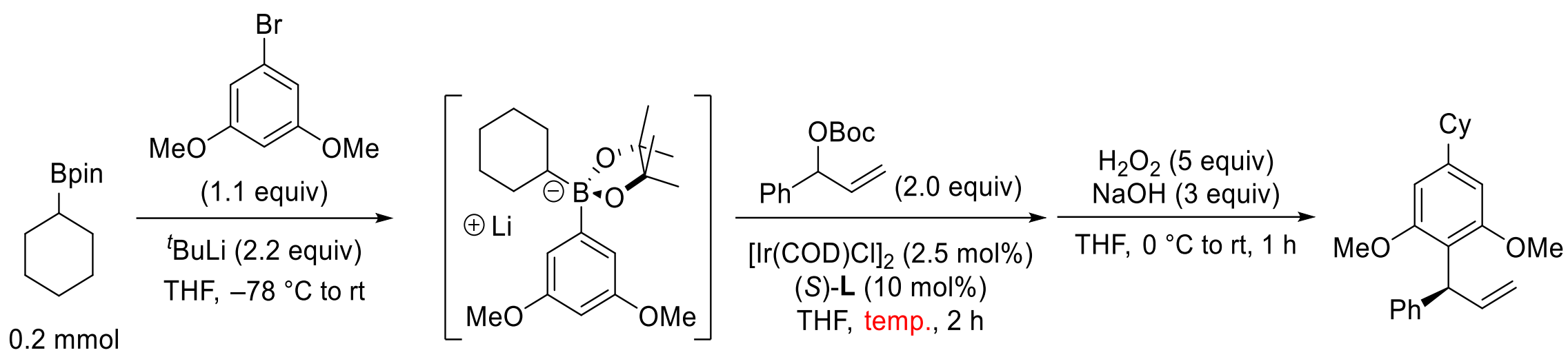

entry	temp.	yield (%)[a]	ee (%)[b]
1	rt	65	>99
2[c]	0 °C	55	>99
3	40 °C	51	>99
4	60 °C	37	99

Table S1: Reaction temperature screening

[a] ^{1}H NMR yields are reported; [b] enantiomeric excess was determined after hydroboration-oxidation; [c] allylation was run for 17 h.

2.1.3. Reaction stoichiometry screening

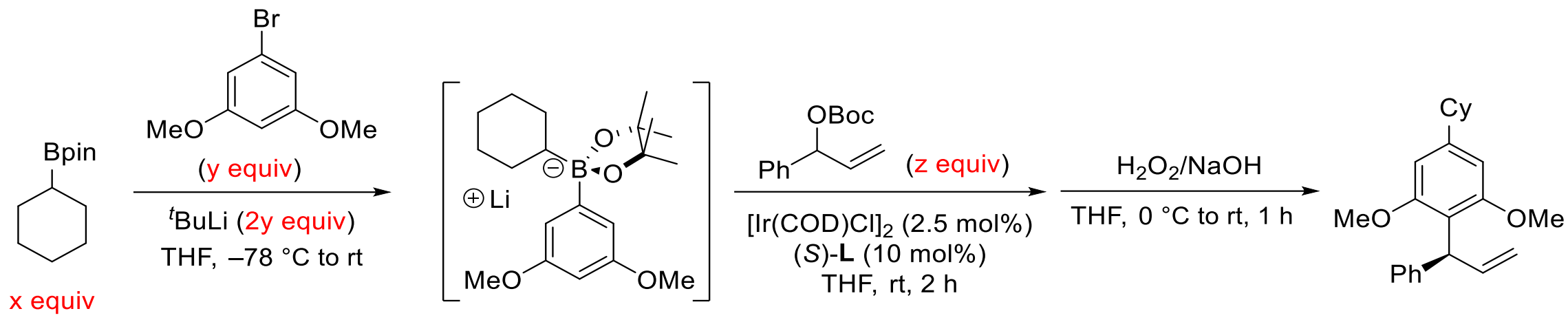

entry	x	y	z	yield (%) [a]	ee (%) [b]
1	1	1.1	2	65	>99
2	1	1.1	1	35	99
3	1	1.1	3	67	>99
4	1	1.3	2	40	99
5	1.1	1	2	59	99
6	1	1	2	62	99

Table S2: Reaction stoichiometry screening

[a] ^{1}H NMR yields are reported; [b] enantiomeric excess was determined after hydroboration-oxidation.

2.1.4. Reaction solvent screening

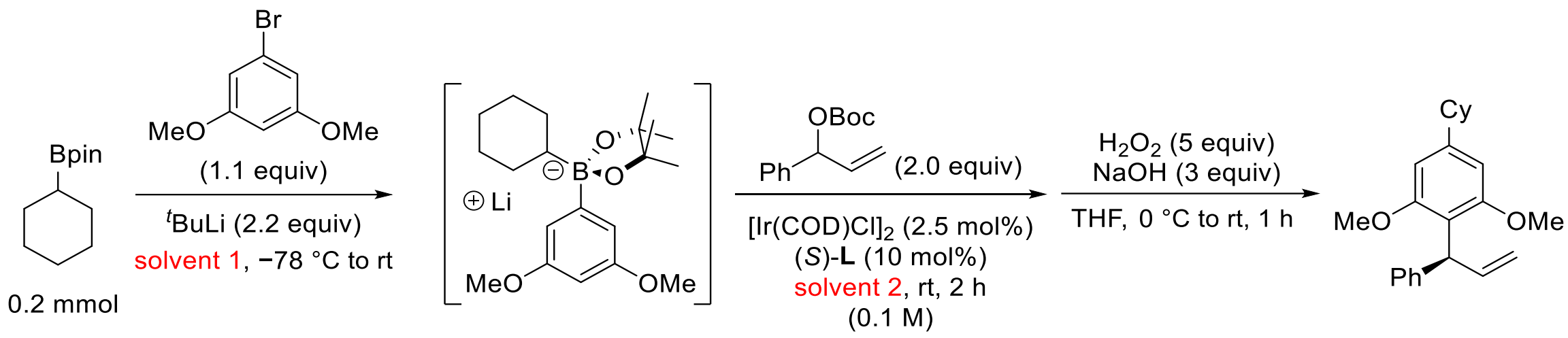

entry	solvent 1	solvent 2	yield (%) [a]	ee (%) [b]
1[d]	THF	THF	65	>99
2[d]	Et_2O	Et_2O	70	99
3[c,e]	Et_2O	DCM	64	>99
4[c,e]	Et_2O	toluene	57	>99
5[c,e]	Et_2O	1,4-dioxane	76	98
6[c,e]	Et_2O	EtOAc	57	>99
7[c,e]	Et_2O	Et_2O	75	99
8[c,e]	THF	THF	70	99
9[c,e]	THF	DCM	80	99
10[c,e]	THF	toluene	80	99
11[c,e]	THF	1,4-dioxane	80	99
12[c,d]	THF	1,4-dioxane	74	96
12[d]	THF	1,4-dioxane	71	>99
13[e]	THF	1,4-dioxane	77 (77)[f]	>99

Table S3: Reaction solvent screening

[a] ^{1}H NMR yields are reported; [b] enantiomeric excess was determined after hydroboration-oxidation; [c] solvent switch was performed; [d] 0.05 M; [e] 0.1 M; [f] isolated yield.

2.2. Synthesis of Starting Materials

2.2.1. Synthesis of boronic esters

Boronic esters used in this article were previously synthesized according to the corresponding literature reports.[2]

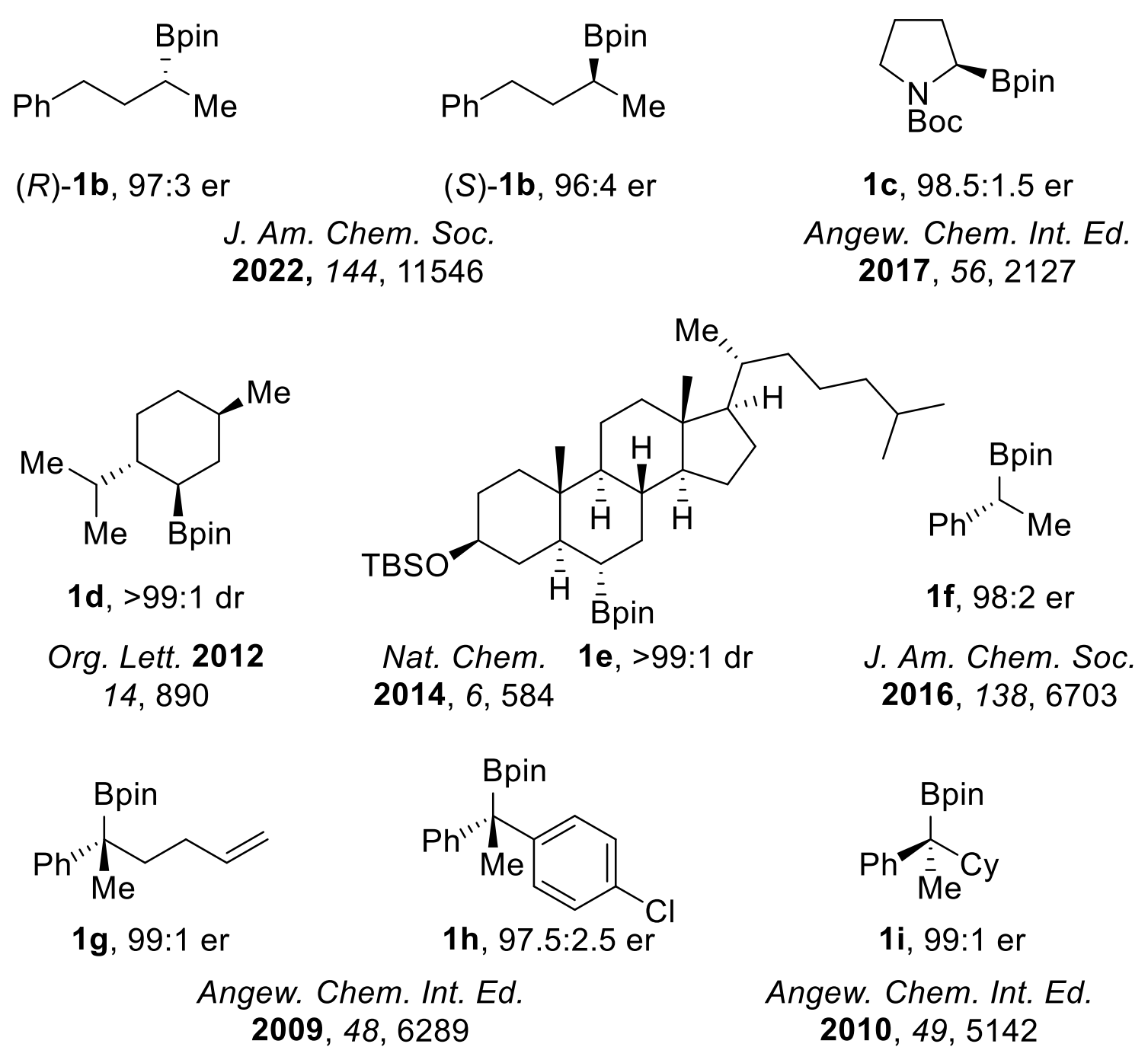

2.2.2. Synthesis of allyl carbonates

Allyl carbonates used in this article were synthesized according to previous reports.[3]

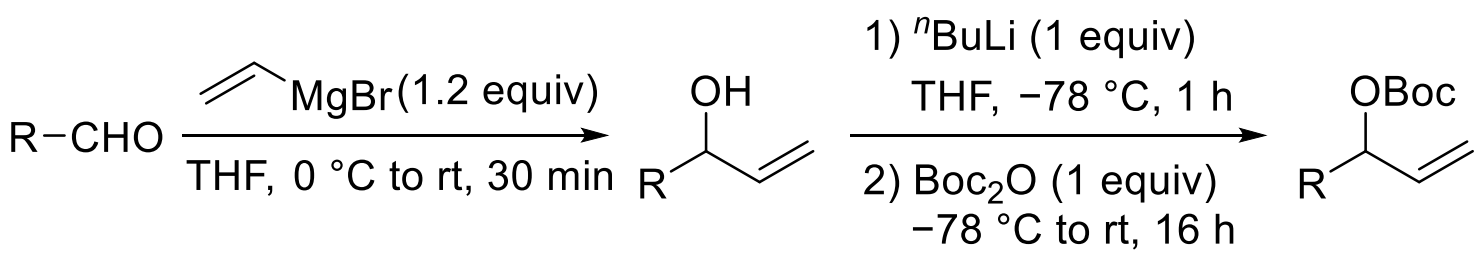

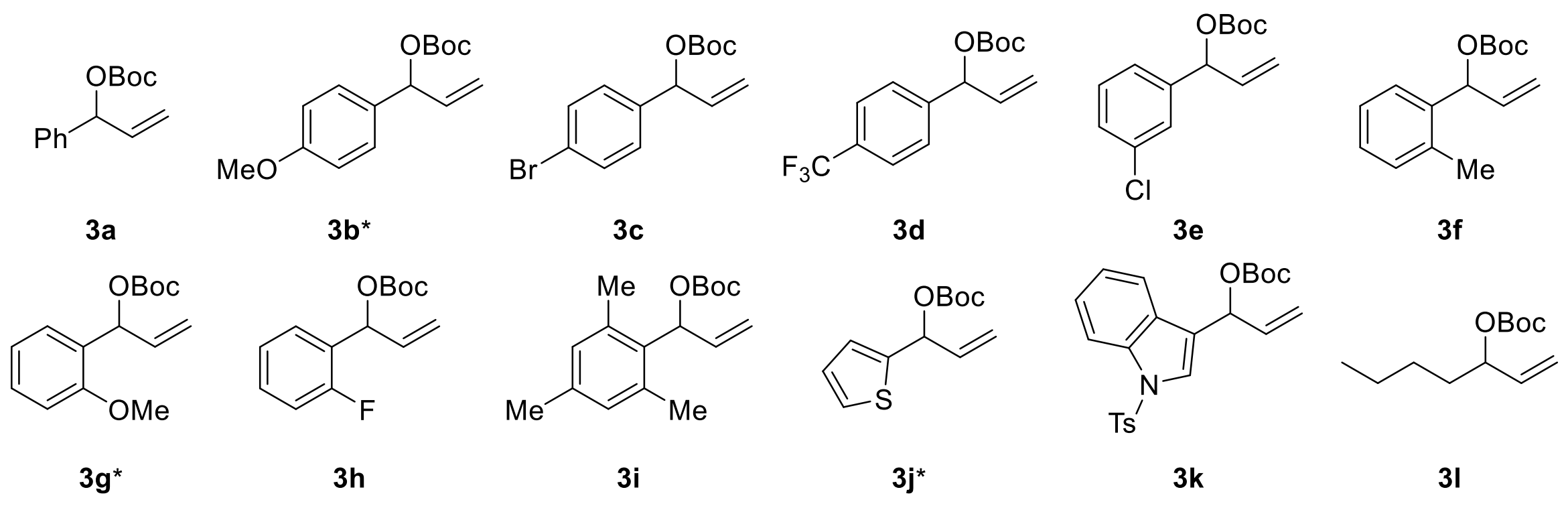

*3 V% of Et_3N in hexane was used as the eluent for silica gel column chromatography.

2.2.3 Synthesis of ligands

(*S*)-**L** is commercially available from Merck and Fluorochem. (*R*)-**L** was synthesized according to previous reports[3] and (*rac*)-**L'** was synthesized according to previous reports.[4]

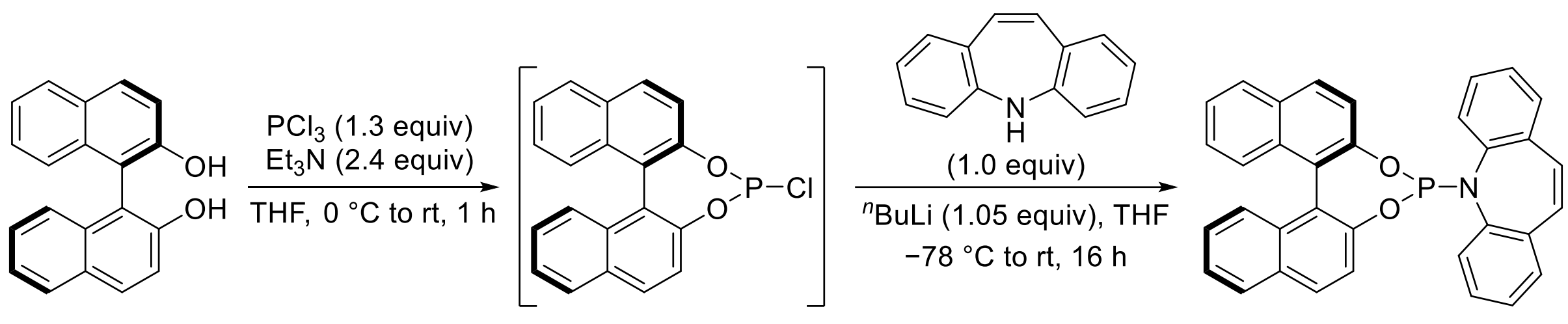

(*R*)-**L**

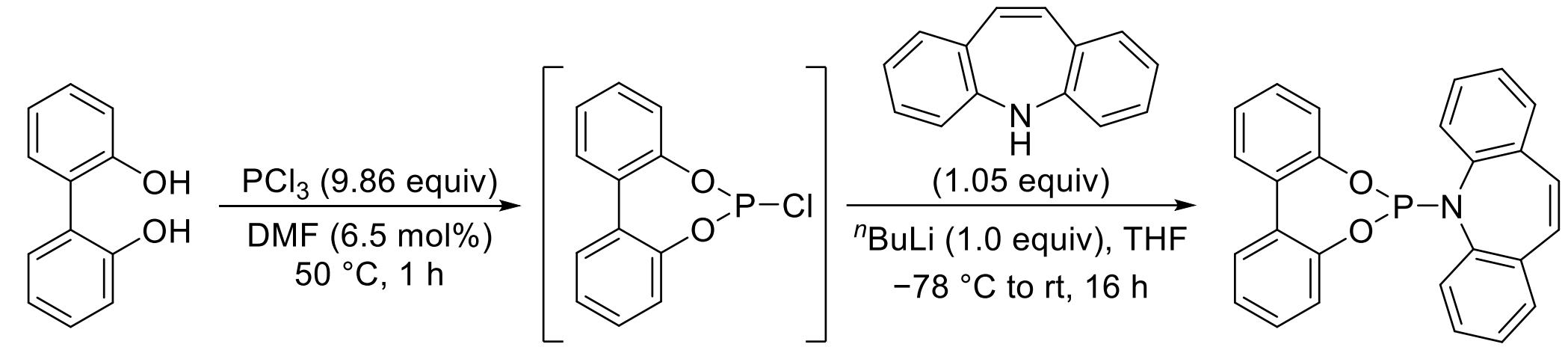

(*rac*)-**L'**

2.3. General Procedures

2.3.1. General procedure A: asymmetric allylation of resorcinol boronates

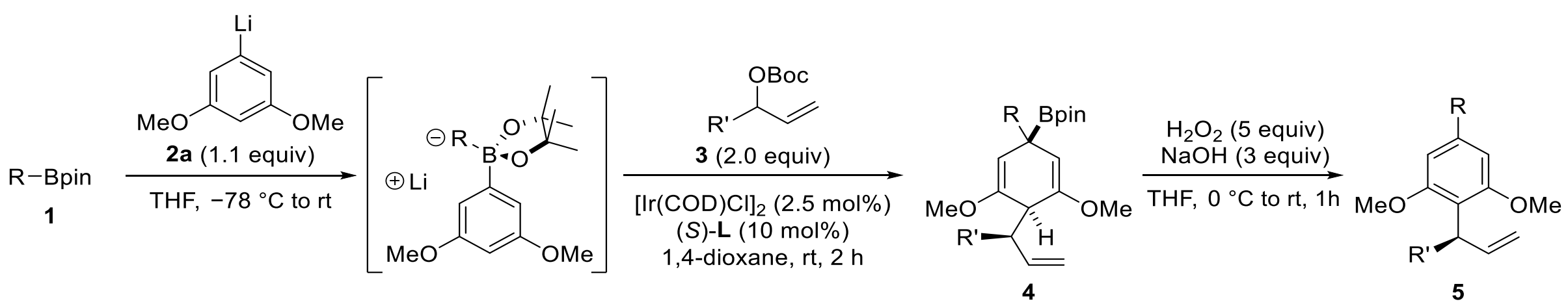

Step 1 boronate complex generation: A flame-dried 20 mL Schlenk tube equipped with a magnetic stir bar was cooled down to room temperature under vacuum, then back-filled with N_2 3 times. 1-Bromo-3,5-dimethoxybenzene (47.8 mg, 0.220 mmol, 1.10 equiv) was added under N_2 and the tube was back-filled with N_2 one more time. Anhydrous THF (0.5 mL) was added to the tube under N_2. The solution was cooled to −78 °C (dry ice/acetone) and ^{t}BuLi (0.26 mL, 0.44 mmol, 2.2 equiv, 1.67 M in hexane) was added dropwise (Note 1). The mixture was allowed to stir for 1 hour at −78 °C. Meanwhile, to an oven-dried 7 mL vial equipped with a magnetic stir bar was added boronic ester (0.20 mmol, 1.0 equiv) and anhydrous THF (0.3 mL). The vial was sealed with a septum and removed from the glovebox. The boronic ester solution was then added to the aryl lithium solution at −78 °C and the vessel was rinsed with additional THF (0.2 mL). The mixture was allowed to stir for ~5 min at −78 °C and then

warmed to room temperature and further stirred for 30 min to form the boronate complex (Note 2). **Step 2 asymmetric allylation:** To an oven-dried 7 mL vial equipped with a magnetic stir bar in a glovebox, was added $[Ir(cod)Cl]_2$ (3.4 mg, 5.0 μmol, 2.5 mol%) and (*S*)-**L** or (*R*)-**L** (10.1 mg, 20.0 μmol, 10.0 mol%). The vial was sealed with a septum and removed from the glovebox, then anhydrous 1,4-dioxane (0.5 mL) was added under N_2. The mixture was stirred for 30 min at room temperature before allyl *tert*-butyl carbonate (0.40 mmol, 2.0 equiv) was added and stirred for ~1 min to form the π-allyl iridium complex (Note 3). The π-allyl iridium complex solution was then added to the above boronate complex at room temperature. The π-allyl iridium complex solution vial was rinsed with additional anhydrous 1,4-dioxane (0.5 mL). Finally, the mixture was stirred for 2 h at room temperature. The crude reaction mixture was filtered through a pad of silica gel and the filtrate was concentrated under reduced pressure. **Step 3 oxidation:** The crude material was dissolved in THF (3 mL) and then cooled to 0 °C. Next, 3 N aqueous NaOH (0.2 mL, 0.6 mmol, 3.0 equiv) and 30% aqueous H_2O_2 (0.1 mL, 1 mmol, 5 equiv) were added sequentially. The reaction was allowed to warm to room temperature and stirred for 1 h before being quenched with aqueous $Na_2S_2O_3$ (~0.5 mL) at 0 °C. The organic layer was separated, and the aqueous phase was extracted with Et_2O (5 mL) three times. The combined organic layers were dried over $MgSO_4$, filtered, and concentrated under reduced pressure. The crude mixture was purified by flash column chromatography with hexane/toluene to give the corresponding product (Note 4).

Note 1: ^{t}BuLi (1.7 M solution in pentane) was regularly titrated with *N*-benzylbenzamide before use.

Note 2: In some cases, a solvent-switch was employed following formation of the boronate complex. THF was removed under high vacuum, after which the solution of the π-allyl iridium complex in 1,4-dioxane or DCM was added to the boronate complex.

Note 3: The π-allyl iridium complex solution should be prepared during boronate complex formation.

Note 4: Generally, it's easier to isolate desired product from side products (1,3-dimethoxybenzene and remaining allyl carbonate) using hexane/toluene as the eluent. Product formation could be monitored by TLC analysis, using vanillin stain.

2.3.2. General procedure B: hydroboration-oxidation to measure enantiomeric excess

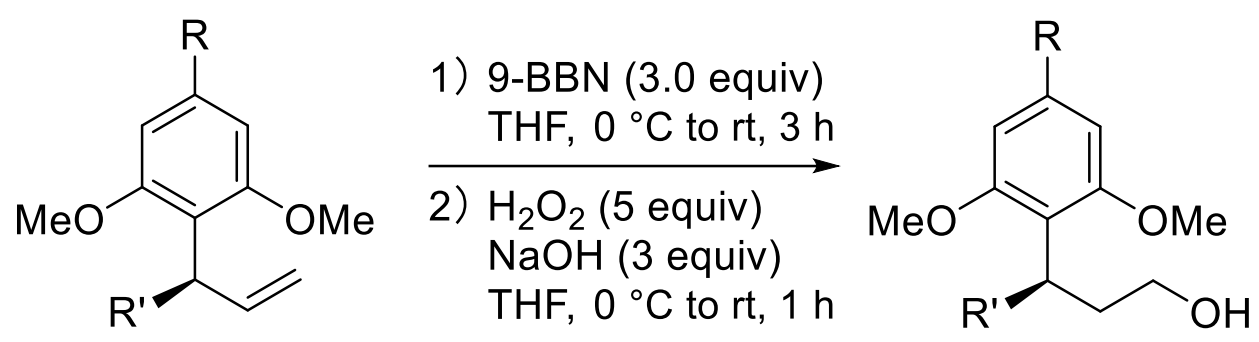

For products which could not be analysed directly by chiral HPLC, hydroboration-oxidation was performed. 9-BBN (0.15 mmol, 3.0 equiv, 0.5 M in THF) was added to a solution of the alkene (0.05 mmol, 1 equiv) in THF (0.5 mL, 0.1 M) at 0 °C. The mixture was stirred at room temperature for 3 h. The reaction was cooled to 0 °C, then 3 N aqueous NaOH (0.15 mL, 0.45 mmol, 9.0 equiv) and 30% aqueous H_2O_2 (0.075 mL, 0.75 mmol, 15 equiv) were added. The reaction was allowed to warm to room temperature and stirred for 1 h before being quenched with aqueous $Na_2S_2O_3$ (~0.5 mL) at 0 °C. The organic layer was separated, and the aqueous phase was extracted with Et_2O (2 mL) three times. The combined organic layers were dried over $MgSO_4$, filtered, and concentrated under reduced pressure. The crude mixture was purified by flash column chromatography or preparative TLC with hexane/EtOAc as the eluent to give the corresponding product.

2.3.3. General procedure C: Suzuki coupling to measure enantiomeric ratio

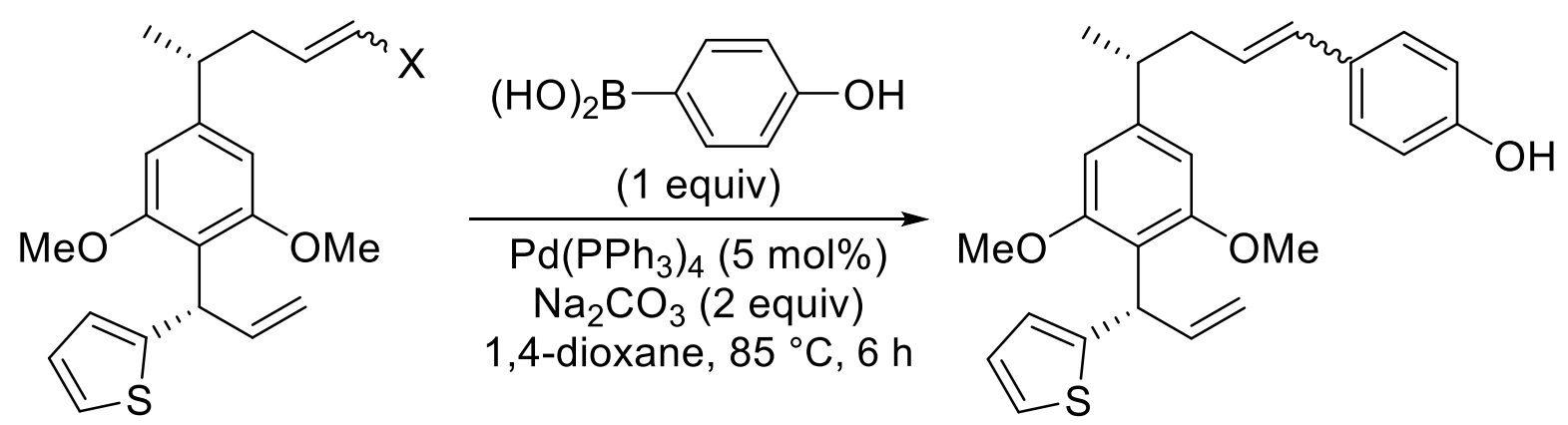

For products **11a-c** which could not be analysed directly by chiral HPLC, Suzuki coupling with (4-hydroxyphenyl)boronic acid was performed. To a 7 mL vial equipped with a magnetic stir bar in a glove box was added vinyl halide (0.05 mmol, 1 equiv), (4-hydroxyphenyl)boronic acid (6.9 mg, 0.050 mmol, 1.0 equiv), and $Pd(PPh_3)_4$ (2.9 mg, 2.5 µmol, 5.0 mol%). 1,4-Dioxane (1 mL) was then added followed by solution of 1 M aq. Na_2CO_3 (0.1 mL, 0.1 mmol, 2 equiv). The vial was sealed and removed from the glovebox. The reaction was stirred at 85 °C for 6 h. The crude reaction mixture was cooled down to room temperature and then filtered through a pad of Na_2SO_4 and silica gel. The filtrate was concentrated under reduced pressure. The crude mixture was purified by preparative TLC with hexane/EtOAc as the eluent to give the corresponding product (Note).

Note: The product is unstable, so quick measurement of the ee is essential.

2.4. Unsuccessful Substrates

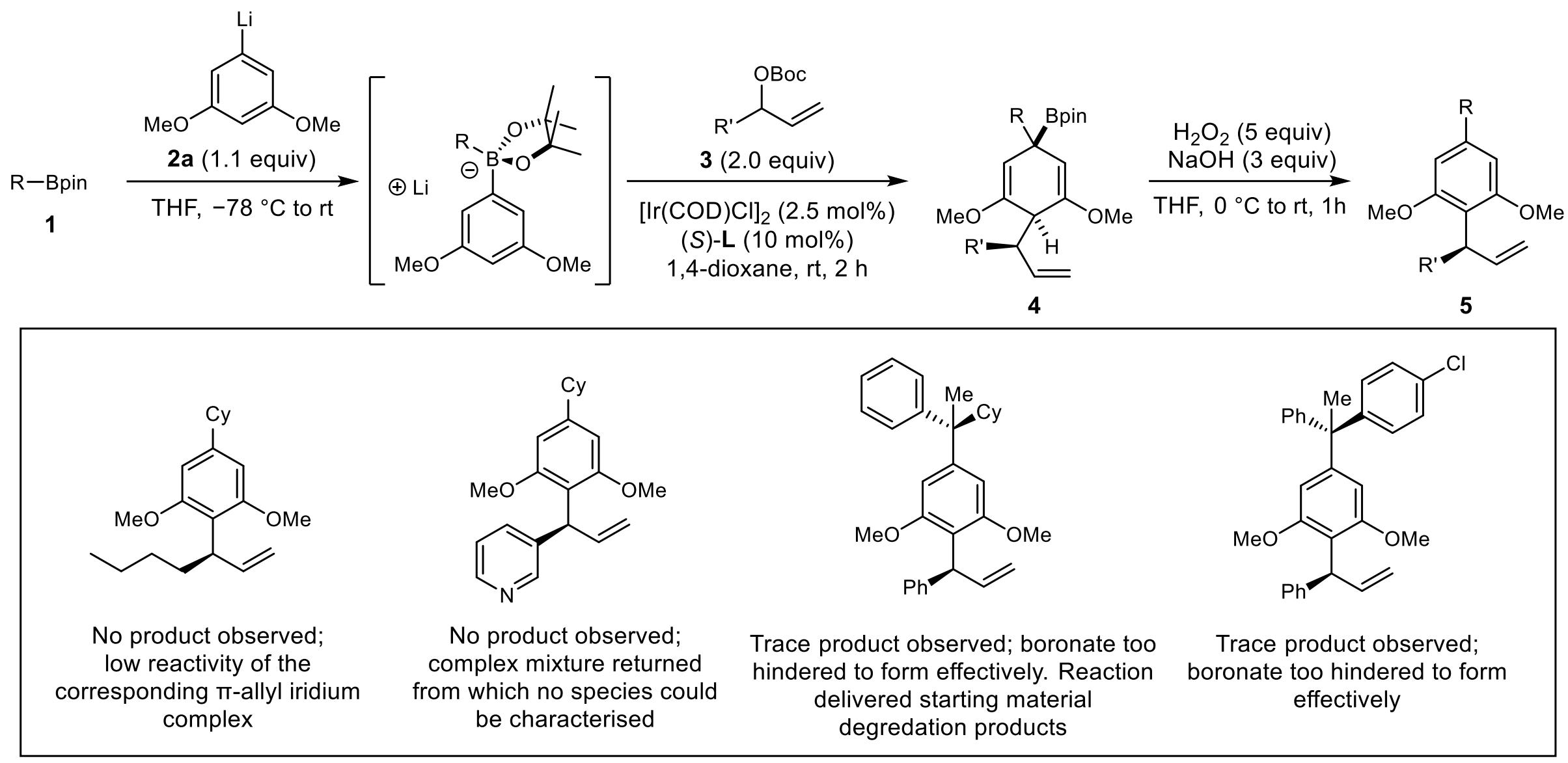

2.5. Isolation of Cyclohexadiene Intermediate

2-((1*S*,4*R*)-3,5-dimethoxy-4-((*R*)-1-phenylallyl)-[1,1'-bi(cyclohexane)]-2,5-dien-1-yl)-4,4,5,5-tetramethyl-1,3,2-dioxaborolane (4a-minor)

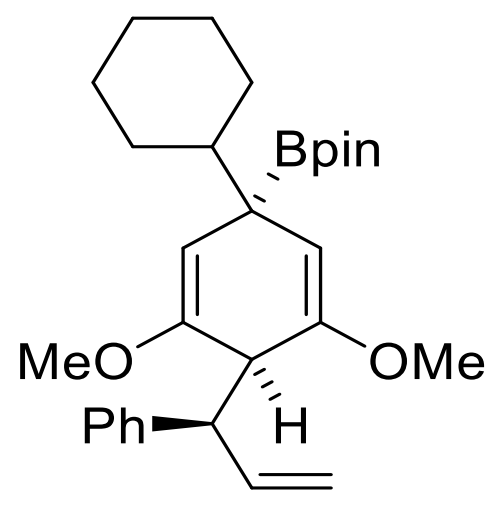

4a-minor

Prepared following **General Procedure A** using boronic ester **1a** (42.0 mg, 0.200 mmol, 1.00 equiv) and carbonate **3a** (93.7 mg, 0.400 mmol, 2.00 equiv) without oxidation. The crude mixture was purified by flash column chromatography (hexane/toluene = 2:1 to 1:1) gave the title compound (23.1 mg, 25%) as a colourless oil.

R*f* = 0.16 (2:1 hexane/toluene, UV, $KMnO_4$)

^{1}H NMR (400 MHz, $CDCl_3$) δ 7.24 – 7.16 (m, 4H), 7.14 – 7.05 (m, 1H), 6.29 (dt, *J* = 18.0, 9.2 Hz, 1H), 5.00 – 4.97 (m, 1H), 4.97 – 4.92 (m, 1H), 4.56 (s, 1H), 4.52 (s, 1H), 3.87 (dd, *J* = 9.2, 4.0 Hz, 1H), 3.45 (d, *J* = 3.2 Hz, 6H), 3.22 (d, *J* = 4.0 Hz, 1H), 1.73 – 1.68 (m, 1H), 1.68 – 1.65

(m, 1H), 1.66 – 1.51 (m, 3H), 1.52 – 1.42 (m, 1H), 1.28 – 1.12 (m, 14H), 1.11 – 0.92 (m, 3H) ppm. **^{13}C NMR** (126 MHz, $CDCl_3$) δ 152.9, 152.4, 144.0, 140.5, 128.5, 127.7, 125.6, 114.8, 98.5, 98.3, 83.1, 54.3, 54.1, 52.3, 48.5, 45.8, 29.0, 28.9, 27.1, 27.1, 27.0, 24.9, 24.9 ppm.

Specific rotation $[\alpha]_D^{24}$ = +16 (c = 2.0, CH_2Cl_2).

HRMS (ESI) m/z calculated for $C_{29}H_{42}BO_4$ $[M+H]^+$, 465.3171, found: 465.3175.

2-((1*R*,4*R*)-3,5-dimethoxy-4-((*R*)-1-phenylallyl)-[1,1'-bi(cyclohexane)]-2,5-dien-1-yl)-4,4,5,5-tetramethyl-1,3,2-dioxaborolane (4a-major)

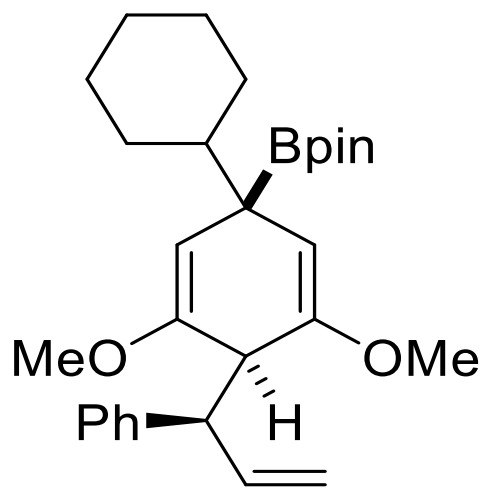

4a-major

Prepared following **General Procedure A** using boronic ester **1a** (42.0 mg, 0.200 mmol, 1.00 equiv) and carbonate **3a** (93.7 mg, 0.400 mmol, 2.00 equiv) without oxidation. The crude mixture was purified by flash column chromatography (hexane/toluene = 2:1 to 1:1) gave the title compound (27.9 mg, 30%) as a white solid.

R_f = 0.10 (2:1 hexane/toluene, UV, $KMnO_4$)

^{1}H NMR (400 MHz, $CDCl_3$) δ 7.21 – 7.15 (m, 2H), 7.14 – 7.08 (m, 1H), 7.07 – 7.01 (m, 2H), 6.59 – 6.45 (m, 1H), 5.08 – 4.95 (m, 2H), 4.58 (s, 1H), 4.55 (s, 1H), 4.03 (dd, *J* = 8.8, 2.8 Hz, 1H), 3.57 (d, *J* = 2.9 Hz, 6H), 3.28 (d, *J* = 2.7 Hz, 1H), 1.54 – 1.44 (m, 3H), 1.28 – 1.12 (m, 14H), 1.02 – 0.83 (m, 3H), 0.67 – 0.50 (m, 3H) ppm. **^{13}C NMR** (126 MHz, $CDCl_3$) δ 151.9, 151.3, 142.6, 142.0, 129.3, 127.8, 126.1, 114.0, 98.7, 98.5, 83.1, 54.3, 54.0, 51.4, 48.9, 45.7, 29.3, 29.3, 27.0, 26.9, 26.9, 24.8 ppm.

Specific rotation $[\alpha]_D^{24}$ = +8 (c = 2.0, CH_2Cl_2).

HRMS (ESI) m/z calculated for $C_{29}H_{42}BO_4$ $[M+H]^+$, 465.3171, found: 465.3186.

2.6. Characterization Data of Reaction Products

(*S*)-5-cyclohexyl-1,3-dimethoxy-2-(1-phenylallyl)benzene (5a)

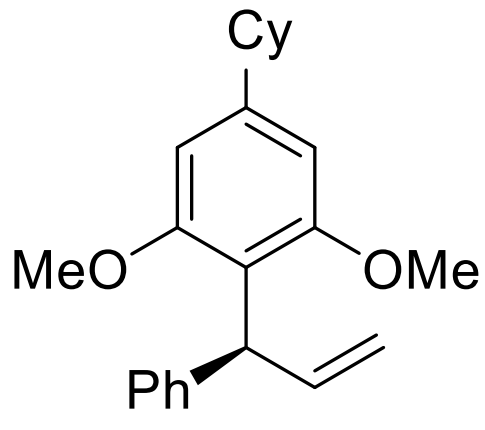

5a

Prepared following **General Procedure A** using boronic ester **1a** (42.0 mg, 0.200 mmol, 1.00 equiv) and carbonate **3a** (93.7 mg, 0.400 mmol, 2.00 equiv). Purification by flash column chromatography (hexane/toluene = 5:1) gave the title compound (52.0 mg, 77%, >99% ee) as a white solid.

R*f* = 0.63 (2:1 hexane/toluene, UV, $KMnO_4$)

^{1}H NMR (400 MHz, $CDCl_3$) δ 7.25 – 7.19 (m, 4H), 7.16 – 7.10 (m, 1H), 6.60 (ddd, *J* = 17.0, 10.0, 8.4 Hz, 1H), 6.42 (s, 2H), 5.34 (d, *J* = 8.4 Hz, 1H), 5.21 – 5.08 (m, 2H), 3.71 (s, 6H), 2.53 – 2.41 (m, 1H), 1.95 – 1.81 (m, 4H), 1.81 – 1.70 (m, 1H), 1.50 – 1.33 (m, 4H), 1.32 – 1.19 (m, 1H) ppm.**^{13}C NMR** (101 MHz, $CDCl_3$) δ 158.0, 148.3, 144.5, 140.0, 127.8, 127.7, 125.3, 117.8, 115.5, 103.6, 55.9, 45.2, 44.5, 34.6, 27.1, 26.3 ppm.

Specific rotation $[\alpha]_D^{24}$ = −38 (c = 2.0, CH_2Cl_2).

HRMS (ESI) m/z calculated for $C_{23}H_{29}O_2$ $[M+H]^+$, 337.2162, found: 337.2150.

The enantiomeric excess (ee) was determined using the corresponding alcohol which was prepared following **General Procedure B**. **HPLC conditions**: Chiral column IA, hexane: isopropanol = 95:5, flow rate = 1.0 mL/min, wavelength = 230 nm, t_R = 10.2 min for major isomer, t_R = 9.7 min for minor isomer.

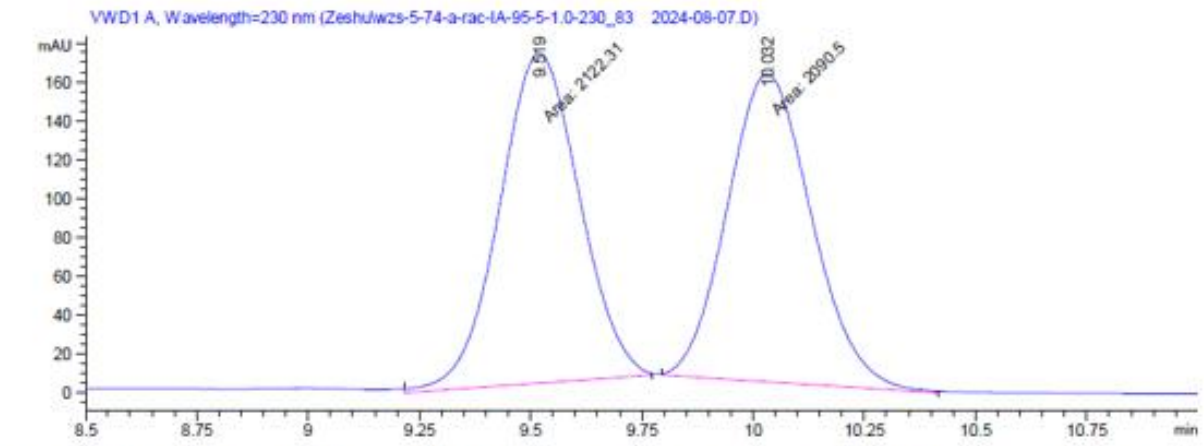

Peak #	RetTime [min]	Type	Width [min]	Area [mAU*s]	Height [mAU]	Area %
1	9.519	MM	0.2082	2122.30811	169.91417	50.3775
2	10.032	MM	0.2198	2090.50122	158.52126	49.6225

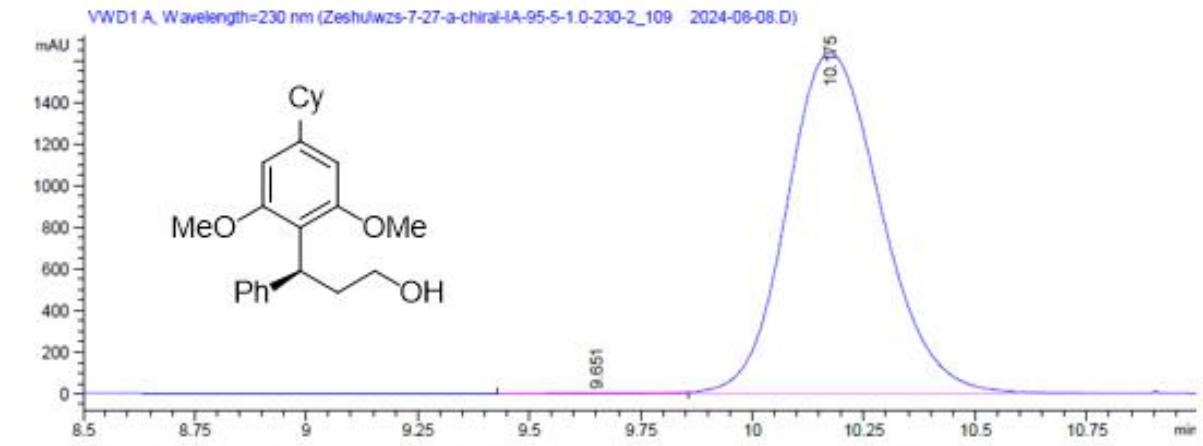

Peak #	RetTime [min]	Type	Width [min]	Area [mAU*s]	Height [mAU]	Area %
1	9.651	BV E	0.1876	26.63377	1.96101	0.1104
2	10.175	VV R	0.2301	2.40975e4	1635.18372	99.8896

(*S*)-5-cyclohexyl-1,3-dimethoxy-2-(1-(4-methoxyphenyl)allyl)benzene (5b)

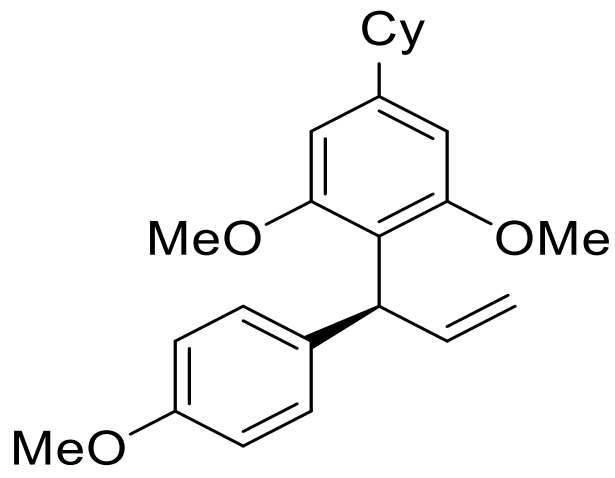

5b

Prepared following **General Procedure A** using boronic ester **1a** (42.0 mg, 0.200 mmol, 1.00 equiv) and allyl carbonate **3b** (106 mg, 0.400 mmol, 2.00 equiv). Purification by flash column chromatography (hexane/toluene = 3:1 to 1:1) gave the title compound (56.4 mg, 77%, >99% ee) as a pale-yellow solid.

R*f* = 0.36 (2:1 hexane/toluene, UV, $KMnO_4$)

^{1}H NMR (400 MHz, $CDCl_3$) δ 7.19 – 7.10 (m, 2H), 6.81 – 6.73 (m, 2H), 6.57 (ddd, *J* = 17.1, 9.9, 8.2 Hz, 1H), 6.41 (s, 2H), 5.26 (d, *J* = 8.3 Hz, 1H), 5.19 – 5.03 (m, 2H), 3.76 (s, 3H), 3.72 (s, 6H), 2.51 – 2.38 (m, 1H), 1.95 – 1.79 (m, 4H), 1.79 – 1.69 (m, 1H), 1.48 – 1.31 (m, 4H), 1.32 – 1.16 (m, 1H) ppm. **^{13}C NMR** (101 MHz, $CDCl_3$) δ 158.0, 157.5, 148.2, 140.5, 136.6, 128.7, 118.0, 115.1, 113.2, 103.7, 56.0, 55.3, 45.2, 43.8, 34.6, 27.1, 26.3 ppm.

Specific rotation $[\alpha]_D^{24} = -30$ (c = 2.0, CH_2Cl_2).

HRMS (ESI) m/z calculated for $C_{24}H_{31}O_3$ $[M+H]^+$, 367.2268, found: 367.2254.

HPLC conditions: Chiral column IC, hexane: isopropanol = 98:2, flow rate = 1.0 mL/min, wavelength = 254 nm, t_R = 4.4 min for major isomer, t_R = 4.8 min for minor isomer.

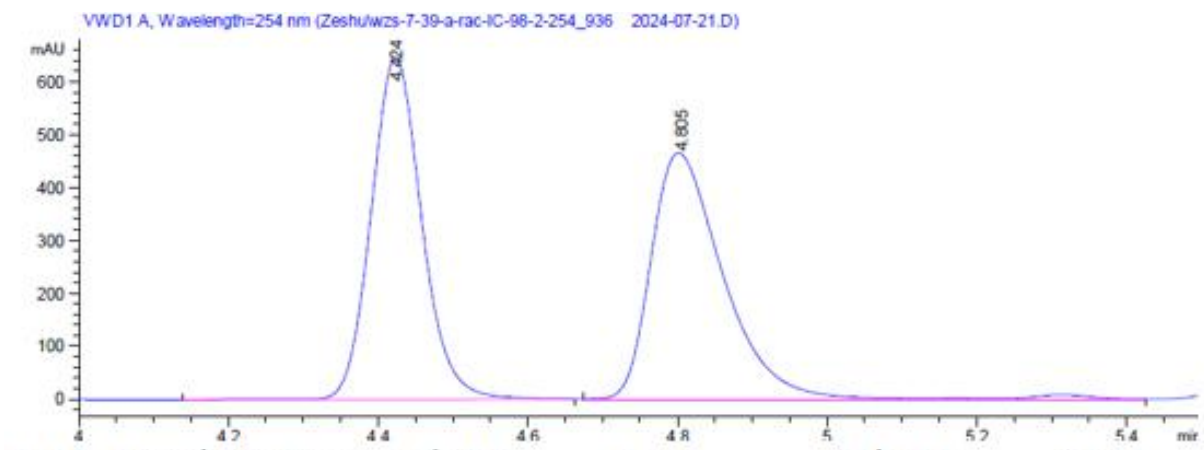

Peak #	RetTime [min]	Type	Width [min]	Area [mAU*s]	Height [mAU]	Area %
1	4.424	BB	0.0754	3036.61621	643.92889	49.2172
2	4.805	BV R	0.1047	3133.20557	459.93768	50.7828

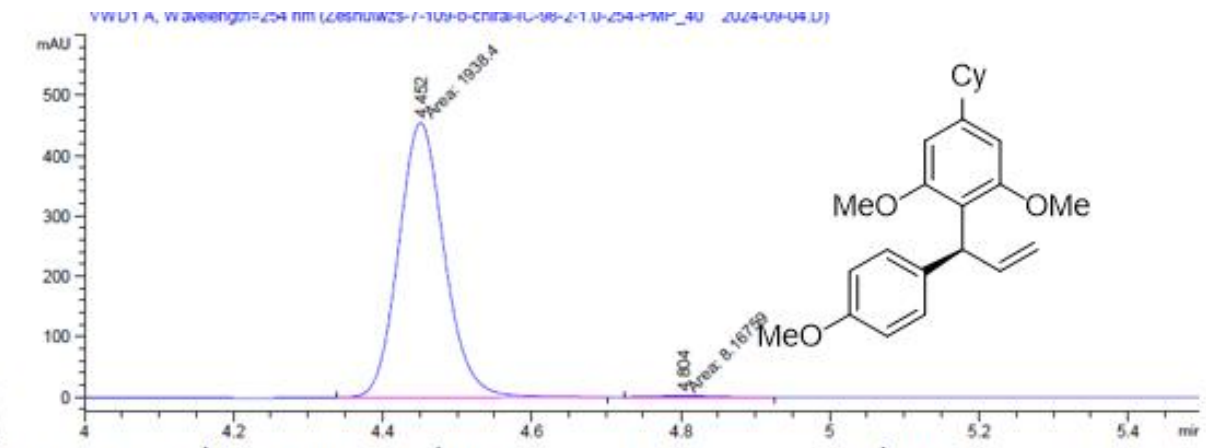

Peak #	RetTime [min]	Type	Width [min]	Area [mAU*s]	Height [mAU]	Area %
1	4.452	MM	0.0708	1938.39954	456.39856	99.5804
2	4.804	MM	0.0888	8.16759	1.53266	0.4196

(*S*)-2-(1-(4-bromophenyl)allyl)-5-cyclohexyl-1,3-dimethoxybenzene (5c)

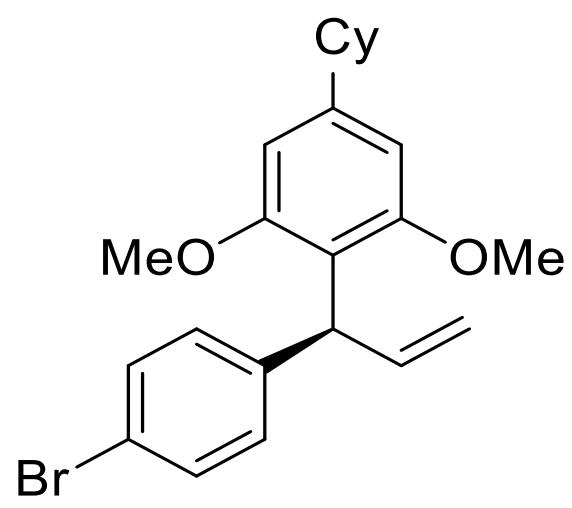

5c

Prepared following **General Procedure A** using boronic ester **1a** (42.0 mg, 0.200 mmol, 1.00 equiv) and carbonate **3c** (125 mg, 0.400 mmol, 2.00 equiv). Purification by flash column chromatography (hexane/toluene = 6:1) gave the title compound (55.7 mg, 67%, >99% ee) as a white solid.

R*f* = 0.69 (2:1 hexane/toluene, UV, $KMnO_4$)

^{1}H NMR (400 MHz, $CDCl_3$) δ 7.35 – 7.30 (m, 2H), 7.13 – 7.06 (m, 2H), 6.52 (ddd, *J* = 17.1, 10.0, 8.3 Hz, 1H), 6.41 (s, 2H), 5.25 (d, *J* = 8.3 Hz, 1H), 5.19 – 5.08 (m, 2H), 3.71 (s, 6H), 2.50 – 2.40 (m, 1H), 1.93 – 1.81 (m, 4H), 1.80 – 1.71 (m, 1H), 1.49 – 1.32 (m, 4H), 1.32 – 1.19 (m, 1H) ppm. **^{13}C NMR** (101 MHz, $CDCl_3$) δ 157.9, 148.6, 143.6, 139.4, 130.8, 129.5, 129.2, 128.4, 119.1, 117.1, 115.9, 103.6, 55.9, 45.3, 43.9, 34.6, 27.0, 26.3 ppm.

Specific rotation $[\alpha]_D^{24}$ = −26 (c = 2.0, CH_2Cl_2).

HRMS (ESI) m/z calculated for $C_{23}H_{28}BrO_2$ $[M+H]^+$, 415.1267, found: 415.1272.

The enantiomeric excess (ee) was determined using the corresponding alcohol which was prepared following **General Procedure B**. **HPLC conditions**: Chiral column IC, hexane: isopropanol = 98:2, flow rate = 1.0 mL/min, wavelength = 230 nm, t_R = 28.4 min for major isomer, t_R = 24.0 min for minor isomer.

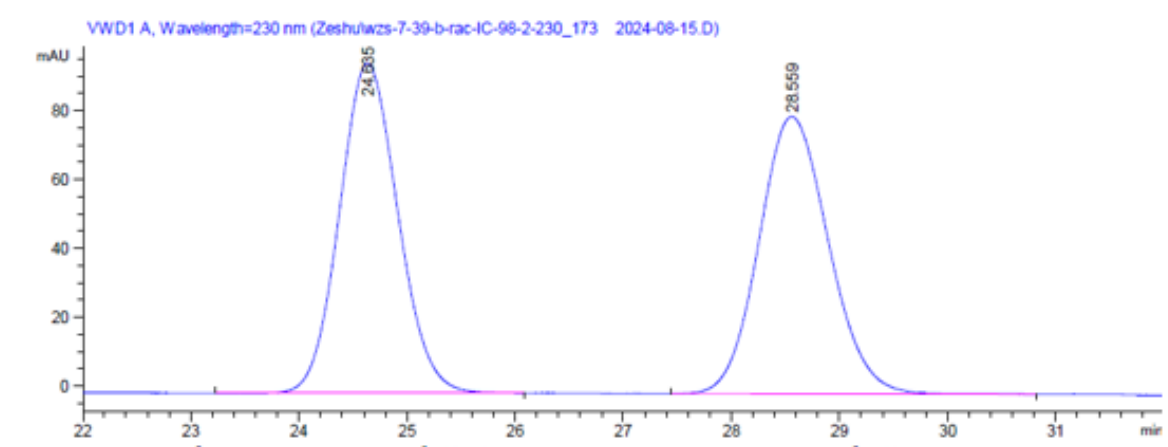

Peak #	RetTime [min]	Type	Width [min]	Area [mAU*s]	Height [mAU]	Area %
1	24.635	BB	0.5848	3607.32593	95.81425	49.9642
2	28.559	BB	0.7008	3612.49463	80.55209	50.0358

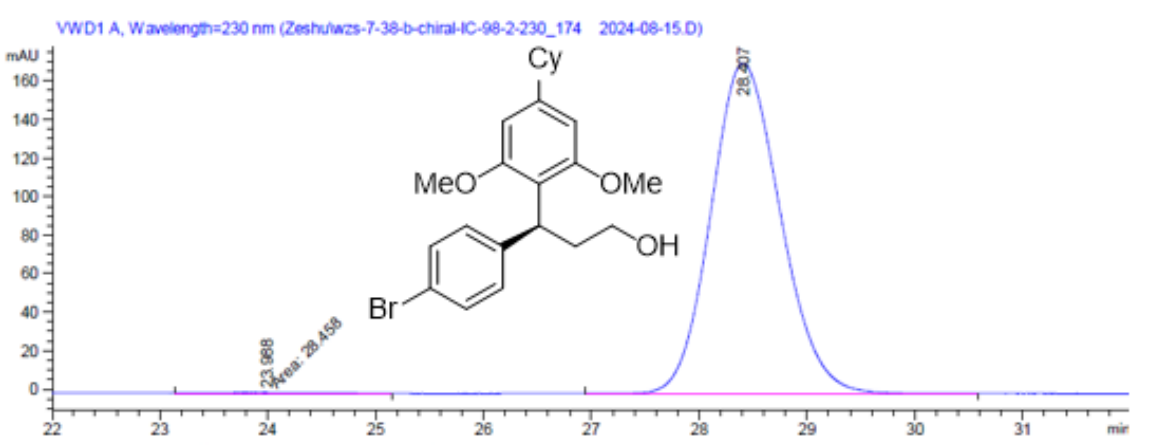

Peak #	RetTime [min]	Type	Width [min]	Area [mAU*s]	Height [mAU]	Area %
1	23.988	MM	1.2399	28.45798	3.82541e-1	0.3693
2	28.407	BB	0.6973	7677.84814	171.40788	99.6307

(*S*)-5-cyclohexyl-1,3-dimethoxy-2-(1-(4-(trifluoromethyl)phenyl)allyl)benzene (5d)

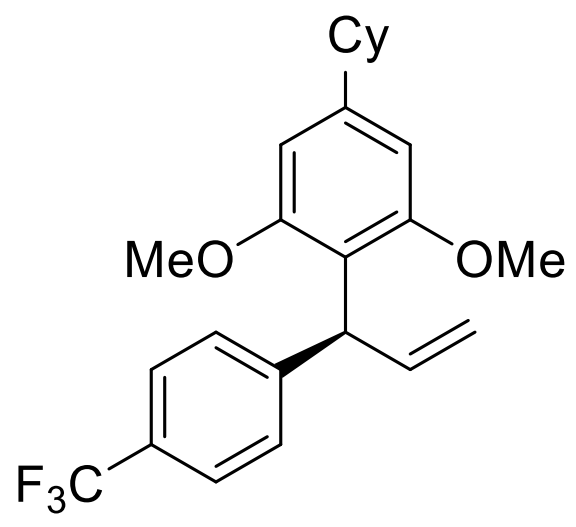

5d

Prepared following **General Procedure A** using boronic ester **1a** (42.0 mg, 0.200 mmol, 1.00 equiv) and carbonate **3d** (121 mg, 0.400 mmol, 2.00 equiv). Purification by flash column chromatography (hexane/toluene = 6:1) gave the title compound (51.8 mg, 64%, >99% ee) as a pale-yellow solid.

R_f = 0.78 (2:1 hexane/toluene, UV, $KMnO_4$)

^{1}H NMR (500 MHz, $CDCl_3$) δ 7.47 (d, *J* = 8.7 Hz, 2H), 7.33 (d, *J* = 8.8 Hz, 2H), 6.54 (ddd, *J* = 17.0, 10.0, 8.3 Hz, 1H), 6.42 (s, 2H), 5.35 (d, *J* = 8.4 Hz, 1H), 5.21 – 5.12 (m, 2H), 3.71 (s, 6H), 2.53 – 2.42 (m, 1H), 1.94 – 1.80 (m, 4H), 1.80 – 1.69 (m, 1H), 1.51 – 1.33 (m, 4H), 1.32 – 1.19 (m, 1H) ppm. **^{13}C NMR** (126 MHz, $CDCl_3$) δ 157.9, 148.8, 128.8 (q, *J* = 1.3 Hz), 139.0, 127.9, 127.6 (q, *J* = 31.3 Hz), 124.7 (q, *J* = 3.8 Hz), 124.7 (q, *J* = 270.0 Hz), 116.8, 116.3, 103.5, 55.8, 45.3, 44.3, 34.6, 27.0, 26.3 ppm. **^{19}F NMR** (376 MHz, $CDCl_3$) δ −62.0 (s).

Specific rotation $[\alpha]_D^{24}$ = −48 (c = 2.0, CH_2Cl_2).

HRMS (ESI) m/z calculated for $C_{24}H_{28}F_3O_2$ $[M+H]^+$, 405.2036, found: 405.2042.

The enantiomeric excess (ee) was determined using the corresponding alcohol which was prepared following **General Procedure B**. **HPLC conditions**: Chiral column IC, hexane: isopropanol = 98:2, flow rate = 1.0 mL/min, wavelength = 230 nm, t_R = 16.3 min for major isomer, t_R = 14.2 min for minor isomer.

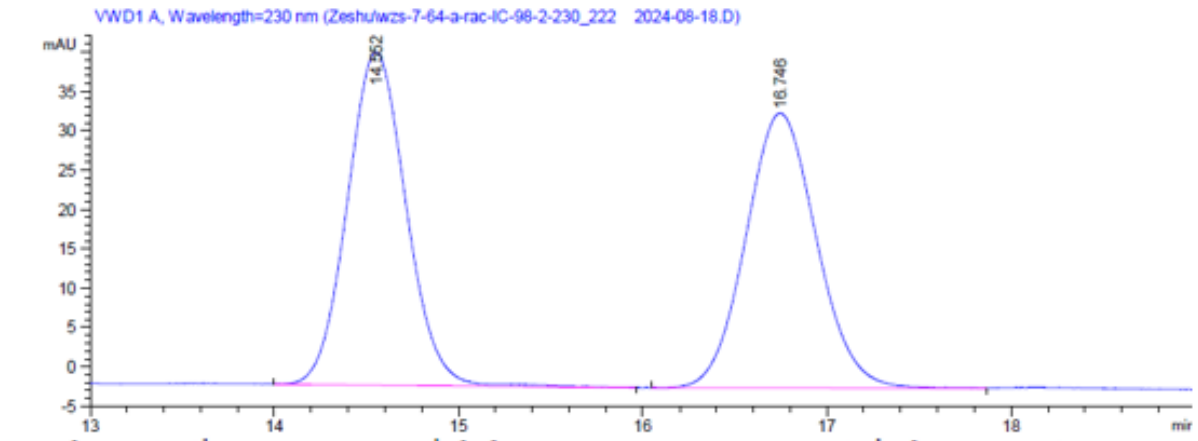

Peak #	RetTime [min]	Type	Width [min]	Area [mAU*s]	Height [mAU]	Area %
1	14.552	BB	0.3467	939.58679	42.27063	50.2755
2	16.746	BB	0.4157	929.28754	34.87996	49.7245

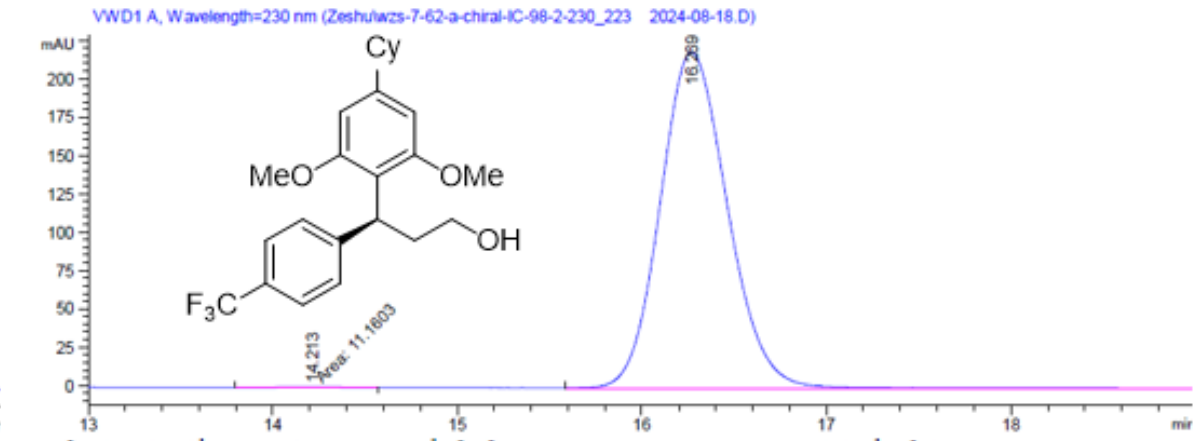

Peak #	RetTime [min]	Type	Width [min]	Area [mAU*s]	Height [mAU]	Area %
1	14.213	MM	0.3628	11.16030	5.12706e-1	0.2004
2	16.269	VB	0.3923	5559.10547	218.95676	99.7996

(*S*)-2-(1-(3-chlorophenyl)allyl)-5-cyclohexyl-1,3-dimethoxybenzene (5e)

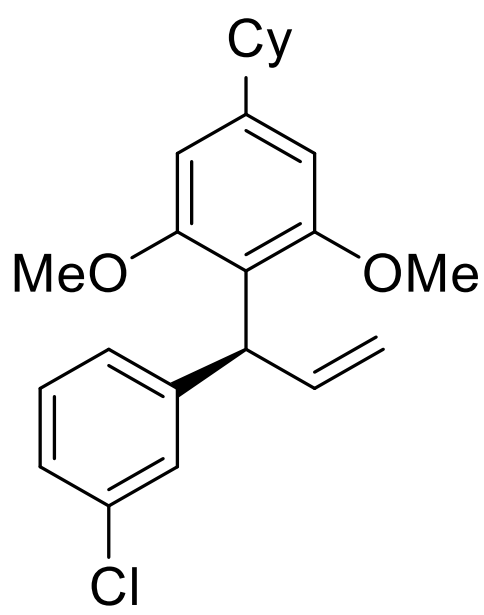

5e

Prepared following **General Procedure A** using boronic ester **1a** (42.0 mg, 0.200 mmol, 1.00 equiv) and carbonate **3e** (107 mg, 0.400 mmol, 2.00 equiv). Purification by flash column chromatography (hexane/toluene = 8:1) gave the title compound (44.7 mg, 60%, >99% ee) as a pale yellow solid.

R*f* = 0.74 (2:1 hexane/toluene, UV, $KMnO_4$)

^{1}H NMR (500 MHz, $CDCl_3$) δ 7.25 – 7.21 (m, 1H), 7.18 – 7.08 (m, 3H), 6.53 (ddd, *J* = 17.0, 10.0, 8.4 Hz, 1H), 6.42 (s, 2H), 5.29 (d, *J* = 8.4 Hz, 1H), 5.22 – 5.11 (m, 2H), 3.72 (s, 6H), 2.53 – 2.43 (m, 1H), 1.95 – 1.82 (m, 4H), 1.81 – 1.72 (m, 1H), 1.50 – 1.36 (m, 4H), 1.32 – 1.22 (m, 1H) ppm. **^{13}C NMR** (126 MHz, $CDCl_3$) δ 157.9, 148.7, 146.8, 139.1, 133.6, 128.9, 127.9, 125.9, 125.5, 116.9, 116.1, 103.6, 55.9, 45.3, 44.2, 34.6, 34.6, 27.1, 26.3 ppm.

Specific rotation $[\alpha]_D^{24}$ = −53 (c = 2.0, CH_2Cl_2).

HRMS (ESI) m/z calculated for $C_{23}H_{28}ClO_2$ $[M+H]^+$, 371.1772, found: 371.1758.

The enantiomeric excess (ee) was determined using the corresponding alcohol which was prepared following **General Procedure B**. **HPLC conditions**: Chiral column IC, hexane:

isopropanol = 95:5, flow rate = 1.0 mL/min, wavelength = 230 nm, t_R = 19.9 min for major isomer, t_R = 13.0 min for minor isomer.

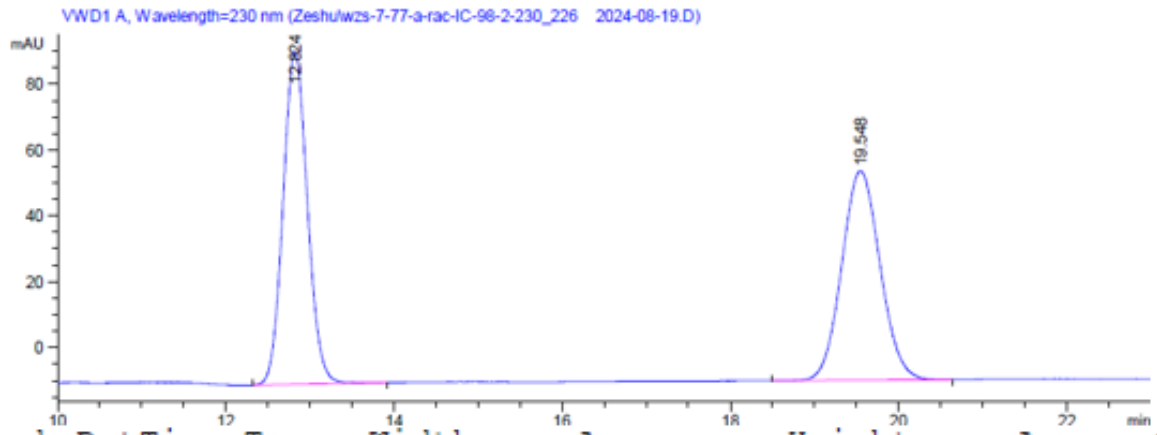

Peak #	RetTime [min]	Type	Width [min]	Area [mAU*s]	Height [mAU]	Area %
1	12.824	BB	0.3135	2032.51099	100.96693	50.1988
2	19.548	BB	0.4958	2016.40991	63.48784	49.8012

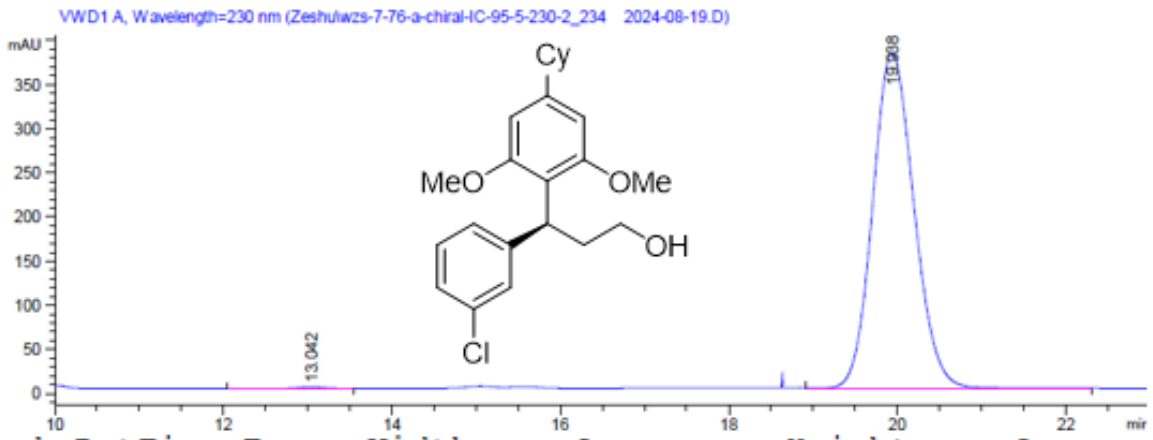

Peak #	RetTime [min]	Type	Width [min]	Area [mAU*s]	Height [mAU]	Area %
1	13.042	VB R	0.3440	39.84793	1.75182	0.3080
2	19.938	BB	0.5268	1.28999e4	380.23288	99.6920

(*S*)-5-cyclohexyl-1,3-dimethoxy-2-(1-(o-tolyl)allyl)benzene (5f)

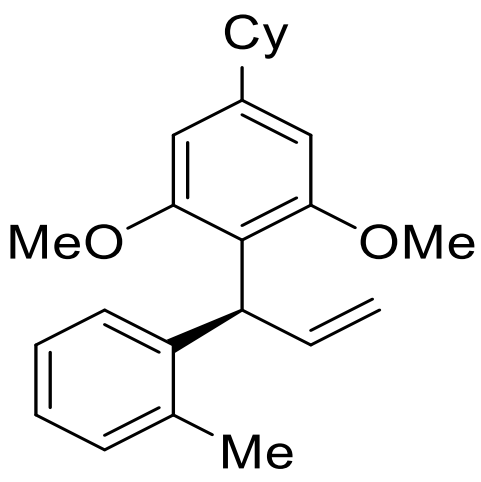

5f

Prepared following **General Procedure A** using boronic ester **1a** (42.0 mg, 0.200 mmol, 1.00 equiv) and carbonate **3f** (99.3 mg, 0.400 mmol, 2.00 equiv). Purification by flash column chromatography (hexane/toluene = 8:1) gave the title compound (44.1 mg, 63%, >99% ee) as a pale-yellow oil.

R_f = 0.57 (2:1 hexane/toluene, UV, $KMnO_4$)

^{1}H NMR (500 MHz, $CDCl_3$) δ 7.43 (d, J = 7.7 Hz, 1H), 7.18 – 7.10 (m, 1H), 7.10 – 7.04 (m, 2H), 6.50 (ddd, J = 17.1, 10.1, 7.1 Hz, 1H), 6.41 (s, 2H), 5.35 (d, J = 7.4 Hz, 1H), 5.07 (dt, J = 10.1, 1.6 Hz, 1H), 4.96 (dt, J = 17.1, 1.8 Hz, 1H), 3.70 (s, 6H), 2.51 – 2.42 (m, 1H), 2.23 (s, 3H), 1.95 – 1.83 (m, 4H), 1.81 – 1.71 (m, 1H), 1.47 – 1.35 (m, 4H), 1.33 – 1.24 (m, 1H) ppm.
^{13}C NMR (126 MHz, $CDCl_3$) δ 158.3, 148.3, 142.5, 140.4, 136.5, 129.9, 129.2, 125.6, 125.2, 117.2, 113.9, 103.8, 55.9, 45.2, 42.3, 34.6, 34.6, 27.0, 26.3, 19.6 ppm.

Specific rotation $[\alpha]_D^{24}$ = −41 (c = 2.0, CH_2Cl_2).

HRMS (ESI) m/z calculated for $C_{24}H_{31}O_2$ $[M+H]^+$, 351.2319, found: 351.2315.

The enantiomeric excess (ee) was determined using the corresponding alcohol which was prepared following **General Procedure B**. **HPLC conditions**: Chiral column IA, hexane: isopropanol = 95:5, flow rate = 1.0 mL/min, wavelength = 230 nm, t_R = 9.9 min for major isomer, t_R = 9.3 min for minor isomer.

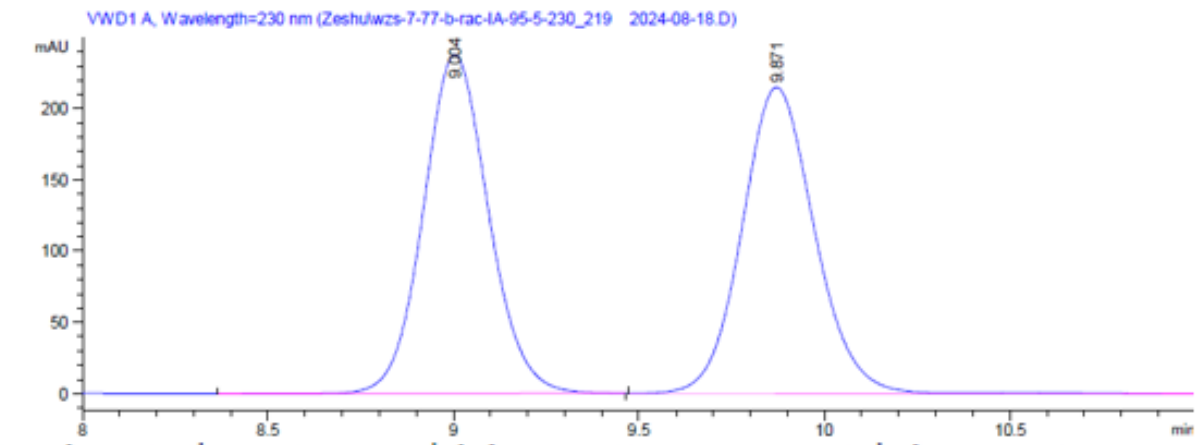

Peak #	RetTime [min]	Type	Width [min]	Area [mAU*s]	Height [mAU]	Area %
1	9.004	BB	0.1894	2911.45483	237.79192	49.7458
2	9.871	BV R	0.2108	2941.20483	214.85950	50.2542

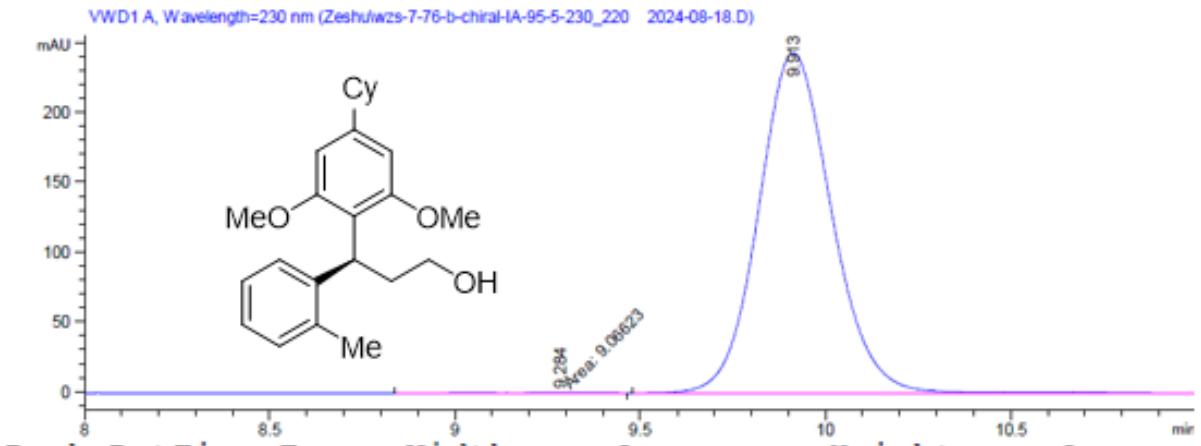

Peak #	RetTime [min]	Type	Width [min]	Area [mAU*s]	Height [mAU]	Area %
1	9.284	MM	0.3728	9.06623	4.05308e-1	0.2700
2	9.913	BV R	0.2117	3349.23926	243.33057	99.7300

(*S*)-5-cyclohexyl-1,3-dimethoxy-2-(1-(2-methoxyphenyl)allyl)benzene (5g)

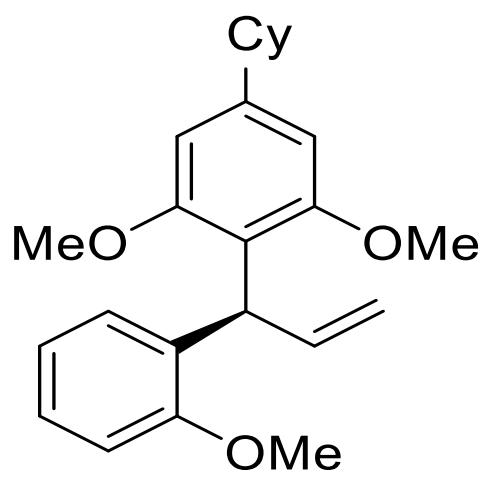

5g

Prepared following **General Procedure A** using boronic ester **1a** (42.0 mg, 0.200 mmol, 1.00 equiv) and carbonate **3g** (106 mg, 0.400 mmol, 2.00 equiv). Purification by flash column chromatography (hexane/toluene = 3:1 to 2:1) gave the title compound (47.7 mg, 65%, 98% ee) as a colourless oil.

R$_f$ = 0.5 (1:1 hexane/toluene, UV, $KMnO_4$)

^{1}H NMR (400 MHz, $CDCl_3$) δ 7.33 (dd, *J* = 7.6, 1.8 Hz, 1H), 7.18 – 7.09 (m, 1H), 6.92 – 6.84 (m, 1H), 6.81 (dd, *J* = 8.2, 1.2 Hz, 1H), 6.54 – 6.42 (m, 1H), 6.40 (s, 2H), 5.59 (d, *J* = 7.0 Hz, 1H), 5.07 – 4.93 (m, 2H), 3.72 (s, 3H), 3.72 (s, 6H), 2.51 – 2.39 (m, 1H), 1.93 – 1.80 (m, 4H), 1.79 – 1.71 (m, 1H), 1.48 – 1.34 (m, 4H), 1.32 – 1.23 (m, 1H) ppm. **^{13}C NMR** (101 MHz, $CDCl_3$) δ 158.5, 157.5, 147.9, 140.4, 132.6, 130.0, 126.7, 120.1, 117.8, 113.8, 111.0, 103.9, 56.1, 55.9, 45.1, 38.8, 34.6, 27.1, 26.4 ppm.

Specific rotation $[\alpha]_D^{24}$ = −8 (c = 3.0, CH_2Cl_2).

HRMS (ESI) m/z calculated for $C_{24}H_{31}O_3$ $[M+H]^+$, 367.2268, found: 367.2270.

HPLC conditions: Chiral column ADH, hexane: isopropanol = 99:1, flow rate = 1.0 mL/min, wavelength = 254 nm, t_R = 4.7 min for major isomer, t_R = 4.9 min for minor isomer.

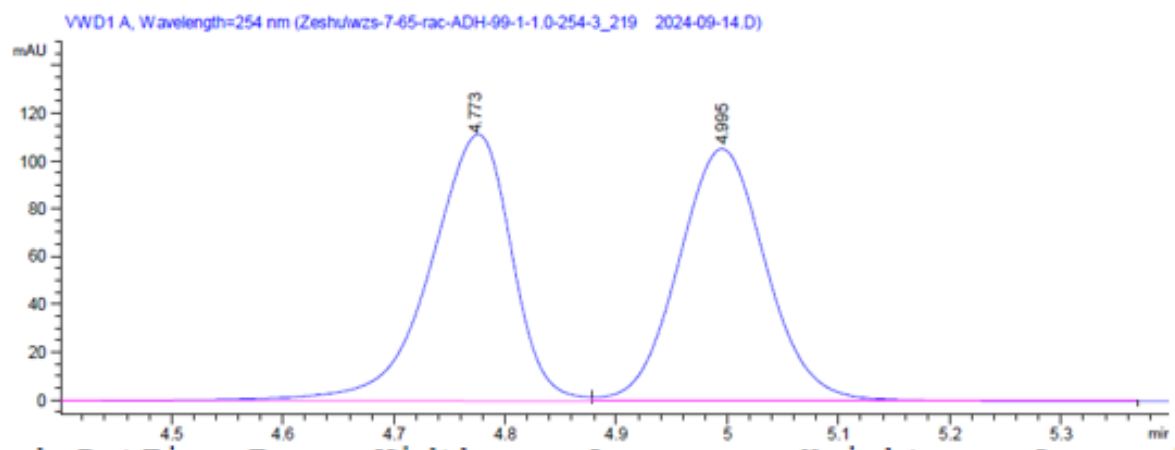

Peak #	RetTime [min]	Type	Width [min]	Area [mAU*s]	Height [mAU]	Area %
1	4.773	BV	0.0808	569.04456	109.93855	49.6293
2	4.995	VB	0.0874	577.54425	105.16238	50.3707

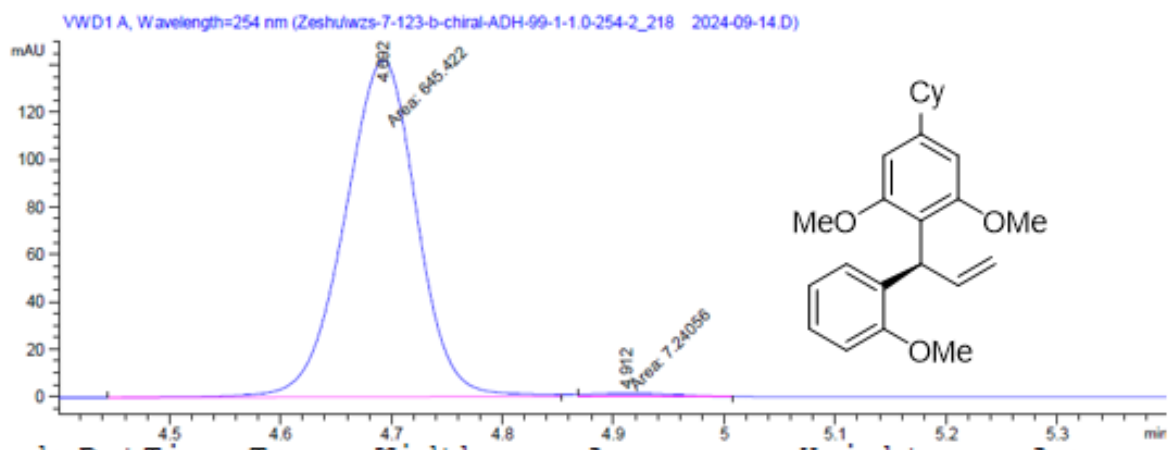

Peak #	RetTime [min]	Type	Width [min]	Area [mAU*s]	Height [mAU]	Area %
1	4.692	MM	0.0754	645.42206	142.66217	98.8906
2	4.912	MM	0.0851	7.24056	1.41839	1.1094

(*R*)-5-cyclohexyl-2-(1-(2-fluorophenyl)allyl)-1,3-dimethoxybenzene (5h)

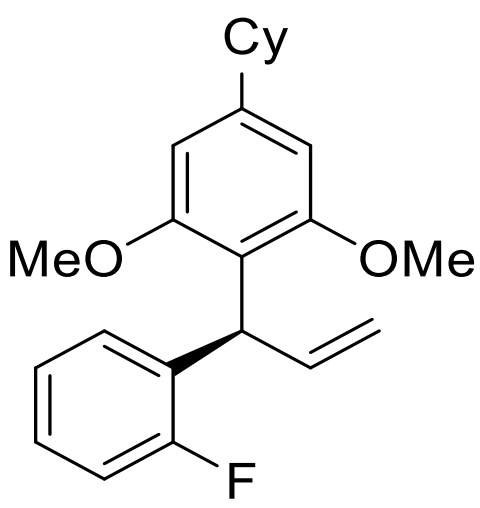

5h

Prepared following **General Procedure A** using boronic ester **1a** (42.0 mg, 0.200 mmol, 1.00 equiv) and carbonate **3h** (101 mg, 0.400 mmol, 2.00 equiv). Purification by flash column chromatography (hexane/toluene = 5:1) gave the title compound (55.2 mg, 78%, >99% ee) as a pale-yellow solid.

R_f = 0.63 (2:1 hexane/toluene, UV, $KMnO_4$)

^{1}H NMR (500 MHz, $CDCl_3$) δ 7.42 – 7.34 (m, 1H), 7.15 – 7.07 (m, 1H), 7.06 – 6.99 (m, 1H), 6.92 (ddd, J = 10.6, 8.1, 1.3 Hz, 1H), 6.56 – 6.45 (m, 1H), 6.40 (s, 2H), 5.56 (d, J = 7.3 Hz, 1H), 5.11 – 5.01 (m, 2H), 3.72 (s, 6H), 2.50 – 2.38 (m, 1H), 1.92 – 1.80 (m, 4H), 1.79 – 1.70 (m, 1H), 1.49 – 1.32 (m, 4H), 1.31 – 1.18 (m, 1H) ppm. **^{13}C NMR** (126 MHz, $CDCl_3$) δ 161.0 (d, J = 246.2 Hz), 158.2, 148.5, 139.1, 131.1 (d, J = 13.9 Hz), 130.6 (d, J = 4.6 Hz), 127.1 (d, J = 8.2 Hz), 123.2 (d, J = 3.3 Hz), 116.7, 114.9 (d, J = 22.3 Hz), 114.8, 103.8, 56.0, 45.2, 38.3, 38.3, 34.6, 27.1, 26.3 ppm. **^{19}F NMR** (376 MHz, $CDCl_3$) δ −116.4 (ddd, J = 10.4, 7.9, 5.1 Hz).

Specific rotation $[\alpha]_D^{24}$ = −58 (c = 2.0, CH_2Cl_2).

HRMS (ESI) m/z calculated for $C_{23}H_{28}FO_2$ $[M+H]^+$, 355.2068, found: 355.2054.

The enantiomeric excess (ee) was determined using the corresponding alcohol which was prepared following **General Procedure B**. **HPLC conditions**: Chiral column IA, hexane: isopropanol = 95:5, flow rate = 1.0 mL/min, wavelength = 230 nm, t_R = 9.6 min for major isomer, t_R = 10.3 min for minor isomer.

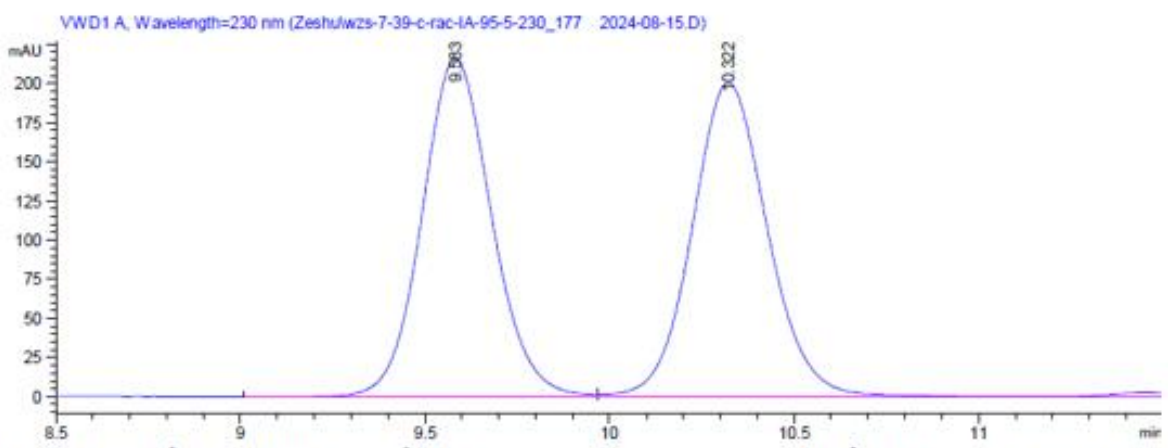

Peak #	RetTime [min]	Type	Width [min]	Area [mAU*s]	Height [mAU]	Area %
1	9.583	BV	0.2024	2828.57666	215.76709	49.3463
2	10.322	VV R	0.2218	2903.52100	200.52304	50.6537

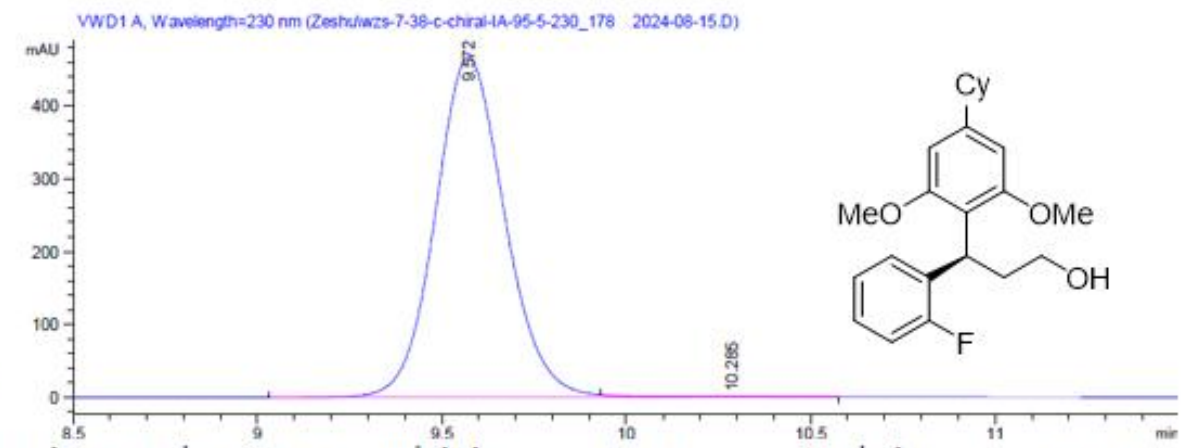

Peak #	RetTime [min]	Type	Width [min]	Area [mAU*s]	Height [mAU]	Area %
1	9.572	BV R	0.2023	6106.14502	466.22150	99.7091
2	10.285	VB E	0.2808	17.81223	9.10040e-1	0.2909

(*S*)-5-cyclohexyl-2-(1-mesitylallyl)-1,3-dimethoxybenzene (5i)

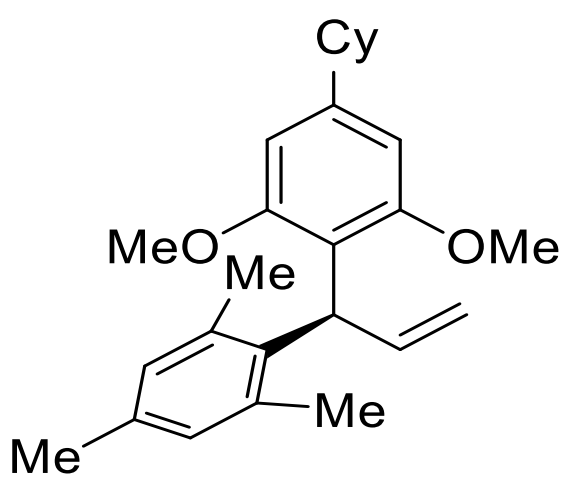

5i

Prepared following **General Procedure A** using boronic ester **1a** (42.0 mg, 0.200 mmol, 1.00 equiv) and carbonate **3i** (111 mg, 0.400 mmol, 2.00 equiv). Purification by flash column chromatography (hexane/toluene = 5:1) gave the title compound (23.5 mg, 31%, >99% ee) as a white solid.

$\mathbf{R}_f$ = 0.70 (2:1 hexane/toluene, UV, $KMnO_4$)

^{1}H NMR (400 MHz, $CDCl_3$) δ 6.75 (s, 2H), 6.50 (ddd, *J* = 16.8, 10.0, 6.7 Hz, 1H), 6.37 (s, 2H), 5.27 (d, *J* = 6.7 Hz, 1H), 4.84 (dt, *J* = 10.0, 1.9 Hz, 1H), 4.67 (dt, *J* = 17.0, 2.0 Hz, 1H), 3.66 (s, 6H), 2.50 – 2.36 (m, 1H), 2.24 (s, 6H), 2.21 (s, 3H), 1.91 – 1.78 (m, 4H), 1.78 – 1.69 (m, 1H), 1.43 – 1.32 (m, 4H), 1.31 – 1.20 (m, 1H) ppm. **^{13}C NMR** (101 MHz, $CDCl_3$) δ 158.7,

147.9, 140.9, 137.5, 137.4, 134.6, 129.6, 117.4, 110.2, 103.8, 55.9, 45.1, 43.6, 34.7, 34.6, 27.1, 26.3, 20.8, 20.7 ppm.

Specific rotation $[\alpha]_D^{24}$ = +130 (c = 2.0, CH_2Cl_2).

HRMS (ESI) m/z calculated for $C_{26}H_{35}O_2$ $[M+H]^+$, 379.2632, found: 379.2629.

The enantiomeric excess (ee) was determined using the corresponding alcohol which was prepared following **General Procedure B**. **HPLC conditions**: Chiral column IA, hexane: isopropanol = 100:0, flow rate = 1.0 mL/min, wavelength = 230 nm, t_R = 6.8 min for major isomer, t_R = 6.0 min for minor isomer.

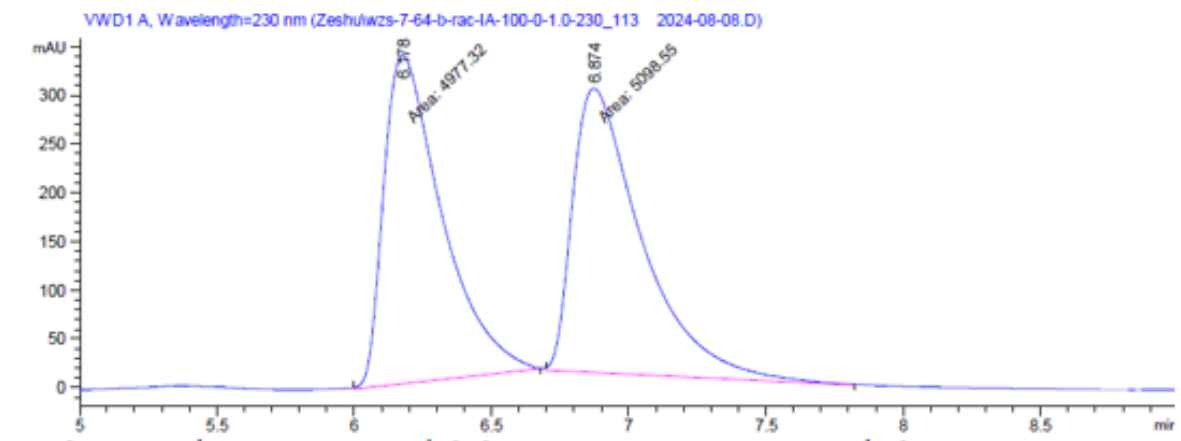

Peak #	RetTime [min]	Type	Width [min]	Area [mAU*s]	Height [mAU]	Area %
1	6.178	MM	0.2456	4977.32471	337.81735	49.3984
2	6.874	MM	0.2913	5098.54980	291.67923	50.6016

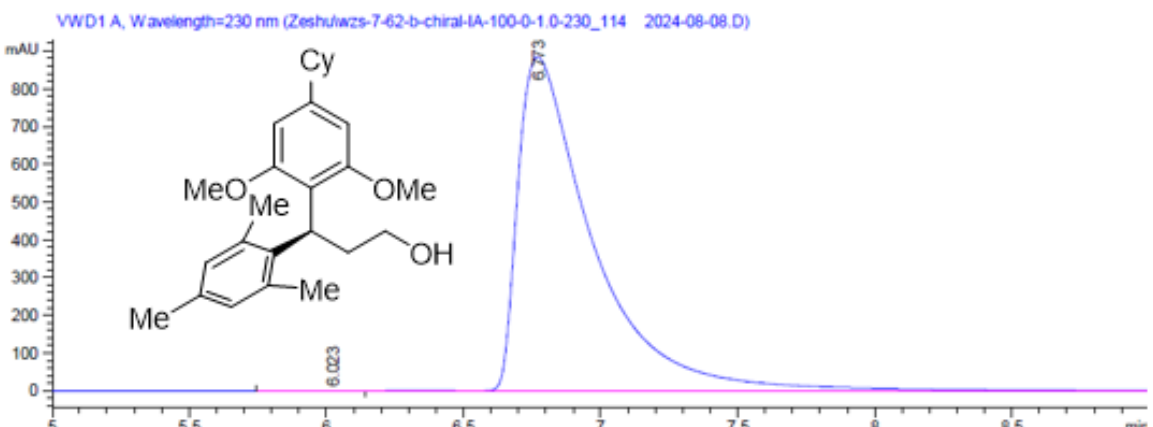

Peak #	RetTime [min]	Type	Width [min]	Area [mAU*s]	Height [mAU]	Area %
1	6.023	BV E	0.1866	9.21473	6.58211e-1	0.0536
2	6.773	VV R	0.2773	1.71922e4	885.96985	99.9464

(*R*)-2-(1-(4-cyclohexyl-2,6-dimethoxyphenyl)allyl)thiophene (5j)

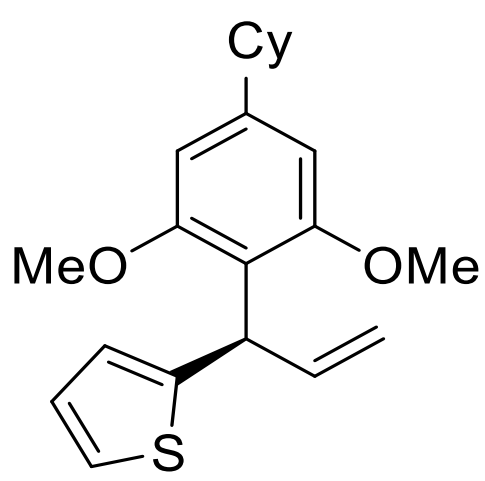

5j

Prepared following **General Procedure A** using boronic ester **1a** (42.0 mg, 0.200 mmol, 1.00 equiv) and carbonate **3j** (96.1 mg, 0.400 mmol, 2.00 equiv). Purification by flash column chromatography (hexane/toluene = 5:1) gave the title compound (46.6 mg, 68%, 98% ee) as a pale-yellow solid.

R_f = 0.73 (1:1 hexane/toluene, UV, Vanillin)

^{1}H NMR (400 MHz, $CDCl_3$) δ 7.07 (dd, J = 5.2, 1.3 Hz, 1H), 6.87 (dd, J = 5.1, 3.5 Hz, 1H), 6.79 – 6.73 (m, 1H), 6.61 (ddd, J = 17.0, 9.9, 8.0 Hz, 1H), 6.42 (s, 2H), 5.51 (d, J = 8.1 Hz,

1H), 5.18 (dt, J = 17.1, 1.5 Hz, 1H), 5.08 (ddd, J = 9.9, 1.8, 0.9 Hz, 1H), 3.77 (s, 6H), 2.53 – 2.40 (m, 1H), 1.95 – 1.80 (m, 4H), 1.80 – 1.69 (m, 1H), 1.50 – 1.32 (m, 4H), 1.32 – 1.14 (m, 1H) ppm. **^{13}C NMR** (126 MHz, $CDCl_3$) δ 157.9, 148.9, 148.7, 140.0, 126.3, 123.7, 123.0, 117.4, 115.2, 103.6, 55.9, 45.3, 40.7, 34.6, 27.1, 26.3 ppm.

Specific rotation $[\alpha]_D^{24} = -38$ (c = 2.0, CH_2Cl_2).

HRMS (ESI) m/z calculated for $C_{21}H_{27}O_2S$ $[M+H]^+$, 343.1726, found: 343.1721.

The enantiomeric excess (ee) was determined using the corresponding alcohol which was prepared following **General Procedure B**. **HPLC conditions**: Chiral column IB, hexane: isopropanol = 95:5, flow rate = 1.0 mL/min, wavelength = 230 nm, t_R = 9.8 min for major isomer, t_R = 8.8 min for minor isomer.

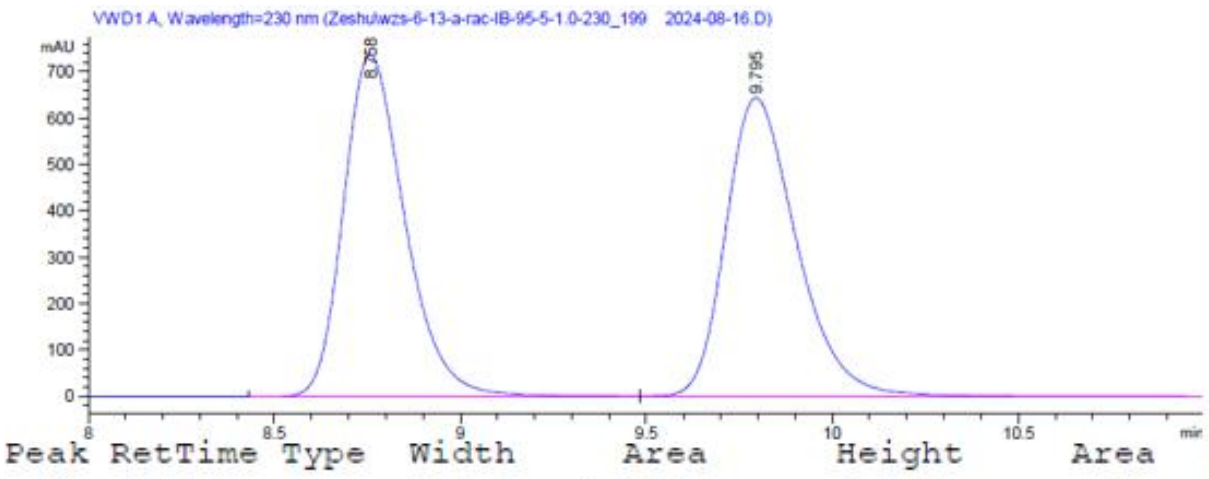

Peak #	RetTime [min]	Type	Width [min]	Area [mAU*s]	Height [mAU]	Area %
1	8.758	BV	0.1782	8492.95801	736.51538	49.6311
2	9.795	VV R	0.2057	8619.20996	643.64484	50.3689

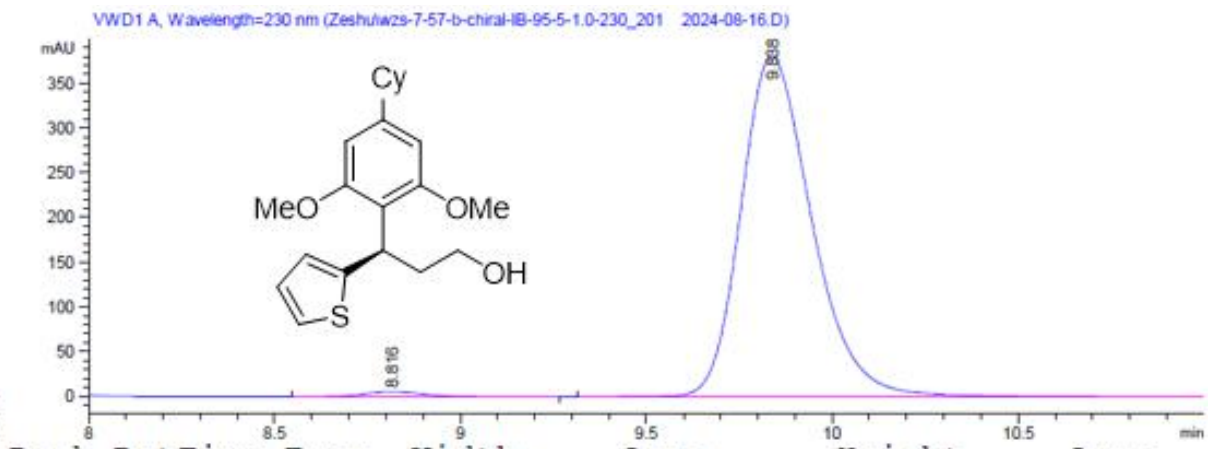

Peak #	RetTime [min]	Type	Width [min]	Area [mAU*s]	Height [mAU]	Area %
1	8.816	BB	0.1813	60.88668	5.16112	1.1829
2	9.838	BV R	0.2051	5086.34375	380.78503	98.8171

(*S*)-3-(1-(4-cyclohexyl-2,6-dimethoxyphenyl)allyl)-1-tosyl-1*H*-indole (5k)

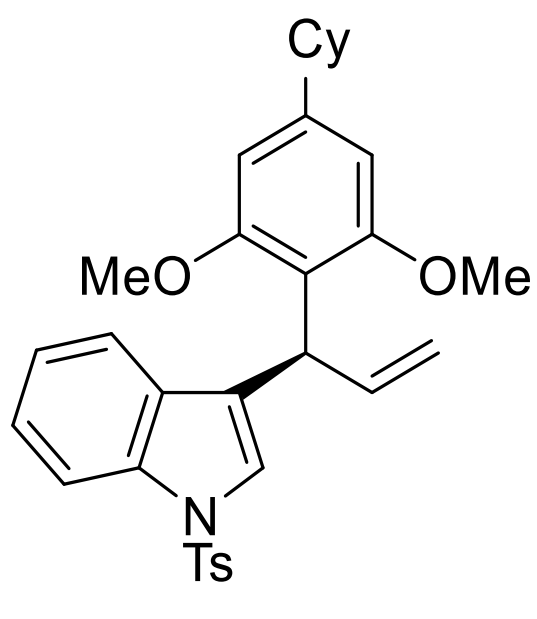

5k

Prepared following a modified **General Procedure A** using boronic ester **1a** (42.0 mg, 0.200 mmol, 1.00 equiv) and carbonate **3k** (171 mg, 0.400 mmol, 2.00 equiv) in which a solvent switch from THF to 1,4-dioxane was performed before **Step 2** of the reaction. Purification by flash column chromatography (hexane/EtOAc = 10:1) gave the title compound (62.5 mg, 59%, 99% ee) as a pale-yellow oil.

R$_f$ = 0.63 (5:1 hexane/EtOAc, UV, Vanillin)

^{1}H NMR (500 MHz, $CDCl_3$) δ 7.92 (d, *J* = 8.2 Hz, 1H), 7.71 (d, *J* = 8.5 Hz, 2H), 7.39 – 7.33 (m, 2H), 7.23 – 7.14 (m, 3H), 7.11 – 7.05 (m, 1H), 6.54 (ddd, *J* = 17.4, 10.0, 7.8 Hz, 1H), 6.38 (s, 2H), 5.42 (dd, *J* = 7.9, 1.4 Hz, 1H), 5.16 (dt, *J* = 17.1, 1.6 Hz, 1H), 5.09 (ddd, *J* = 10.0, 1.8, 1.0 Hz, 1H), 3.71 (s, 6H), 2.50 – 2.39 (m, 1H), 2.32 (s, 3H), 1.91 – 1.80 (m, 4H), 1.78 – 1.70 (m, 1H), 1.46 – 1.33 (m, 4H), 1.30 – 1.21 (m, 1H) ppm. **^{13}C NMR** (126 MHz, $CDCl_3$) δ 158.0, 148.6, 144.6, 138.9, 135.7, 135.3, 131.3, 129.8, 126.8, 125.4, 124.1, 123.9, 122.9, 120.5, 115.8, 115.3, 113.6, 103.5, 55.8, 45.3, 36.3, 34.6, 27.0, 26.3, 21.7 ppm.

Specific rotation $[\alpha]_D^{24}$ = −53 (c = 3.0, CH_2Cl_2).

HRMS (ESI) m/z calculated for $C_{32}H_{36}NO_4S$ $[M+H]^+$, 530.2360, found: 530.2369.

HPLC conditions: Chiral column IA, hexane: isopropanol = 98:2, flow rate = 1.0 mL/min, wavelength = 254 nm, t_R = 9.1 min for major isomer, t_R = 8.6 min for minor isomer.

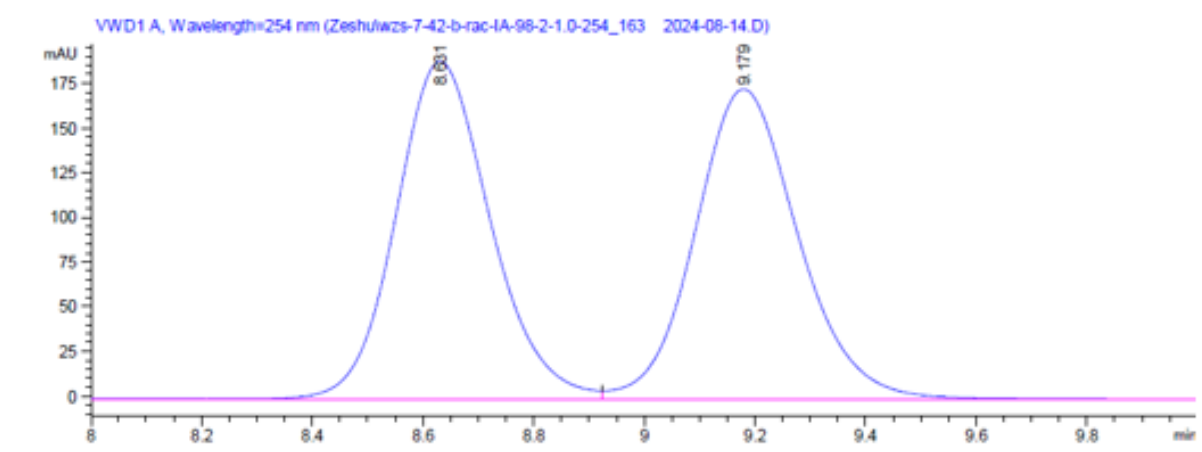

Peak #	RetTime [min]	Type	Width [min]	Area [mAU*s]	Height [mAU]	Area %
1	8.631	BV	0.1830	2252.17676	188.64276	49.9089
2	9.179	VV R	0.2017	2260.40088	173.29817	50.0911

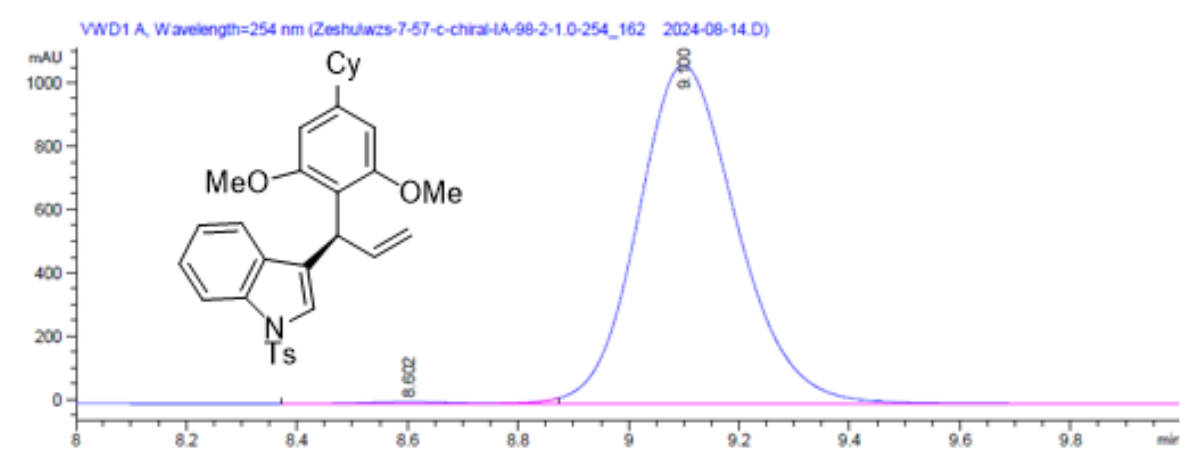

Peak #	RetTime [min]	Type	Width [min]	Area [mAU*s]	Height [mAU]	Area %
1	8.602	BV E	0.1709	68.23932	6.11795	0.5050
2	9.100	VB R	0.1962	1.34447e4	1068.91418	99.4950

1,3-Dimethoxy-2-((*S*)-1-phenylallyl)-5-((*R*)-4-phenylbutan-2-yl)benzene (5m)

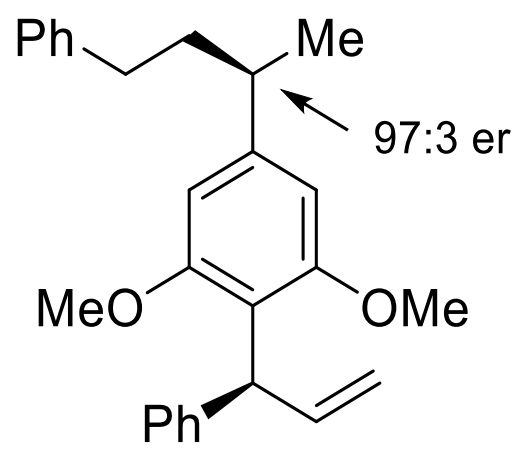

5m

Prepared following **General Procedure A** using boronic ester (*R*)-**1b** (52.0 mg, 0.200 mmol, 1.00 equiv), allyl carbonate **3a** (93.7 mg, 0.400 mmol, 2.00 equiv) and (*S*)-**L**. Purification by

flash column chromatography (hexane/toluene = 5:1) gave the title compound (61.3 mg, 79%, 97:3 dr) as a pale-yellow oil.

R*f* = 0.45 (2:1 hexane/toluene, UV, Vanillin)

¹H NMR (400 MHz, $CDCl_3$) δ 7.23 – 7.13 (m, 6H), 7.13 – 7.01 (m, 4H), 6.52 (ddd, *J* = 17.1, 9.9, 8.3 Hz, 1H), 6.32 (s, 2H), 5.26 (d, *J* = 8.3 Hz, 1H), 5.12 – 5.00 (m, 2H), 3.63 (s, 6H), 2.66 – 2.54 (m, 1H), 2.53 – 2.41 (m, 2H), 1.91 – 1.75 (m, 2H), 1.20 (d, *J* = 7.0 Hz, 3H) ppm. **¹³C NMR** (101 MHz, $CDCl_3$) δ 158.2, 147.6, 144.4, 142.7, 140.0, 128.5, 128.4, 127.8, 127.7, 125.8, 125.4, 118.0, 115.5, 103.8, 56.0, 44.5, 40.2, 40.0, 34.1, 22.5 ppm.

Specific rotation $[\alpha]_D^{24}$ = −21 (c = 2.0, CH_2Cl_2).

HRMS (ESI) m/z calculated for $C_{27}H_{31}O_2$ $[M+H]^+$, 387.2319, found: 387.2322.

The diastereomeric ratio (dr) was determined using the corresponding alcohol which was prepared following **General Procedure B**. **HPLC conditions**: Chiral column IB, hexane: isopropanol = 98:2, flow rate = 1.0 mL/min, wavelength = 230 nm, t_R = 27.1 min for major isomer, t_R = 25.3 min for minor isomer.

1,3-Dimethoxy-2-((*R*)-1-phenylallyl)-5-((*R*)-4-phenylbutan-2-yl)benzene (5n)

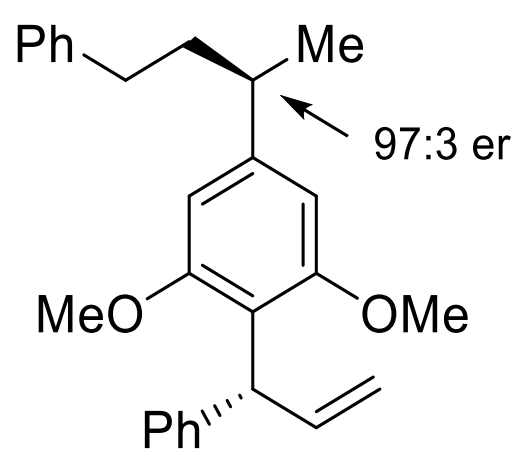

5n

Prepared following **General Procedure A** using boronic ester (*R*)-**1b** (52.0 mg, 0.200 mmol, 1.00 equiv), allyl carbonate **3a** (93.7 mg, 0.400 mmol, 2.00 equiv), allyl carbonate **3a** and (*R*)-**L**. Purification by flash column chromatography (hexane/toluene = 5:1) gave the title compound (60.6 mg, 78%, 97:3 dr) as a pale-yellow solid.

R*f* and **NMR** data are consistent with **5m**.

Specific rotation $[\alpha]_D^{24}$ = +28 (c = 2.0, CH_2Cl_2).

The diastereomeric ratio (dr) was determined using the corresponding alcohol which was prepared following **General Procedure B**. **HPLC conditions**: Chiral column IB, hexane: isopropanol = 98:2, flow rate = 1.0 mL/min, wavelength = 230 nm, t_R = 22.6 min for major isomer, t_R = 21.9 min for minor isomer.

1,3-Dimethoxy-2-((*S*)-1-phenylallyl)-5-((*S*)-4-phenylbutan-2-yl)benzene (5o)

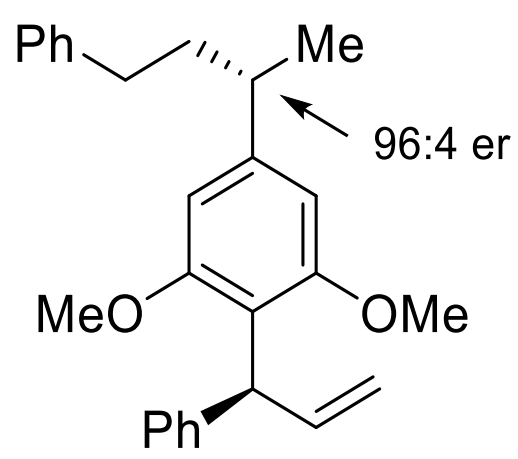

5o

Prepared following **General Procedure A** using boronic ester (*S*)-**1b** (52.0 mg, 0.200 mmol, 1.00 equiv), allyl carbonate **3a** (93.7 mg, 0.400 mmol, 2.00 equiv), allyl carbonate **3a** and (*S*)-**L**. Purification by flash column chromatography (hexane/toluene = 5:1) gave the title compound (60.6 mg, 78%, 95:5 dr) as a pale-yellow solid.

R*f* and **NMR** data are consistent with **5m**.

Specific rotation $[\alpha]_D^{24}$ = −28 (c = 2.0, CH_2Cl_2).

The diastereomeric ratio (dr) was determined using the corresponding alcohol which was prepared following **General Procedure B**. **HPLC conditions**: Chiral column IB, hexane: isopropanol = 98:2, flow rate = 1.0 mL/min, wavelength = 230 nm, t_R = 24.4 min for major isomer, t_R = 26.7 min for minor isomer.

1,3-Dimethoxy-2-((*R*)-1-phenylallyl)-5-((*S*)-4-phenylbutan-2-yl)benzene (5p)

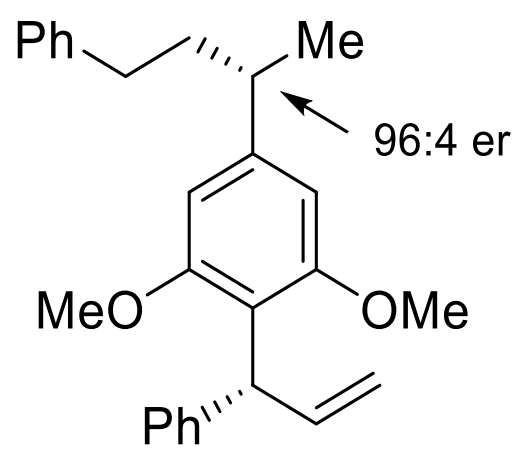

5p

Prepared following **General Procedure A** using boronic ester (*S*)-**1b** (52.0 mg, 0.200 mmol, 1.00 equiv), allyl carbonate **3a** (93.7 mg, 0.400 mmol, 2.00 equiv), allyl carbonate **3a** and (*R*)-**L**. Purification by flash column chromatography (hexane/toluene = 5:1) gave the title compound (63.7 mg, 82%, 95:5 dr) as a pale-yellow oil.

R*f* and **NMR** data are consistent with **5m**.

Specific rotation $[\alpha]_D^{24}$ = +22 (c = 3.0, CH_2Cl_2).

The diastereomeric ratio (dr) was determined using the corresponding alcohol which was prepared following **General Procedure B**. **HPLC conditions**: Chiral column IB, hexane: isopropanol = 98:2, flow rate = 1.0 mL/min, wavelength = 230 nm, t_R = 22.0 min for major isomer, t_R = 22.9 min for minor isomer.

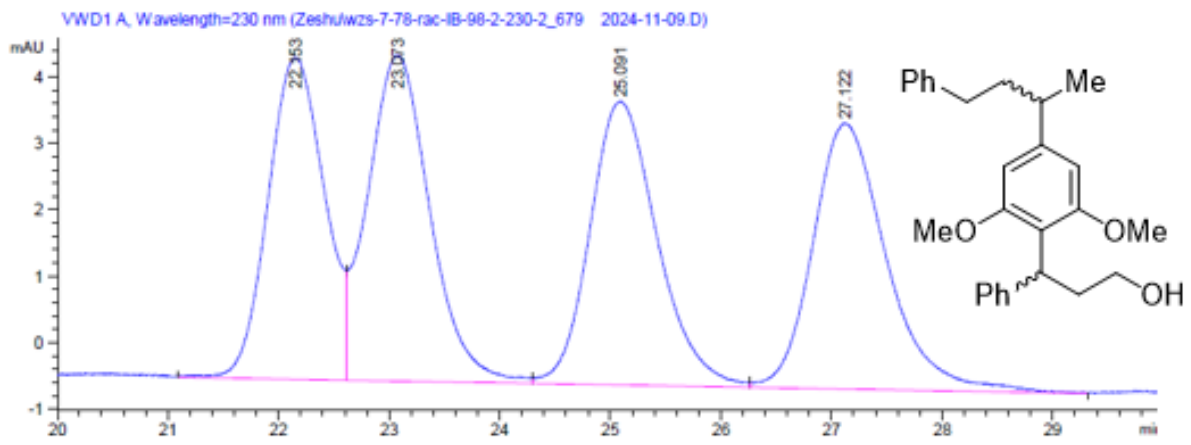

Peak #	RetTime [min]	Type	Width [min]	Area [mAU*s]	Height [mAU]	Area %
1	22.153	BV	0.5387	171.46149	4.83769	23.5406
2	23.073	VV	0.5792	191.93939	4.90114	26.3521
3	25.091	VV	0.6533	180.23964	4.26382	24.7458
4	27.122	VB	0.6906	184.72351	3.99685	25.3614

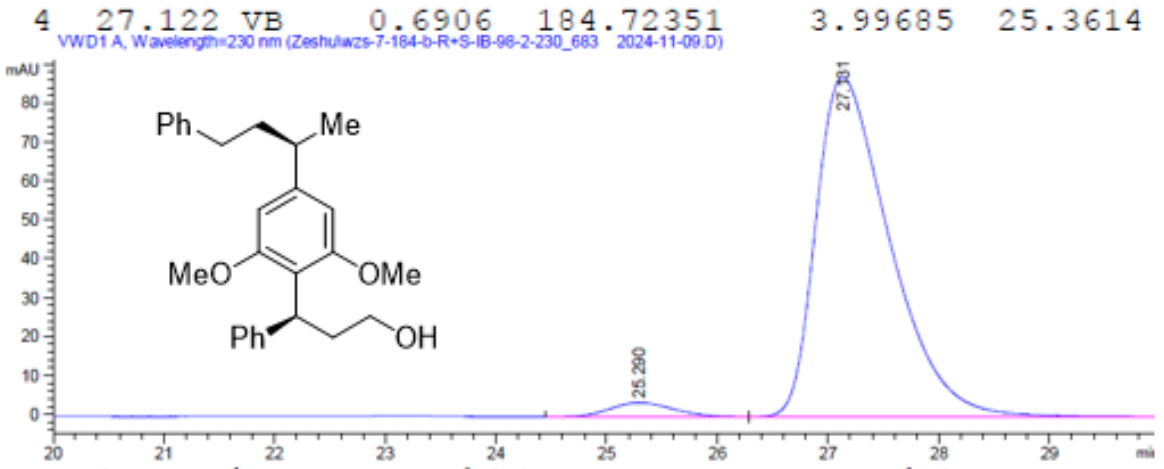

Peak #	RetTime [min]	Type	Width [min]	Area [mAU*s]	Height [mAU]	Area %
1	25.290	BV	0.6261	143.06729	3.58207	3.418
2	27.131	VB	0.7039	4041.66650	87.18158	96.581

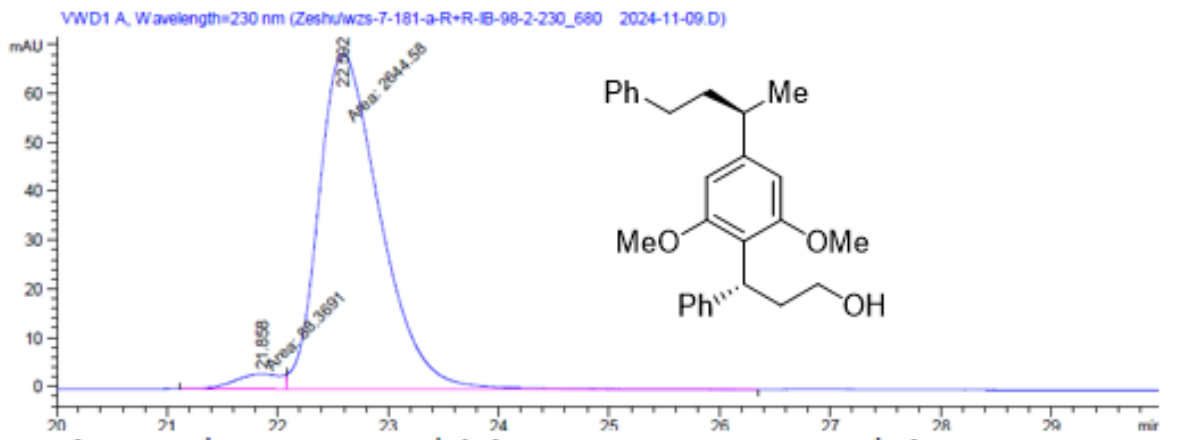

Peak #	RetTime [min]	Type	Width [min]	Area [mAU*s]	Height [mAU]	Area %
1	21.852	BV E	0.4106	76.38970	2.83355	2.7951
2	22.592	VB R	0.5947	2656.56421	68.57453	97.2049

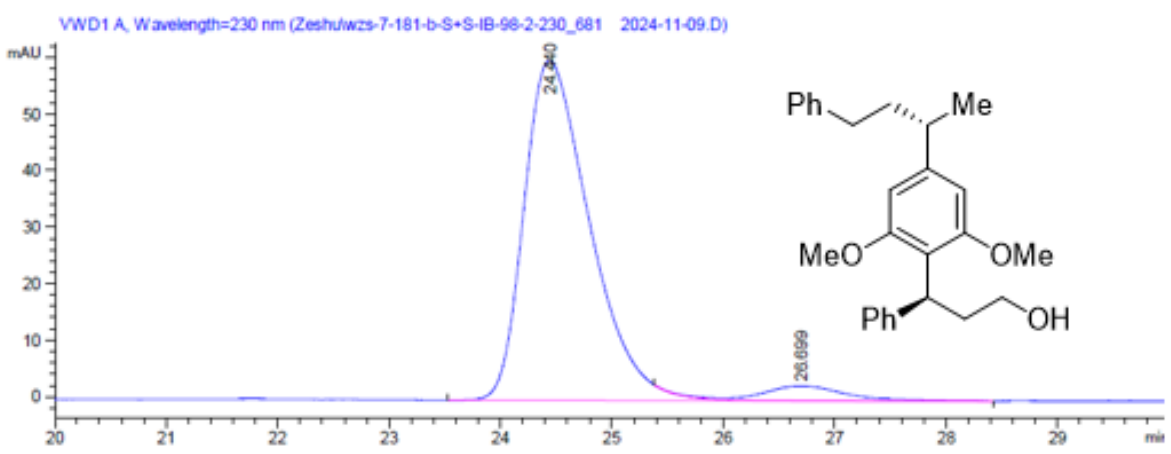

Peak #	RetTime [min]	Type	Width [min]	Area [mAU*s]	Height [mAU]	Area %
1	24.440	BV R	0.6232	2460.05664	60.09315	94.7270
2	26.699	VB E	0.7595	136.94002	2.62717	5.2730

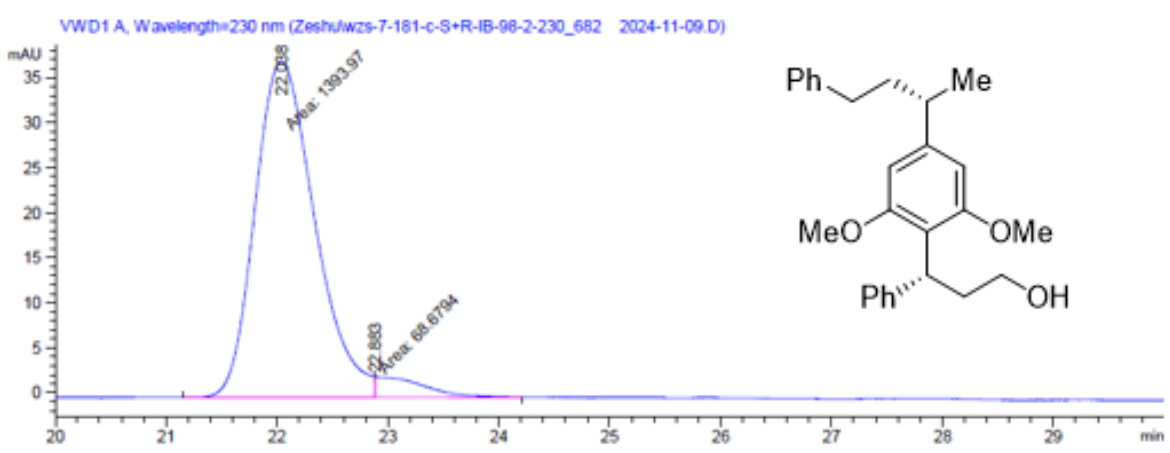

Peak #	RetTime [min]	Type	Width [min]	Area [mAU*s]	Height [mAU]	Area %
1	22.038	MF	0.6238	1393.97290	37.24395	95.3045
2	22.883	FM	0.5018	68.67937	2.28101	4.6955

***Tert*-butyl (*S*)-2-(3,5-dimethoxy-4-((*S*)-1-phenylallyl)phenyl)pyrrolidine-1-carboxylate (5q)**

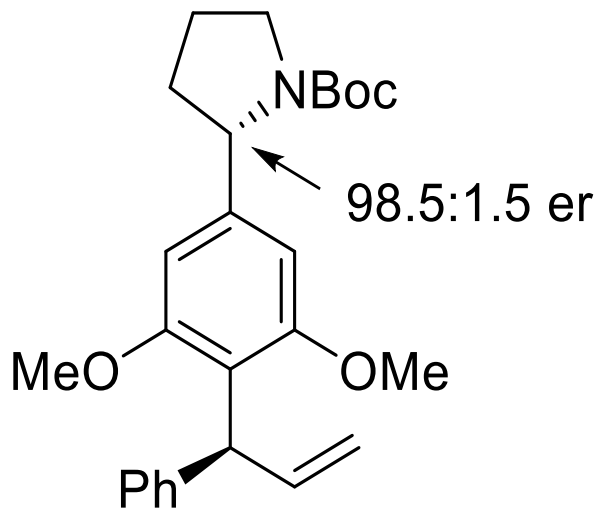

5q

Prepared following **General Procedure A** using boronic ester **1c** (59.4 mg, 0.200 mmol, 1.00 equiv) and carbonate **3a** (93.7 mg, 0.400 mmol, 2.00 equiv) except for oxidation step for 24 h. Purification by flash column chromatography (hexane/EtOAc = 10:1) gave the title compound (21.3 mg, 25%, 97:3 dr) as a pale-yellow oil.

R_f = 0.1 (25:1 hexane/EtOAc, UV, Vanillin)

^{1}H NMR (400 MHz, $CDCl_3$) δ 7.25 – 7.05 (m, 5H), 6.57 (ddd, *J* = 17.1, 10.0, 8.3 Hz, 1H), 6.36 (s, 2H), 5.33 (d, *J* = 8.3 Hz, 1H), 5.20 – 5.08 (m, 2H), 5.00 – 4.60 (m, 1H), 3.73 – 3.44 (m, 8H), 2.30 (s, 1H), 2.02 – 1.76 (m, 3H), 1.56 – 1.11 (m, 9H) ppm. (*^{1}H NMR was complicated by the presence of rotamers*) **^{13}C NMR** (101 MHz, $CDCl_3$) δ 158.2, 154.8, 144.3, 139.8, 127.7, 127.5, 125.4, 118.6, 115.6, 102.3, 79.4, 61.9, 56.0, 47.3, 44.4, 28.6, 28.4, 23.6 ppm.

Specific rotation $[\alpha]_D^{24}$ = −66 (c = 1.3, CH_2Cl_2).

HRMS (ESI) m/z calculated for $C_{26}H_{33}NNaO_4$ $[M+Na]^+$, 446.2302, found: 446.2289.

HPLC conditions: Chiral column ADH, hexane: isopropanol = 95:5, flow rate = 1.0 mL/min, wavelength = 230 nm, t_R = 7.4 min for major isomer, t_R = 4.8 min for minor isomer.

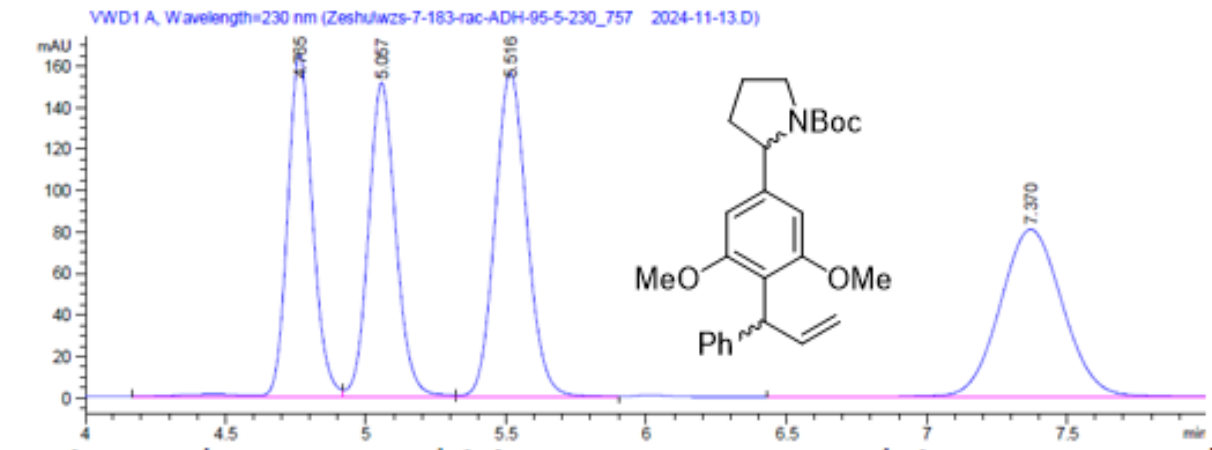

Peak #	RetTime [min]	Type	Width [min]	Area [mAU*s]	Height [mAU]	Area %
1	4.765	VV R	0.0950	1027.41003	164.82837	22.5678
2	5.057	VV	0.1085	1024.74219	150.67281	22.5092
3	5.516	VB	0.1242	1231.24255	155.90085	27.0451
4	7.370	BV R	0.2444	1269.15002	80.79770	27.8778

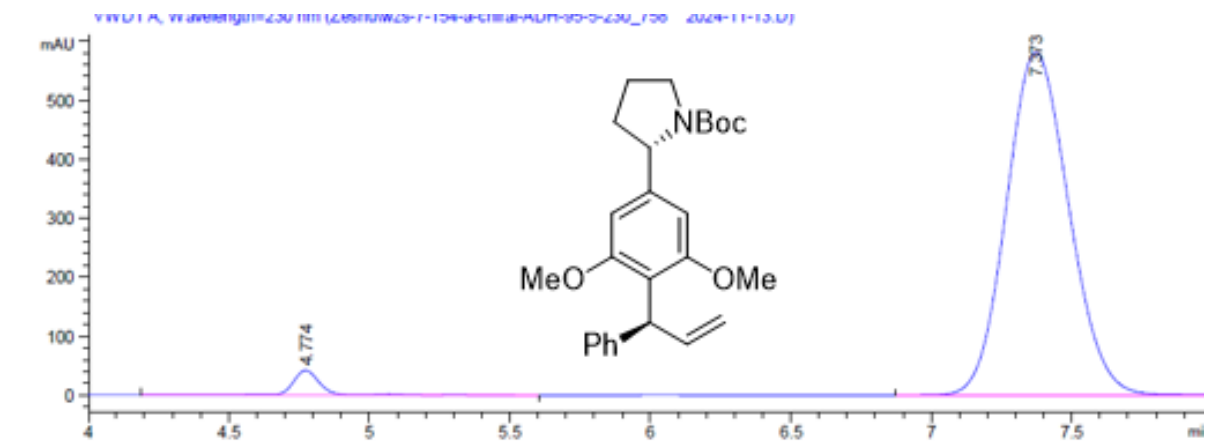

Peak #	RetTime [min]	Type	Width [min]	Area [mAU*s]	Height [mAU]	Area %
1	4.774	VV R	0.0995	294.23676	41.82477	3.1637
2	7.373	BV R	0.2416	9006.09180	580.91791	96.8363

5-((1*R*,2*S*,5*R*)-2-isopropyl-5-methylcyclohexyl)-1,3-dimethoxy-2-((*S*)-1-phenylallyl)benzene (5r)

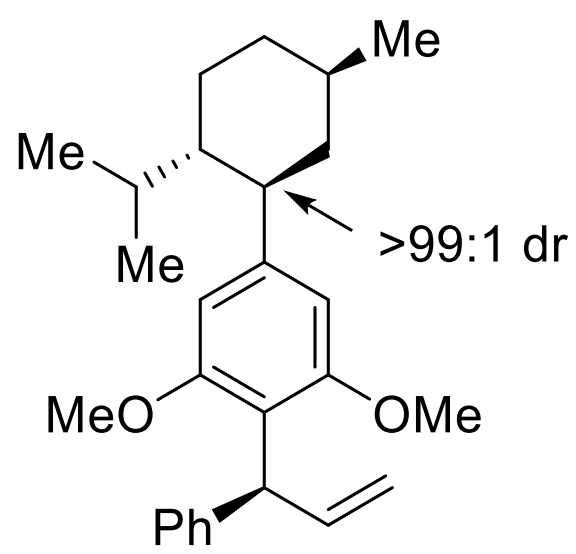

5r

Prepared following **General Procedure A** using boronic ester **1d** (53.2 mg, 0.200 mmol, 1.00 equiv) and carbonate **3a** (93.7 mg, 0.400 mmol, 2.00 equiv). Purification by flash column chromatography (hexane/toluene = 5:1) gave the title compound (29.8 mg, 38%, >99:1 dr) as a white solid.

R_f = 0.2 (4:1 hexane/toluene, UV, Vanillin)

^{1}H NMR (500 MHz, $CDCl_3$) δ 7.24 – 7.18 (m, 4H), 7.18 – 7.09 (m, 1H), 6.65 – 6.51 (m, 1H), 6.35 (s, 2H), 5.32 (d, *J* = 8.4 Hz, 1H), 5.19 – 5.08 (m, 2H), 3.70 (s, 6H), 2.42 – 2.33 (m, 1H), 1.85 – 1.77 (m, 2H), 1.77 – 1.70 (m, 1H), 1.54 – 1.35 (m, 3H), 1.19 – 1.08 (m, 2H), 1.06 – 0.96 (m, 1H), 0.91 (d, *J* = 6.5 Hz, 3H), 0.81 (d, *J* = 7.6 Hz, 3H), 0.70 (d, *J* = 7.6 Hz, 3H) ppm. **^{13}C NMR** (126 MHz, $CDCl_3$) δ 158.1, 146.9, 144.5, 140.2, 127.8, 127.7, 125.3, 117.6, 115.4, 56.0, 48.7, 47.6, 45.4, 44.6, 35.4, 33.4, 27.5, 24.8, 22.7, 21.8, 15.7 ppm.

Specific rotation $[\alpha]_D^{24}$ = −61 (c = 2.0, CH_2Cl_2).

HRMS (ESI) m/z calculated for $C_{27}H_{37}O_2$ $[M+H]^+$, 393.2788, found: 393.2775.

HPLC conditions: Chiral column IC, hexane: isopropanol = 99:1, flow rate = 1.0 mL/min, wavelength = 230 nm, t_R = 3.5 min for major isomer, t_R = 3.8 min for minor isomer.

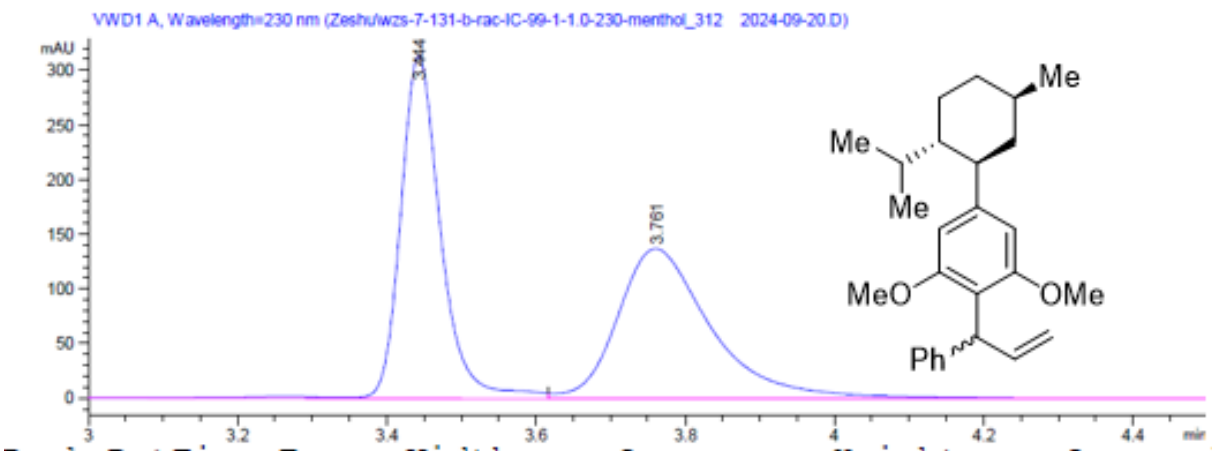

Peak #	RetTime [min]	Type	Width [min]	Area [mAU*s]	Height [mAU]	Area %
1	3.444	VV R	0.0612	1191.54102	311.74033	48.9473
2	3.761	VB	0.1377	1242.79419	137.21094	51.0527

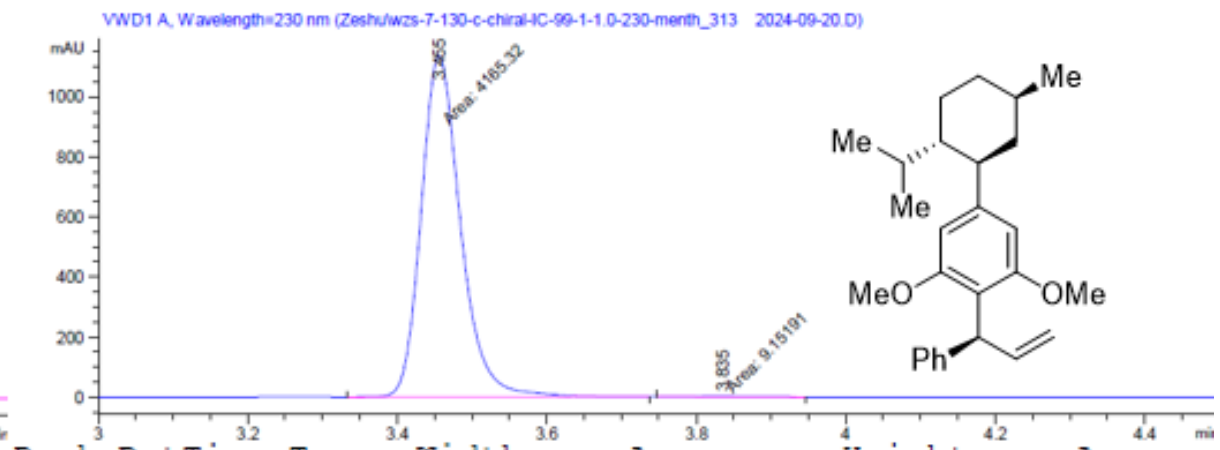

Peak #	RetTime [min]	Type	Width [min]	Area [mAU*s]	Height [mAU]	Area %
1	3.455	MM	0.0609	4165.31738	1139.79919	99.7808
2	3.835	MM	0.0906	9.15191	1.68278	0.2192

***Tert*-butyl(((3*S*,5*R*,6*S*,8*S*,9*S*,10*R*,13*R*,14*S*,17*R*)-6-(3,5-dimethoxy-4-((*S*)-1-phenylallyl)phenyl)-10,13-dimethyl-17-((*R*)-6-methylheptan-2-yl)hexadecahydro-1*H*-cyclopenta[*a*]phenanthren-3-yl)oxy)dimethylsilane (5s)**

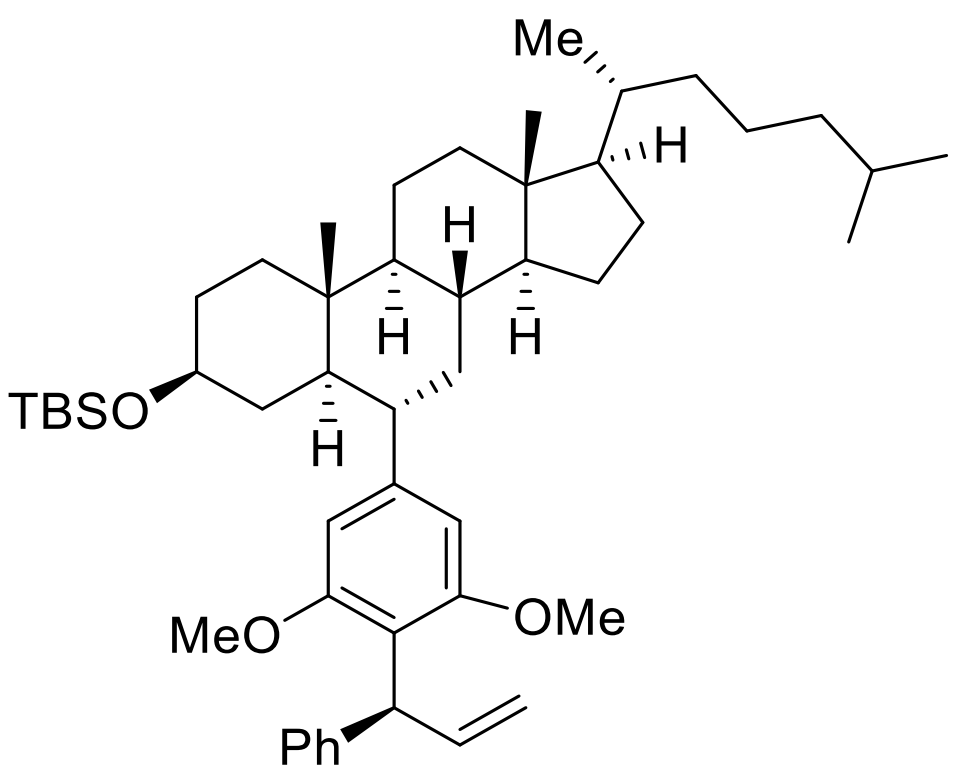

5s

Prepared following **General Procedure A** using boronic ester **1e** (126 mg, 0.200 mmol, 1.00 equiv) and carbonate **3a** (93.7 mg, 0.400 mmol, 2.00 equiv) except for oxidation step for 24 h. Purification by flash column chromatography (hexane/toluene = 5:1) gave the title compound (102 mg, 68%, >99:1 dr) as a white solid.

R_f = 0.63 (1:1 hexane/toluene, UV, Vanillin)

^{1}H NMR (500 MHz, $CDCl_3$) δ 7.23 – 7.15 (m, 4H), 7.15 – 7.07 (m, 1H), 6.55 (ddd, J = 16.9, 10.0, 8.2 Hz, 1H), 6.31 (s, 2H), 5.32 (d, J = 8.3 Hz, 1H), 5.17 – 5.07 (m, 2H), 3.67 (s, 6H), 3.44 – 3.35 (m, 1H), 2.42 – 2.33 (m, 1H), 2.05 – 1.97 (m, 1H), 1.88 – 1.71 (m, 3H), 1.71 – 1.63 (m, 1H), 1.61 – 1.42 (m, 5H), 1.42 – 0.95 (m, 19H), 0.95 – 0.90 (m, 6H), 0.87 (d, J = 2.1 Hz, 3H), 0.86 (d, J = 2.1 Hz, 3H), 0.78 (s, 9H), 0.68 (s, 3H), –0.09 (d, J = 2.0 Hz, 6H) ppm. **^{13}C NMR**

(126 MHz, $CDCl_3$) δ 146.4, 144.5, 139.9, 127.8, 127.5, 125.3, 118.0, 115.5, 72.9, 56.5, 56.4, 56.0, 54.6, 50.2, 45.4, 44.3, 42.8, 41.1, 40.3, 39.7, 37.7, 36.3, 36.2, 35.9, 35.5, 35.1, 31.9, 28.4, 28.2, 26.1, 24.4, 24.0, 23.0, 22.7, 21.5, 18.8, 18.5, 13.5, 12.2, 0.2, –4.6, –4.6 ppm.

Specific rotation $[\alpha]_D^{24} = -7$ (c = 3.0, CH_2Cl_2).

HRMS (ESI) m/z calculated for $C_{50}H_{79}O_3Si$ $[M+H]^+$, 755.5793, found: 755.5786.

The diastereomeric ratio (dr) was determined using the corresponding alcohol which was prepared following **General Procedure B**. **HPLC conditions**: Chiral column IA, hexane: isopropanol = 98:2, flow rate = 1.0 mL/min, wavelength = 230 nm, t_R = 9.2 min for major isomer.

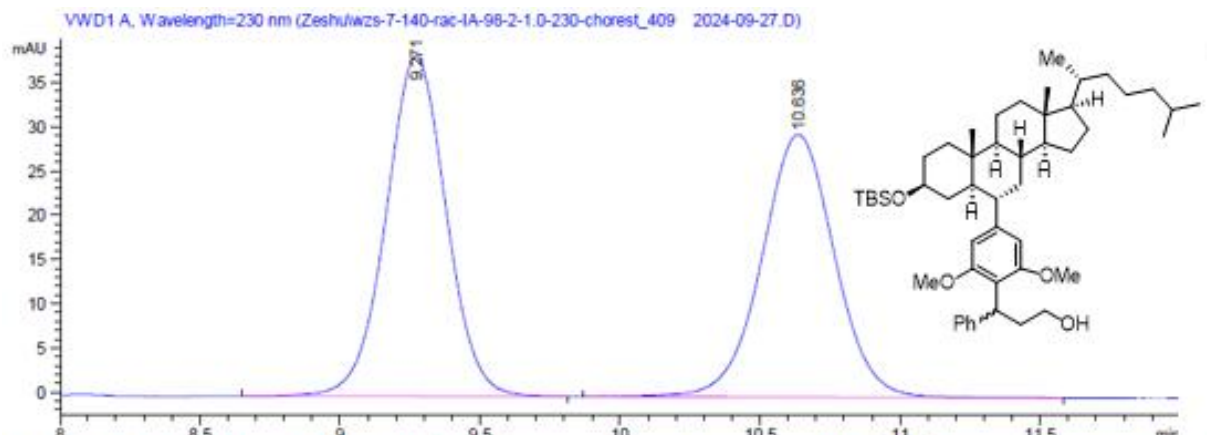

Peak #	RetTime [min]	Type	Width [min]	Area [mAU*s]	Height [mAU]	Area %
1	9.271	BB	0.2332	575.19080	38.36473	51.7723
2	10.636	BB	0.2807	535.81116	29.64709	48.2277

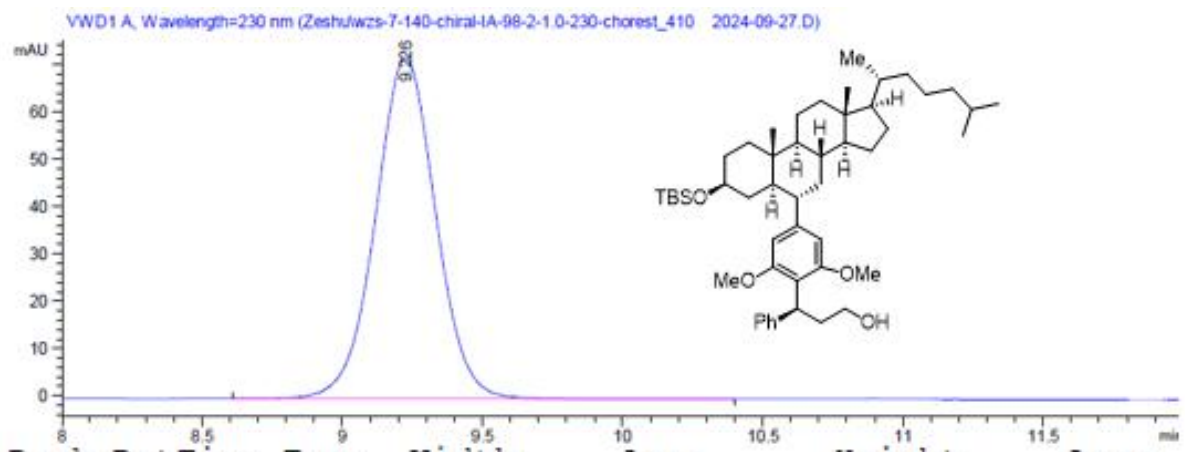

Peak #	RetTime [min]	Type	Width [min]	Area [mAU*s]	Height [mAU]	Area %
1	9.226	BB	0.2299	1077.39758	71.98642	100.0000

1,3-Dimethoxy-2-((*S*)-1-phenylallyl)-5-((*R*)-1-phenylethyl)benzene (5t)

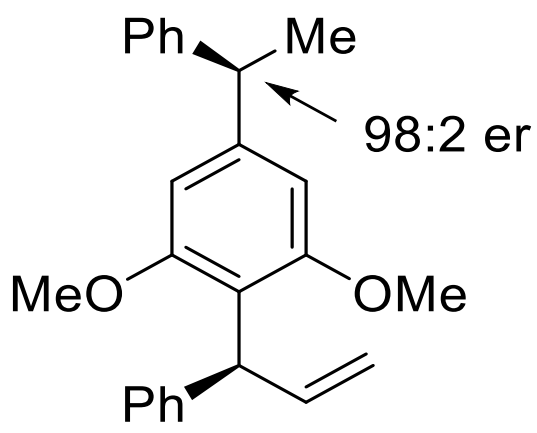

5t

Prepared following **General Procedure A** using boronic ester **1f** (46.4 mg, 0.200 mmol, 1.00 equiv) and carbonate **3a** (93.7 mg, 0.400 mmol, 2.00 equiv). Purification by flash column chromatography (hexane/toluene = 5:1) gave the title compound (36.6 mg, 51%, 98:2 dr) as a colourless oil.

R_f = 0.2 (4:1 hexane/toluene, UV, Vanillin)

^{1}H NMR (500 MHz, $CDCl_3$) δ 7.24 – 7.19 (m, 2H), 7.19 – 7.09 (m, 7H), 7.06 – 7.01 (m, 1H), 6.50 (ddd, *J* = 17.1, 9.9, 8.4 Hz, 1H), 6.34 (s, 2H), 5.24 (d, *J* = 8.4 Hz, 1H), 5.10 – 5.00 (m, 2H), 4.02 (q, *J* = 7.2 Hz, 1H), 3.58 (s, 6H), 1.56 (d, *J* = 7.2 Hz, 3H) ppm. **^{13}C NMR** (126 MHz, $CDCl_3$) δ 158.1, 146.4, 144.3, 139.9, 128.5, 127.8, 127.7, 127.7, 126.2, 125.4, 118.1, 115.5, 104.6, 55.9, 45.3, 44.5, 22.1 ppm.

Specific rotation $[\alpha]_D^{24} = -27$ (c = 1.1, CH_2Cl_2).

HRMS (ESI) m/z calculated for $C_{25}H_{27}O_2$ $[M+H]^+$, 359.2006, found: 359.1995.

The diastereomeric ratio (dr) was determined using the corresponding alcohol which was prepared following **General Procedure B** (Note). **HPLC conditions**: Chiral column IC, hexane: isopropanol = 95:5, flow rate = 1.0 mL/min, wavelength = 230 nm, t_R = 27.3 min for major isomer, t_R = 29.0 min for minor isomer.

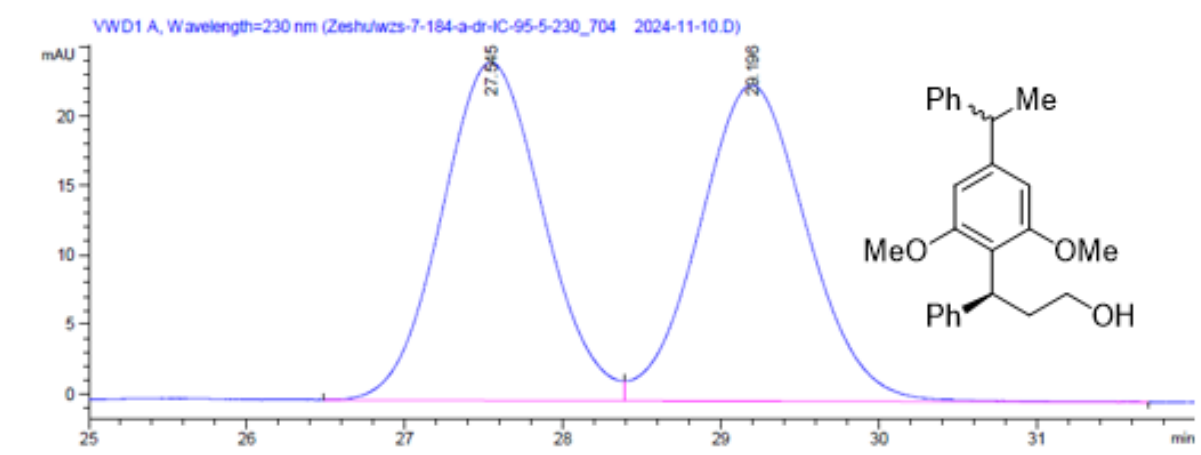

Peak #	RetTime [min]	Type	Width [min]	Area [mAU*s]	Height [mAU]	Area %
1	27.545	BV	0.6981	1086.04053	24.34419	50.1366
2	29.196	VB	0.7286	1080.12451	22.75104	49.8634

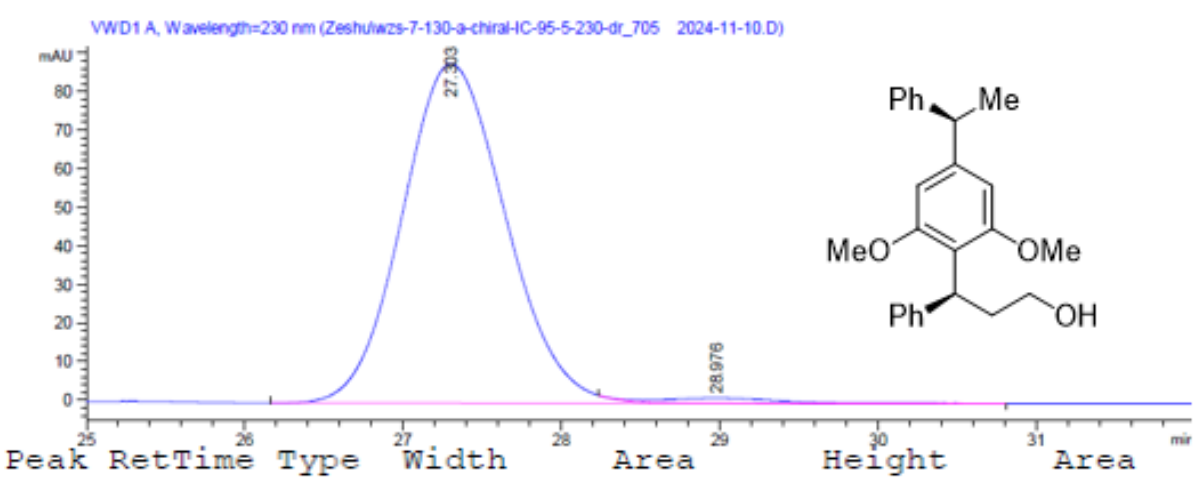

Peak #	RetTime [min]	Type	Width [min]	Area [mAU*s]	Height [mAU]	Area %
1	27.303	VV R	0.7133	3993.49414	87.93979	98.3338
2	28.976	VB E	0.7127	67.66550	1.33721	1.6662

Note: The fully racemic product, which consists of four diastereoisomers, could not be resolved by chiral HPLC. Therefore, we prepared the racemic standard from the racemic boronic ester and (*S*)-**L** to simplify the spectral analysis.

((2*R*,3*R*)-pent-4-ene-2,3-diyl)dibenzene (5t')

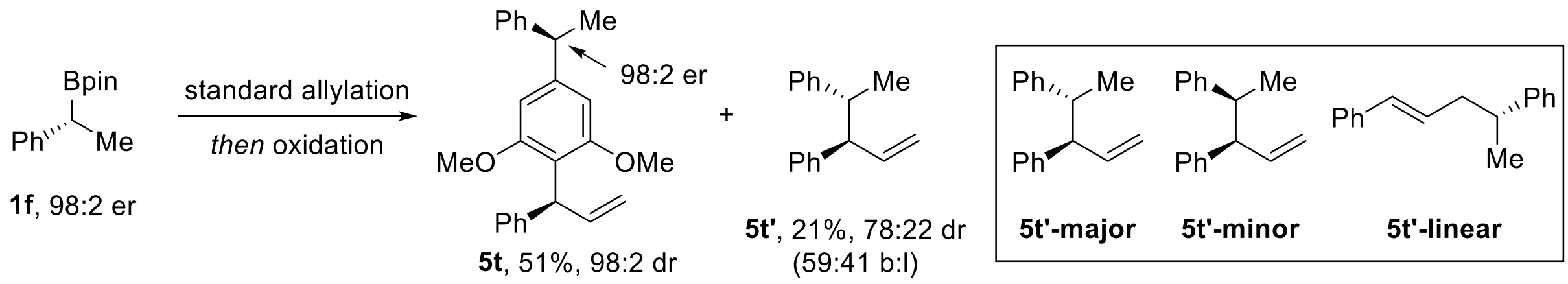

Compound **5t'** as the major side product was prepared following **General Procedure A** using boronic ester **1f** (46.4 mg, 0.200 mmol, 1.00 equiv) and carbonate **3a** (93.7 mg, 0.400 mmol, 2.00 equiv). Purification by flash column chromatography (hexane/toluene = 20:1) gave the title compound (9.3 mg, 21%, 59:41 b:l, 78:22 dr) as a pale-yellow oil.

All recorded spectroscopic data of compound **5t'** matched that previously reported.[5]

R*f* = 0.56 (4:1 hexane/toluene, UV)

¹H NMR (500 MHz, $CDCl_3$) δ 7.29 – 7.02 (m, 7.3 H, $H_{5t'-major+5t'-minor+5t'-linear}$), 7.00 – 6.88 (m, 2.7H, $H_{5t'-major+5t'-minor}$), 6.35 – 6.26 (m, 0.41H, $H_{5t'-linear}$), 6.10 – 5.96 (m, 0.87H, $H_{5t'-major+5t'-linear}$), 5.77 (ddd, *J* = 17.0, 10.3, 8.0 Hz, 0.13H, $H_{5t'-minor}$), 5.06 – 4.96 (m, 0.92H, $H_{5t'-major}$), 4.75 (ddd, *J* = 10.3, 1.7, 0.9 Hz, 0.13H, $H_{5t'-minor}$), 4.66 (dt, *J* = 17.1, 1.4 Hz, 0.13H, $H_{5t'-minor}$), 3.37 – 3.30 (m, 0.59H, $H_{5t'-major+5t'-minor}$), 3.04 – 2.93 (m, 0.59H, $H_{5t'-major+5t'-minor}$), 2.87 – 2.75 (m, 0.41H, $H_{5t'-linear}$), 2.51 – 2.42 (m, 0.41H, $H_{5t'-linear}$), 2.41 – 2.30 (m, 0.41H, $H_{5t'-linear}$), 1.26 (d, *J* = 7.0 Hz, 1.38H, $H_{5t'-major}$), 1.23 (d, *J* = 7.0 Hz, 1.23H, $H_{5t'-linear}$), 1.01 (d, *J* = 6.9 Hz, 0.13H, $H_{5t'-minor}$) ppm. **¹³C NMR** (126 MHz, $CDCl_3$) δ 147.1, 145.7, 145.5, 143.6, 143.5, 140.8, 140.7, 137.9, 131.4, 129.2, 128.6, 128.6, 128.5, 128.4, 128.3, 128.2, 128.1, 128.1, 128.0, 127.9, 127.1, 127.0, 126.5, 126.3, 126.2, 126.1, 126.0, 125.9, 115.8, 115.5, 58.4, 57.6, 45.6, 45.1, 42.1, 40.3, 21.6, 20.9, 20.4 ppm.

(*S*)-1,3-dimethoxy-5-phenethyl-2-(1-phenylallyl)benzene (5u)

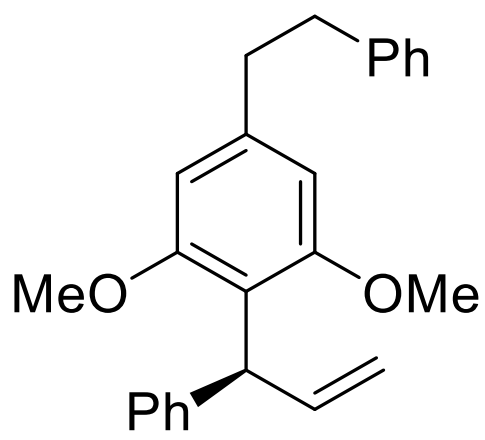

5u

Prepared following **General Procedure A** using 4,4,5,5-tetramethyl-2-phenethyl-1,3,2-dioxaborolane (46.4 mg, 0.200 mmol, 1.00 equiv) and allyl carbonate **3a** (93.7 mg, 0.400 mmol, 2.00 equiv) in which a solvent switch from THF to DCM was performed before **Step 2** of the reaction. Purification by flash column chromatography (hexane/toluene = 5:1) gave the title compound (33.0 mg, 46%, >99% ee) as a white solid.

R*f* = 0.43 (2:1 hexane/toluene, UV, Vanillin)

¹H NMR (400 MHz, $CDCl_3$) δ 7.36 – 7.29 (m, 2H), 7.29 – 7.20 (m, 7H), 7.19 – 7.11 (m, 1H), 6.61 (ddd, *J* = 17.1, 10.0, 8.3 Hz, 1H), 6.40 (s, 2H), 5.37 (d, *J* = 8.3 Hz, 1H), 5.26 – 5.11 (m, 2H), 3.69 (s, 6H), 3.01 – 2.86 (m, 4H) ppm. **¹³C NMR** (101 MHz, $CDCl_3$) δ 158.2, 144.4,

141.9, 139.9, 128.6, 128.5, 127.8, 127.6, 126.1, 125.4, 118.1, 115.6, 105.3, 56.0, 44.4, 38.6, 38.0 ppm.

Specific rotation $[\alpha]_D^{24} = -32$ (c = 2.0, CH_2Cl_2).

HRMS (ESI) m/z calculated for $C_{25}H_{27}O_2$ $[M+H]^+$, 359.2006, found: 359.2012.

The enantiomeric excess (ee) was determined using the corresponding alcohol which was prepared following **General Procedure B**. **HPLC conditions**: Chiral column IC, hexane: isopropanol = 90:10, flow rate = 1.0 mL/min, wavelength = 230 nm, t_R = 15.7 min for major isomer, t_R = 13.0 min for minor isomer.

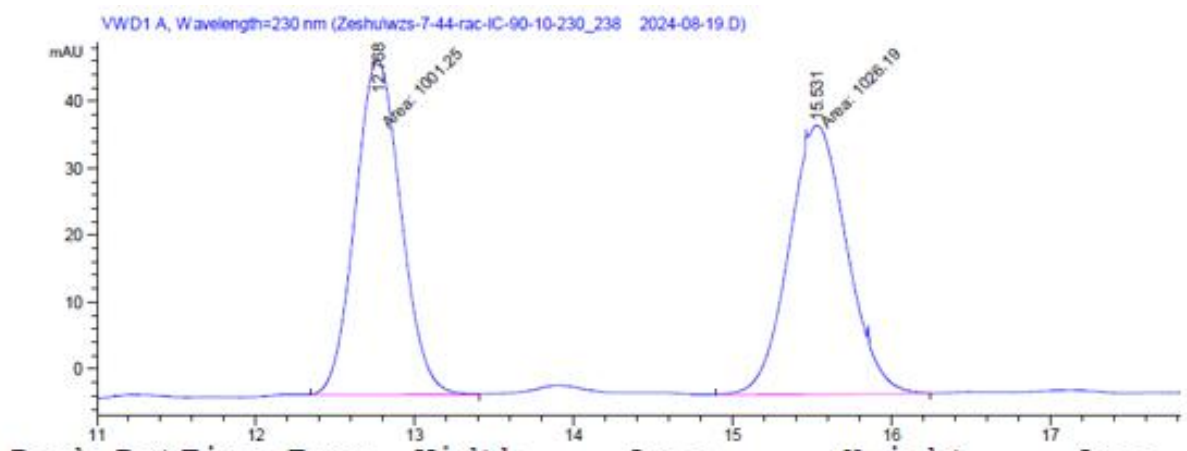

Peak #	RetTime [min]	Type	Width [min]	Area [mAU*s]	Height [mAU]	Area %
1	12.768	MM	0.3330	1001.25146	50.10662	49.3850
2	15.531	MM	0.4249	1026.18774	40.24804	50.6150

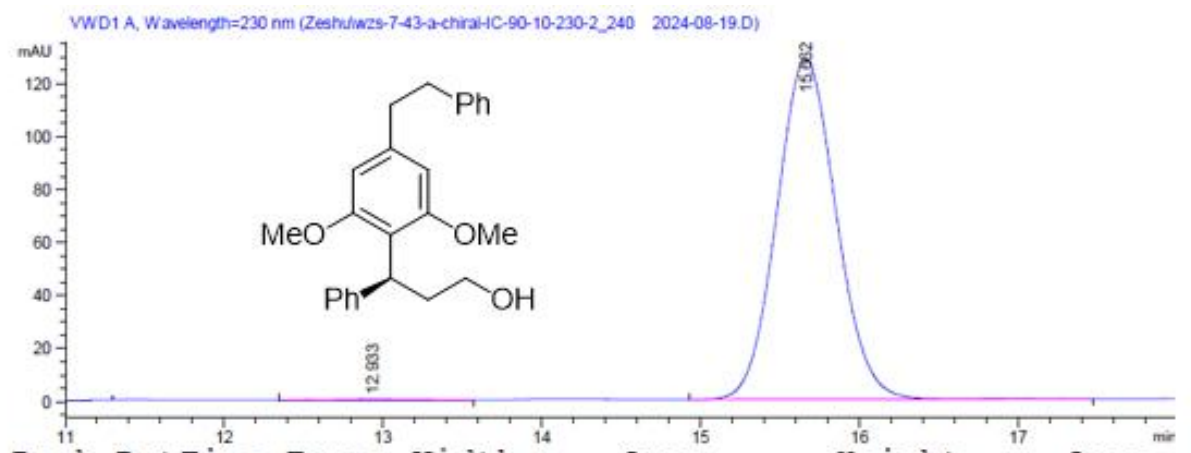

Peak #	RetTime [min]	Type	Width [min]	Area [mAU*s]	Height [mAU]	Area %
1	12.933	BB	0.3852	13.18618	4.32081e-1	0.3909
2	15.662	BV R	0.4059	3359.78369	128.56703	99.6091

(*S*)-5-(*tert*-butyl)-1,3-dimethoxy-2-(1-phenylallyl)benzene (5v)

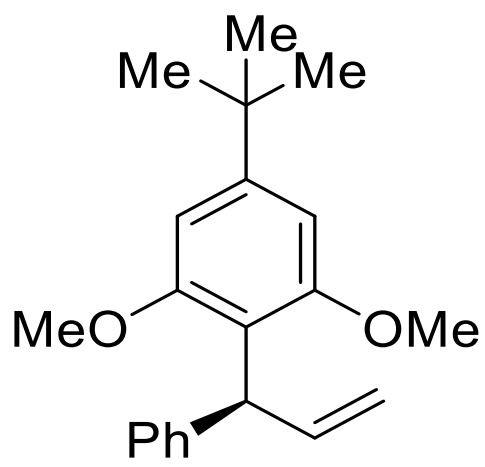

5v

Prepared following **General Procedure A** using 2-(*tert*-butyl)-4,4,5,5-tetramethyl-1,3,2-dioxaborolane (36.8 mg, 0.200 mmol, 1.00 equiv) and allyl carbonate **3a** (93.7 mg, 0.400 mmol, 2.00 equiv). Purification by flash column chromatography (hexane/toluene = 5:1) gave the title compound (45.8 mg, 74%, >99% ee) as a white solid.

$\mathbf{R_f}$ = 0.59 (2:1 hexane/toluene, UV, Vanillin)

^{1}H NMR (500 MHz, $CDCl_3$) δ 7.26 – 7.18 (m, 4H), 7.17 – 7.09 (m, 1H), 6.67 – 6.55 (m, 3H), 5.34 (d, J = 8.4 Hz, 1H), 5.19 – 5.08 (m, 2H), 3.72 (s, 6H), 1.32 (s, 9H) ppm. **^{13}C NMR** (126

MHz, $CDCl_3$) δ 157.8, 151.4, 144.5, 140.0, 127.8, 127.7, 125.4, 117.7, 115.5, 102.6, 56.0, 44.5, 35.2, 31.5 ppm.

Specific rotation $[\alpha]_D^{24}$ = −44 (c = 2.0, CH_2Cl_2).

HRMS (ESI) m/z calculated for $C_{21}H_{27}O_2$ $[M+H]^+$, 311.2006, found: 311.2002.

The enantiomeric excess (ee) was determined using the corresponding alcohol which was prepared following **General Procedure B**. **HPLC conditions**: Chiral column IC, hexane: isopropanol = 95:5, flow rate = 1.0 mL/min, wavelength = 230 nm, t_R = 21.3 min for major isomer.

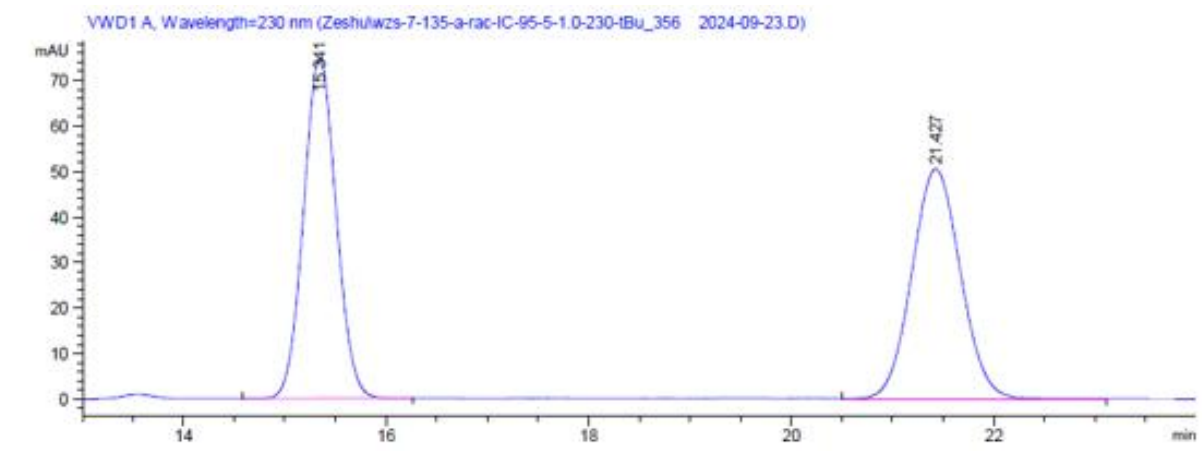

Peak #	RetTime [min]	Type	Width [min]	Area [mAU*s]	Height [mAU]	Area %
1	15.341	BB	0.3488	1671.84314	74.60683	49.9459
2	21.427	BB	0.5163	1675.46655	50.35518	50.0541

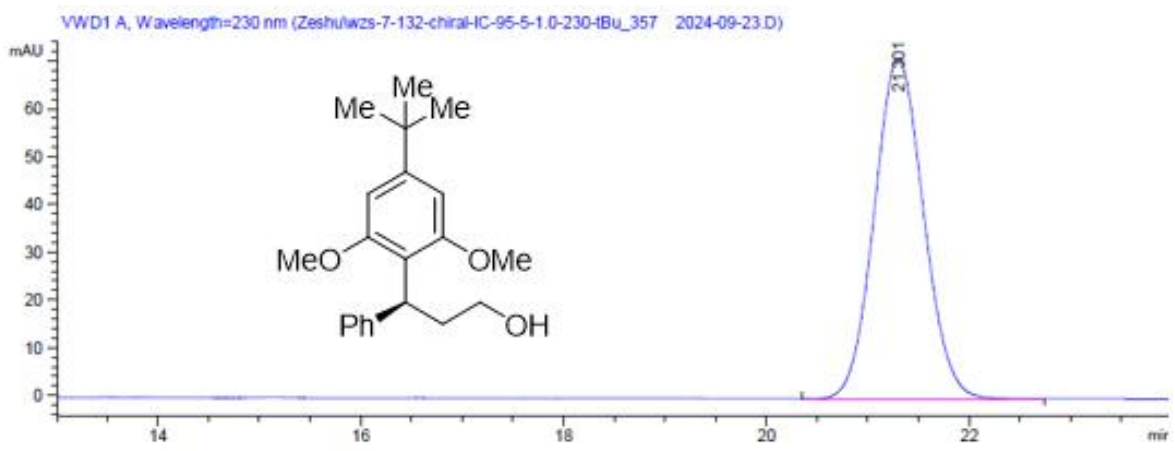

Peak #	RetTime [min]	Type	Width [min]	Area [mAU*s]	Height [mAU]	Area %
1	21.301	BB	0.5250	2405.00537	71.21996	100.0000

1,3-Dimethoxy-2-((*S*)-1-phenylallyl)-5-((*R*)-2-phenylhex-5-en-2-yl)benzene (5w)

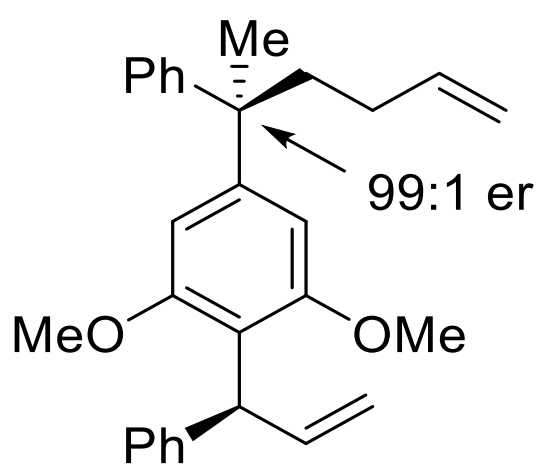

5w

Prepared following a modified **General Procedure A** using boronic ester **1g** (57.2 mg, 0.200 mmol, 1.00 equiv) and carbonate **3a** (93.7 mg, 0.400 mmol, 2.00 equiv) in which the oxidation step was performed for 24 h. Purification by flash column chromatography (hexane/toluene = 5:1) gave the title compound (26.0 mg, 32%, 94:6 dr) as a pale-yellow oil.

$\mathbf{R_f}$ = 0.73 (2:1 hexane/toluene, UV, Vanillin)

^{1}H NMR (500 MHz, $CDCl_3$) δ 7.34 – 7.07 (m, 10H), 6.64 – 6.53 (m, 1H), 6.38 (s, 2H), 5.87 – 5.75 (m, 1H), 5.31 (d, *J* = 8.4 Hz, 1H), 5.18 – 5.07 (m, 2H), 5.04 – 4.90 (m, 2H), 3.61 (s, 6H),

2.23 – 2.09 (m, 2H), 1.94 – 1.81 (m, 2H), 1.64 (s, 3H) ppm. **^{13}C NMR** (126 MHz, $CDCl_3$) δ 157.8, 149.6, 149.3, 144.4, 140.0, 139.3, 128.1, 127.8, 127.7, 127.5, 125.8, 125.4, 117.9, 115.5, 114.3, 104.7, 56.0, 46.7, 44.5, 41.2, 29.5, 27.8 ppm.

Specific rotation $[\alpha]_D^{24} = -55$ (c = 1.2, CH_2Cl_2).

HRMS (ESI) m/z calculated for $C_{29}H_{33}O_2$ $[M+H]^+$, 413.2475, found: 413.2481.

The diastereomeric ratio (dr) was determined using the corresponding alcohol which was prepared following **General Procedure B**. **HPLC conditions**: Chiral column IA, hexane: isopropanol = 90:10, flow rate = 1.0 mL/min, wavelength = 230 nm, t_R = 23.8 min for major isomer, t_R = 15.1 min for minor isomer.

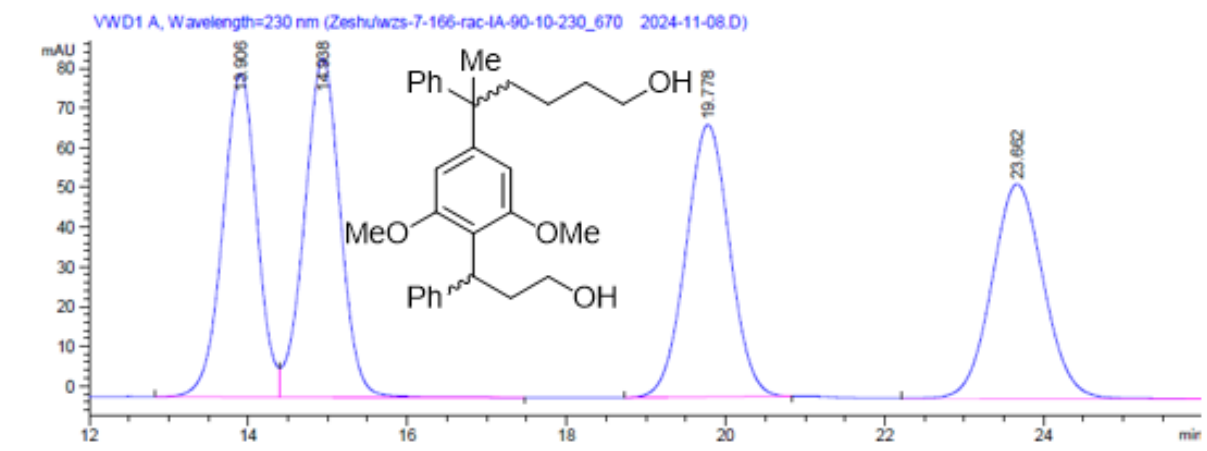

Peak #	RetTime [min]	Type	Width [min]	Area [mAU*s]	Height [mAU]	Area %
1	13.906	BV	0.4613	2428.19043	81.46760	23.9180
2	14.938	VB	0.4838	2667.07495	85.37653	26.2710
3	19.778	BB	0.5972	2620.40137	68.58041	25.8113
4	23.662	BB	0.7011	2436.49683	54.00206	23.9998

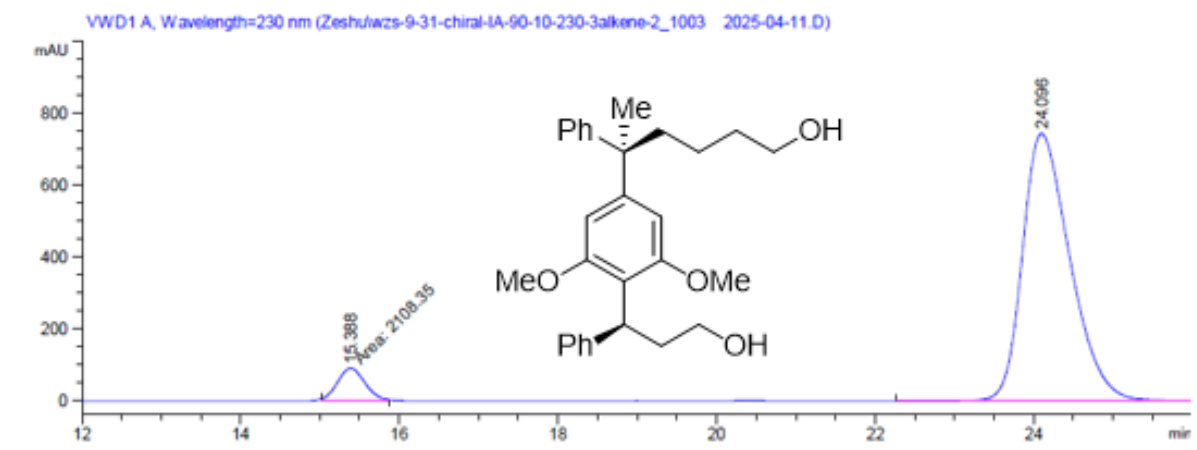

Peak #	RetTime [min]	Type	Width [min]	Area [mAU*s]	Height [mAU]	Area %
1	15.388	MM	0.3954	2108.35156	88.87177	6.3528
2	24.096	BB	0.6387	3.10795e4	744.05566	93.6472

(*S*)-3,5-dimethoxy-4-(1-phenylallyl)-1,1'-biphenyl (5y)

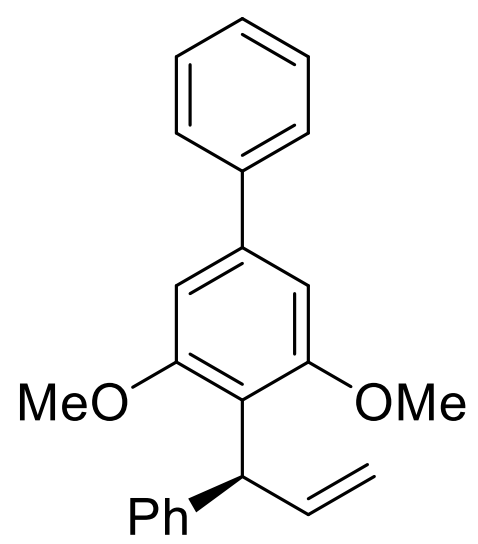

5y

Prepared following **General Procedure A** using 4,4,5,5-tetramethyl-2-phenyl-1,3,2-dioxaborolane (40.8 mg, 0.200 mmol, 1.00 equiv) and allyl carbonate **3a** (93.7 mg, 0.400 mmol, 2.00 equiv) in which a solvent switch from THF to DCM was performed before **Step 2**

of the reaction. Purification by flash column chromatography (hexane/toluene = 5:1) gave the title compound (19.2 mg, 29%, >99% ee) as a white solid (Note).

Note: Analysis of the reaction mixture revealed that the phenyl-dimethoxybenzene coupling product (3,5-dimethoxy-1,1'-biphenyl) was generated in approximately 20-30% yield. Presumably, this arises due to the low reactivity of the boronate complex, which, if unable to react with the π-allyl-iridium complex, will undergo the observed coupling upon oxidation.

R_f = 0.43 (2:1 hexane/toluene, UV, Vanillin)

^{1}H NMR (400 MHz, $CDCl_3$) δ 7.62 – 7.55 (m, 2H), 7.49 – 7.39 (m, 2H), 7.40 – 7.31 (m, 1H), 7.29 – 7.21 (m, 4H), 7.19 – 7.10 (m, 1H), 6.77 (s, 2H), 6.63 (ddd, *J* = 17.2, 10.0, 8.3 Hz, 1H), 5.41 (d, *J* = 8.3 Hz, 1H), 5.25 – 5.12 (m, 2H), 3.78 (s, 6H) ppm. **^{13}C NMR** (101 MHz, $CDCl_3$) δ 158.5, 144.1, 141.7, 141.4, 139.6, 128.9, 127.9, 127.6, 127.5, 127.3, 125.5, 119.6, 115.8, 104.2, 56.1, 44.4 ppm.

Specific rotation $[\alpha]_D^{24}$ = −94 (c = 0.7, CH_2Cl_2). **HRMS** (ESI) m/z calculated for $C_{23}H_{23}O_2$ $[M+H]^+$, 331.1693, found: 331.1681.

The enantiomeric excess (ee) was determined using the corresponding alcohol which was prepared following **General Procedure B**. **HPLC conditions**: Chiral column IB, hexane: isopropanol = 95:5, flow rate = 1.0 mL/min, wavelength = 230 nm, t_R = 15.7 min for major isomer.

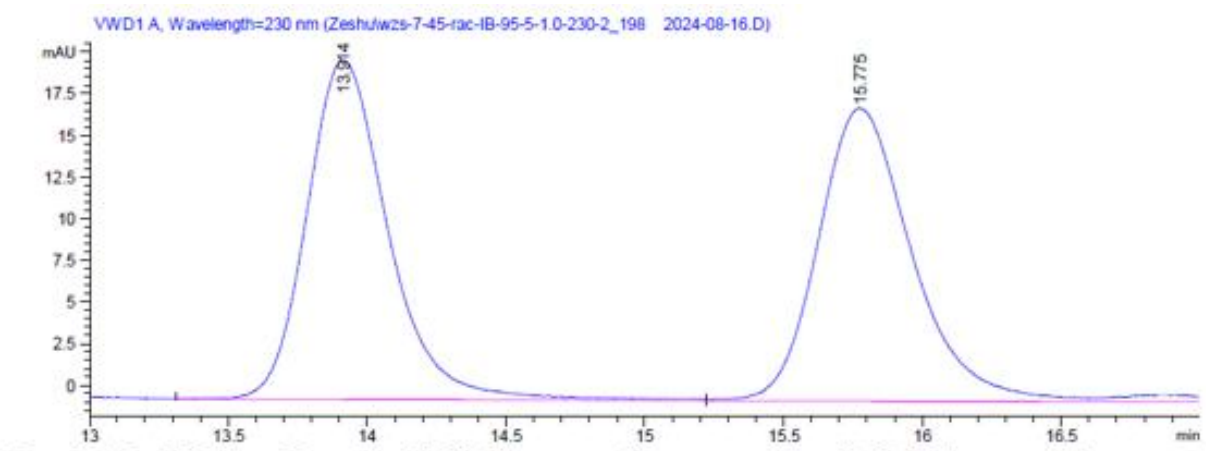

Peak #	RetTime [min]	Type	Width [min]	Area [mAU*s]	Height [mAU]	Area %
1	13.914	BV	0.3116	416.99112	20.37444	49.7334
2	15.775	VV R	0.3555	421.46143	17.54793	50.2666

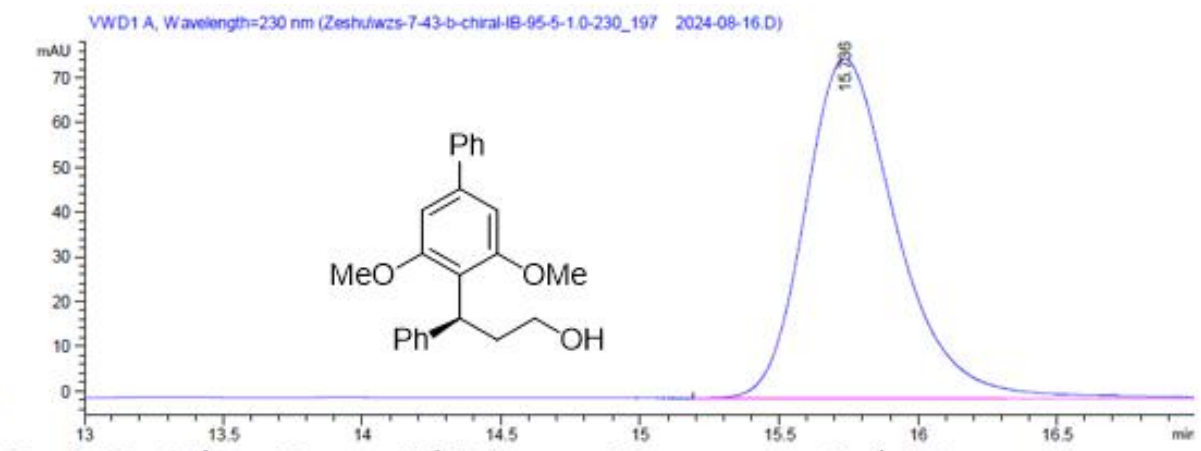

Peak #	RetTime [min]	Type	Width [min]	Area [mAU*s]	Height [mAU]	Area %
1	15.736	BV R	0.3551	1762.48938	75.97144	100.0000

3. The Total Synthesis of Merocyclophane A

3.1. Forward Synthesis of Merocyclophane A

(*E*)-Trimethyl(3-(4,4,5,5-tetramethyl-1,3,2-dioxaborolan-2-yl)prop-1-en-1-yl)silane (14)

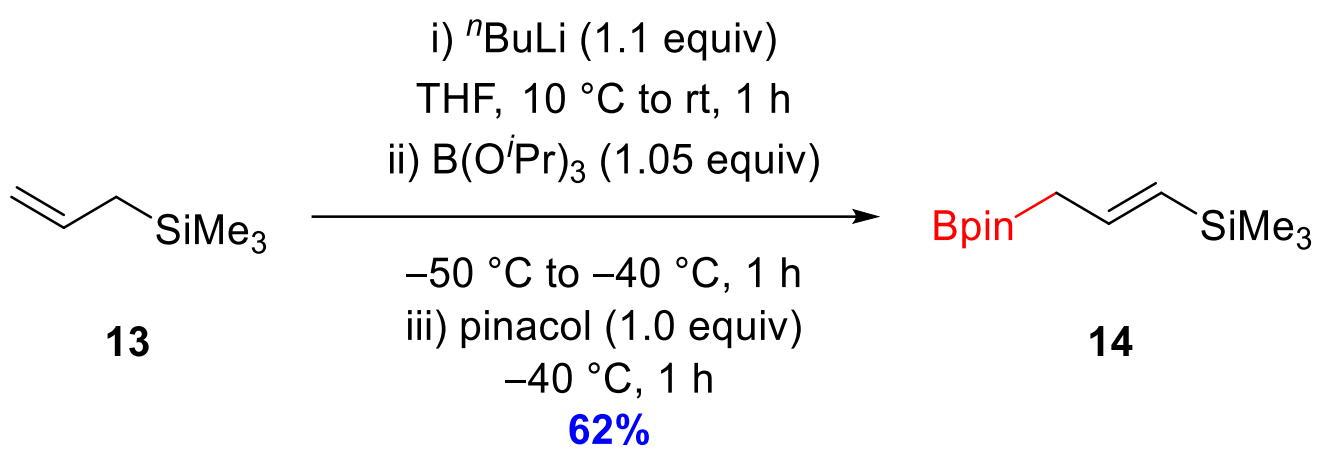

According to a literature procedure.[6] To a flame-dried 250 mL round bottom flask was added allyltrimethylsilane (7.95 mL, 50.0 mmol, 1.00 equiv) and THF (17 mL). The solution was cooled to 10 °C and ^{n}BuLi (24 mL, 55 mmol, 1.1 equiv, 2.3 M in hexane) was added dropwise by syringe pump over 30 min. The reaction mixture was stirred at room temperature for 1 h before cooled down to –50 °C. Triisopropylborate (12.1 mL, 52.5 mmol, 1.05 equiv) was added dropwise by syringe pump over 30 min. The reaction mixture was stirred at this temperature for 30 min before warmed up to –40 °C. A solution of pinacol (5.91 g, 50.0 mmol, 1.00 equiv) in THF (6 mL) was added dropwise by syringe pump over 30 min at –40 °C. The reaction mixture was stirred at this temperature for 30 min before being poured into a solution of AcOH (3.6 g, 60 mmol, 1.2 equiv) in water (14 mL) at 0 °C. The organic layer was separated and the aqueous phase was extracted with Et_2O (70 mL) three times. The combined organic layers were washed with brine (70 mL), dried over $MgSO_4$, filtered, and concentrated under reduced pressure. The crude mixture was distilled under reduced pressure to obtain vinylsilane (**14**) (7.41 g, 31.0 mmol, 62%) as a colourless oil.

R*f* = 0.48 (20:1 hexane/EtOAc, Vanillin)

^{1}H NMR (400 MHz, $CDCl_3$) δ 6.06 (dt, *J* = 18.4, 7.1 Hz, 1H), 5.62 (dt, *J* = 18.4, 1.5 Hz, 1H), 1.81 (dd, *J* = 7.1, 1.5 Hz, 2H), 1.24 (s, 12H), 0.02 (s, 9H) ppm. **^{13}C NMR** (101 MHz, $CDCl_3$) δ 142.2, 130.8, 83.3, 25.0 24.9, –0.9 ppm.

HRMS (EI) m/z calculated for $C_{11}H_{22}BO_2Si$ [M−Me]$^+$, 225.1477, found: 225.1475.

All recorded spectroscopic data matched that previously reported.[7]

(*R*,*E*)-trimethyl(4-(4,4,5,5-tetramethyl-1,3,2-dioxaborolan-2-yl)pent-1-en-1-yl)silane (15)

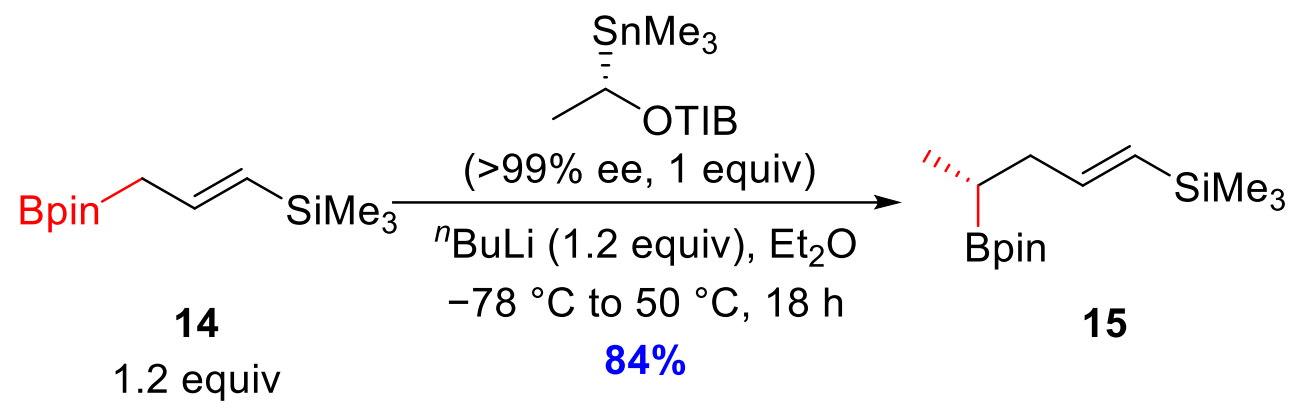

According to a modified literature procedure.[8] To a flame-dried Schlenk tube sealed with a Suba-Seal septum was added (*S*)-1-(trimethylstannyl)ethyl 2,4,6-triisopropylbenzoate (1.37 g, 3.13 mmol, 1.00 equiv) and anhydrous Et_2O (12 mL) under N_2. The resulting solution was cooled to –78 °C (acetone/dry ice). ^{n}BuLi (2.47 mL, 3.76 mmol, 1.20 equiv, 1.52 M in hexane) was added dropwise and the resulting reaction mixture was stirred at –78 °C for 1 h. A solution of vinylsilane (**14**) (903 mg, 3.76 mmol, 1.20 equiv) in anhydrous Et_2O (3 mL) was added dropwise and the vessel was rinsed with additional Et_2O (1 mL). The resulting solution was stirred at –78 °C for 1 h. The reaction mixture was then warmed to room temperature and heated at 50 °C (oil bath) for 18 h. The reaction mixture was cooled to room temperature, filtered through a pad of silica gel and concentrated under reduced pressure. The crude mixture was purified by flash column chromatography (hexane/EtOAc = 50:1) gave the chiral secondary boronic ester **15** (708 mg, 2.64 mmol, 84%, >99:1 er) as a colourless oil.

$\mathbf{R}_f$ = 0.48 (20:1 hexane/EtOAc, Vanillin)

^{1}H NMR (400 MHz, $CDCl_3$) δ 6.02 (dt, *J* = 18.5, 6.3 Hz, 1H), 5.61 (dt, *J* = 18.5, 1.5 Hz, 1H), 2.30 – 2.18 (m, 1H), 2.17 – 2.06 (m, 1H), 1.22 (s, 12H), 1.16 – 1.06 (m, 1H), 0.96 (d, *J* = 7.4 Hz, 3H), 0.02 (s, 9H) ppm. **^{13}C NMR** (101 MHz, $CDCl_3$) δ 147.0, 130.2, 83.0, 40.4, 24.9, 24.9, 15.4, –1.0 ppm.

Specific rotation $[\alpha]_D^{24}$ = −4 (c = 1.0, CH_2Cl_2).

HRMS (EI) m/z calculated for $C_{13}H_{26}BO_2Si$ [M−Me]$^+$, 253.1790, found: 253.1788.

Mosher ester analysis

The enantiomeric ratio was determined after derivatisation to the corresponding Mosher ester which was performed using the following procedure: **15** (42.9 mg, 0.160 mmol) was dissolved in THF (3 mL) and cooled to 0 °C before a pre-mixed solution of 30% aqueous H_2O_2 (0.5 mL) and 3 M aqueous NaOH (1.0 mL) was added slowly. The mixture was warmed to room temperature and stirred overnight. The reaction was cooled to 0 °C and quenched by dropwise

addition of 10% aqueous $Na_2S_2O_3$ (3 mL). The biphasic mixture was warmed to room temperature and the layers separated. The aqueous layer was extracted with Et_2O (6 mL × 3) and the combined organic layers were washed with brine (20 mL) and dried with $MgSO_4$. The reaction mixture was filtered and the solvent removed *in vacuo*. The crude alcohol was purified by flash column chromatography (Et_2O:pentane, 1:3) to furnish the corresponding alcohol.

To the purified alcohol (12.7 mg, 0.080 mmol, 1.00 equiv) was added DMAP (14.7 mg, 0.120 mmol, 1.50 equiv) and DCM (1.5 mL). (R)-(−)-α-Methoxy-α-(trifluoromethyl)phenylacetyl chloride (22.5 µL, 0.120 mmol) was then added and the reaction mixture stirred overnight. The crude reaction mixture was filtered through a plug of silica, eluting with DCM and the solvent was removed *in vacuo*. The crude Mosher ester was then subjected to NMR analysis, comparing against the corresponding racemic sample which was synthesised using *N*,*N*,*N′*,*N′*-tetramethylethylenediamine in place of sparteine (see below).

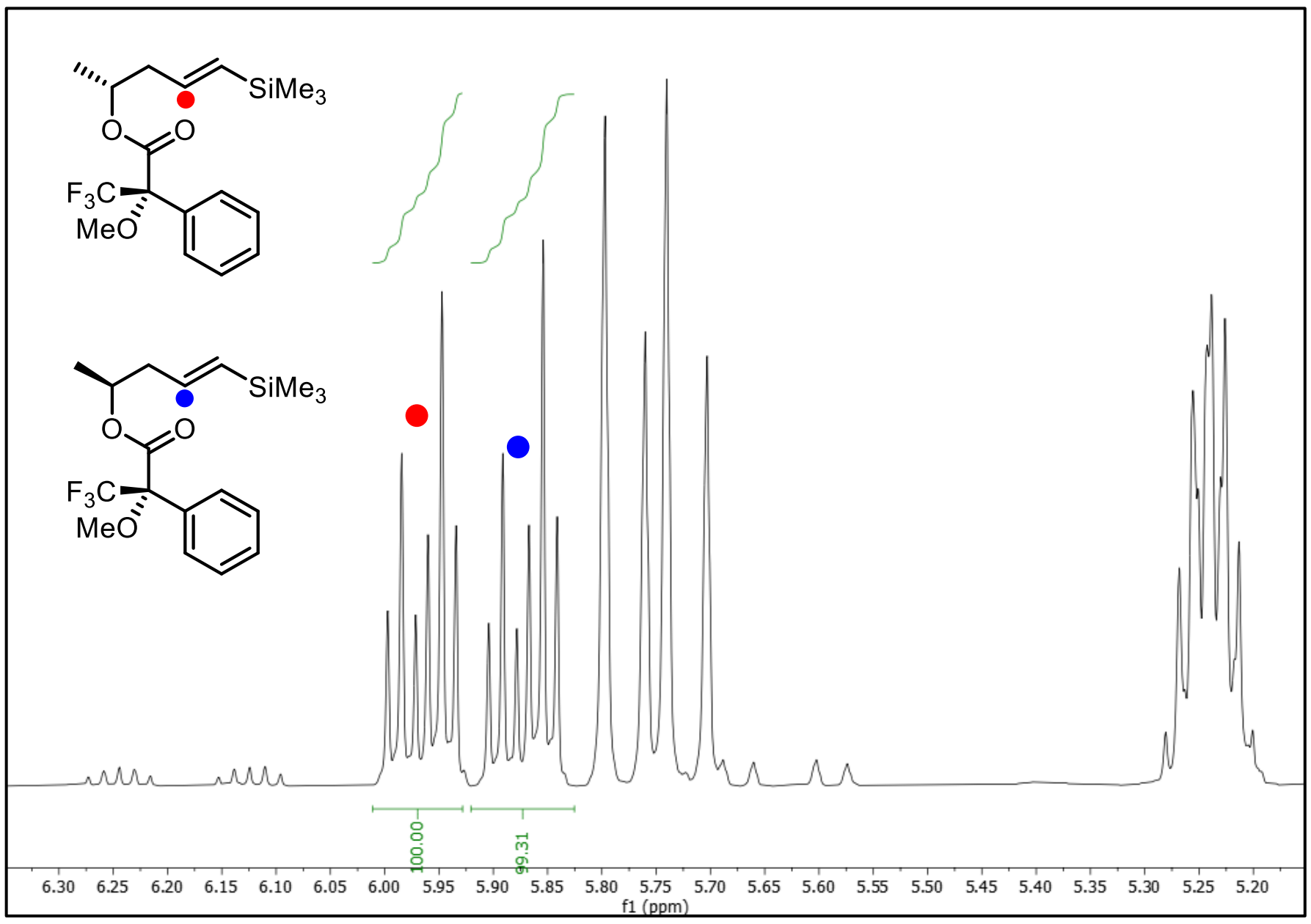

Derived from racemic boronic ester ***15***

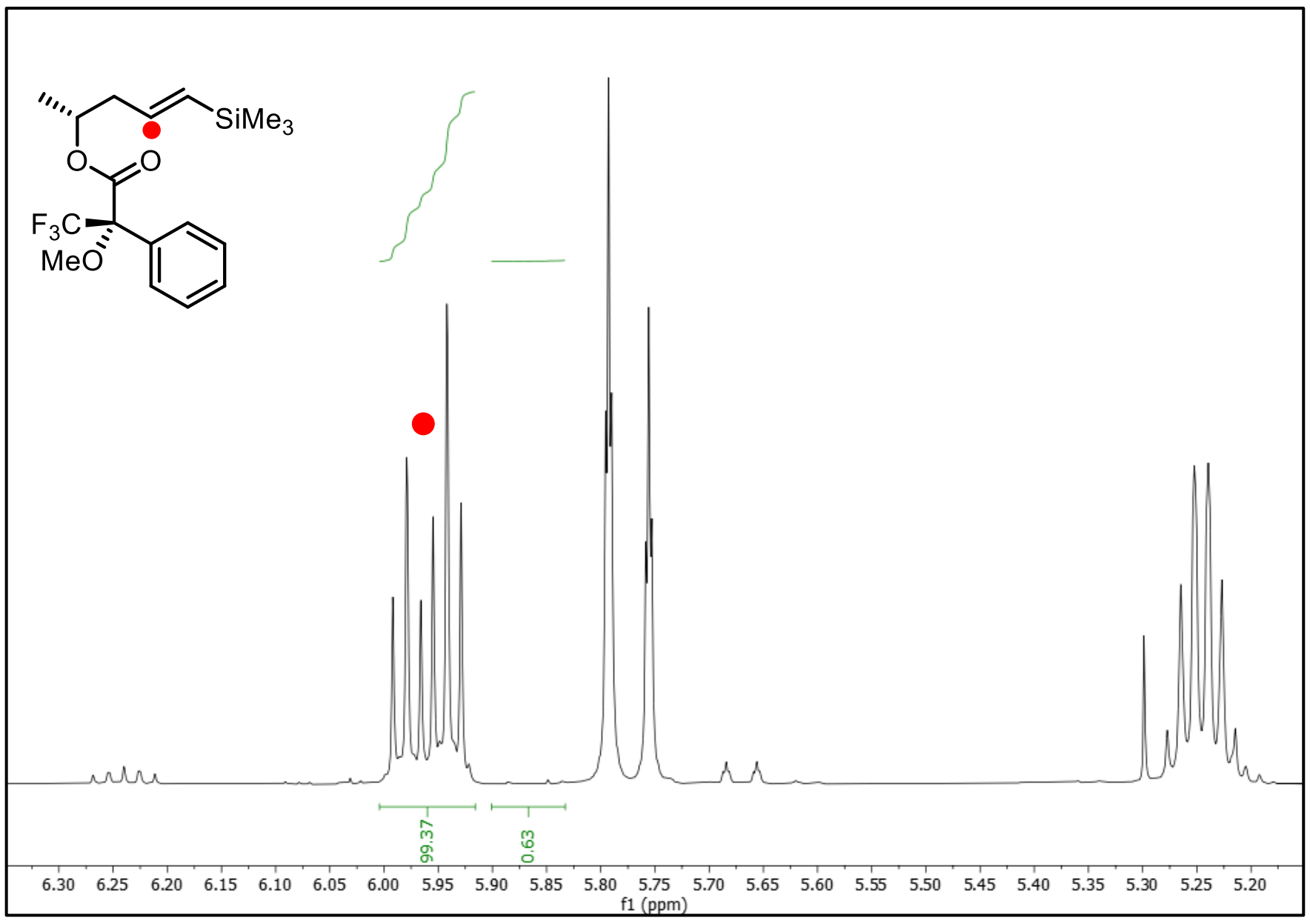

*Derived from enantioenriched boronic ester **15**, synthesised according to the above procedure*

(*R*,*Z*)-2-(5-bromopent-4-en-2-yl)-4,4,5,5-tetramethyl-1,3,2-dioxaborolane (12c)

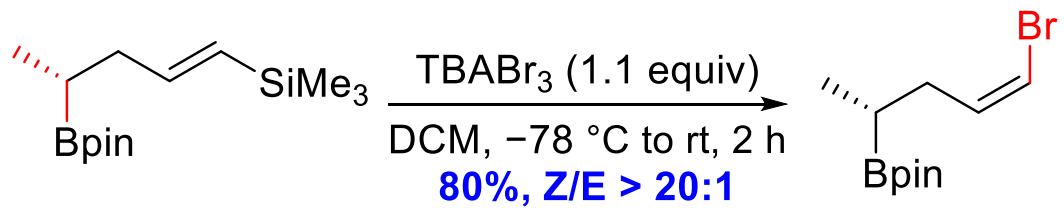

15 **12c**

According to a modified literature procedure.[9] To a flame-dried 50 mL Schlenk tube was added vinylsilane (**15**) (708 mg, 2.64 mmol, 1.00 equiv) and anhydrous DCM (8 mL) under N_2. The resulting solution was cooled to –78 °C (acetone/dry ice). To the solution was added tetrabutylammonium tribromide (1.40 g, 2.90 mmol, 1.10 equiv) in DCM (5 mL) dropwise (Note 1). The reaction mixture was then warmed to room temperature and stirred for 2 h, until TLC indicated complete consumption of the starting material. The reaction was then quenched with aqueous $Na_2S_2O_3$ (5 mL). The organic layer was separated and the aqueous phase was extracted with DCM (15 mL) three times. The combined organic layers were washed with brine (15 mL), dried over $MgSO_4$ and concentrated under reduced pressure. The crude mixture was purified by flash column chromatography (hexane/EtOAc = 50:1) gave *Z*-vinylbromide **12c** (580 mg, 2.11 mmol, 84%, >20:1 *Z*/*E*) as a colourless oil (Note 2 & 3).

Note 1: In the original report, Br_2 was used as the brominating reagent and provided an excellent *Z*/*E* ratio. However, when we used Br_2 under similar conditions, the *Z*/*E* ratio was

lower than 8:1. In contrast, using $TBABr_3$ resulted in a significantly improved *Z*/*E* ratio for the vinyl bromide.

Note 2: The purity of the boronic ester is crucial for the subsequent allylation step. If the product is not pure after chromatography, it can be purified further by distillation.

Note 3: The *Z*/*E* ratio of the vinyl bromide could be calculated by GC-FID analysis.

R_f = 0.50 (20:1 hexane/EtOAc, Vanillin)

^{1}H NMR (500 MHz, $CDCl_3$) δ 6.17 – 6.07 (m, 2H), 2.35 – 2.19 (m, 2H), 1.23 (d, *J* = 1.3 Hz, 12H), 1.19 – 1.12 (m, 1H), 1.00 (d, *J* = 8.8 Hz, 3H) ppm. **^{13}C NMR** (126 MHz, $CDCl_3$) δ 135.0, 107.9, 83.3, 33.1, 24.9, 24.9, 15.3 ppm.

Specific rotation $[\alpha]_D^{24}$ = −4 (c = 1.0, CH_2Cl_2).

HRMS (ESI) m/z calculated for $C_{10}H_{17}BBrO_2$ $[M-Me]^+$, 259.0499, found: 259.0497.

2-((*S*)-1-(4-((*R*,*Z*)-5-bromopent-4-en-2-yl)-2,6-dimethoxyphenyl)allyl)thiophene (11c)

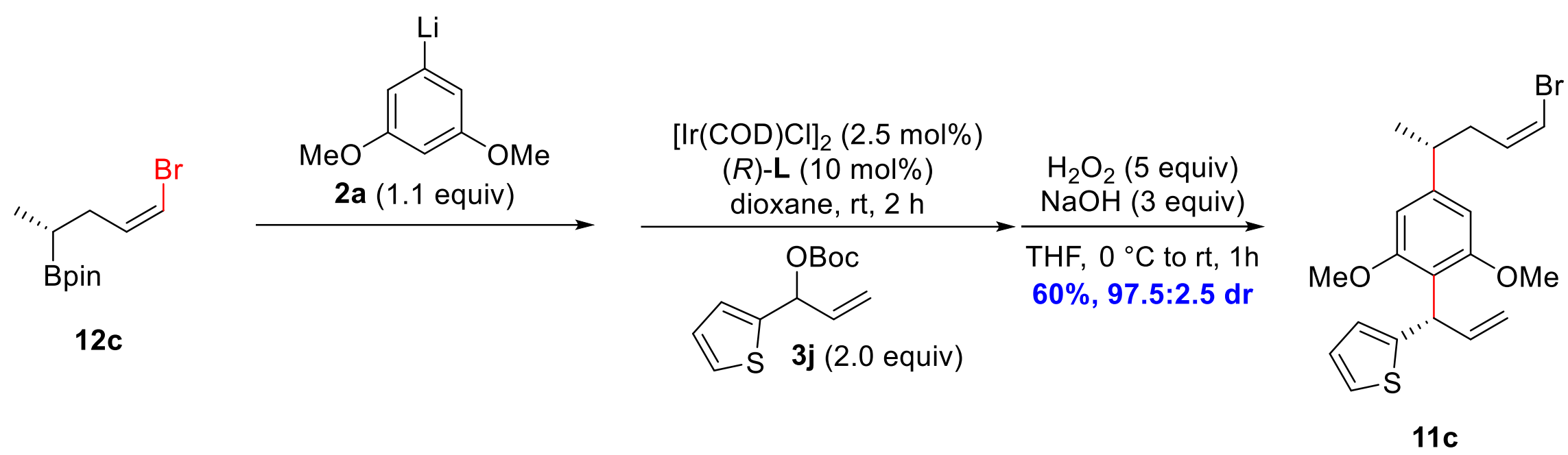

Prepared following **General Procedure A** using boronic ester **12c** (385 mg, 1.40 mmol, 1.00 equiv) and allyl carbonate **3j** (673 mg, 2.80 mmol, 2.00 equiv) with (*R*)-**L**. Purification by flash column chromatography (hexane/toluene = 5:1) gave the title compound (343 mg, 60%, 97.5:2.5 dr) as a pale-yellow oil.

R_f = 0.52 (2:1 hexane/toluene, UV, Vanillin)

^{1}H NMR (500 MHz, $CDCl_3$) δ 7.08 (dd, *J* = 5.1, 1.3 Hz, 1H), 6.88 (dd, *J* = 5.1, 3.5 Hz, 1H), 6.79 – 6.73 (m, 1H), 6.67 – 6.56 (m, 1H), 6.42 (s, 2H), 6.18 (dt, *J* = 6.9, 1.5 Hz, 1H), 6.02 (q, *J* = 7.0 Hz, 1H), 5.52 (d, *J* = 8.0 Hz, 1H), 5.19 (dt, *J* = 17.2, 1.6 Hz, 1H), 5.09 (ddd, *J* = 9.9, 1.8, 0.9 Hz, 1H), 3.78 (s, 6H), 2.90 – 2.79 (m, 1H), 2.54 – 2.46 (m, 2H), 1.30 (d, *J* = 6.9 Hz,

3H) ppm. **^{13}C NMR** (126 MHz, $CDCl_3$) δ 158.0, 148.7, 146.9, 139.9, 139.9, 133.5, 126.3, 123.8, 123.0, 117.9, 115.3, 108.8, 103.6, 56.0, 40.7, 39.6, 38.1, 21.9 ppm.

Specific rotation $[\alpha]_D^{24}$ = +10 (c = 1.0, CH_2Cl_2).

HRMS (ESI) m/z calculated for $C_{20}H_{24}BrO_2S$ $[M+H]^+$, 407.0675, found: 407.0679.

The diastereomeric ratio (dr) was determined after Suzuki coupling of the vinyl bromide with (4-hydroxyphenyl)boronic acid according to **General Procedure C**. **HPLC conditions**: Chiral column IA, hexane: isopropanol = 98:2, flow rate = 1.0 mL/min, wavelength = 254 nm, t_R = 64.8 min for major isomer, t_R = 72.3 min for minor isomer.

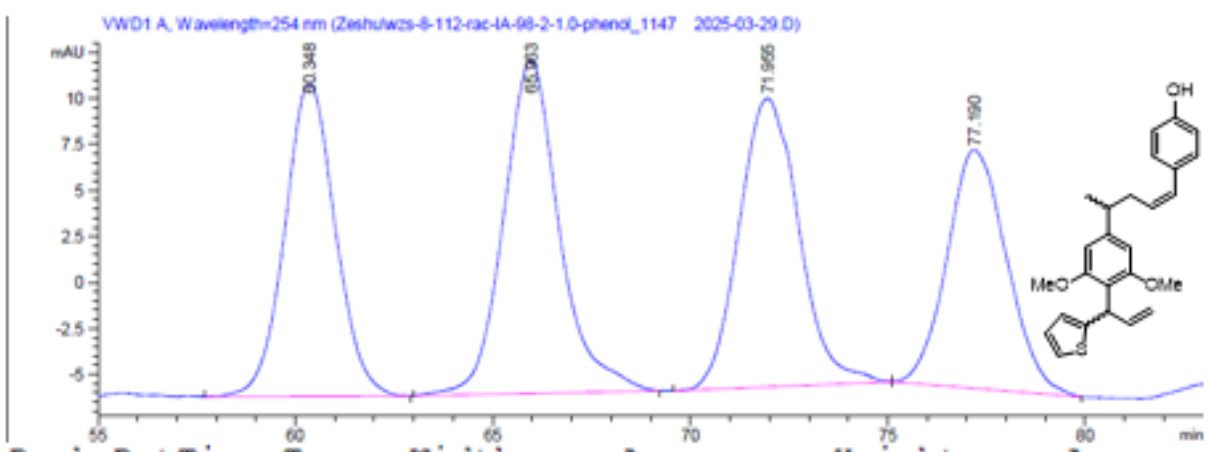

Peak #	RetTime [min]	Type	Width [min]	Area [mAU*s]	Height [mAU]	Area %
1	60.348	BB	1.3303	1517.78784	16.99329	24.0233
2	65.963	BB	1.4113	1781.57751	18.13175	28.1985
3	71.955	BB	1.4946	1652.77124	15.68116	26.1598
4	77.190	BB	1.4295	1365.84937	12.97010	21.6184

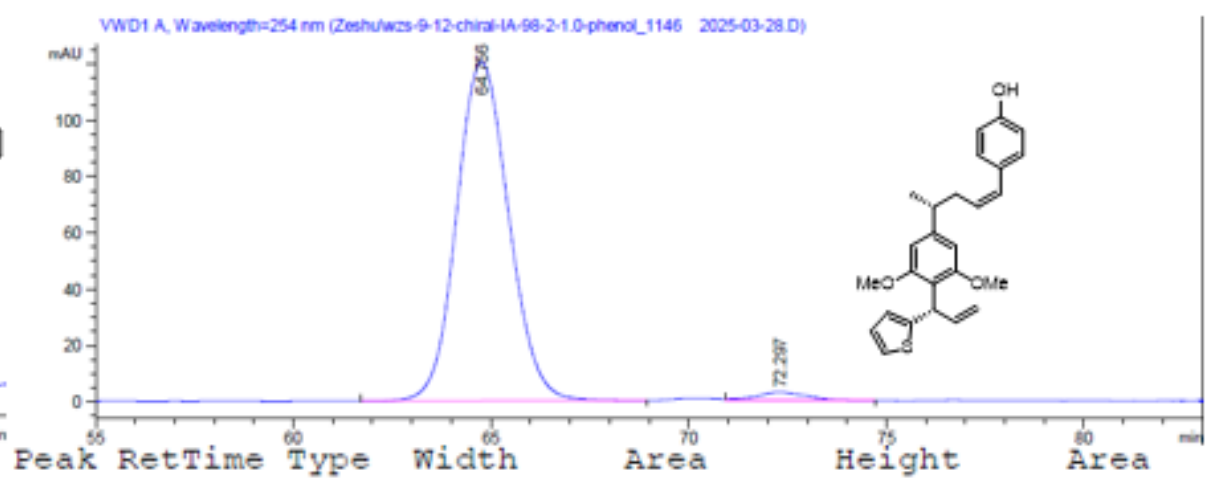

Peak #	RetTime [min]	Type	Width [min]	Area [mAU*s]	Height [mAU]	Area %
1	64.756	BB	1.4037	1.10447e4	120.56242	97.6241
2	72.297	VB	1.0810	268.79971	2.96502	2.3759

2,2'-((2*S*,5*Z*,8*R*,10*S*,13*Z*,16*R*)-1^2,1^6,9^3,9^5-tetramethoxy-8,16-dimethyl-1,9(1,4)-dibenzenacyclohexadecaphane-5,13-diene-2,10-diyl)dithiophene (10)

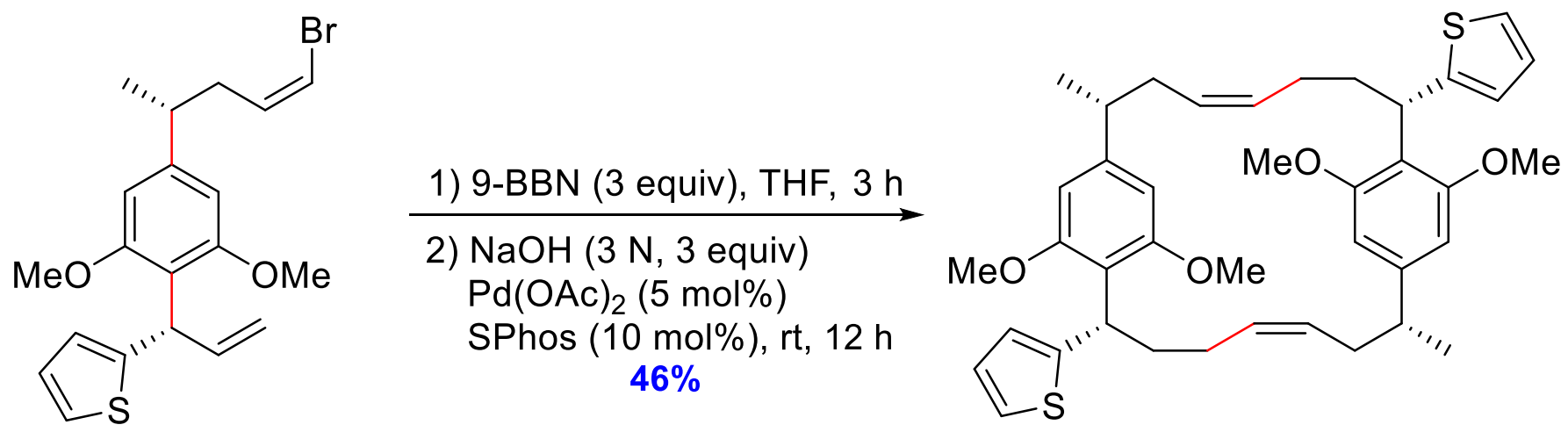

11c **10**

The *B*-alkyl Suzuki coupling was performed according to a modified literature procedure.[10] To a 7 mL vial in a glove box was added vinyl bromide **11c** (20.4 mg, 0.0500 mmol, 1.00 equiv) and anhydrous THF (0.65 mL). The vial was sealed with a septum and removed from the glovebox. A solution of 9-borabicyclo[3.3.1]nonane solution (0.6 mL, 0.3 mmol, 3 equiv, 0.5 M in THF) was then added to the reaction dropwise at 0 °C. The reaction was stirred at room temperature for 3 h before aqueous 3 N NaOH (50 µL, 0.15 mmol, 3.0 equiv) was added. In a separate 50 mL Schlenk tube, $Pd(OAc)_2$ (0.60 mg, 2.5 µmol, 5.0 mol%,), S-Phos (2.1 mg, 5.0

μmol, 10 mol%,) and THF (1 mL) were combined and stirred at room temperature for 10 min before diluted with an additional THF (8 mL). The R-BBN solution was then added to the catalyst solution by syringe pump over 30 min and the vessel was rinsed with additional THF (1 mL). The reaction mixture was then stirred at room temperature for 12 h. Aqueous 30% H_2O_2 (0.1 mL) was added and stirred for 1 h followed by aqueous $Na_2S_2O_3$ (~0.5 mL) at 0 °C. The organic layer was separated, and the aqueous phase was extracted with Et_2O (3 mL) three times. The combined organic layers were washed with brine (3 mL), dried over $MgSO_4$, filtered, and concentrated under reduced pressure. The crude mixture was purified by flash column chromatography (hexane/EtOAc = 20:1) gave dimer **10** (7.6 mg, 0.012 mmol, 46%) as white solid (Note 1&2).

Note 1: Trimer and tetramer side products could be observed by TLC under alternative reaction conditions (Table S4). These oligomers are more polar than the desired dimer. Notably, the use of $Pd(PPh_3)_4$ in combination with S-Phos as the catalyst system significantly suppressed formation of these side products.

Note 2: As an alternative purification method, preparative thin-layer chromatography using hexane/EtOAc as the eluent can be employed to isolate the desired compound.

R_f = 0.59 (5:1 hexane/EtOAc, UV, Vanillin)

^{1}H NMR (500 MHz, $CDCl_3$) δ 7.02 (dd, *J* = 5.1, 1.3 Hz, 2H), 6.88 – 6.82 (m, 2H), 6.82 – 6.77 (m, 2H), 6.25 (s, 4H), 5.53 – 5.43 (m, 2H), 5.43 – 5.32 (m, 2H), 4.84 (dd, *J* = 12.4, 5.1 Hz, 2H), 3.74 (s, 12H), 2.70 – 2.57 (m, 2H), 2.56 – 2.46 (m, 2H), 2.09 – 1.96 (m, 2H), 1.96 – 1.83 (m, 4H), 1.84 – 1.72 (m, 2H), 1.73 – 1.60 (m, 2H), 1.09 (d, *J* = 6.9 Hz, 6H) ppm. **^{13}C NMR** (126 MHz, $CDCl_3$) δ 158.1*, 150.6, 148.3, 131.0, 128.5, 126.1, 123.2, 122.4, 116.4, 104.3*, 101.7*, 56.5*, 55.0*, 40.0, 36.3, 34.8, 33.7, 25.8, 19.4 ppm. (**the signals of ^{13}C NMR showed broad peak due to rotation in macrocycle*)

Specific rotation $[\alpha]_D^{24}$ = − 20 (c = 1.0, CH_2Cl_2).

HRMS (ESI) m/z calculated for $C_{40}H_{49}O_4S_2$ $[M+H]^+$, 657.3067, found: 657.3035.

(2*S*,8*R*,10*S*,16*R*)-2,10-dibutyl-8,16-dimethyl-1,9(1,4)-dibenzenacyclohexadecaphane-1^2,1^6,9^3,9^5-tetraol (6)

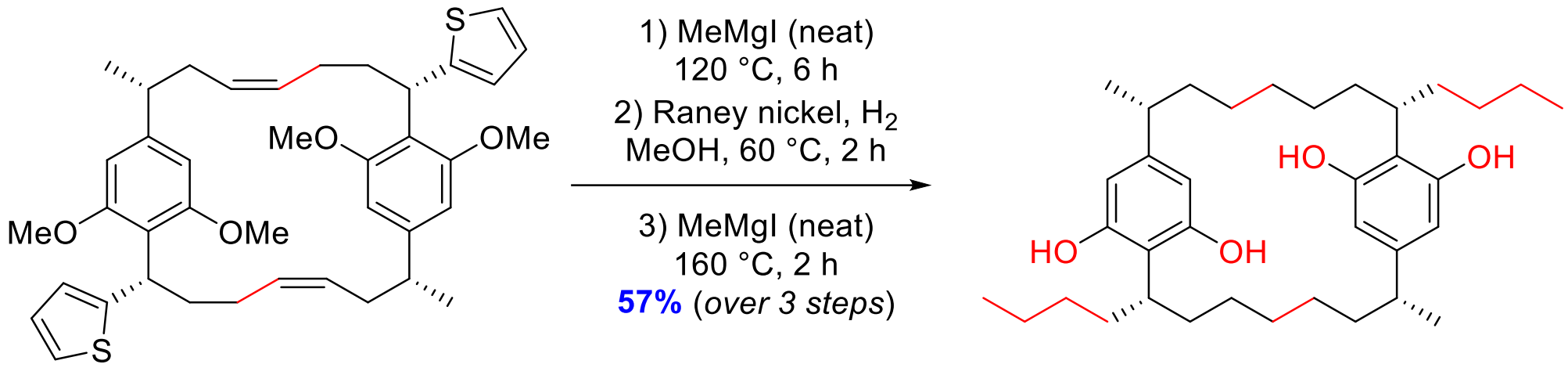

The final three steps of the synthesis were performed according to modified literature procedures.[11-12] **Step 1 partial demethylation**: In a flame-dried 20 mL Schlenk tube equipped with a magnetic stir bar was added dimer **10** (16.4 mg, 0.0250 mmol, 1.00 equiv) and MeMgI (3.75 mL, 11.3 mmol, 450 equiv, 3 M in Et_2O) under N_2 (Note 1). The reaction was stirred at room temperature until it became homogenous. The solvent was removed carefully under vacuum, and the tube was back-filled with N_2. The reaction was subsequently stirred at 120 ºC for 6 h (Note 2) before cooled down to room temperature. Anhydrous Et_2O (5 mL) was added to the tube under N_2 and the mixture was sonicated for approximately 5 minutes until it became homogeneous. The resulting solution was transferred to pre-cooled Et_2O/H_2O (1:1, 30 mL) at 0 ºC dropwise and the vessel was rinsed with additional Et_2O (5 mL). The resulting mixture was allowed to warm to room temperature, then acidified using 1.0 N aqueous HCl (10 mL) until precipitate dissolved. The layers were separated and the aqueous layer was extracted successively with EtOAc (4 × 20 mL), followed by CH_2Cl_2/MeOH (19:1, 4 × 20 mL). The combined organic layers were dried over anhydrous Na_2SO_4, filtered, and concentrated in vacuo. The crude residue was purified by preparative thin-layer chromatography using hexane/EtOAc (5:1) to afford the partially demethylated product (Note 3). **Step 2 reduction**: To a solution of the above crude product in MeOH (2.0 mL) in a 10 mL Schlenk tube was added Raney nickel (slurry in water, 0.2 mL). Then the tube was charged with 1 atm of H_2 by freeze-pump-thaw (FPT) and the reaction mixture was stirred at 60 ºC for 2 h. Then the reaction mixture was filtered through a plug of celite (Note 4) and washed with MeOH and EtOAc. The filtrate was then concentrated in vacuo to obtain the crude mixture. **Step 3 demethylation**: According to **Step 1**, in a flame-dried 20 mL Schlenk tube equipped with a magnetic stir bar was added the above crude material and MeMgI (3.75 mL, 11.3 mmol, 450 equiv, 3 M in Et_2O) under N_2 (Note 1). The reaction was stirred at room temperature until it became homogenous. The solvent was removed carefully under vacuum, and the tube was back-filled with N_2. The

reaction was subsequently stirred at 160 ºC for 2 h (Note 2) before being cooled down to room temperature. Anhydrous Et_2O (5 mL) was added to the tube under N_2 and the mixture was sonicated for approximately 5 minutes until it became homogeneous. The resulting solution was transferred to pre-cooled Et_2O/H_2O (1:1, 30 mL) at 0 ºC dropwise and the vessel was rinsed with additional Et_2O (5 mL). The resulting mixture was allowed to warm to room temperature, then acidified using 1.0 N aqueous HCl (10 mL) until the precipitate dissolved. The layers were separated and the aqueous layer was extracted successively with EtOAc (4 × 20 mL), followed by CH_2Cl_2/MeOH (19:1, 4 × 20 mL). The combined organic layers were dried over anhydrous Na_2SO_4, filtered, and concentrated in vacuo. The crude residue was purified by flash column chromatography (hexane/EtOAc = 5:1) gave merocyclophane A **6** (7.9 mg, 0.0143 mmol, 57%) as white solid (Note 5).

Note 1: Prior to use, dimer **10** was azeotropically dried with toluene (3×) to ensure removal of residual moisture.

Note 2: When the mixture of dimer **10** and the Grignard reagent was stirred under vacuum as the previous report described,[11] a viscous solution formed that caused the stir bar to stop, resulting in low conversion. In contrast, stirring at elevated temperature under positive nitrogen pressure maintained smooth agitation and significantly improved the conversion.

Note 3: A small amount of unreacted starting material is typically present in the crude product. Compounds with at least one methyl group removed can be clearly distinguished by TLC and were isolated by preparative TLC.

Note 4: Raney nickel is a pyrophoric material in its dry form. During filtration through a Celite plug, it is essential to keep the surface of the Celite continuously moist with solvent (e.g., MeOH or EtOAc) to prevent exposure to air.

Note 5: Merocyclophane A exhibits weak UV absorption but responds strongly to vanillin staining.

$\mathbf{R}_{\boldsymbol{f}}$ = 0.39 (5:1 hexane/EtOAc, Vanillin)

^{1}H NMR (500 MHz, CD_3OD) δ 6.02 (d, J = 18.7 Hz, 4H), 3.15 – 3.06 (m, 2H), 2.36 – 2.25 (m, 2H), 2.03 – 1.85 (m, 4H), 1.55 – 1.39 (m, 4H), 1.36 – 1.06 (m, 20H), 1.05 – 0.86 (m, 6H), 0.83 (t, J = 7.1 Hz, 6H), 0.75 – 0.61 (m, 4H) ppm. **^{13}C NMR** (126 MHz, CD_3OD) δ 158.6, 157.0, 146.8, 116.3, 109.2, 104.6, 42.0, 40.7, 36.9, 35.4, 34.9, 32.7, 31.8, 30.9, 30.7, 24.0, 23.6, 14.6 ppm.

Specific rotation $[\alpha]_D^{22} = -29.5$ (c = 0.6, CH_3OH).

HRMS (ESI) m/z calculated for $C_{36}H_{56}NaO_4$ $[M+Na]^+$, 575.4071, found: 575.4088.

All recorded spectroscopic data matched that previously reported.[13a]

3.2. Synthesis of *E*-Vinyl Halides (11a/b)

(*R*,*E*)-trimethyl(4-(4,4,5,5-tetramethyl-1,3,2-dioxaborolan-2-yl)pent-1-en-1-yl)silane (15)

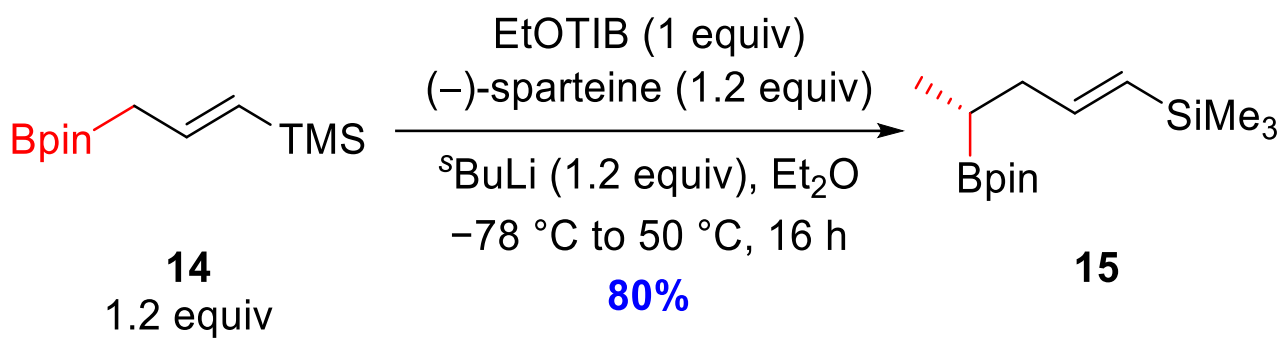

According to a modified literature procedure.[8] Ethyl 2,4,6-triisopropylbenzoate (1.38 g, 5.00 mmol, 1.00 equiv) and (−)-sparteine (1.38 mL, 6.00 mmol, 1.20 equiv) were added to a 100 mL flame-dried Schlenk tube under N_2. Anhydrous Et_2O (30 mL, 0.17 M) was added and the resulting solution was cooled to –78 °C (acetone/dry ice). *s*BuLi (4.32 mL, 6.00 mmol, 1.20 equiv, 1.39 M in hexane) was added dropwise and the resulting reaction mixture was stirred at –78 °C for 5 h. A solution of boronic ester **14** (1.44 g, 6.00 mmol, 1.20 equiv) in anhydrous Et_2O (5 mL) was added dropwise, and the vessel was rinsed with additional Et_2O (1 mL). The resulting solution was stirred at –78 °C for 1 h, warmed to room temperature and then heated at 50 °C (oil bath) for 16 h. The reaction mixture was cooled to room temperature and quenched with a 2 N aqueous HCl (30 mL). The layers were separated and the aqueous phase was washed with Et_2O (30 mL) three times. The combined organic layers were washed with 2 N HCl (30 mL), and the combined aqueous phases were retained for (−)-sparteine recovery. The organic phase was washed with water (30 mL), brine (30 mL), dried over $MgSO_4$, filtered, and concentrated under reduced pressure. The crude mixture was purified by flash column chromatography (hexane/toluene = 5:1) gave secondary boronic ester **15** (1.07 g, 4.0 mmol, 80%, 96:4 er) as a colourless oil (Note).

Note: Residual ethyl 2,4,6-triisopropylbenzoate (EtOTIB) is commonly observed and is often inseparable by flash chromatography with hexane/EtOAc. However, using hexane/toluene as the eluent allowed for the efficient removal of EtOTIB, typically after 2–3 rounds of chromatography.

Mosher ester analysis

The enantiomeric ratio was determined after derivatisation to the corresponding Mosher ester which was performed using the following procedure: **15** (42.9 mg, 0.160 mmol) was dissolved in THF (3 mL) and cooled to 0 °C before a pre-mixed solution of 30% aqueous H_2O_2 (0.5 mL) and 3 M aqueous NaOH (1.0 mL) was added slowly. The mixture was warmed to room temperature and stirred overnight. The reaction was cooled to 0 °C and quenched by dropwise addition of 10% aqueous $Na_2S_2O_3$ (3 mL). The biphasic mixture was warmed to room temperature and the layers separated. The aqueous layer was extracted with Et_2O (6 mL × 3) and the combined organic layers were washed with brine (20 mL) and dried with $MgSO_4$. The reaction mixture was filtered and the solvent removed *in vacuo*. The crude alcohol was purified by flash column chromatography (Et_2O:pentane, 1:3) to furnish the corresponding alcohol.

To the purified alcohol (12.7 mg, 0.080 mmol, 1.0 equiv) was added DMAP (14.7 mg, 0.120 mmol, 1.5 equiv) and DCM (1.5 mL). (R)-(−)-α-Methoxy-α-(trifluoromethyl)phenylacetyl chloride (22.5 µL, 0.120 mmol) was then added and the reaction mixture stirred overnight. The crude reaction mixture was filtered through a plug of silica, eluting with DCM and the solvent was removed *in vacuo*. The crude Mosher ester was then subjected to NMR analysis, comparing against the corresponding racemic sample which was synthesised using *N*,*N*,*N*′,*N*′-tetramethylethylenediamine in place of sparteine (see below).

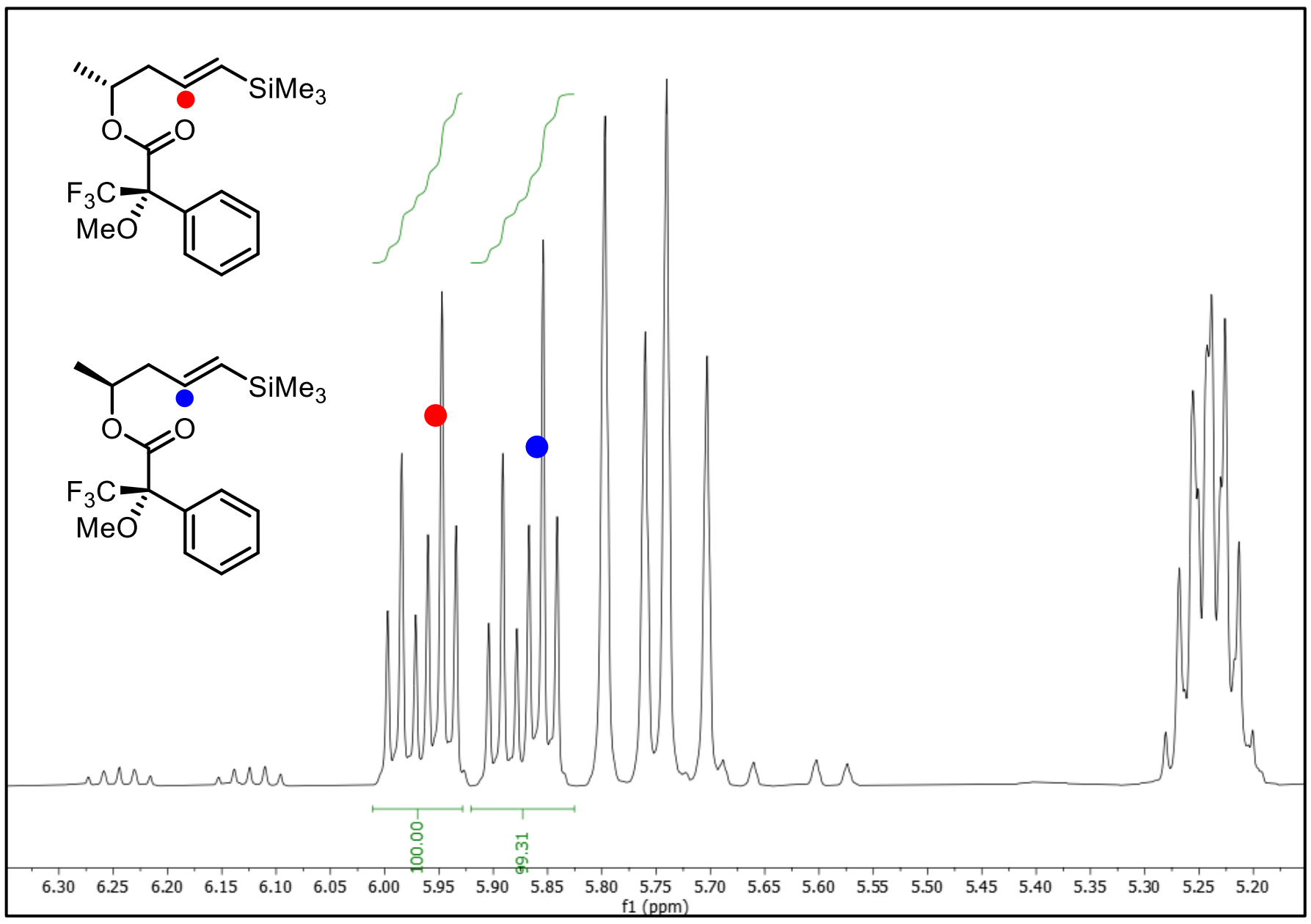

*Derived from racemic boronic ester **15***

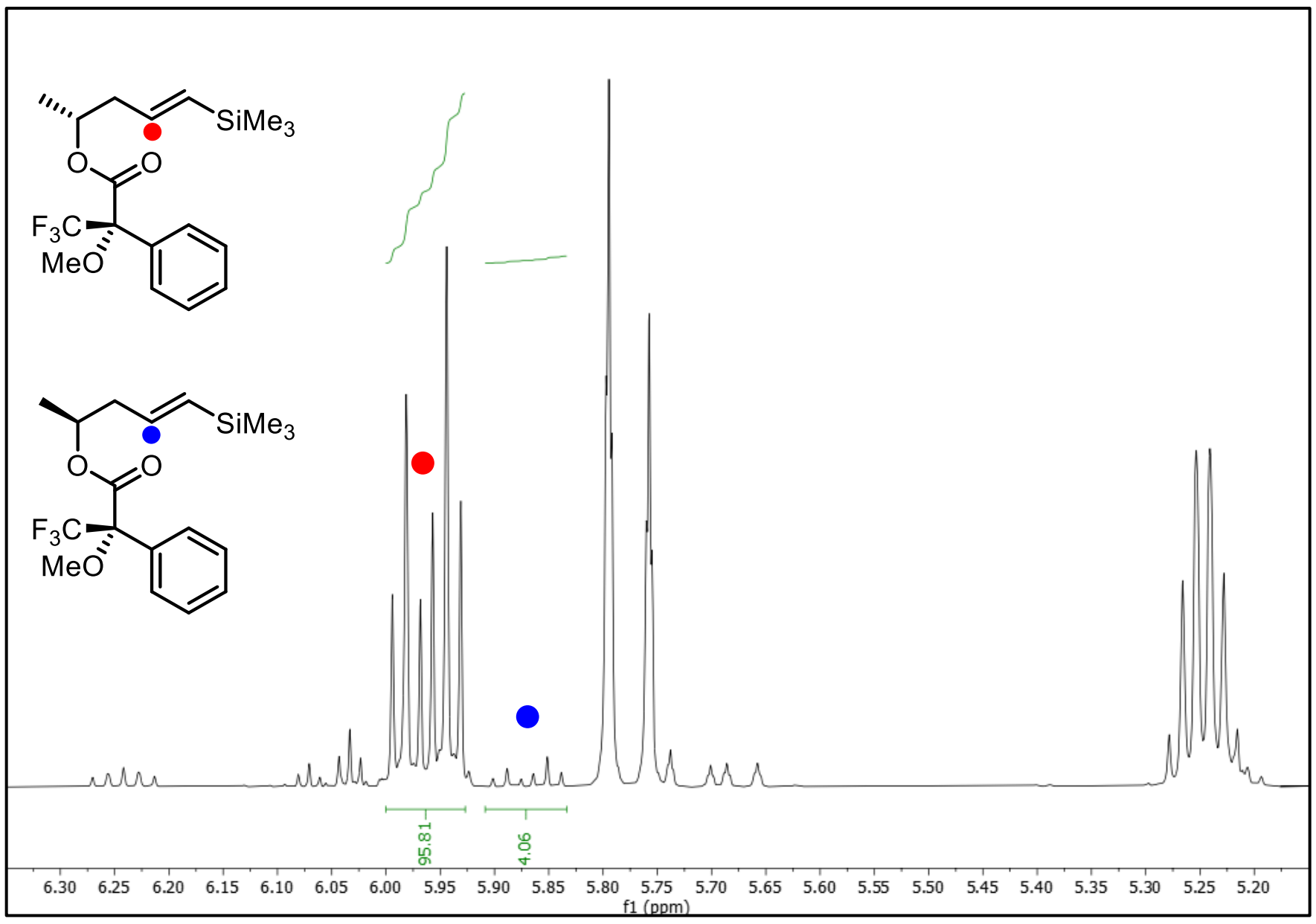

*Derived from enantioenriched boronic ester **15**, synthesised according to the above procedure*

(*R*,*E*)-2-(5-bromopent-4-en-2-yl)-4,4,5,5-tetramethyl-1,3,2-dioxaborolane (12a)

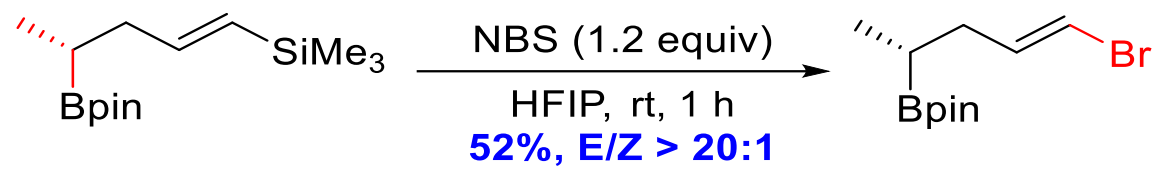

15 **12a**

According to a modified literature procedure.[14] To a flame-dried 25 mL round-bottom flask equipped with a magnetic stir bar in a glove box, was added (*E*)-vinylsilane **15** (268 mg, 1.00 mmol, 1.00 equiv) and hexafluoroisopropanol (HFIP, 10 mL). *N*-bromosuccinimide (214 mg, 1.2 mmol, 1.2 equiv) was then added, the flask was sealed with a septum, and removed from glove box. The reaction mixture was stirred at room temperature for 1 h before being quenched with aqueous $Na_2S_2O_3$ (1 mL). HFIP was removed under reduced pressure and the resulting residue was diluted with DCM (5 mL) and water (5 mL). The organic layer was separated and the aqueous phase was extracted with DCM (5 mL) three times. The combined organic layers were washed with brine (5 mL), dried over anhydrous $MgSO_4$, filtered, and concentrated under reduced pressure. The crude mixture was purified by flash column chromatography (hexane/EtOAc = 50:1) gave (*E*)-vinylbromide **12a** (142 mg, 0.52 mmol, 52%, >20:1 *E*/*Z*) as a colourless oil (Note).

Note: The *E*/*Z* ratio of the vinyl bromide could be calculated by GC-FID analysis.

R_f = 0.41 (20:1 hexane/EtOAc, Vanillin)

^{1}H NMR (500 MHz, $CDCl_3$) δ 6.17 (dt, *J* = 13.4, 7.5 Hz, 1H), 6.01 (dt, *J* = 13.4, 1.4 Hz, 1H), 2.22 – 2.11 (m, 1H), 2.10 – 1.99 (m, 1H), 1.23 (s, 12H), 1.17 – 1.08 (m, 1H), 0.97 (d, *J* = 7.4 Hz, 3H) ppm. **^{13}C NMR** (126 MHz, $CDCl_3$) δ 138.0, 104.6, 83.3, 36.4, 24.9, 24.9, 15.1 ppm.

Specific rotation $[\alpha]_D^{24}$ = −4 (c = 1.0, CH_2Cl_2).

HRMS (ESI) m/z calculated for $C_{10}H_{17}BBrO_2$ $[M-Me]^+$, 259.0499, found: 259.0497.

2-((*S*)-1-(4-((*R*,*E*)-5-bromopent-4-en-2-yl)-2,6-dimethoxyphenyl)allyl)thiophene (11a)

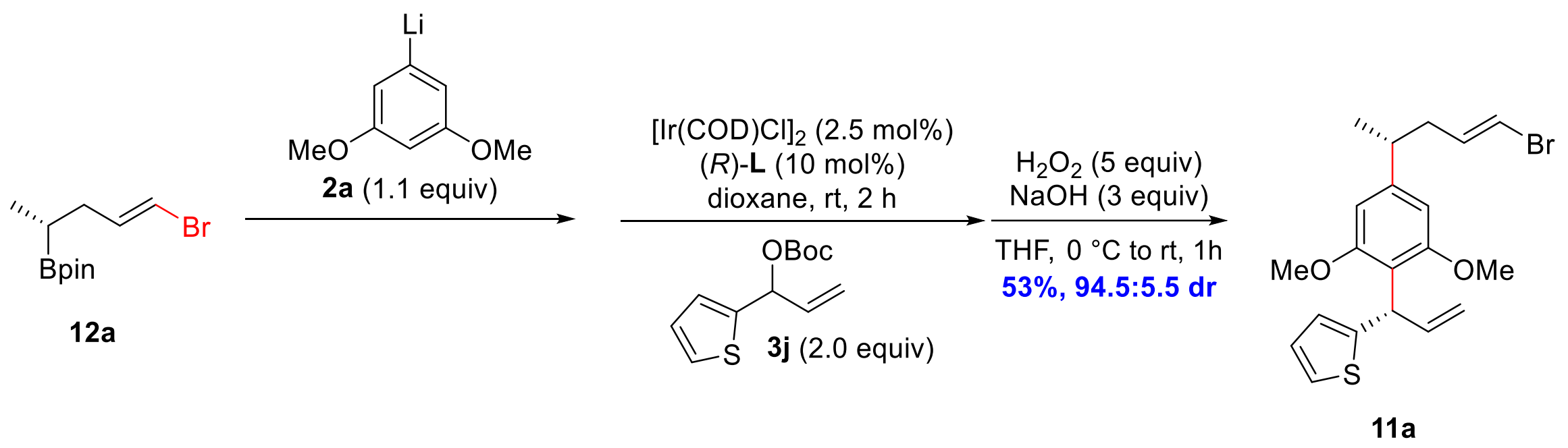

Prepared following **General Procedure A** using boronic ester **12a** (110 mg, 0.400 mmol, 1.00 equiv) and carbonate **3j** (192 mg, 0.800 mmol, 2.00 equiv) with (*R*)-**L**. Purification by flash column chromatography (hexane/toluene = 5:1) gave the title compound (86.4 mg, 53%, 94.5:5.5 dr) as a pale-yellow solid.

R_f = 0.63 (2:1 hexane/toluene, UV, Vanillin)

^{1}H NMR (500 MHz, $CDCl_3$) δ 7.08 (dd, *J* = 5.1, 1.2 Hz, 1H), 6.88 (dd, *J* = 5.1, 3.5 Hz, 1H), 6.79 – 6.73 (m, 1H), 6.61 (ddd, *J* = 17.5, 9.9, 8.0 Hz, 1H), 6.37 (s, 2H), 6.20 – 6.08 (m, 1H), 6.03 (d, *J* = 13.5 Hz, 1H), 5.52 (dd, *J* = 8.0, 1.3 Hz, 1H), 5.19 (dt, *J* = 17.0, 1.5 Hz, 1H), 5.09 (ddd, *J* = 9.8, 1.8, 0.8 Hz, 1H), 3.77 (s, 6H), 2.81 – 2.71 (m, 1H), 2.39 – 2.21 (m, 2H), 1.26 (d, *J* = 6.9 Hz, 3H) ppm. **^{13}C NMR** (126 MHz, $CDCl_3$) δ 158.0, 148.7, 146.6, 139.9, 136.4, 126.3, 123.7, 123.0, 118.0, 115.4, 105.6, 103.6, 56.0, 41.7, 40.7, 40.0, 21.2 ppm.

Specific rotation $[\alpha]_D^{24}$ = −30 (c = 1.0, CH_2Cl_2).

HRMS (ESI) m/z calculated for $C_{20}H_{24}BrO_2S$ $[M+H]^+$, 407.0675, found: 407.0672.

The diastereomeric ratio (dr) was determined after Suzuki coupling of the vinyl bromide with (4-hydroxyphenyl)boronic acid according to **General Procedure C**. **HPLC conditions**: Chiral

column IA, hexane: isopropanol = 95:5, flow rate = 1.0 mL/min, wavelength = 230 nm, t_R = 10.2 min for major isomer.

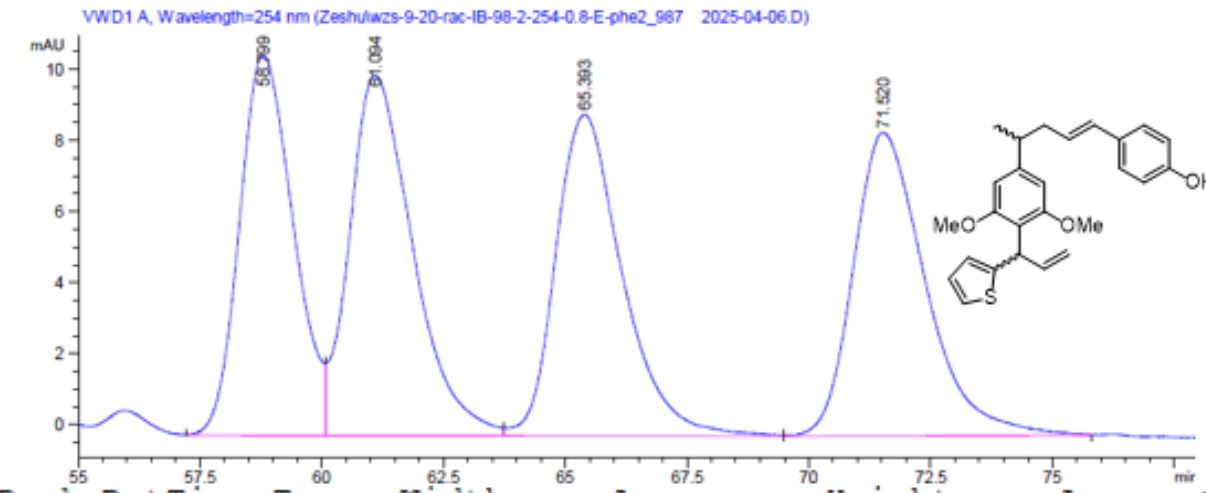

Peak #	RetTime [min]	Type	Width [min]	Area [mAU*s]	Height [mAU]	Area %
1	58.799	BV	1.1671	819.95593	10.70813	23.6084
2	61.094	VV	1.2612	918.04333	10.12939	26.4326
3	65.393	VB	1.3653	859.12158	9.03724	24.7361
4	71.520	BB	1.3376	876.03357	8.53234	25.2230

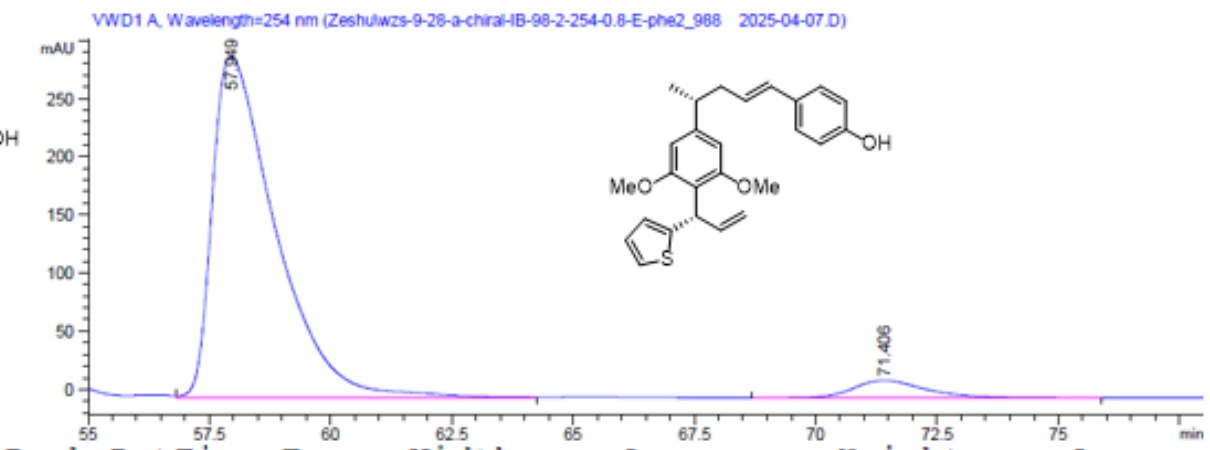

Peak #	RetTime [min]	Type	Width [min]	Area [mAU*s]	Height [mAU]	Area %
1	57.949	VB	1.3494	2.65128e4	293.12454	94.5349
2	71.406	BB	1.5033	1532.71484	14.78872	5.4651

(*R*,*E*)-2-(5-iodopent-4-en-2-yl)-4,4,5,5-tetramethyl-1,3,2-dioxaborolane (12b)

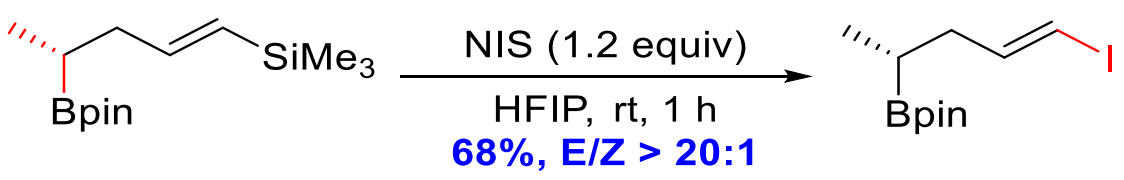

15 **12b**

According to a modified literature procedure.[14] To a flame-dried 25 mL round-bottom flask equipped with a magnetic stir bar in a glove box, was added (*E*)-vinylsilane **15** (268 mg, 1.00 mmol, 1.00 equiv) and hexafluoroisopropanol (10 mL). *N*-iodosuccinimide (270 mg, 1.20 mmol, 1.20 equiv) was added, the flask was sealed with a septum, and removed from glove box. The reaction mixture was stirred at room temperature for 1 h before being quenched with aqueous $Na_2S_2O_3$ (1 mL). HFIP was removed under reduced pressure and the resulting residue was diluted with DCM (5 mL) and water (5 mL). The organic layer was separated, and the aqueous phase was extracted with DCM (5 mL) three times. The combined organic layers were washed with brine (5 mL), dried over anhydrous $MgSO_4$, filtered, and concentrated under reduced pressure. The crude mixture was purified by flash column chromatography (hexane/EtOAc = 50:1) gave (*E*)-vinyl iodide **12b** (219 mg, 0.68 mmol, 68 %, >20:1 *E*/*Z*) as a colourless oil (Note).

Note: The *E*/*Z* ratio of the vinyl iodide could be calculated by GC-FID analysis.

R_f = 0.41 (20:1 hexane/EtOAc, Vanillin)

^{1}H NMR (500 MHz, $CDCl_3$) δ 6.50 (dt, J = 14.5, 7.4 Hz, 1H), 5.97 (dt, J = 14.3, 1.4 Hz, 1H), 2.22 – 2.13 (m, 1H), 2.09 – 2.00 (m, 1H), 1.23 (s, 12H), 1.17 – 1.08 (m, 1H), 0.96 (d, J = 7.4 Hz, 3H) ppm. **^{13}C NMR** (126 MHz, $CDCl_3$) δ 146.5, 83.3, 75.0, 39.6, 25.0, 24.9, 15.1 ppm.

Specific rotation $[\alpha]_D^{24}$ = −4 (c = 1.0, CH_2Cl_2).

HRMS (ESI) m/z calculated for $C_{10}H_{17}BIO_2$ $[M-Me]^+$, 307.0361, found: 307.0355.

2-((*S*)-1-(4-((*R*,*E*)-5-iodopent-4-en-2-yl)-2,6-dimethoxyphenyl)allyl)thiophene (11b)

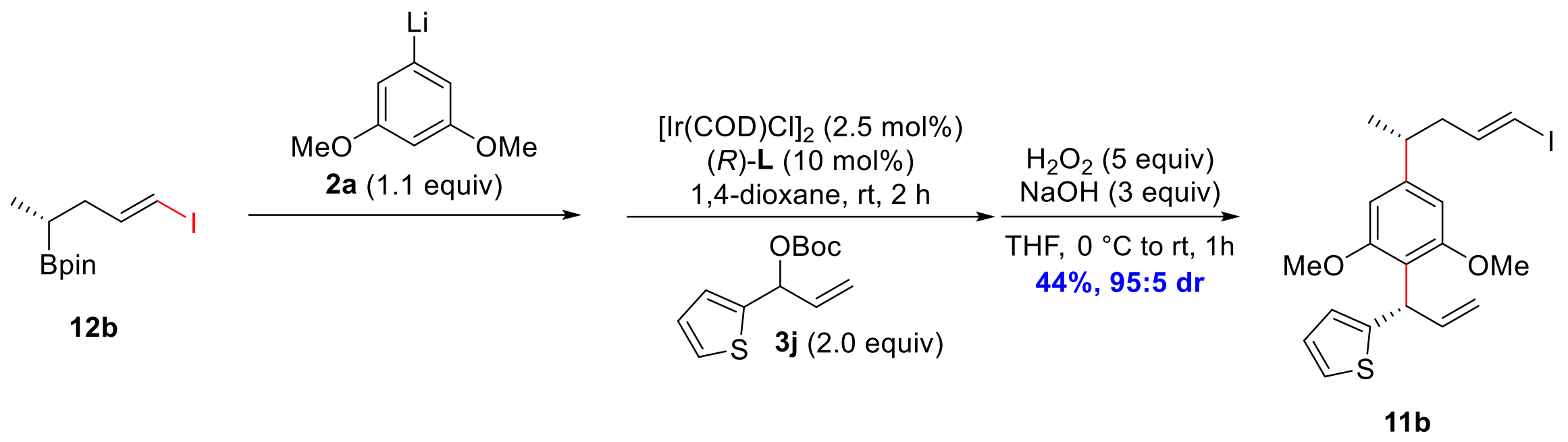

Prepared following **General Procedure A** using boronic ester **12b** (129 mg, 0.400 mmol, 1.00 equiv) and carbonate **3j** (192 mg, 0.800 mmol, 2.00 equiv) with (*R*)-**L**. Purification by flash column chromatography (hexane/toluene = 5:1) gave the title compound (80.7 mg, 44%, 95:5 dr) as a pale-yellow solid.

$\mathbf{R_f}$ = 0.63 (2:1 hexane/toluene, UV, Vanillin)

^{1}H NMR (500 MHz, $CDCl_3$) δ 7.07 (dd, *J* = 5.2, 1.3 Hz, 1H), 6.88 (dd, *J* = 5.1, 3.5 Hz, 1H), 6.78 – 6.73 (m, 1H), 6.61 (ddd, *J* = 17.1, 9.9, 8.0 Hz, 1H), 6.50 – 6.40 (m, 1H), 6.37 (s, 2H), 6.00 (d, *J* = 14.3 Hz, 1H), 5.51 (dd, *J* = 8.0, 1.3 Hz, 1H), 5.19 (dt, *J* = 17.1, 1.5 Hz, 1H), 5.09 (d, *J* = 9.8 Hz, 1H), 3.77 (s, 6H), 2.82 – 2.71 (m, 1H), 2.40 – 2.22 (m, 2H), 1.26 (d, *J* = 7.0 Hz, 3H) ppm. **^{13}C NMR** (126 MHz, $CDCl_3$) δ 158.0, 148.7, 146.6, 144.9, 139.9, 126.3, 123.7, 123.0, 118.0, 115.4, 103.6, 76.0, 56.0, 44.9, 40.7, 39.9, 21.1 ppm.

Specific rotation $[\alpha]_D^{24}$ = +41 (c = 2.0, CH_2Cl_2).

HRMS (ESI) m/z calculated for $C_{20}H_{24}IO_2S$ $[M+H]^+$, 455.0536, found: 455.0550.

The diastereomeric ratio (dr) was determined after Suzuki coupling of the vinyl iodide with (4-hydroxyphenyl)boronic acid according to **General Procedure C**. **HPLC conditions**: Chiral column IB, hexane: isopropanol = 98:2, flow rate = 1.0 mL/min, wavelength = 254 nm, t_R = 57.6 min for major isomer, t_R = 70.9 min for minor isomer.

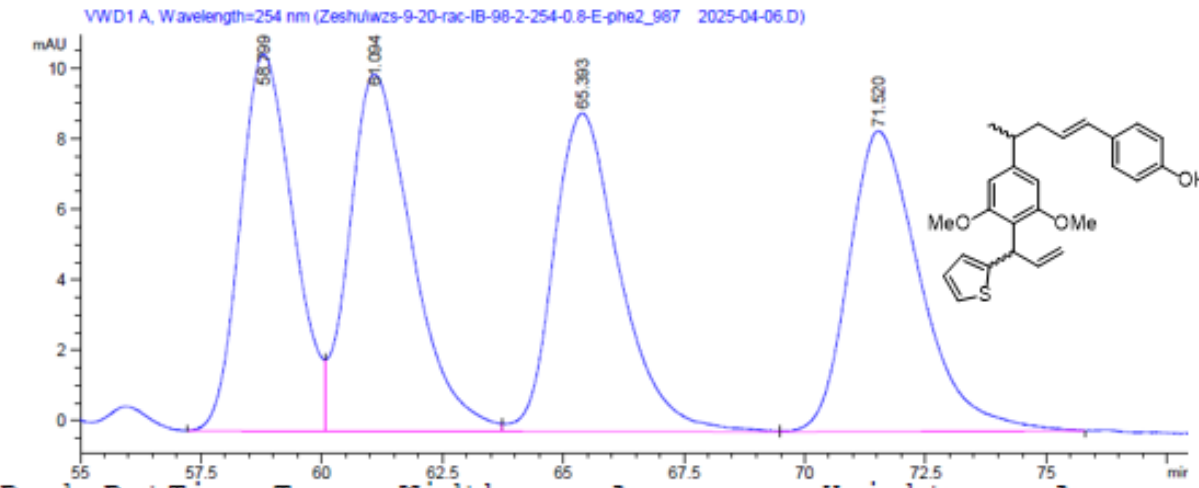

Peak #	RetTime [min]	Type	Width [min]	Area [mAU*s]	Height [mAU]	Area %
1	58.799	BV	1.1671	819.95593	10.70813	23.6084
2	61.094	VV	1.2612	918.04333	10.12939	26.4326
3	65.393	VB	1.3653	859.12158	9.03724	24.7361
4	71.520	BB	1.3376	876.03357	8.53234	25.2230

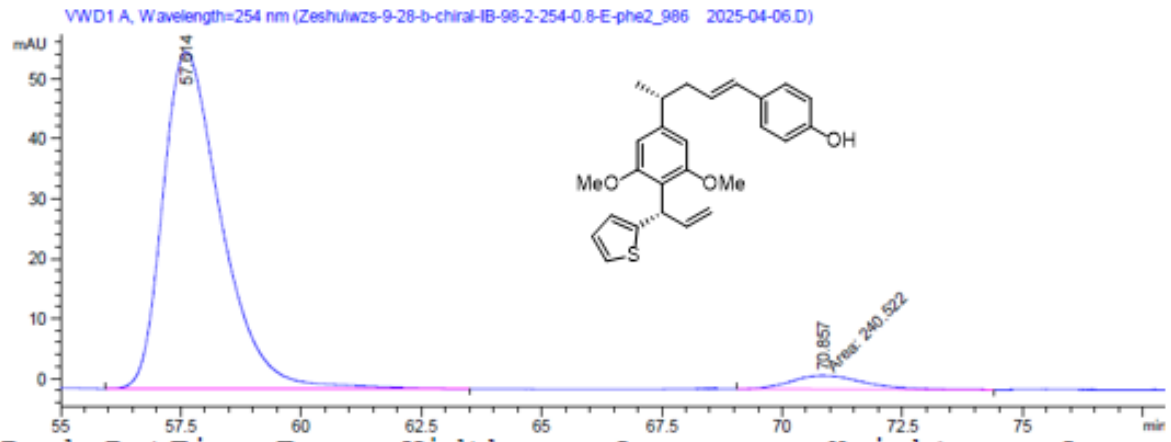

Peak #	RetTime [min]	Type	Width [min]	Area [mAU*s]	Height [mAU]	Area %
1	57.614	BB	1.2834	4745.45068	56.12811	95.1760
2	70.857	MM	1.7561	240.52209	2.28277	4.8240

3.3. Optimization of the Suzuki Cyclodimerization Reaction

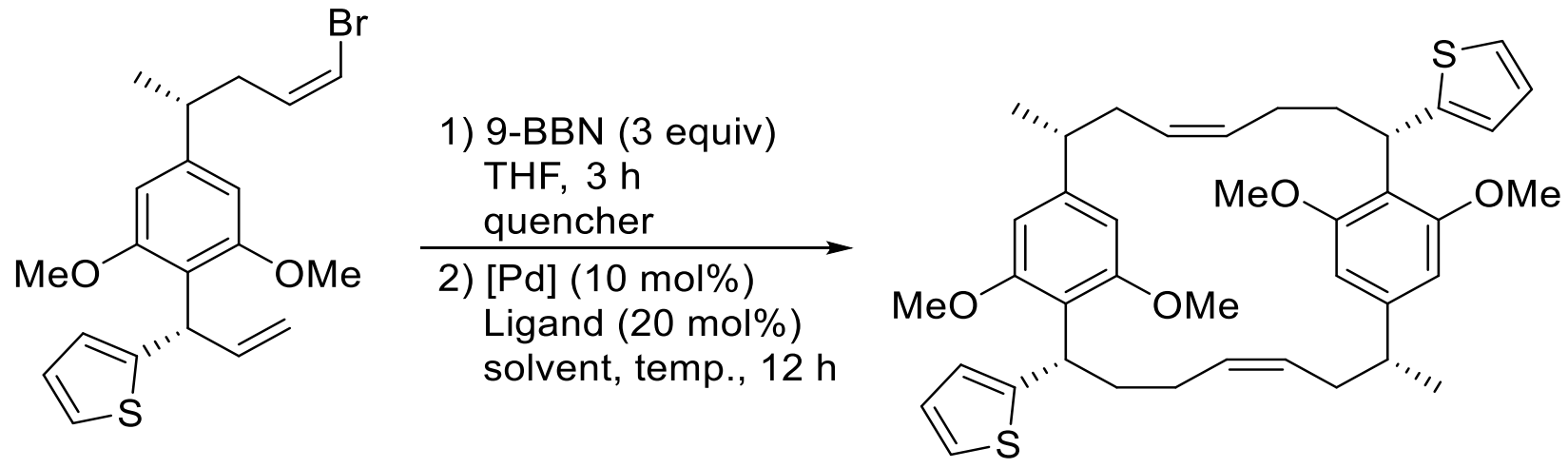

entry	quencher	[Pd]/ligand	conditions	yield (%)
1[a]	H_2O (55 equiv)	Pd(dppf)Cl_2/$AsPh_3$	THF/DMF, 50 ºC	19
2	NaOH (3 N, 3 equiv)	Pd(dppf)Cl_2/$AsPh_3$	THF/DMF, rt	24
3[b]	NaOH (3 N, 3 equiv)	Pd(PPh_3)$_4$	THF, rt	16
4	NaOH (3 N, 3 equiv)	Pd(PPh_3)$_4$	THF, 60 ºC	39
5	NaOH (3 N, 3 equiv)	Pd(PPh_3)$_4$	THF, 80 ºC	35
6[c]	NaOH (3 N, 3 equiv)	Pd(OAc)$_2$/SPhos	THF, rt	43
7[c,d]	NaOH (3 N, 3 equiv)	Pd(OAc)$_2$/SPhos	THF, rt	46
8[c,d]	NaOH (3 N, 3 equiv)	Pd(OAc)$_2$/RuPhos	THF, rt	43

Table S4: Reaction optimization with *Z*-vinyl bromide 11c

[a]Cs_2CO_3 (2 equiv) was used as base; [b]unreacted hydroboration product was observed upon oxidation; [c]5 mol% of [Pd] and 10 mol% of ligand were used; [d]slow addition of R-BBN to catalyst solution was performed over 30 min.

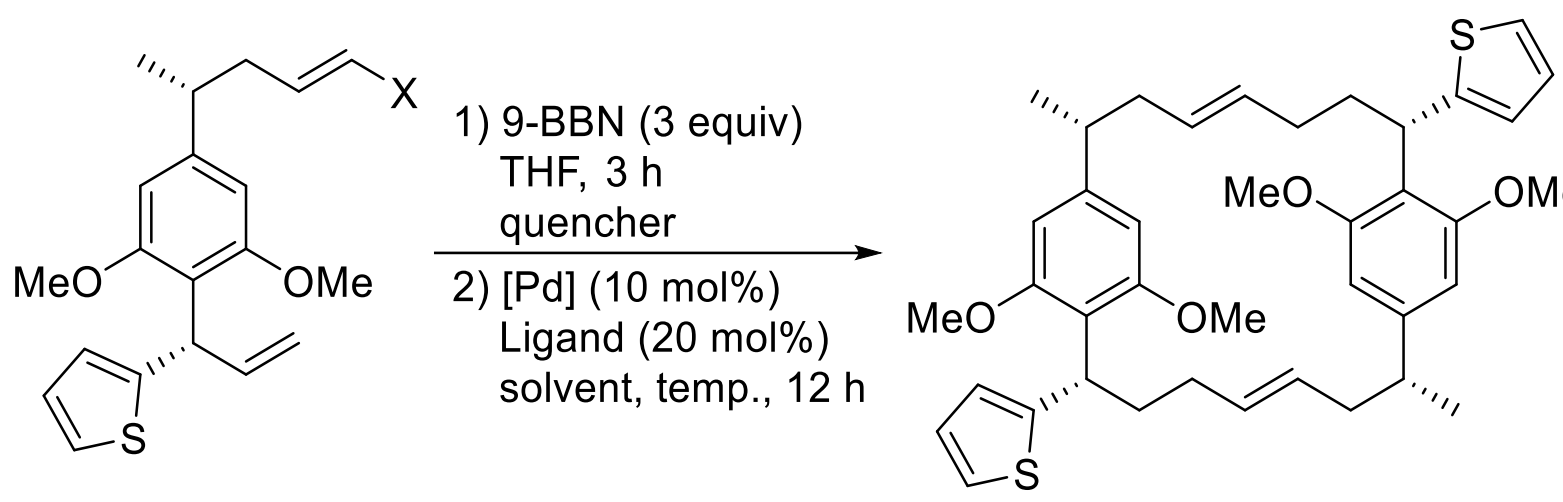

entry	X	quencher	[Pd]/ligand	conditions	yield (%)
1[a]	Br	H_2O (55 equiv)	Pd(dppf)Cl_2/$AsPh_3$	THF/DMF, rt	<5
2[a]	Br	H_2O (55 equiv)	Pd(dppf)Cl_2/$AsPh_3$	THF/DMF, 50 ºC	<5
3[a]	Br	H_2O (55 equiv)	Pd(dppf)Cl_2/$AsPh_3$	THF/DMF, 70 ºC	<5
4[b]	Br	NaOH (3 N, 3 equiv)	Pd(PPh_3)$_4$	THF, 60 ºC	5
5[b]	Br	NaOH (3 N, 3 equiv)	Pd(OAc)$_2$/SPhos	THF, rt	18
6[a]	I	H_2O (55 equiv)	Pd(dppf)Cl_2/$AsPh_3$	THF/DMF, rt	<5
7[b]	I	NaOH (3 N, 3 equiv)	Pd(PPh_3)$_4$	THF, 60 ºC	9
8[b]	I	NaOH (3 N, 3 equiv)	Pd(OAc)$_2$/SPhos	THF, rt	16

Table S5: Reaction optimization with *E*-vinyl halides (11a/b)

[a]Cs_2CO_3 (2 equiv) was used as base; [b]5 mol% of [Pd] catalyst and 10 mol% of ligand were used.

3.4. Optimization of the Demethylation Reaction

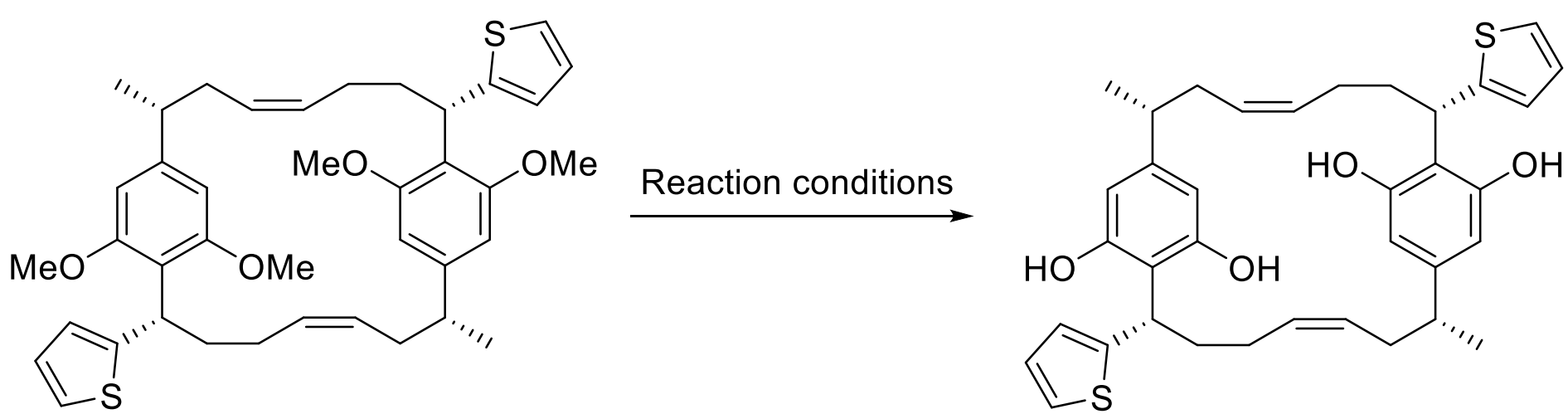

entry	reaction conditions	results
1	PhSH (300 equiv), K_2CO_3 (6 equiv), NMP, 215 °C, 8 h	messy
2	$Et_2NCH_2CH_2SH \cdot HCl$ (100 equiv), NMP, 200 °C, 16 h	messy
3	BBr_3 (10 equiv), –78 °C to rt, 2 h	decomposition
4	BCl_3 (10 equiv), –78 °C to rt, 16 h	decomposition

Table S6: Unsuccessful reaction optimization of the demethylation reaction

3.5. Attempted Cross Metathesis/Ring-Closing Metathesis Cyclodimerization

3.5.1. Synthesis of compound 8

4,4,5,5-Tetramethyl-2-(pent-4-en-1-yl)-1,3,2-dioxaborolane (17)

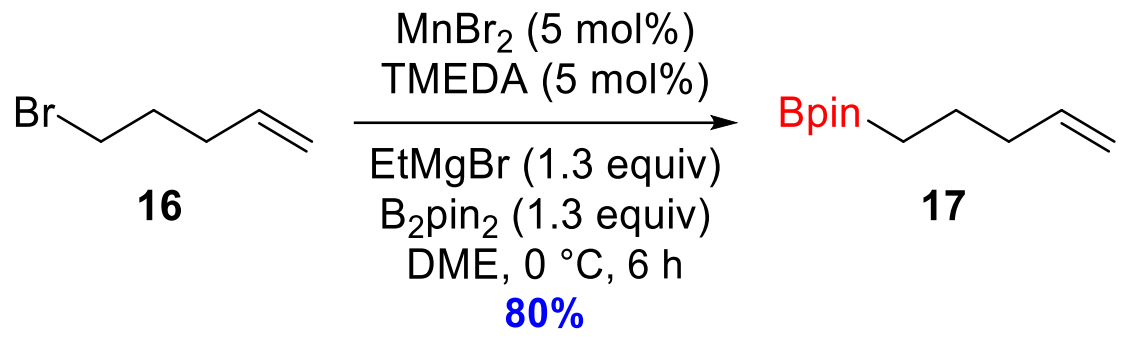

According to a modified literature procedure.[15] A 25 mL flame-dried round-bottom flask was charged with $MnBr_2$ (5 mol%, 161 mg) and bis(pinacolato)diboron (4.95 g, 19.5 mmol, 1.30 equiv) under N_2. The flask was back-filled with N_2, and dimethoxyethane (30 mL) was added, followed by TMEDA (0.11 mL, 0.75 mmol, 5.0 mol%). The mixture was cooled in an ice bath, and ethylmagnesium bromide (6.50 mL, 19.5 mmol, 1.30 equiv, 3 M in Et_2O) was added dropwise. After stirring for 2 minutes, 5-bromopent-1-ene **16** (2.24 g, 15.0 mmol, 1.00 equiv) was added to the reaction mixture in one portion. The reaction mixture was stirred at 0 ºC for 6 hours then quenched with 1 N aqueous HCl (25 mL). The organic layer was separated, and the aqueous phase was extracted with Et_2O (50 mL) three times. The combined organic layers were washed with brine (50 mL), dried over anhydrous $MgSO_4$, filtered, and concentrated under reduced pressure. The crude mixture was purified by flash column chromatography (hexane/EtOAc = 50:1) gave boronic ester **17** (2.35 g, 12.0 mmol, 80%) as a colourless oil (Note).

Note: If the product is not pure after chromatography, it can be purified further by distillation.

R_f = 0.50 (20:1 hexane/EtOAc, Cerium Ammonium Molybdate)

^{1}H NMR (500 MHz, $CDCl_3$) δ 5.79 (ddt, *J* = 17.0, 10.2, 6.7 Hz, 1H), 4.98 (dq, *J* = 17.1, 1.7 Hz, 1H), 4.95 – 4.90 (m, 1H), 2.04 (dt, *J* = 8.0, 6.8 Hz, 2H), 1.50 (p, *J* = 7.7 Hz, 2H), 1.24 (s, 12H), 0.78 (t, *J* = 7.9 Hz, 2H) ppm. **^{13}C NMR** (126 MHz, $CDCl_3$) δ 139.1, 114.6, 83.0, 83.0, 36.5, 25.0, 23.5, 7.8 ppm.

HRMS (ESI) m/z calculated for $C_{10}H_{18}BO_2$ $[M{-}Me]^+$, 181.1394, found: 181.1391.

(*R*)-2-(hept-6-en-2-yl)-4,4,5,5-tetramethyl-1,3,2-dioxaborolane (9)

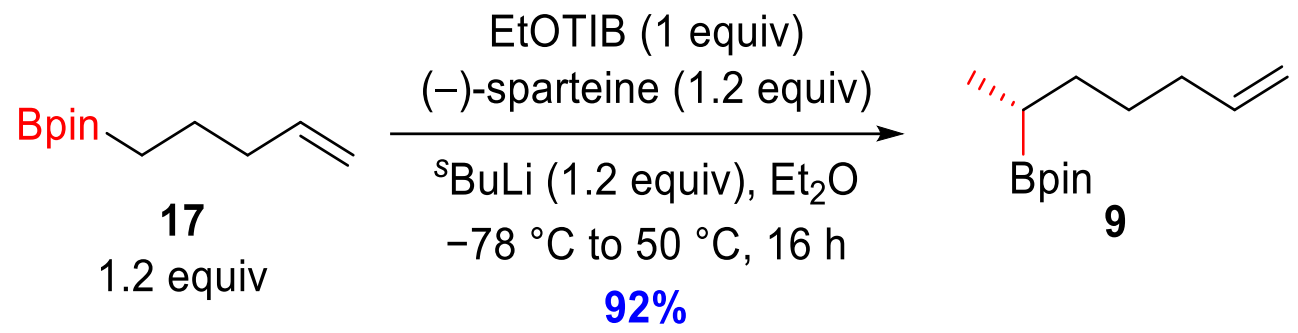

According to a modified literature procedure.[7] Ethyl 2,4,6-triisopropylbenzoate (857 mg, 3.10 mmol, 1.00 equiv) and (−)-sparteine (0.850 mL, 3.72 mmol, 1.20 equiv) were added to a 50 mL flame-dried Schlenk tube under N_2. Anhydrous Et_2O (18 mL) was added and the resulting solution was cooled to –78 °C (acetone/dry ice). ^{s}BuLi (2.68 mL, 3.72 mmol, 1.20 equiv, 1.39 M in hexane) was added dropwise and the resulting reaction mixture was stirred at –78 °C for 5 h. A solution of boronic ester **17** (729 mg, 3.72 mmol, 1.20 equiv) in anhydrous Et_2O (3 mL) was added dropwise, and the vessel was rinsed with additional Et_2O (1 mL). The resulting solution was stirred at –78 °C for 1 h, warmed to room temperature and then heated at 50 °C (oil bath) for 16 h. The reaction mixture was cooled to room temperature and quenched with a 2 N aqueous HCl (18 mL). The layers were separated and the aqueous phase was washed with Et_2O (20 mL) three times. The combined organic layers were washed with 2 N HCl (18 mL), and the combined aqueous phases were retained for (−)-sparteine recovery. The organic phase was washed with water (20 mL), brine (20 mL), dried over $MgSO_4$, filtered, and concentrated under reduced pressure. The crude mixture was purified by flash column chromatography (hexane/toluene = 5:1) gave secondary boronic ester **9** (642 mg, 2.86 mmol, 92%) as a colourless oil (Note).

Note: Residual ethyl 2,4,6-triisopropylbenzoate (EtOTIB) is commonly observed and is often inseparable by flash chromatography with hexane/EtOAc. However, using hexane/toluene as the eluent allowed for the efficient removal of EtOTIB, typically after 2–3 rounds of chromatography.

R_f = 0.59 (2:1 hexane/toluene, Vanillin)

^{1}H NMR (400 MHz, $CDCl_3$) δ 5.81 (ddt, *J* = 16.9, 10.2, 6.6 Hz, 1H), 4.99 (dq, *J* = 17.1, 1.8 Hz, 1H), 4.95 – 4.89 (m, 1H), 2.08 – 1.98 (m, 2H), 1.53 – 1.28 (m, 4H), 1.23 (s, 12H), 0.96 (d, *J* = 5.5 Hz, 4H) ppm. **^{13}C NMR** (126 MHz, $CDCl_3$) δ 139.4, 114.2, 82.9, 34.2, 32.9, 28.4, 24.9, 24.9, 15.6 ppm.

Specific rotation $[\alpha]_D^{22}$ = −5 (c = 3.8, CH_2Cl_2).

HRMS (ESI) m/z calculated for $C_{12}H_{22}BO_2$ [M−Me]$^+$, 209.1707, found: 209.1705.

2-((*S*)-1-(4-((*R*)-hept-6-en-2-yl)-2,6-dimethoxyphenyl)allyl)thiophene (8)

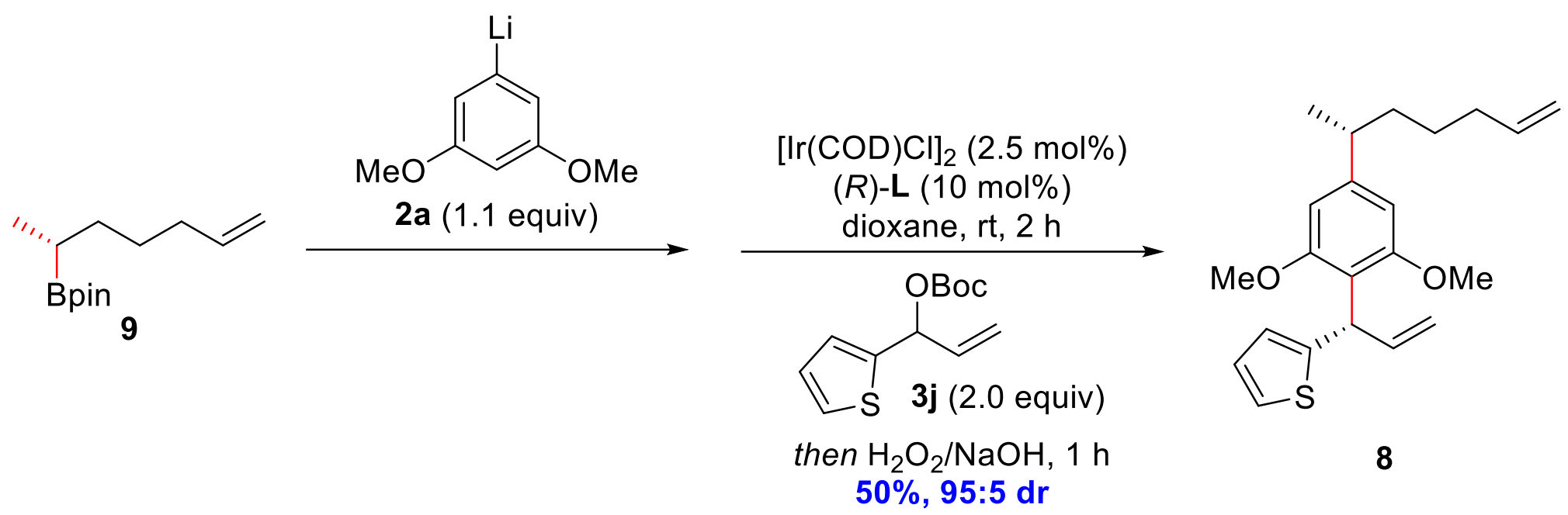

Prepared following **General Procedure A** using boronic ester **9** (134 mg, 0.600 mmol, 1.00 equiv) and carbonate **3a** (288 mg, 1.200 mmol, 2.00 equiv) with (*R*)-**L**. Purification by flash column chromatography (hexane/toluene = 5:1) gave the title compound (107 mg, 50%, 95:5 dr) as a colourless oil.

$\mathbf{R}_f$ = 0.58 (2:1 hexane/toluene, Vanillin)

^{1}H NMR (500 MHz, $CDCl_3$) δ 7.07 (dd, *J* = 5.1, 1.2 Hz, 1H), 6.88 (dd, *J* = 5.1, 3.5 Hz, 1H), 6.78 – 6.73 (m, 1H), 6.61 (ddd, *J* = 17.1, 9.9, 8.0 Hz, 1H), 6.38 (s, 2H), 5.85 – 5.73 (m, 1H), 5.51 (dd, *J* = 8.1, 1.2 Hz, 1H), 5.18 (ddd, *J* = 17.1, 1.8, 1.1 Hz, 1H), 5.08 (ddd, *J* = 9.9, 1.9, 0.8 Hz, 1H), 4.99 (dq, *J* = 17.1, 1.7 Hz, 1H), 4.96 – 4.89 (m, 1H), 3.76 (s, 6H), 2.63 (q, *J* = 7.0 Hz, 1H), 2.10 – 1.99 (m, 2H), 1.67 – 1.51 (m, 2H), 1.46 – 1.26 (m, 2H), 1.24 (d, *J* = 6.9 Hz, 3H) ppm. **^{13}C NMR** (126 MHz, $CDCl_3$) δ 157.9, 148.9, 148.4, 140.0, 139.1, 126.3, 123.7, 123.0, 117.5, 115.3, 114.5, 103.6, 55.9, 40.7, 40.5, 38.0, 34.0, 27.2, 22.3 ppm.

Specific rotation $[\alpha]_D^{24}$ = −10 (c = 1.0, CH_2Cl_2).

HRMS (ESI) m/z calculated for $C_{22}H_{29}O_2S$ [M+H]$^+$, 357.1883, found: 357.1874.

The diastereomeric ratio (dr) was determined using the corresponding alcohol which was prepared following **General Procedure B**. **HPLC conditions**: Chiral column IA, hexane: isopropanol = 90:10, flow rate = 1.0 mL/min, wavelength = 254 nm, t_R = 14.6 min for major isomer, t_R = 15.7 min for minor isomer.

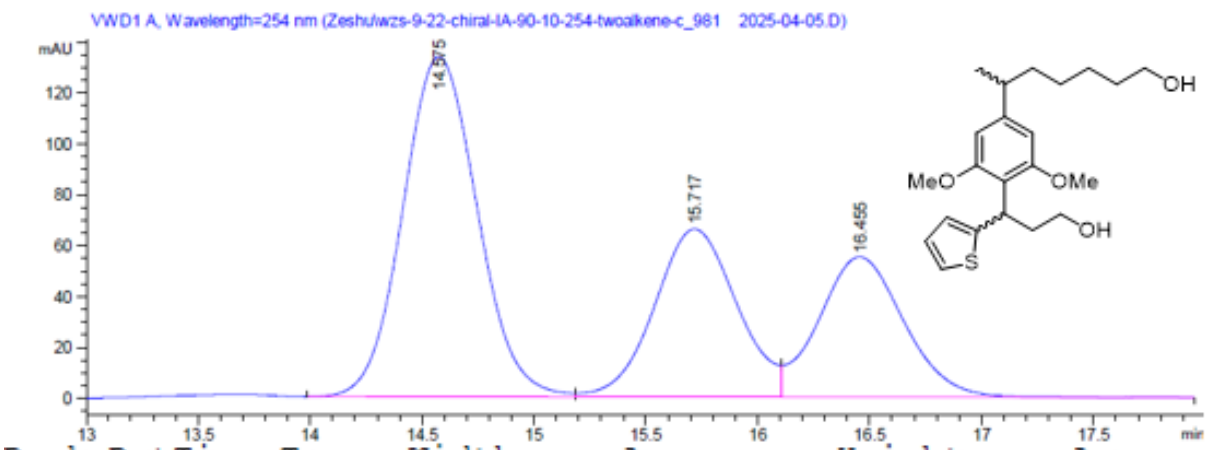

Peak #	RetTime [min]	Type	Width [min]	Area [mAU*s]	Height [mAU]	Area %
1	14.575	BV	0.3634	3094.56030	133.60162	49.5674
2	15.717	VV	0.3941	1686.30029	66.01641	27.0105
3	16.455	VBA	0.4086	1462.27124	55.10407	23.4221

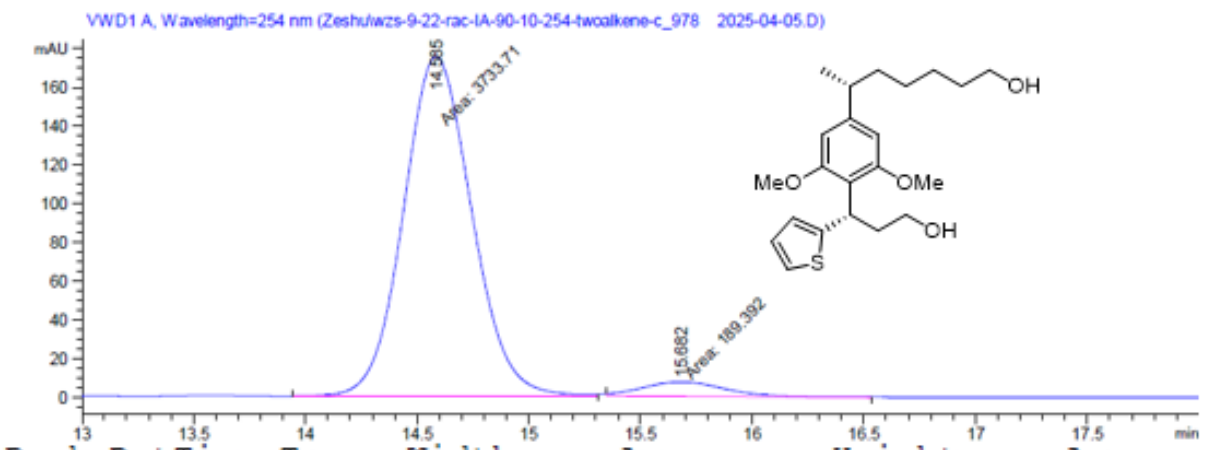

Peak #	RetTime [min]	Type	Width [min]	Area [mAU*s]	Height [mAU]	Area %
1	14.585	MM	0.3550	3733.70996	175.26733	95.1724
2	15.682	MM	0.4236	189.39229	7.45119	4.8276

3.5.2. Cross metathesis/ring-closing metathesis cyclodimerization optimization

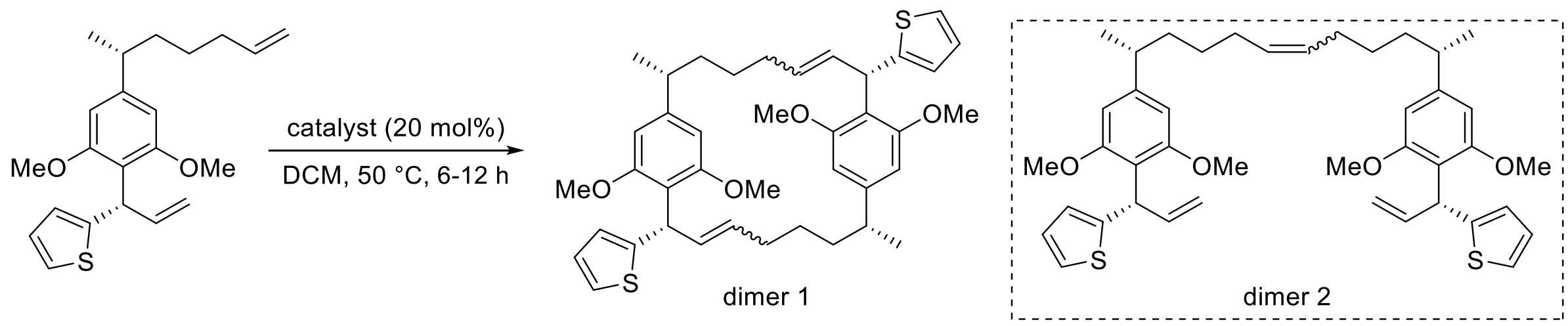

entry	catalyst	yield (%)	
		dimer 1	dimer 2
1	Grubbs I	N.D.	<5%
2	Grubbs II	N.D.	N.D.
3	Grubbs III	N.D.	<5%
4	Hoveyda-Grubbs II	N.D.	<5%
5[a]	Hoveyda-Grubbs II	N.D.	<5%
6[b]	Hoveyda-Grubbs II	N.D.	<5%
7[c]	Hoveyda-Grubbs II	N.D.	N.D.

Table S7: Cross metathesis/ring-closing metathesis cyclodimerization optimization

[a]40 mol% catalyst was used; [b]benzene was used as solvent instead of DCM; [c]toluene was used as solvent instead of DCM at 80 ºC.

3.6. Comparison of Spectroscopic Data for Synthetic and Isolated Merocyclophane A

3.6.1. ^{1}H NMR of merocyclophane A from isolation paper

S3. ^{1}H NMR spectrum (600 MHz, MeOH-d_4) of **1**

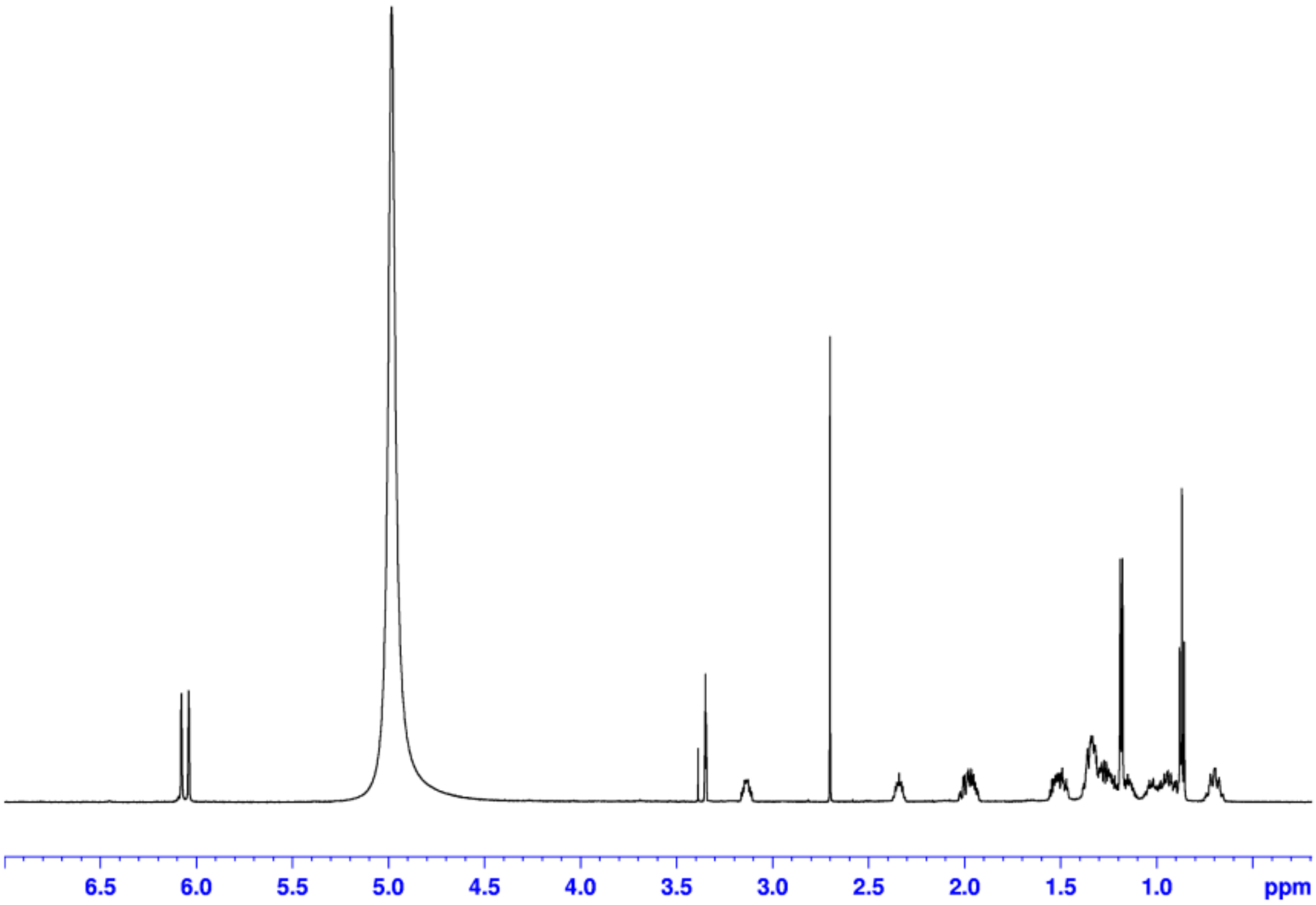

Taken from original isolation paper (*Phytochemistry* **2012**, *79*, 109–115)[13b]

3.6.2. ^{1}H and ^{13}C NMR of previously synthesised merocyclophane A

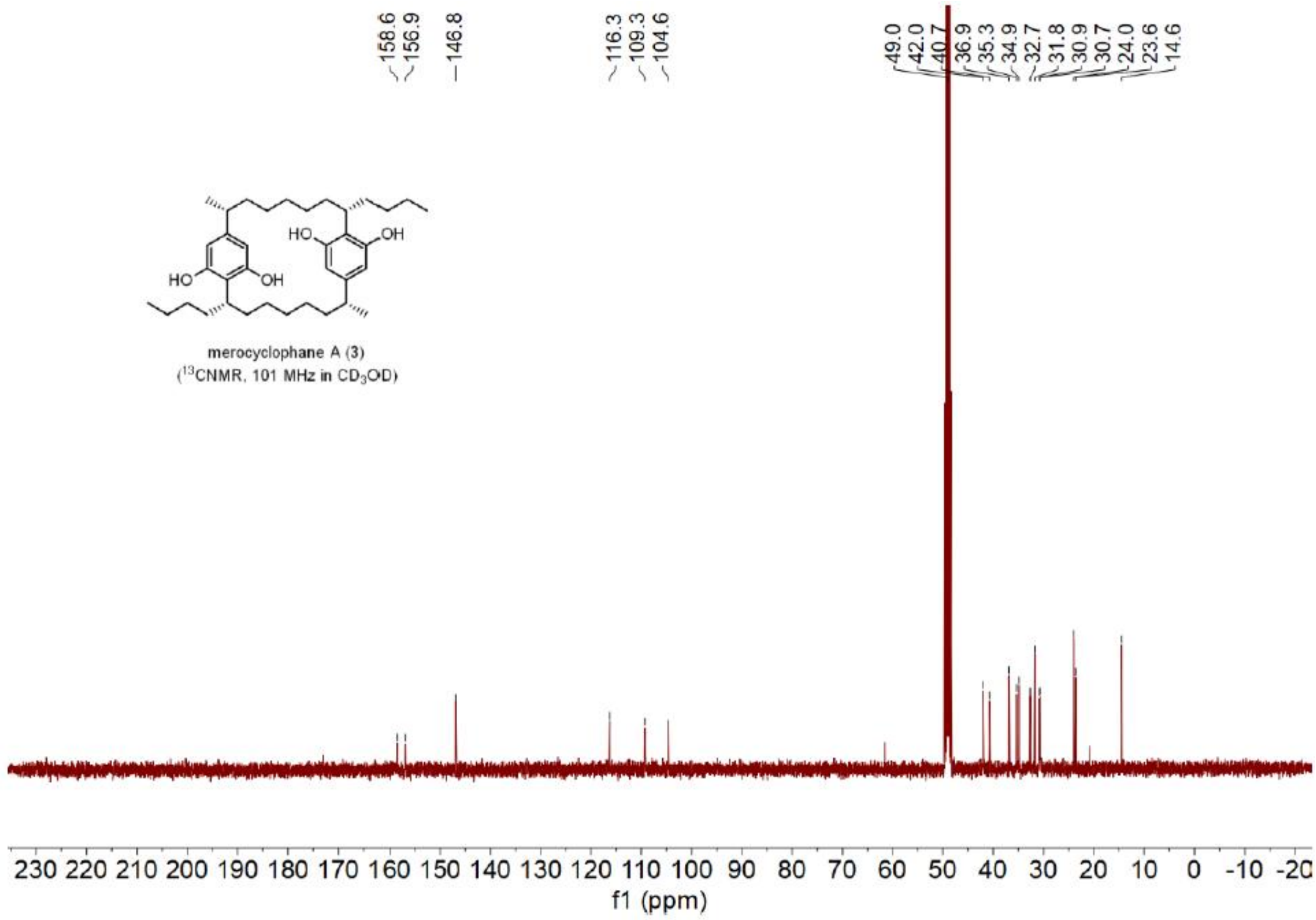

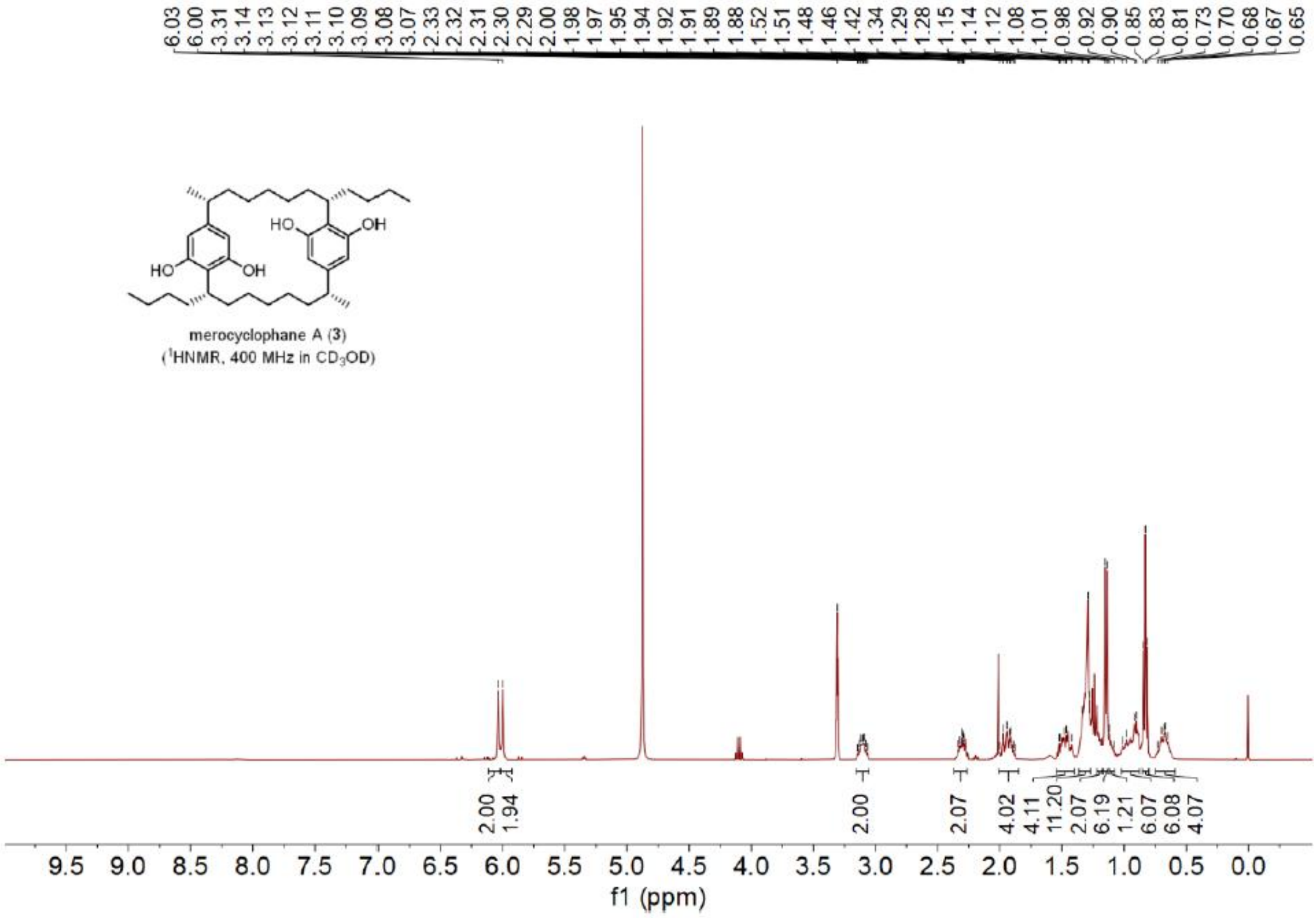

Taken from the report of Xiang and coworkers[13a]

3.6.3. ^{1}H and ^{13}C NMR of merocyclophane A synthesised in this study

^{1}H NMR (500 MHz, CD_3OD) of **6** (merocyclophane A)

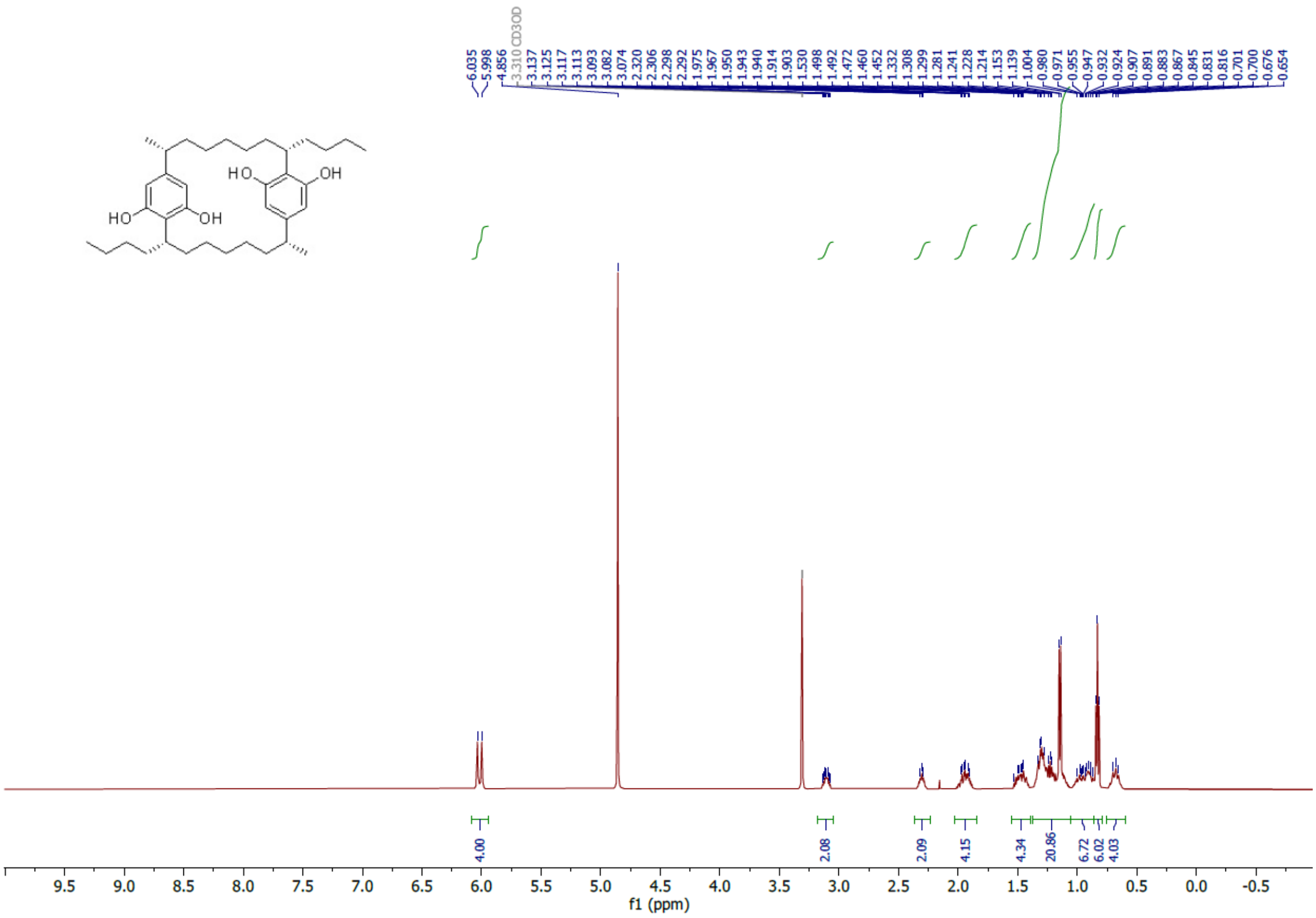

^{13}C NMR (126 MHz, CD_3OD) of **6**

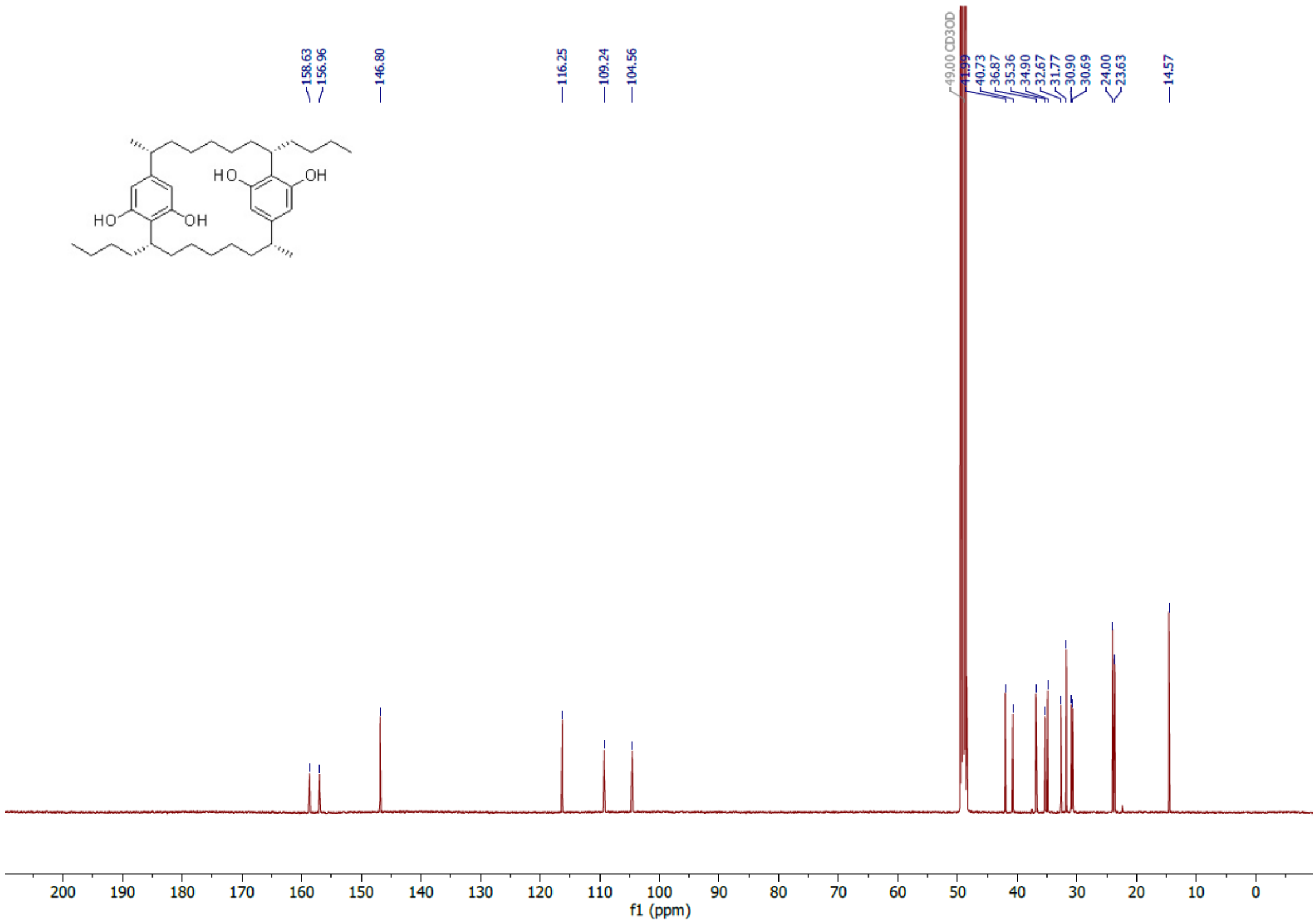

4. X-ray Crystallography

4.1. Crystallographic Data for Compound 5b (CCDC 2539493)

Single colourless block-shaped crystals of **5b** were chosen from the sample as supplied. A suitable crystal 0.32×0.27×0.27 mm^3 was selected and mounted on a Bruker D8 Venture. The crystal was kept at a steady T = 100(2) K during data collection. The structure was solved with the ShelXS[16] structure solution program using the direct methods solution method and by using Olex2 1.5[17] as the graphical interface. The model was refined with version 2019/3 of ShelXL 2019/3[18] using full matrix least squares minimisation on $|\boldsymbol{F}|^2$ minimisation. X-ray diffraction data were collected using a Bruker D8 Venture equipped with an Oxford Cryosystems low-temperature device, operating at T = 100(2) K. The structure was solved in the space group $P2_1$ (# 4) by direct methods using the ShelXS[16] structure solution program and refined by full matrix least squares minimisation on $|\boldsymbol{F}|^2$ using version 2019/3 of ShelXL 2019/3.[18] All non-hydrogen atoms were refined anisotropically. Hydrogen atom positions were calculated geometrically and refined using the riding model.

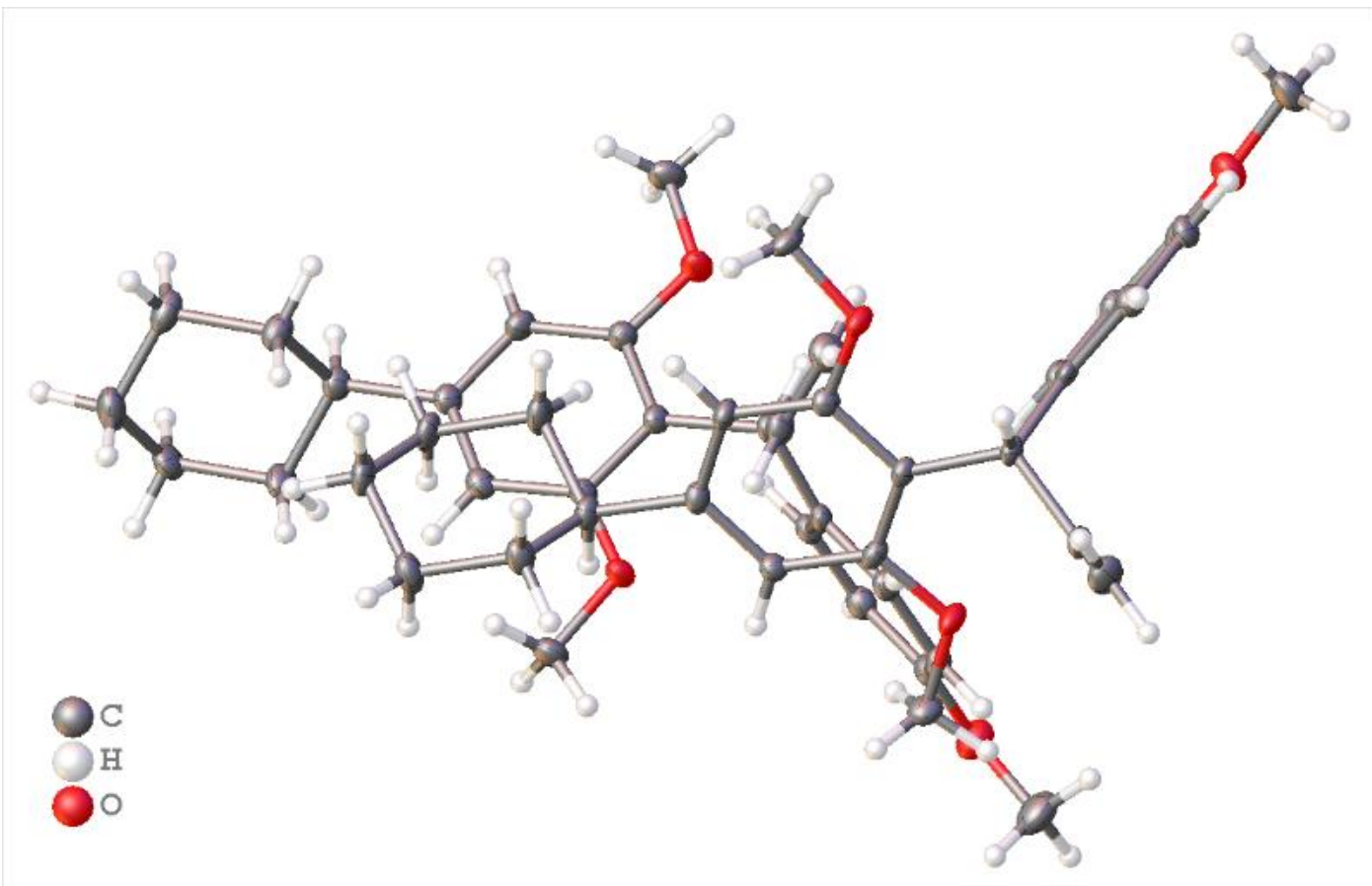

Figure S1: Crystal structure of 5b

4.2. Crystallographic Data for Compound 10 (CCDC 2539492)

Single colourless irregular plate-shaped crystals of **10** were chosen from the sample as supplied. A suitable crystal 0.330×0.110×0.070 mm^3 was selected and mounted on a MITIGEN holder in oil on a Rigaku FRE+ diffractometer with HF Varimax confocal mirrors, a UG2 goniometer and HyPix 6000HE detector. The crystal was kept at a steady T = 100(2) K during

data collection. The structure was solved with the ShelXT 2018/2[19] structure solution program using the using dual methods solution method and by using Olex2 1.5[17] as the graphical interface. The model was refined with version 2018/3 of ShelXL 2018/3[20] using full matrix least squares minimisation on $\boldsymbol{F}^2$ minimisation. X-ray diffraction data were collected using a Rigaku FRE+ diffractometer with HF Varimax confocal mirrors, a UG2 goniometer and HyPix 6000HE detector equipped with an Oxford Cryosystems low-temperature device, operating at T = 100(2) K. The structure was solved in the space group P1 (# 1) by using dual methods using the ShelXT 2018/2[19] structure solution program and refined by full matrix least squares minimisation on $\boldsymbol{F}^2$ using version 2018/3 of ShelXL 2018/3.[18] All non-hydrogen atoms were refined anisotropically. Hydrogen atom positions were calculated geometrically and refined using the riding model.

All four pendant thiophene rings were found to be rotationally disordered, and have been modelled over two sites with complementary occupancies. At least one orientation of each has been modelled using the model of thiophene from the fragment database. In all cases, the two orientations were fixed to have equal bonds and angles. Pairs of atoms from different orientations that are close to each other have been constrained to have equal U_{ij} values using EADP commands, and the pivot carbon atoms of both groups have been fixed at the same coordinates using EXYZ commands.

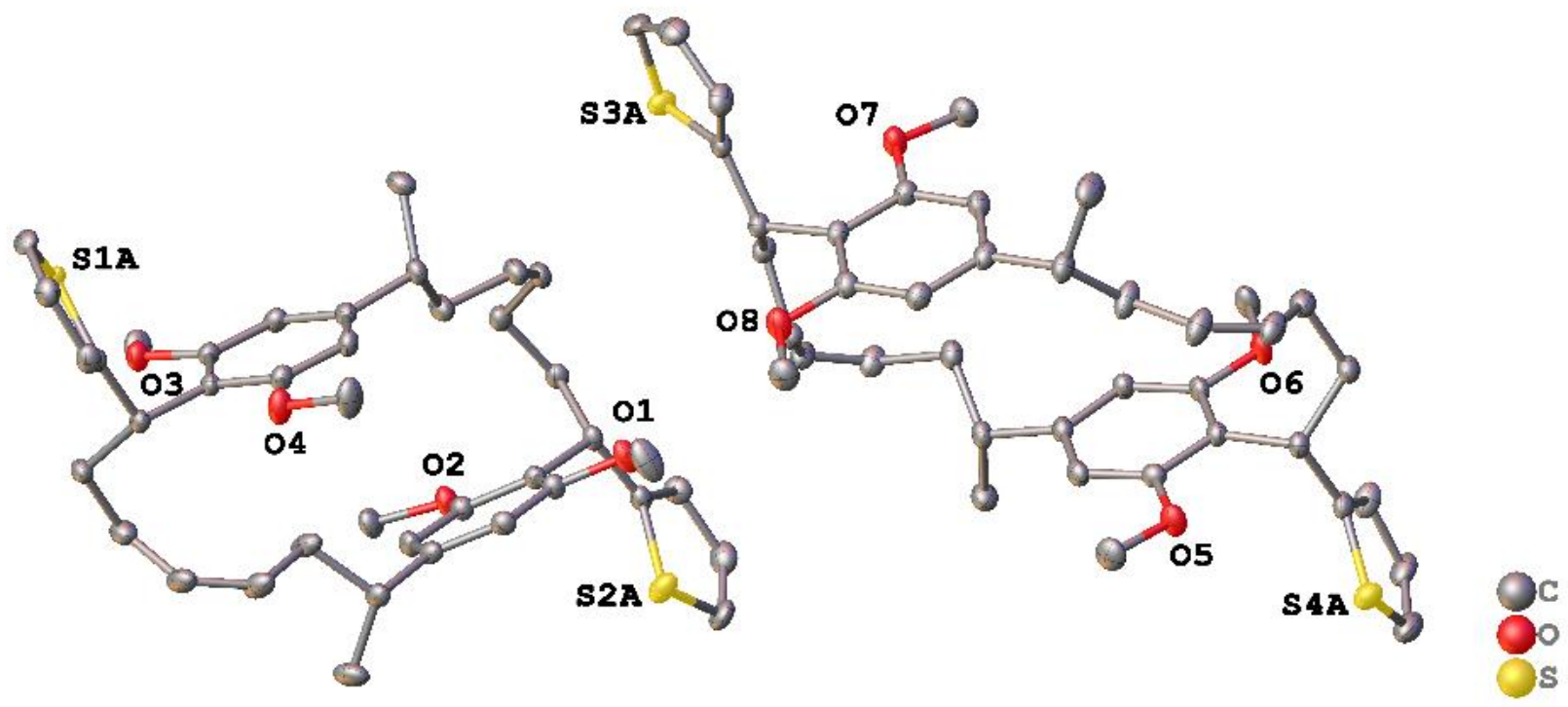

Figure S2: Crystal structure of 10 with hydrogens omitted for clarity

Table S8: Crystal data and structure refinement for 5b and 10[21, 22]

**Compound**	**5b**	**Compound**	**10**

Formula	$C_{24}H_{30}O_3$	Formula	$C_{40}H_{48}O_4S_2$
$D_{calc.}$/ g cm^{-3}	1.188	$D_{calc.}$/ g cm^{-3}	1.218
m/mm^{-1}	0.603	*m*/mm^{-1}	0.188
Formula Weight	366.48	Formula Weight	656.90
Colour	colourless	Colour	colourless
Shape	block-shaped	Shape	irregular plate-shaped
Size/mm	0.32×0.27×0.27	Size/mm^3	0.330×0.110×0.070
T/K	100(2)	*T*/K	100(2)
Crystal System	monoclinic	Crystal System	triclinic
Flack Parameter	0.06(18)	Flack Parameter	-0.011(11)
Space Group	$P2_1$	Hooft Parameter	-0.010(9)
a/Å	12.5742(3)	Space Group	*P*1
b/Å	13.5932(3)	*a*/Å	8.90340(10)
c/Å	13.3266(3)	*b*/Å	9.66340(10)
a/°	90	*c*/Å	22.1862(2)
b/°	115.9356(4)	*a*/°	89.3640(10)
g/°	90	*b*/°	89.5440(10)
V/Å^3	2048.42(8)	*g*/°	69.7960(10)
Z	4	V/Å^3	1791.26(3)
Z'	2	*Z*	2
Wavelength/Å	1.54178	*Z'*	2
Radiation type	CuK$_a$	Wavelength/Å	0.71073
Q_{min}/°	3.688	Radiation type	Mo K$_a$
Q_{max}/°	79.029	Q_{min}/°	1.836

Index range h	$-15 \leq h \leq 15$	$Q_{max}/^{\circ}$	30.507
Index range k	$-17 \leq k \leq 14$	Measured Refl's.	174380
Index range l	$-16 \leq l \leq 16$	Indep't Refl's	21508
Measured Refl's.	37218	Refl's I≥2 *s*(I)	19966
Indep't Refl's	8132	R_{int}	0.0379
Refl's I≥2*s*(I)	8089	Parameters	905
R_{int}	0.0391	Restraints	561
Parameters	493	Largest Peak	0.362
Restraints	1	Deepest Hole	-0.224
Largest Peak/eÅ^3	0.686	GooF	1.045
Deepest Hole/eÅ^3	-0.658	wR_2 (all data)	0.0867
GooF	1.182	wR_2	0.0849
R_1 (I≥2*s*(I) / all)	0.0790 / 0.0791	R_1 (all data)	0.0383
wR_2 (I≥2*s*(I) / all)	0.2298 / 0.2305	R_1	0.0342

5. NMR Spectra

^{1}H NMR (400 MHz, $CDCl_3$) of **4a-minor**

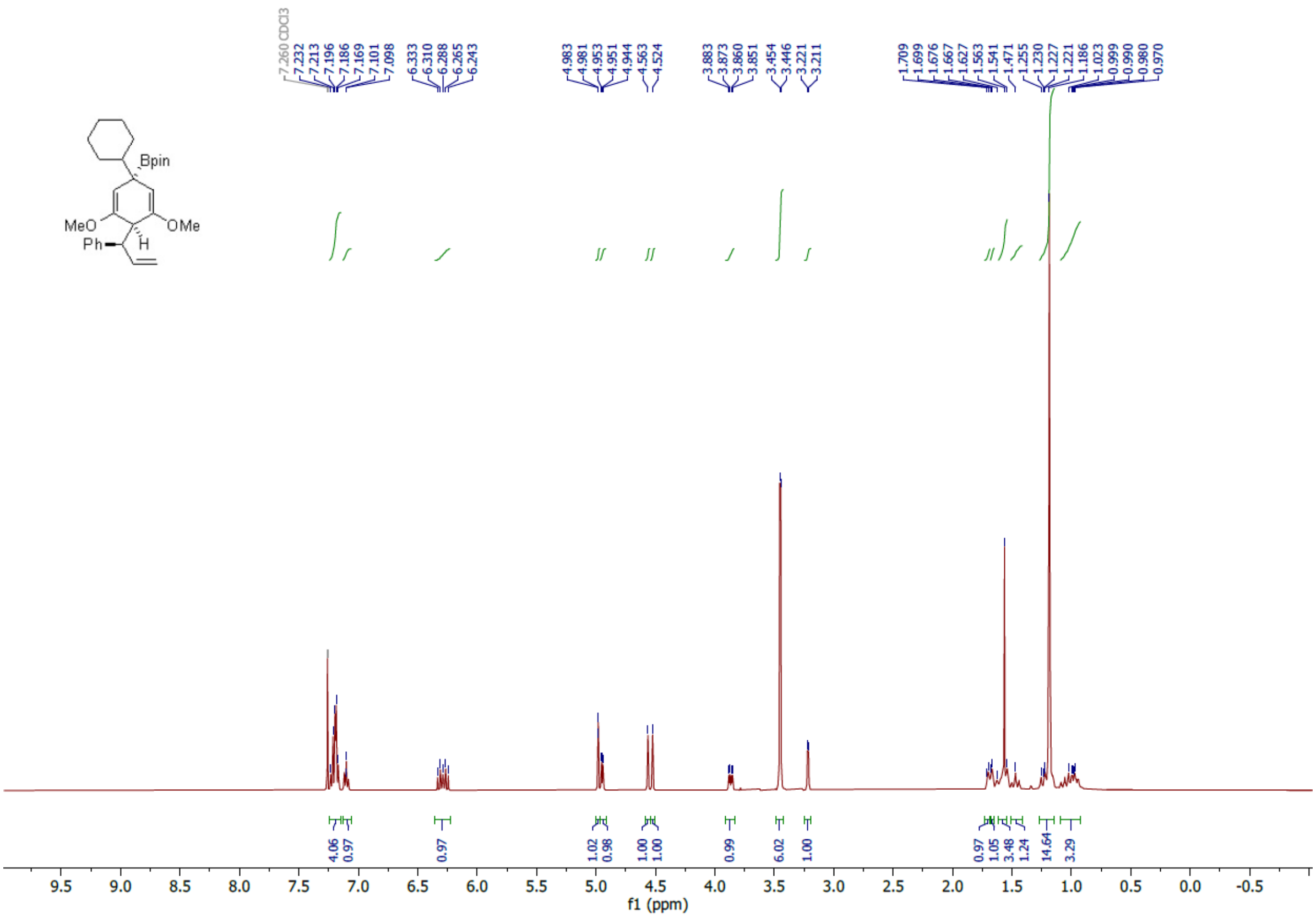

^{13}C NMR (126 MHz, $CDCl_3$) of **4a-minor**

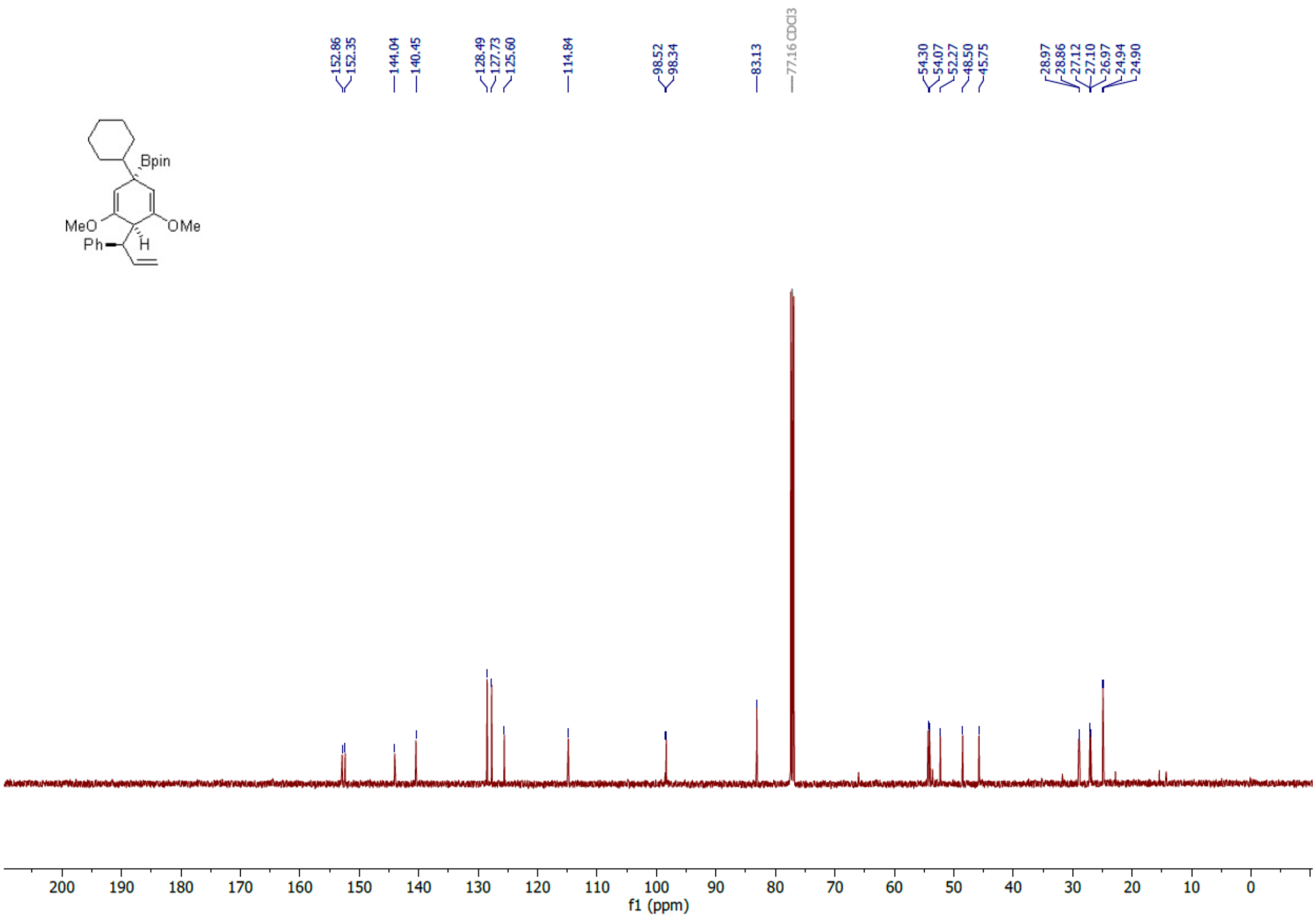

^{1}H NMR (400 MHz, $CDCl_3$) of **4a-major**

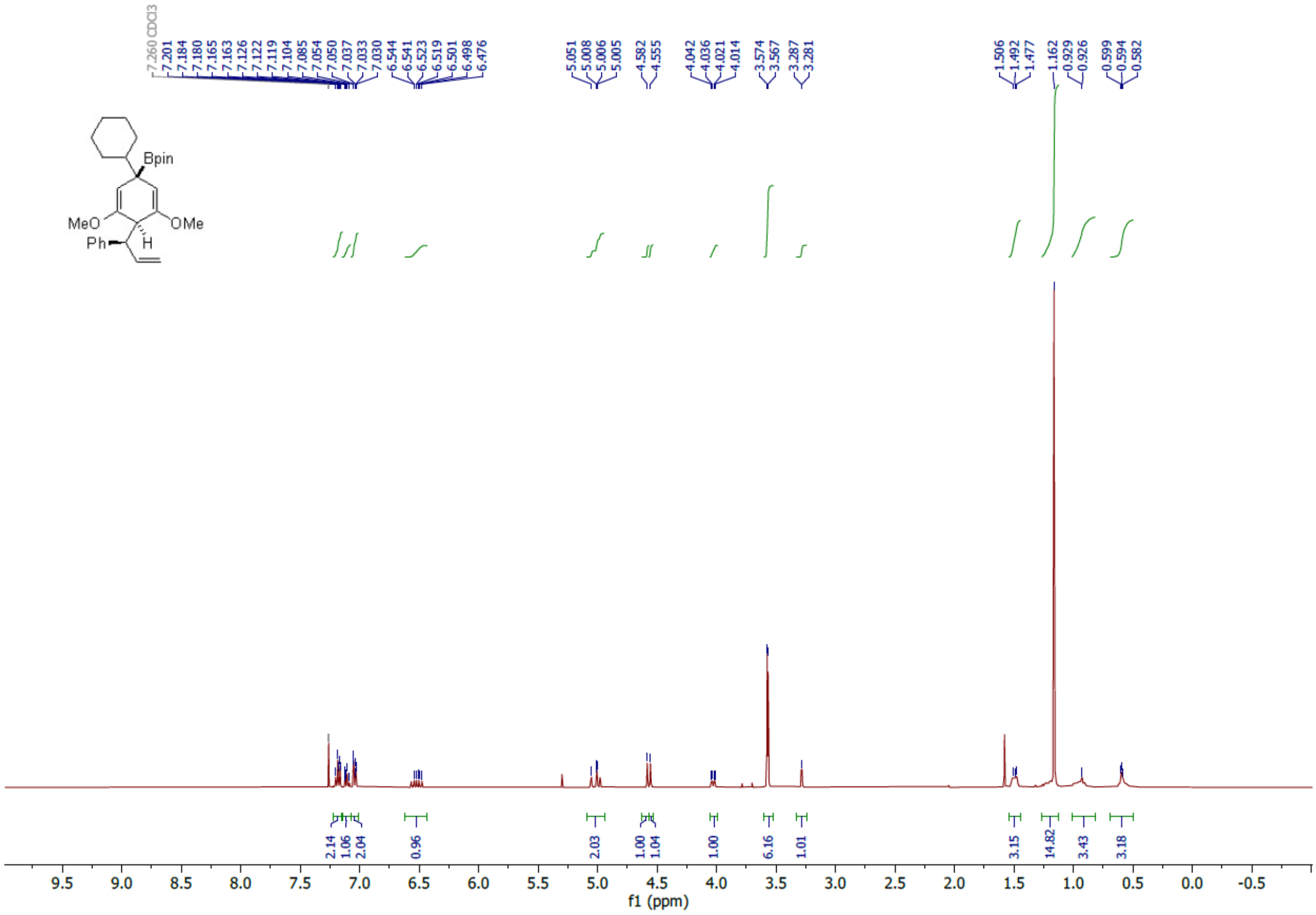

^{13}C NMR (126 MHz, $CDCl_3$) of **4a-major**

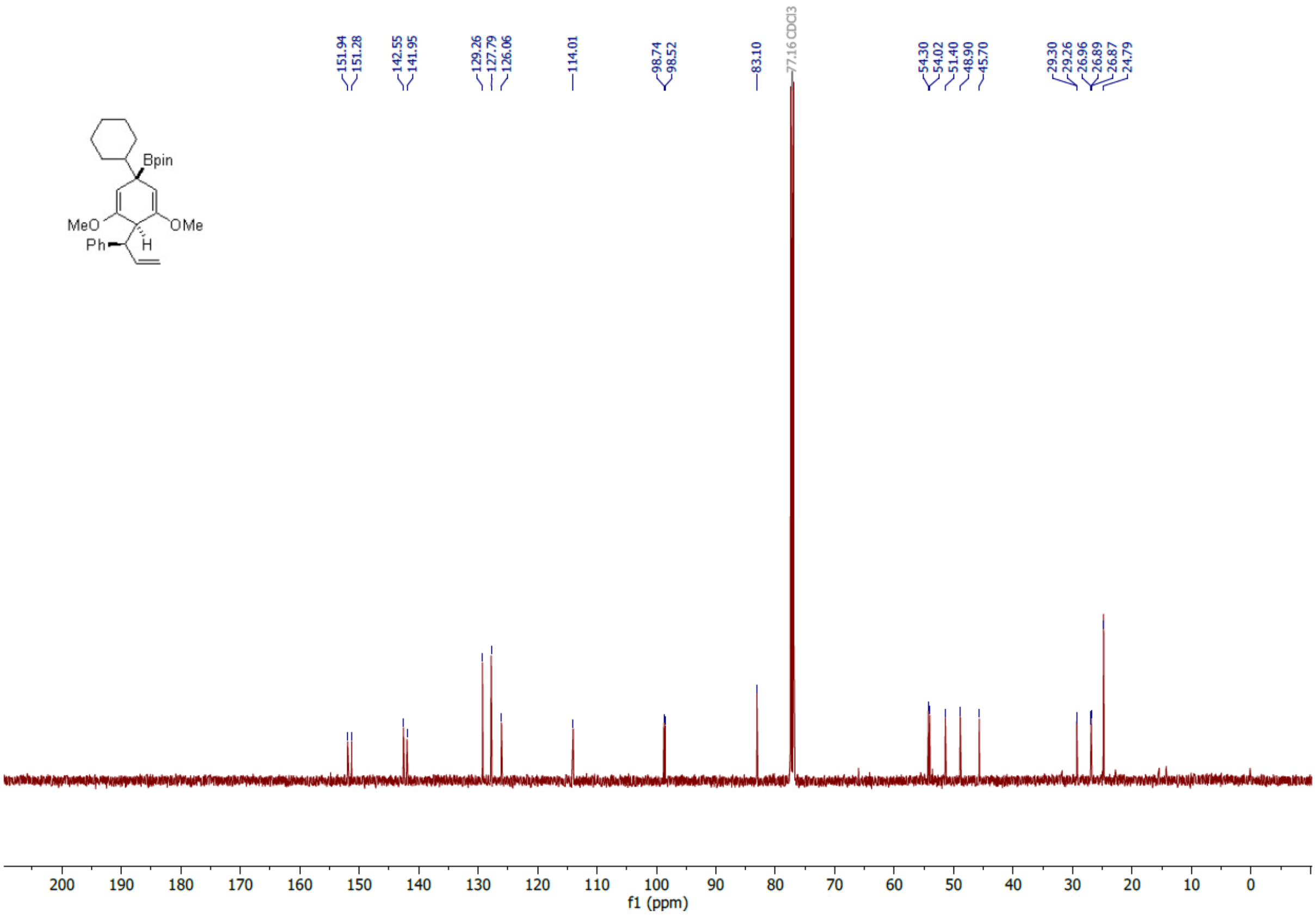

^{1}H NMR (400 MHz, $CDCl_3$) of **5a**

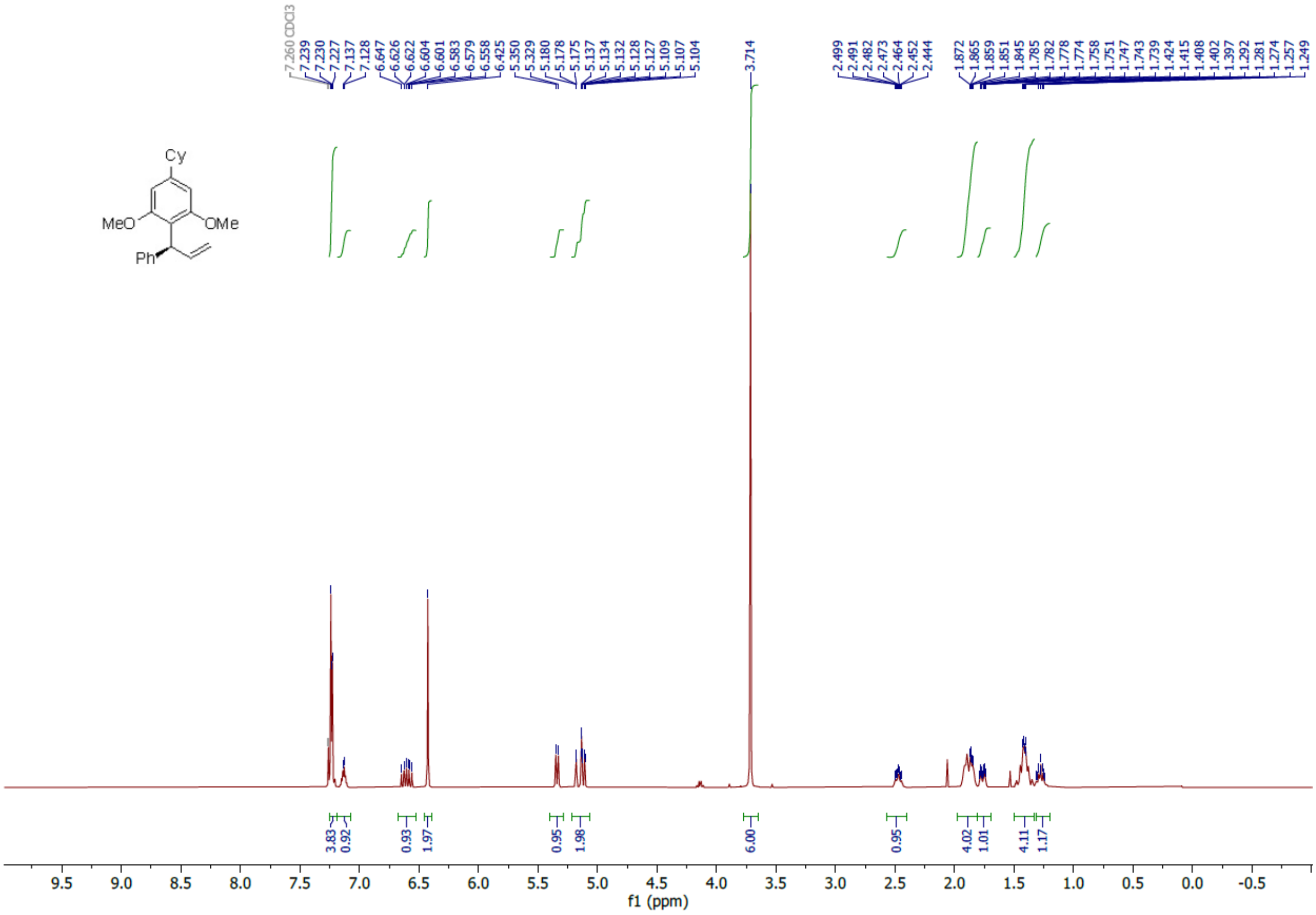

^{13}C NMR (101 MHz, $CDCl_3$) of **5a**

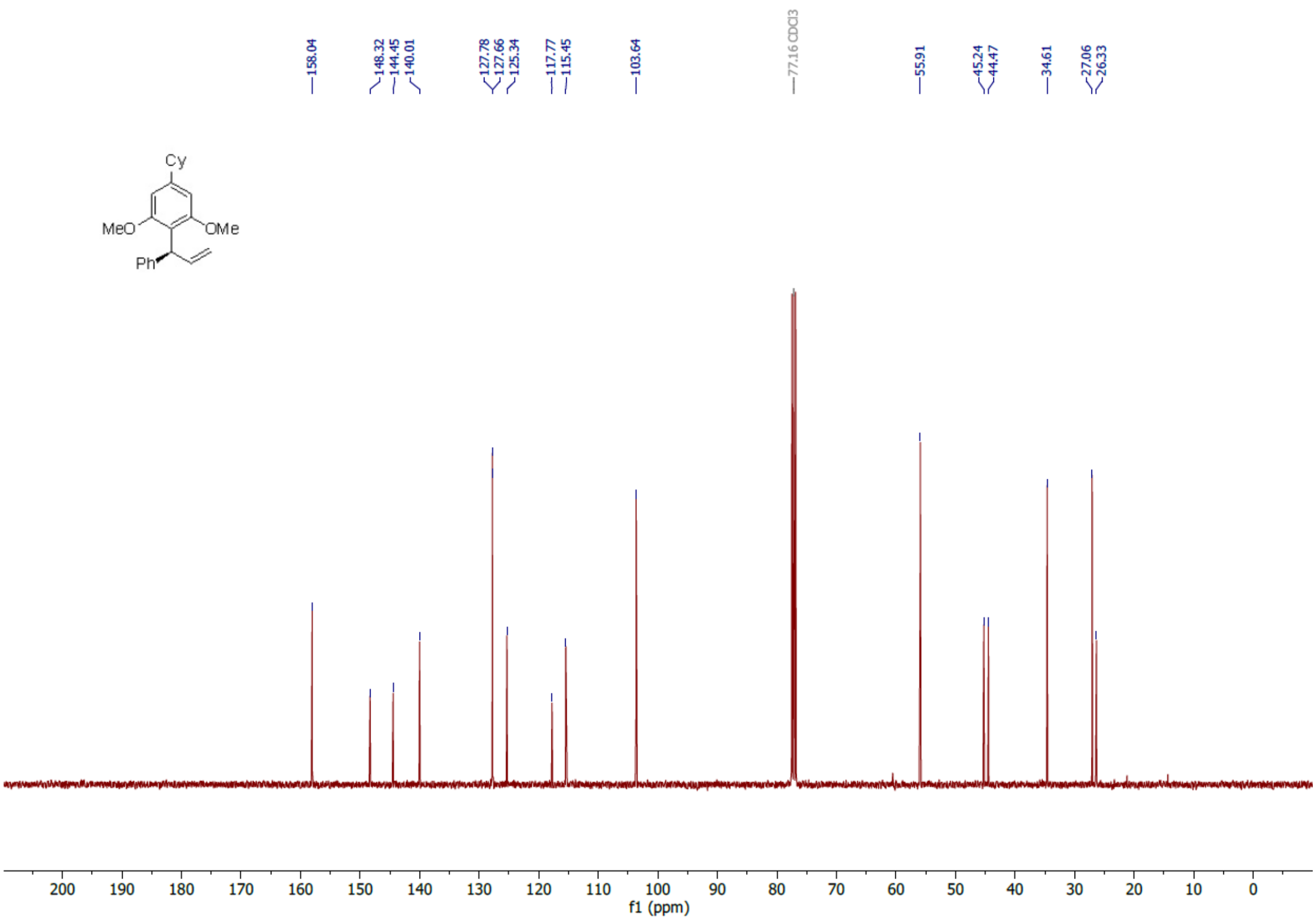

^{1}H NMR (400 MHz, $CDCl_3$) of **5b**

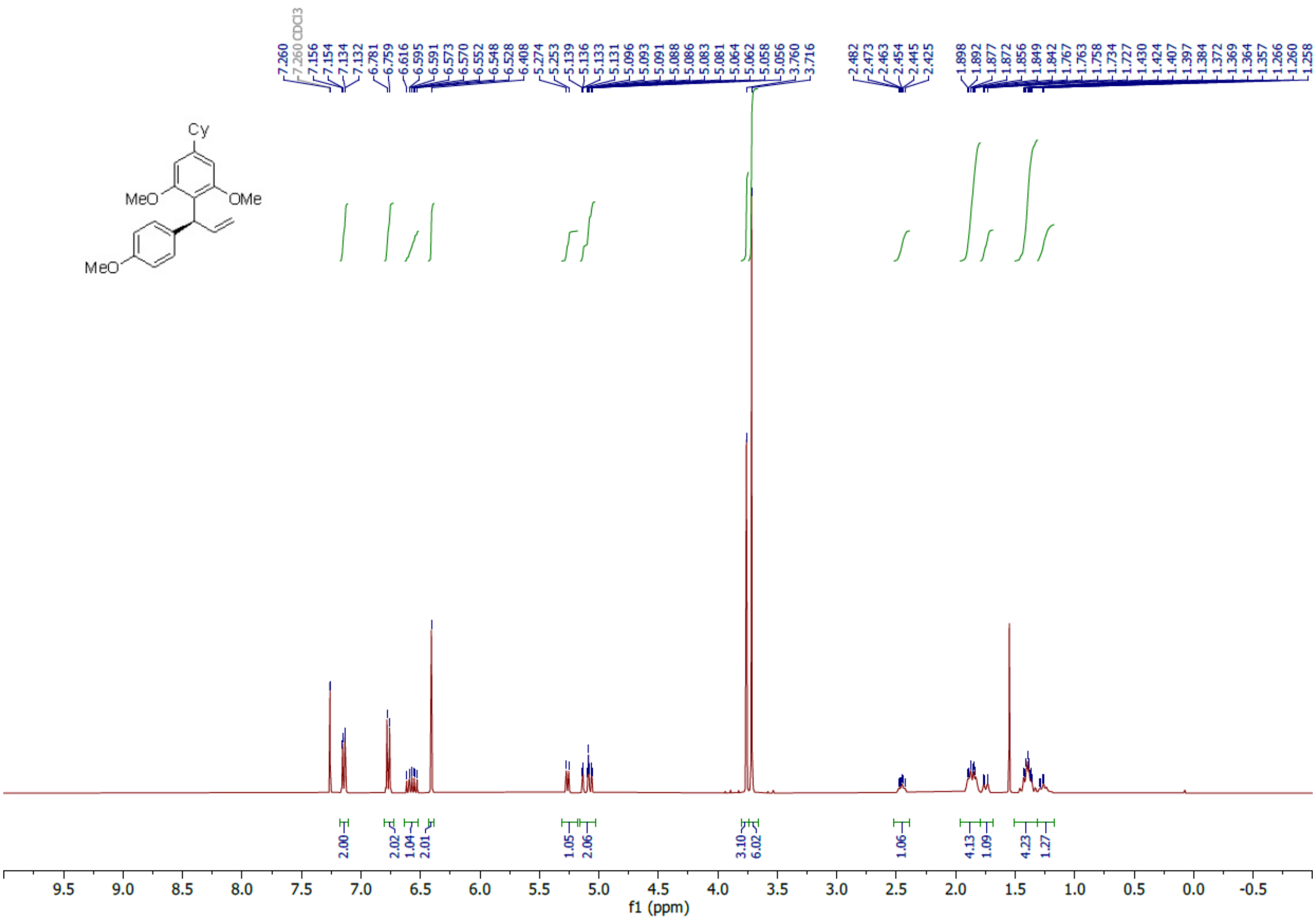

^{13}C NMR (101 MHz, $CDCl_3$) of **5b**

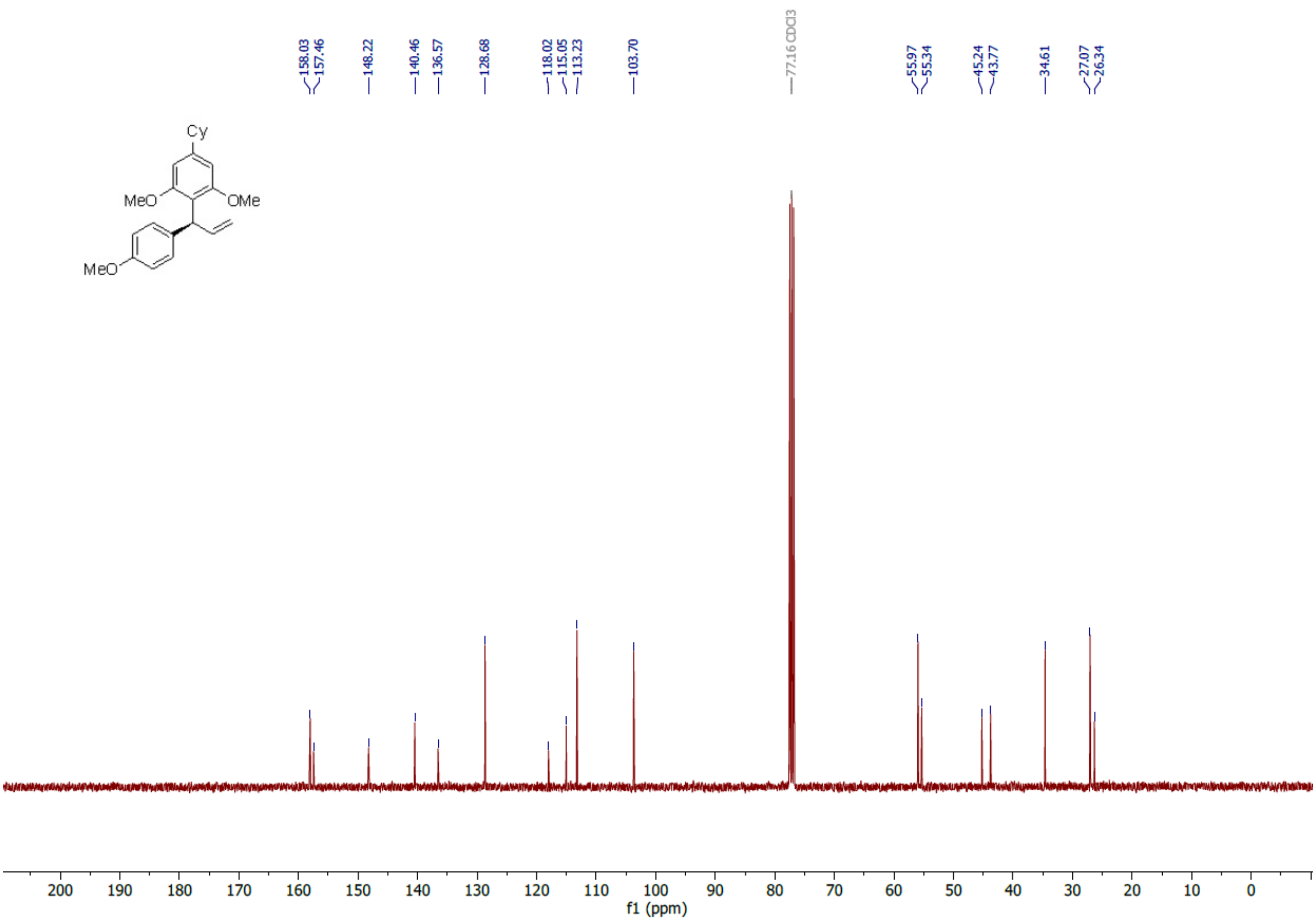

^{1}H NMR (400 MHz, $CDCl_3$) of **5c**

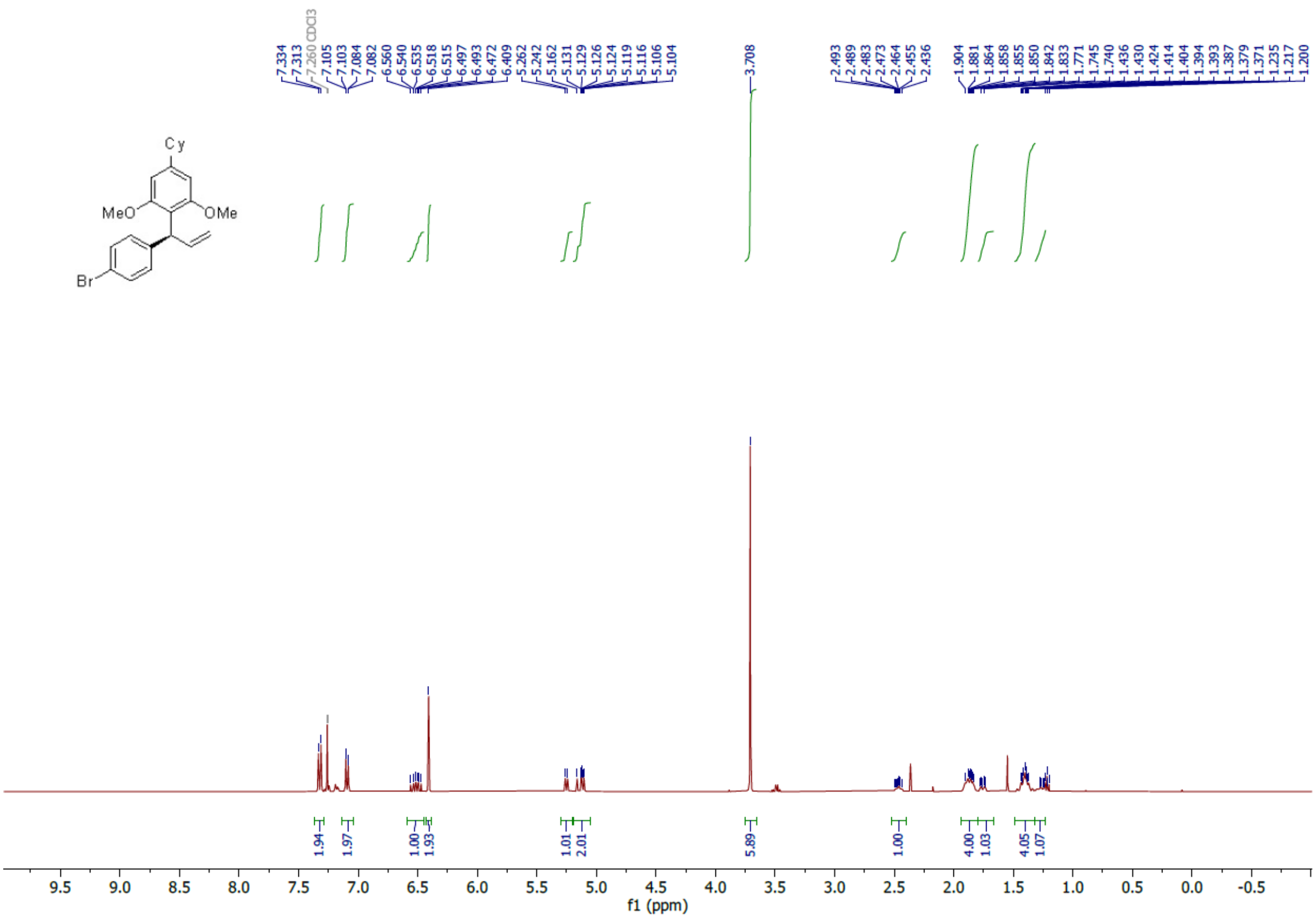

^{13}C NMR (101 MHz, $CDCl_3$) of **5c**

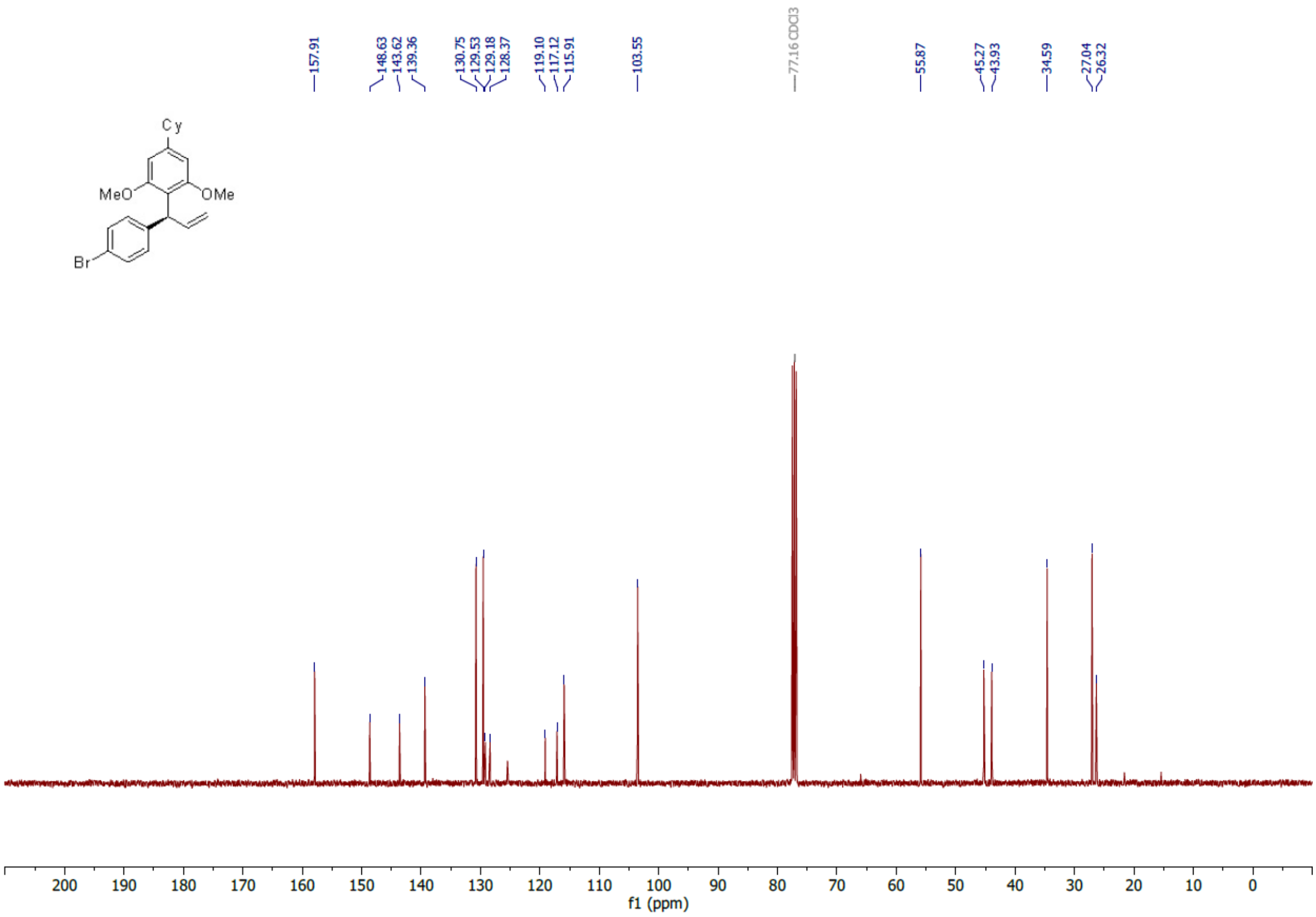

^{1}H NMR (500 MHz, $CDCl_3$) of **5d**

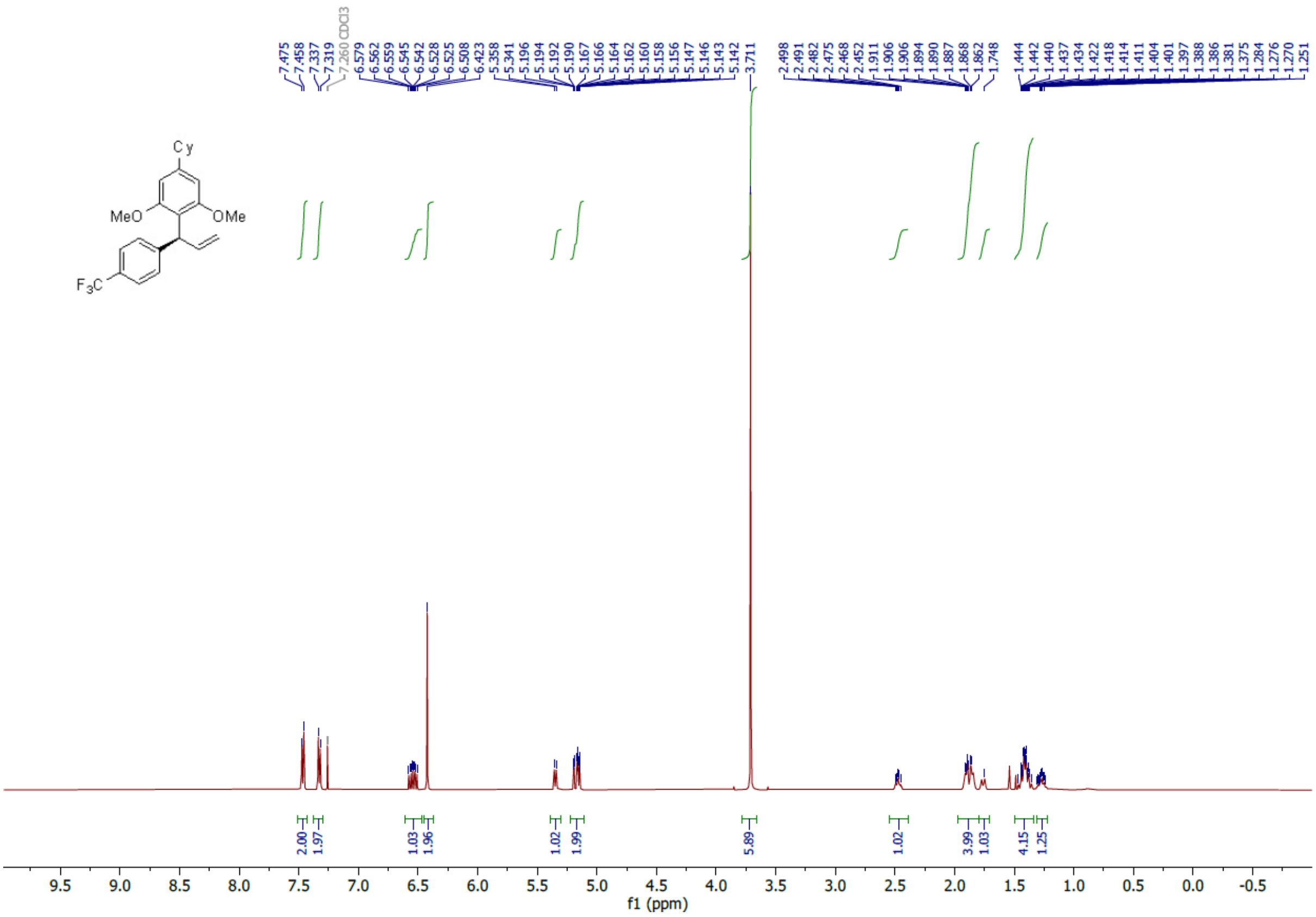

^{13}C NMR (126 MHz, $CDCl_3$) of **5d**

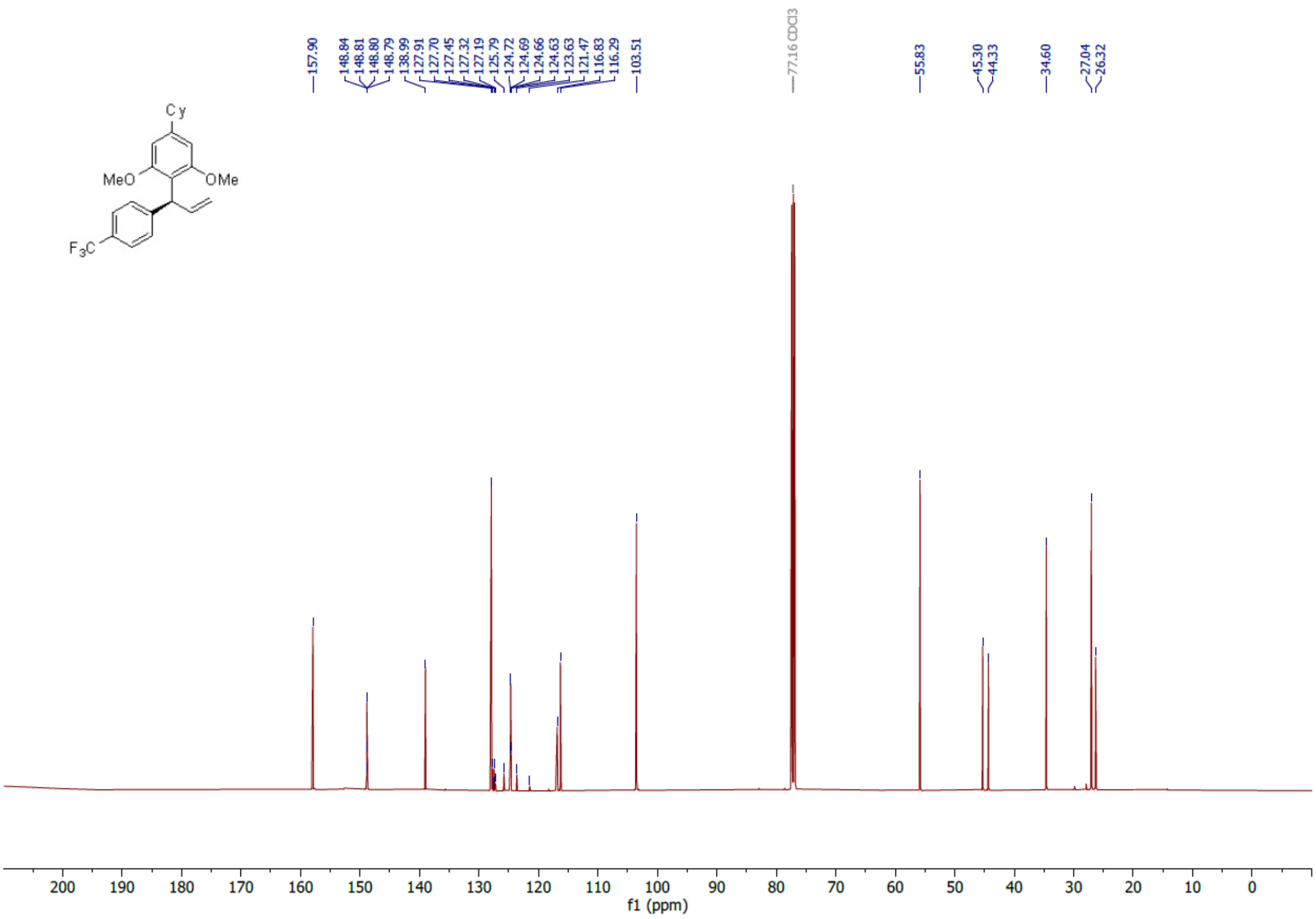

^{19}F NMR (376 MHz, $CDCl_3$) of **5d**

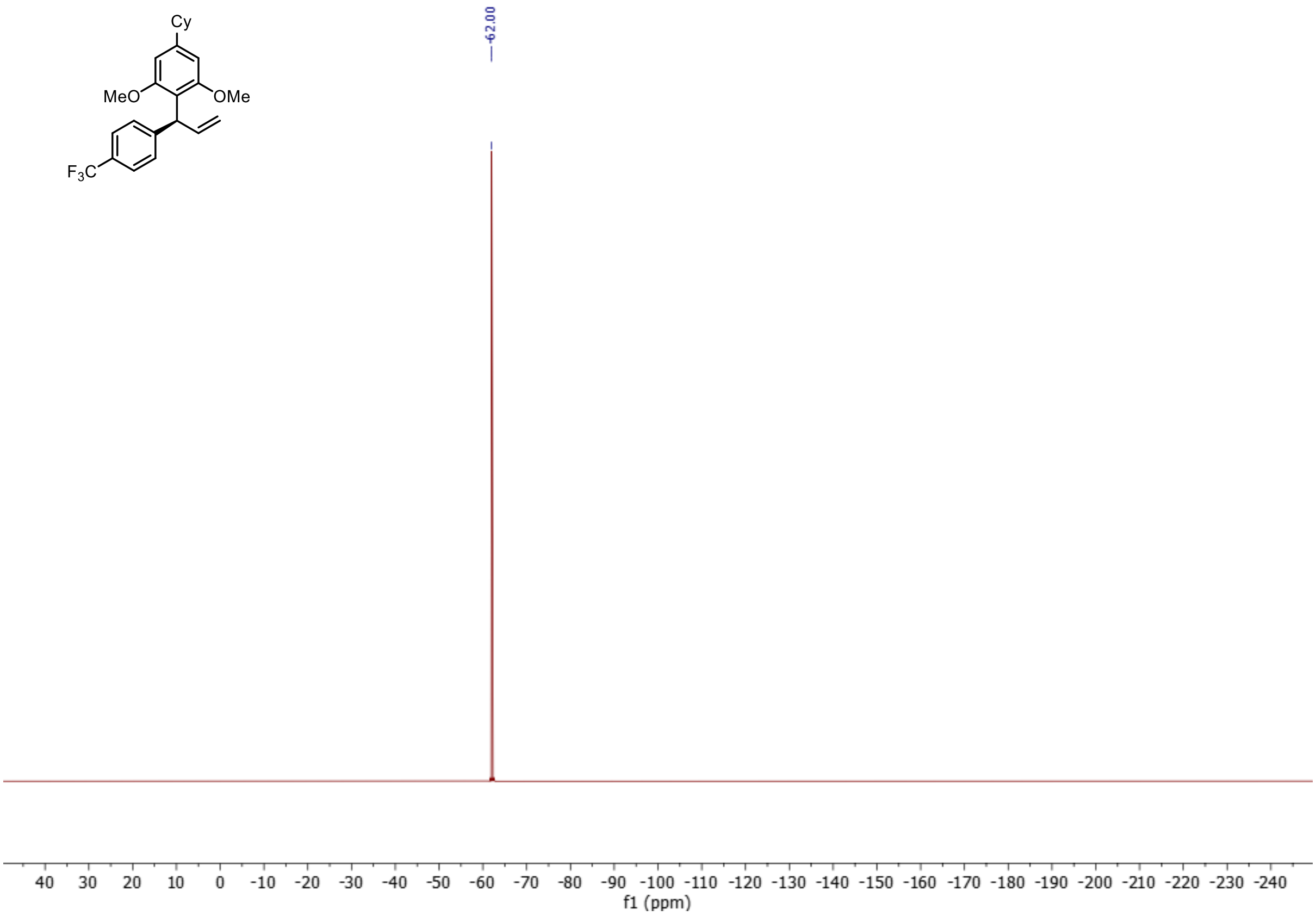

^{1}H NMR (500 MHz, $CDCl_3$) of **5e**

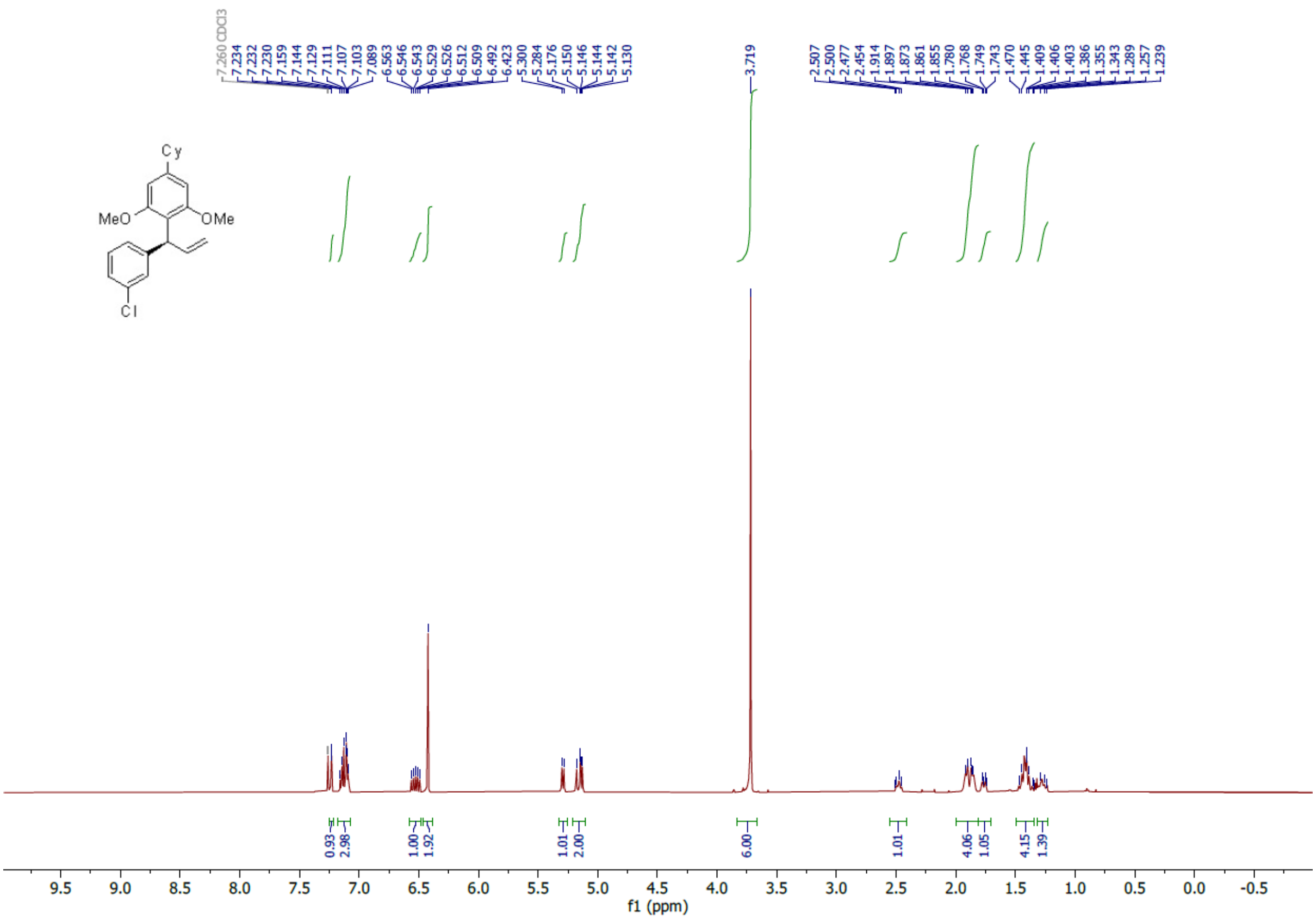

^{13}C NMR (126 MHz, $CDCl_3$) of **5e**

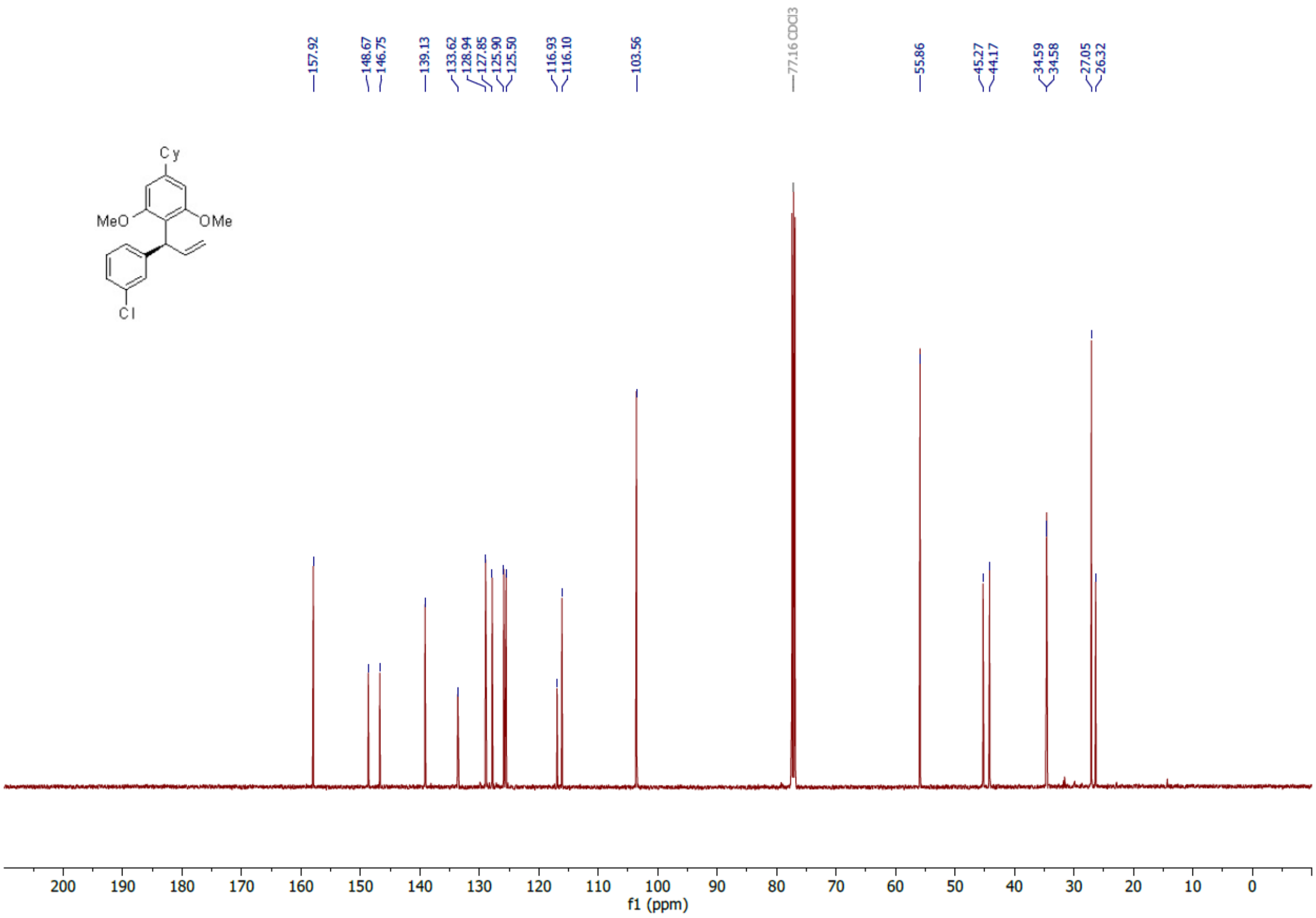

^{1}H NMR (500 MHz, $CDCl_3$) of **5f**

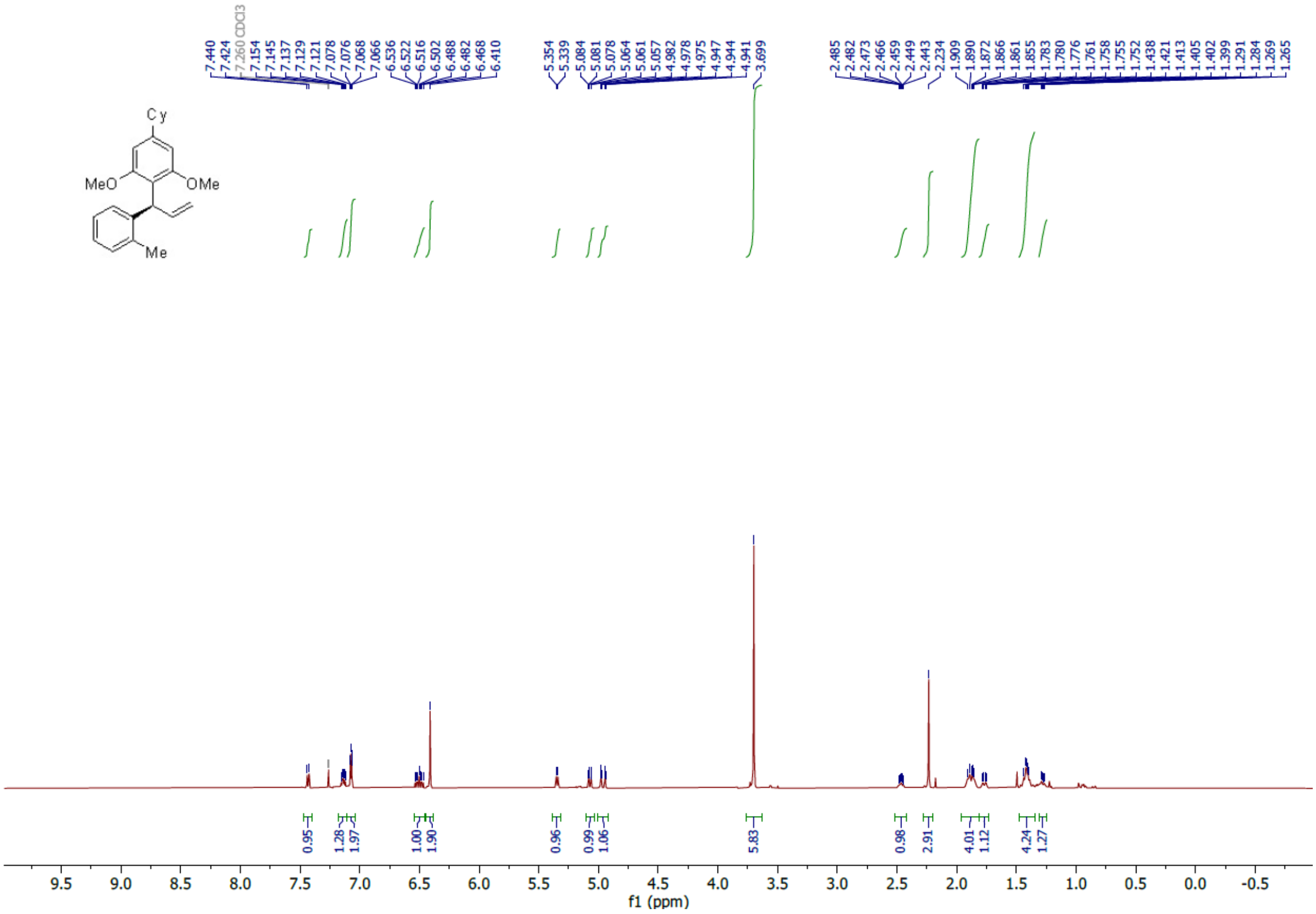

^{13}C NMR (126 MHz, $CDCl_3$) of **5f**

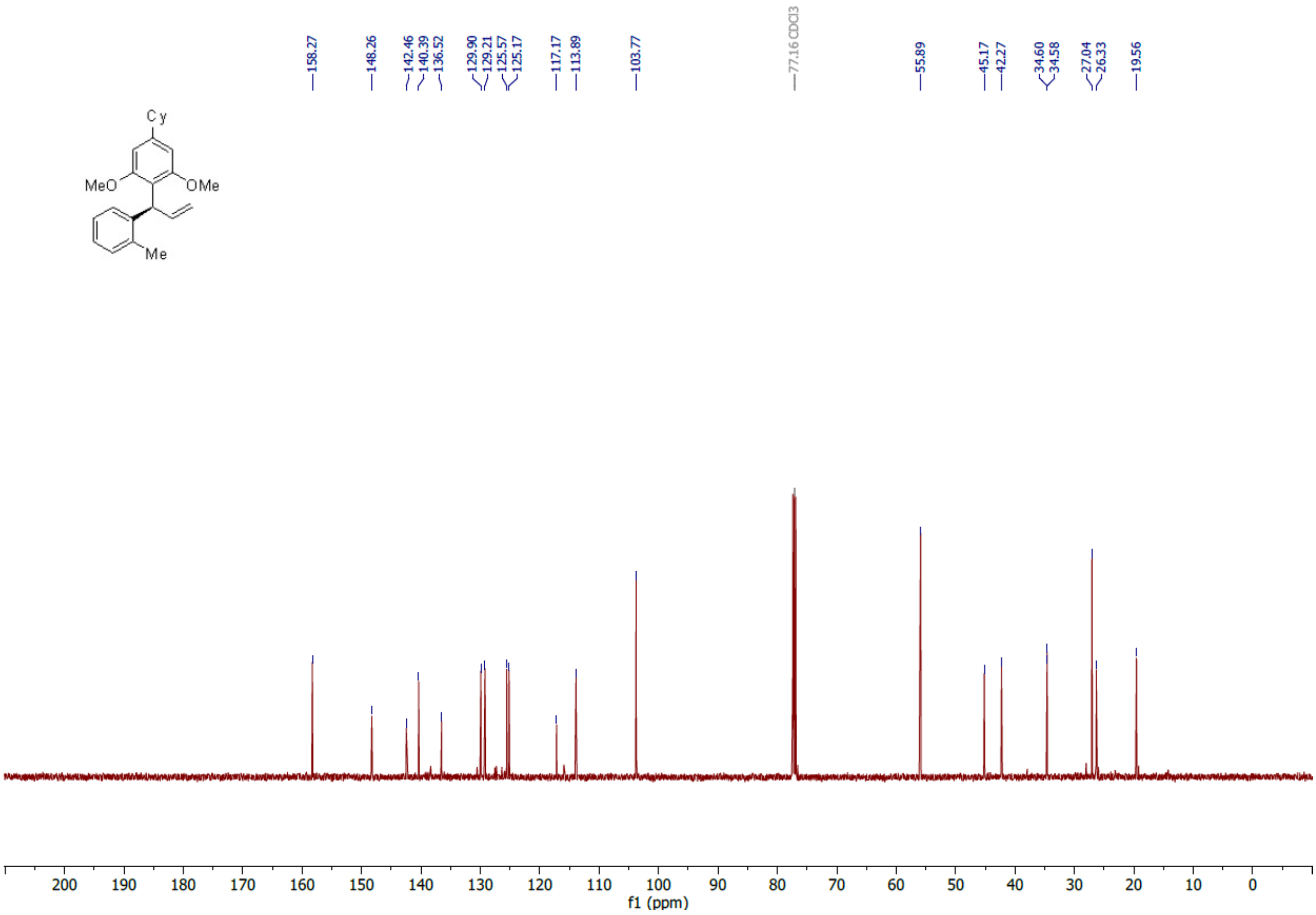

^{1}H NMR (400 MHz, $CDCl_3$) of **5g**

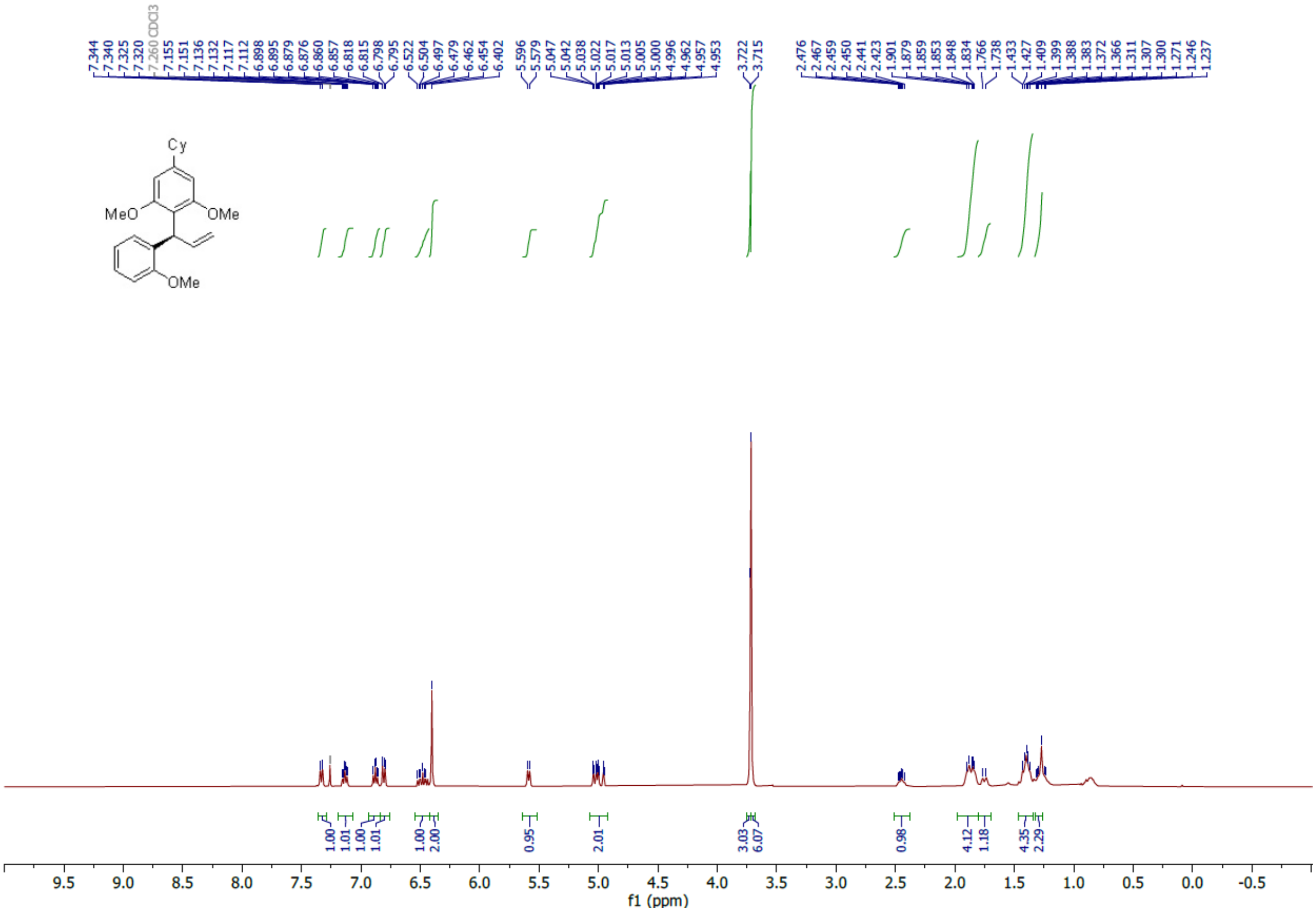

^{13}C NMR (101 MHz, $CDCl_3$) of **5g**

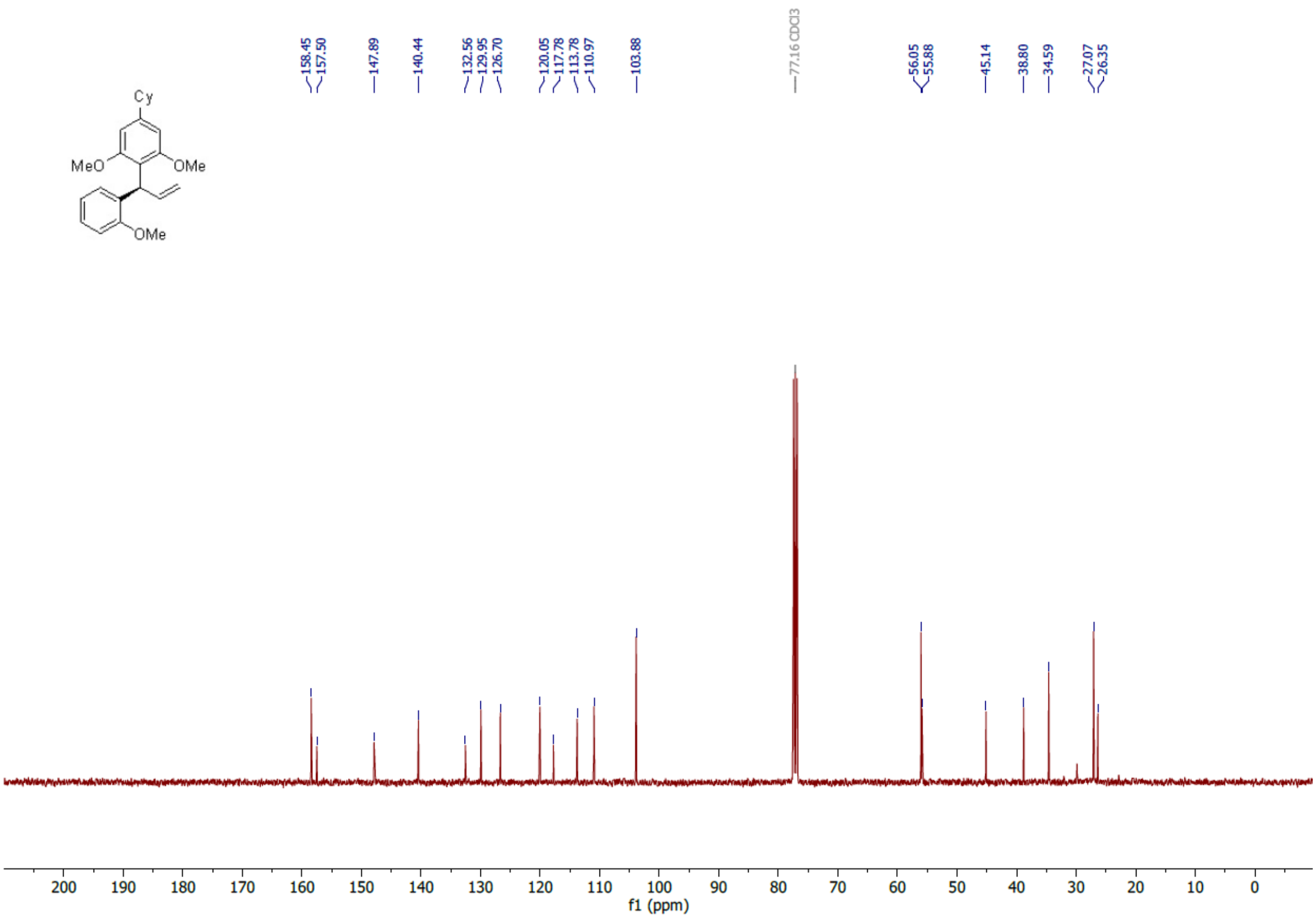

^{1}H NMR (500 MHz, $CDCl_3$) of **5h**

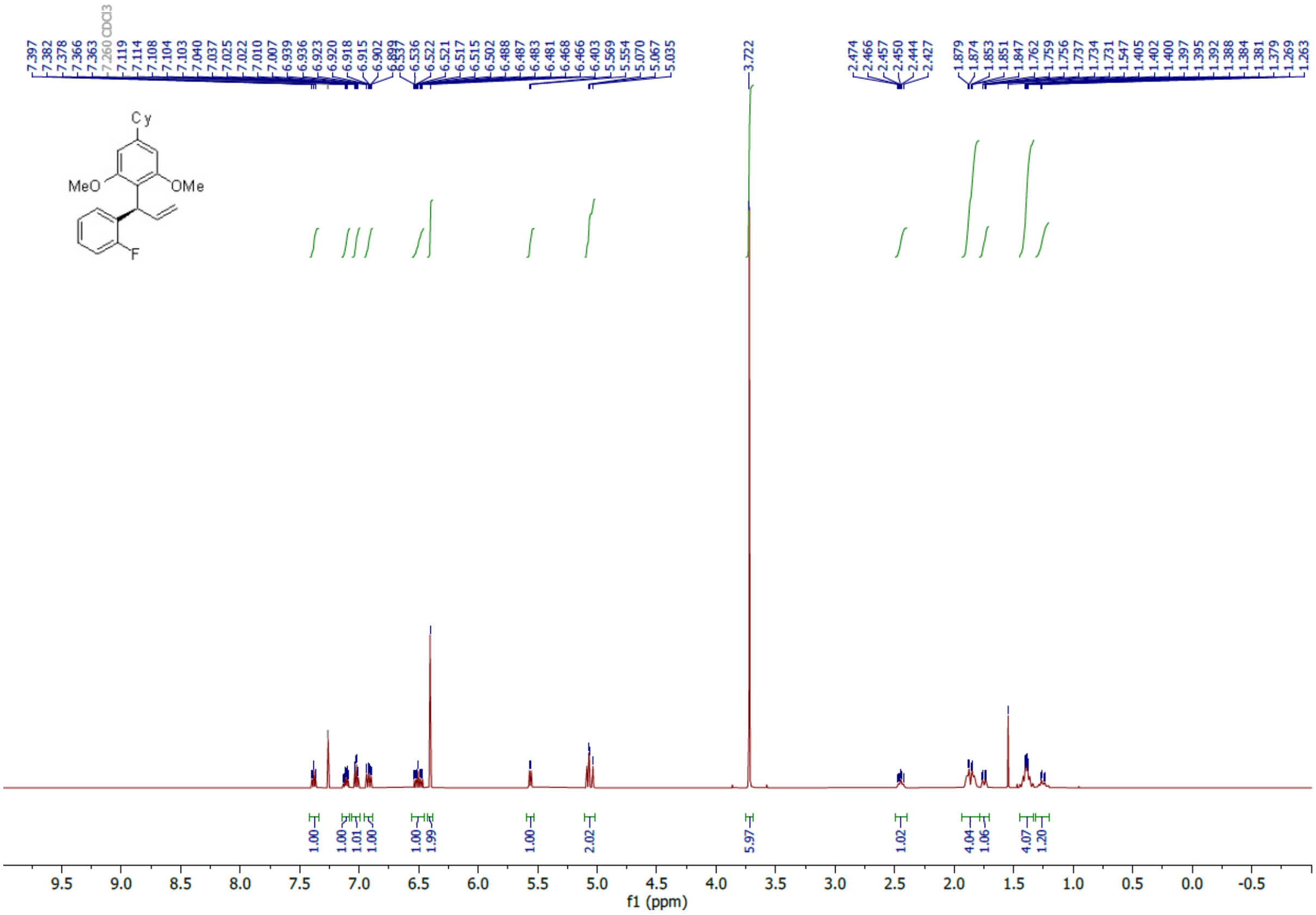

^{13}C NMR (126 MHz, $CDCl_3$) of **5h**

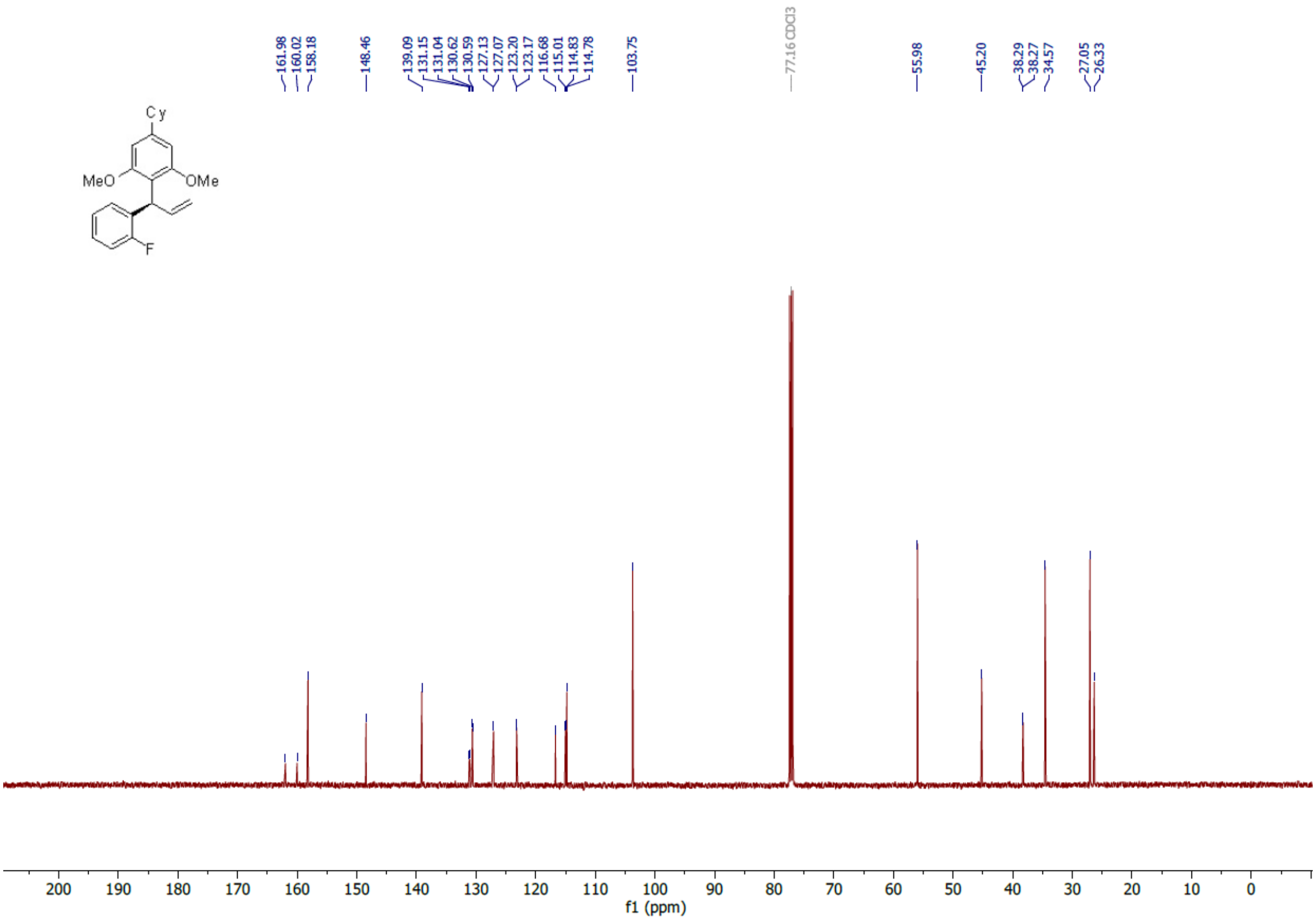

^{19}F NMR (376 MHz, $CDCl_3$) of **5h**

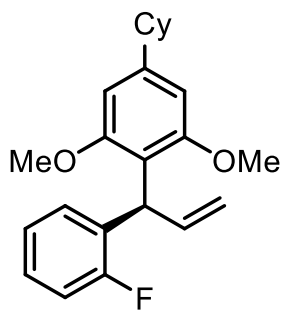

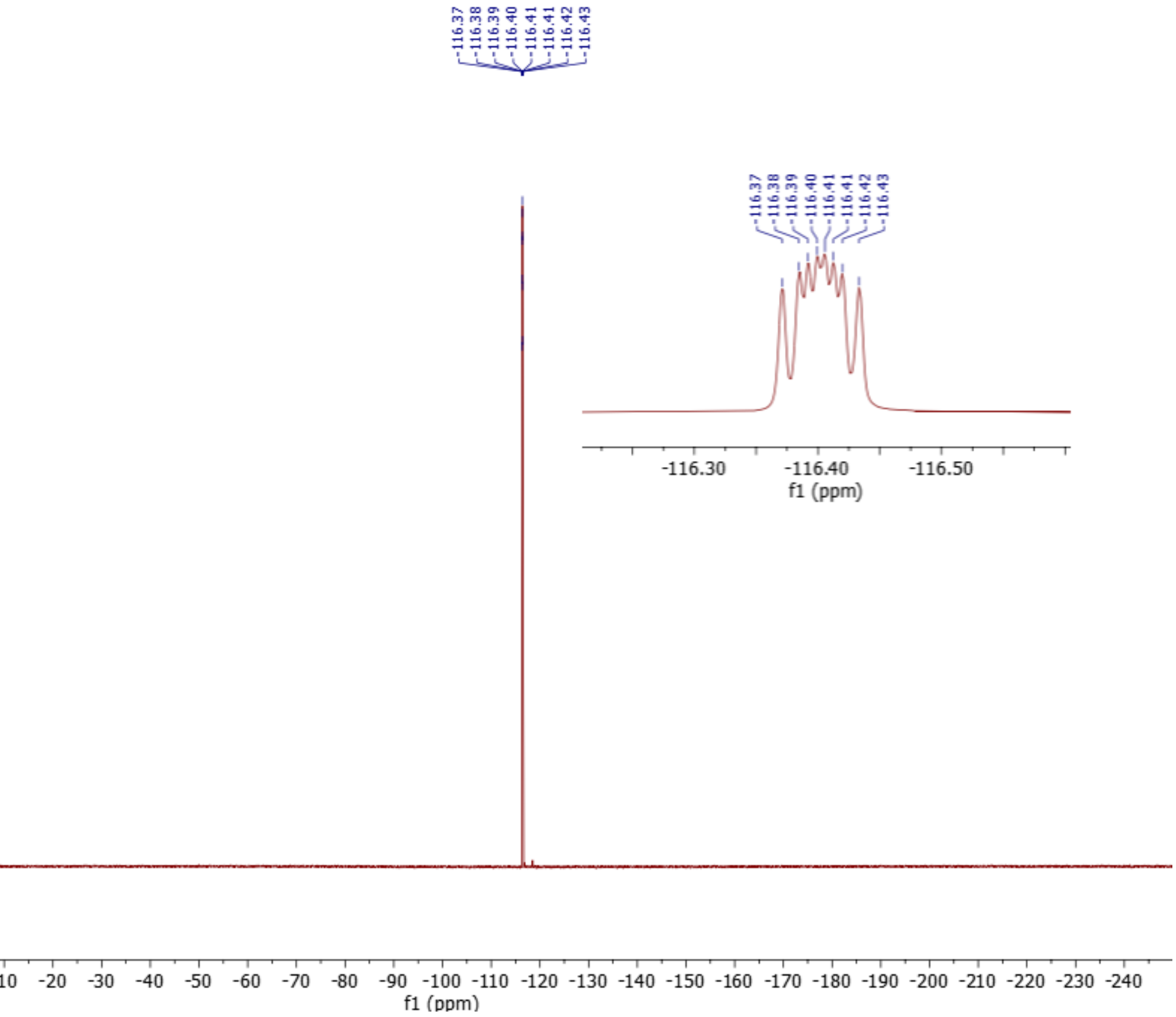

^{1}H NMR (400 MHz, $CDCl_3$) of **5i**

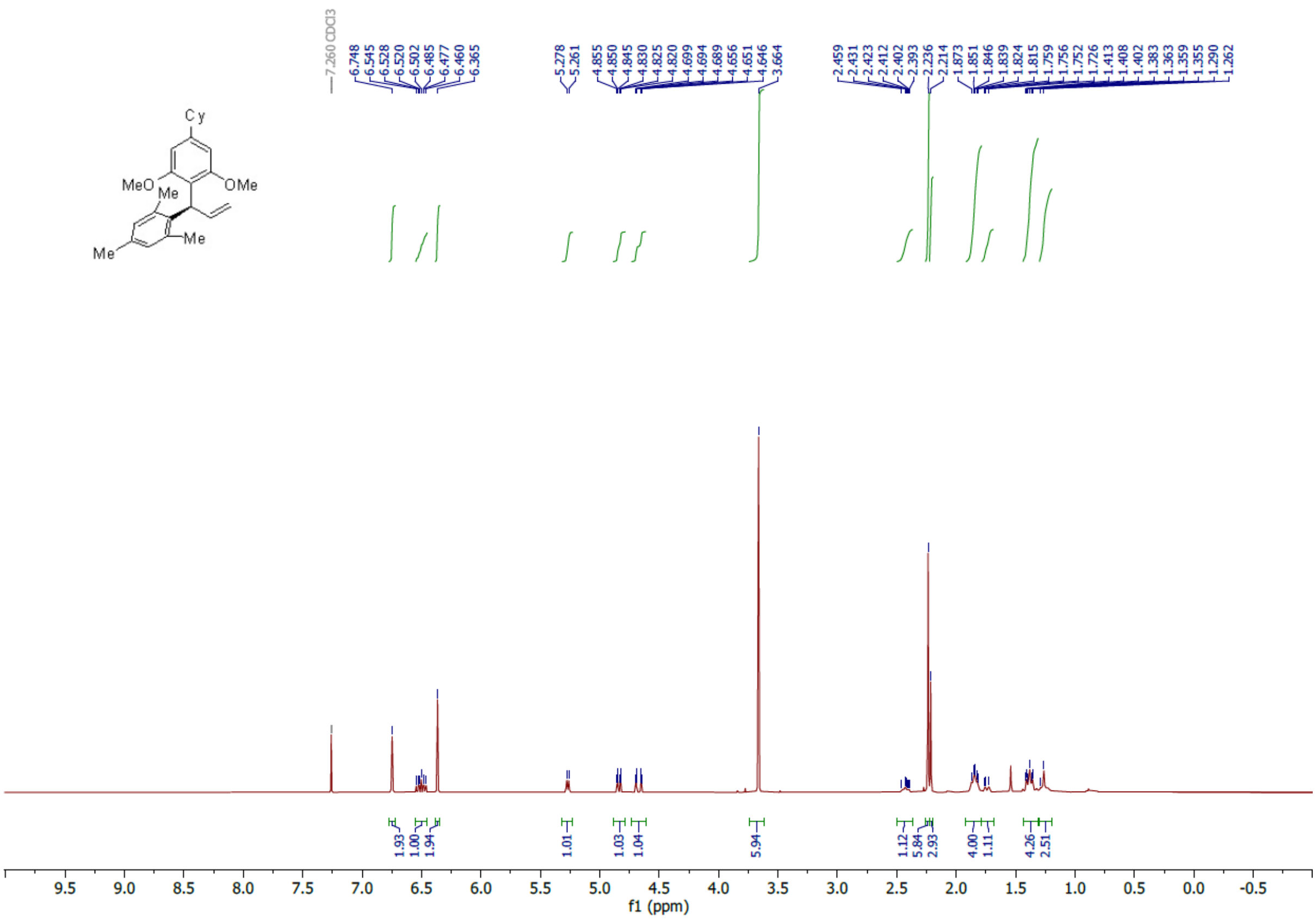

^{13}C NMR (101 MHz, $CDCl_3$) of **5i**

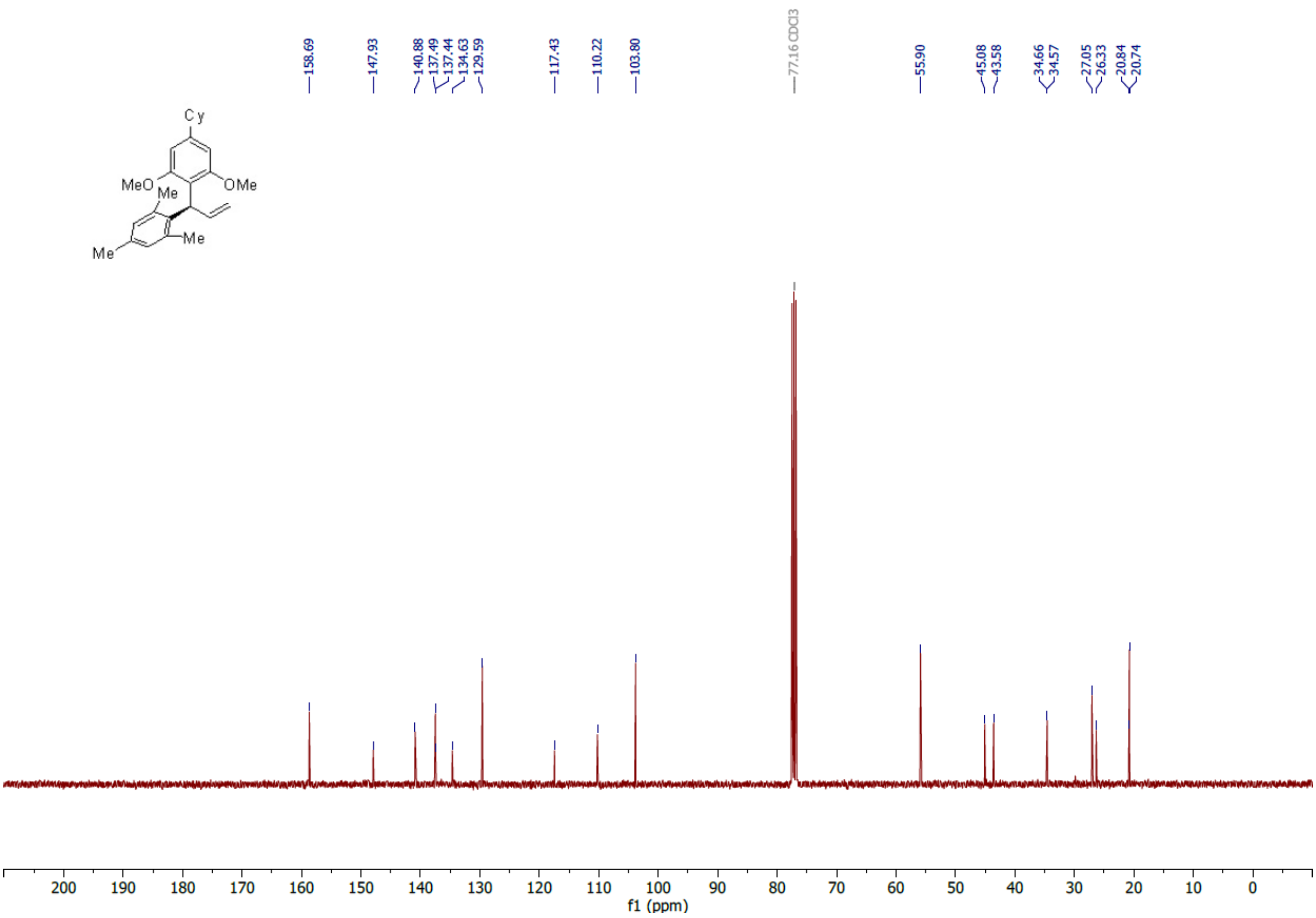

^{1}H NMR (400 MHz, $CDCl_3$) of **5j**

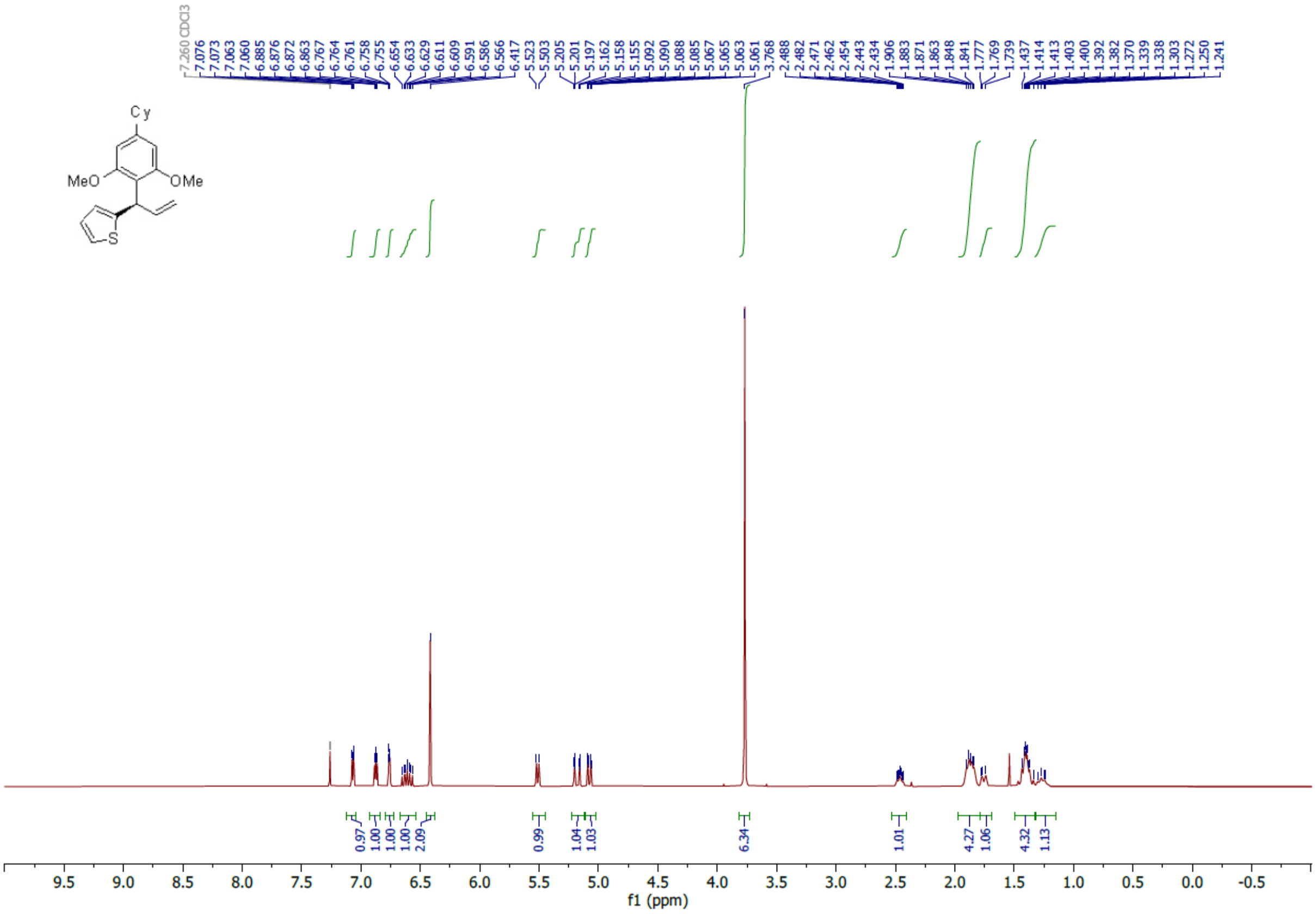

^{13}C NMR (126 MHz, $CDCl_3$) of **5j**

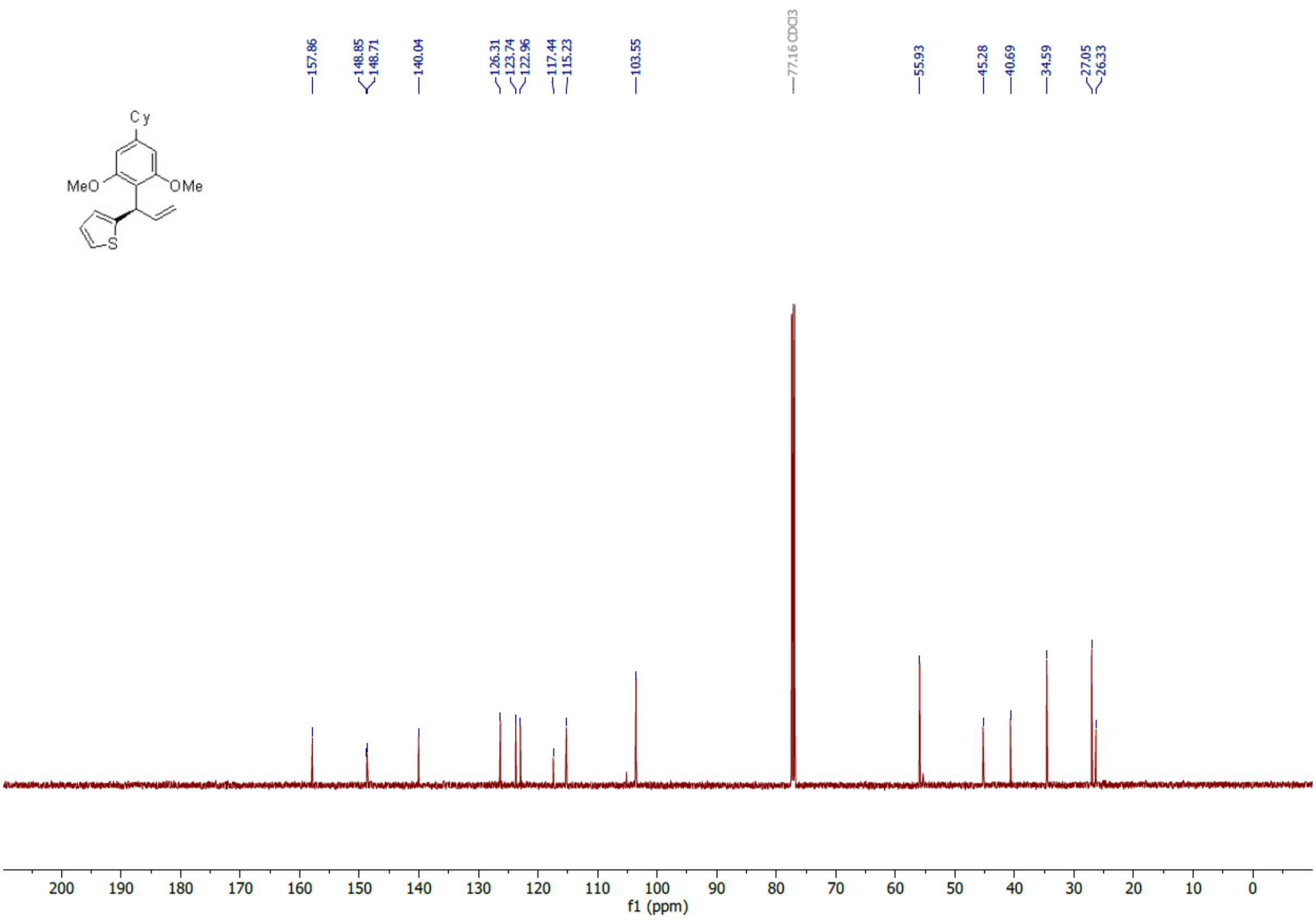

^{1}H NMR (500 MHz, $CDCl_3$) of **5k**

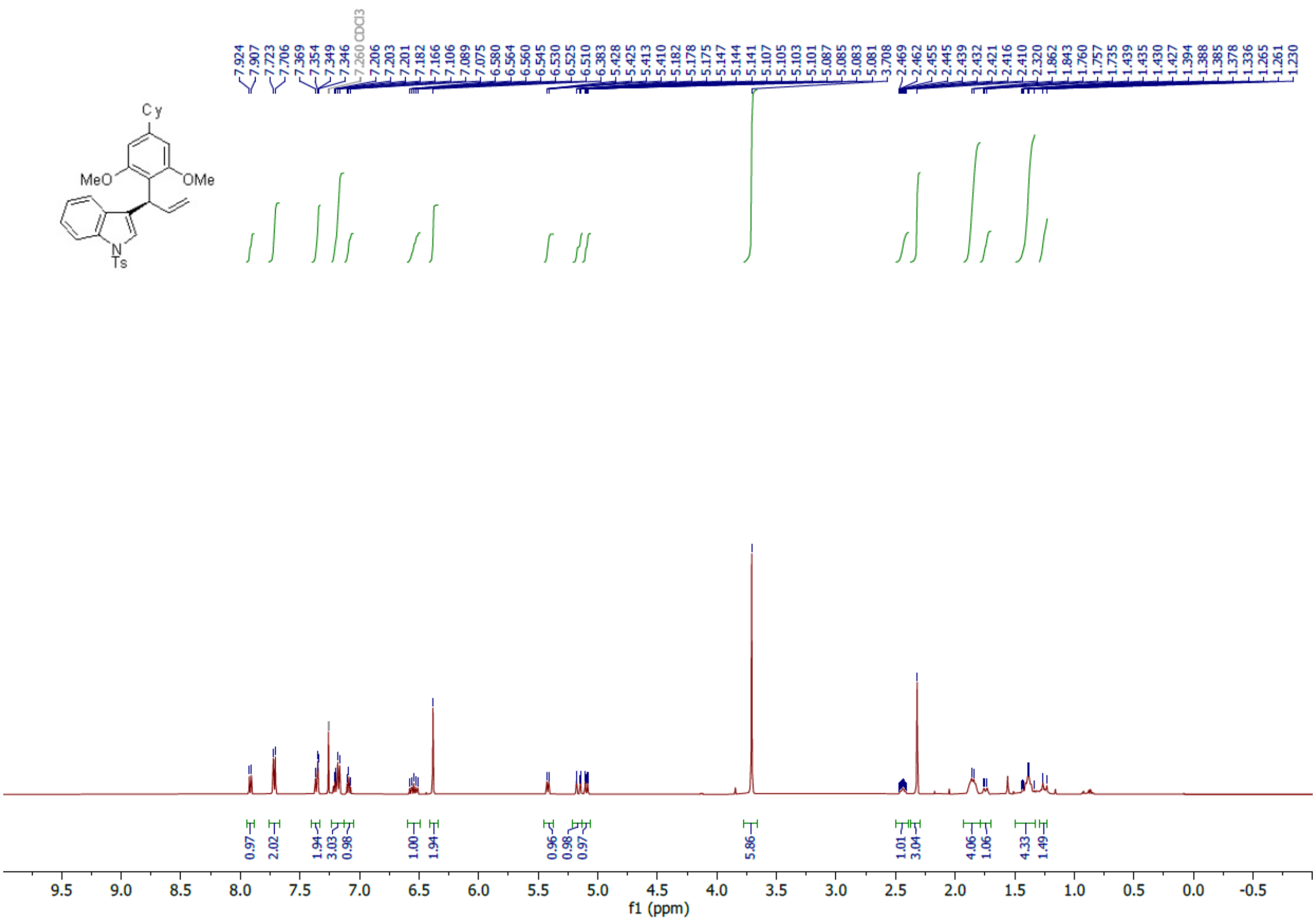

^{13}C NMR (126 MHz, $CDCl_3$) of **5k**

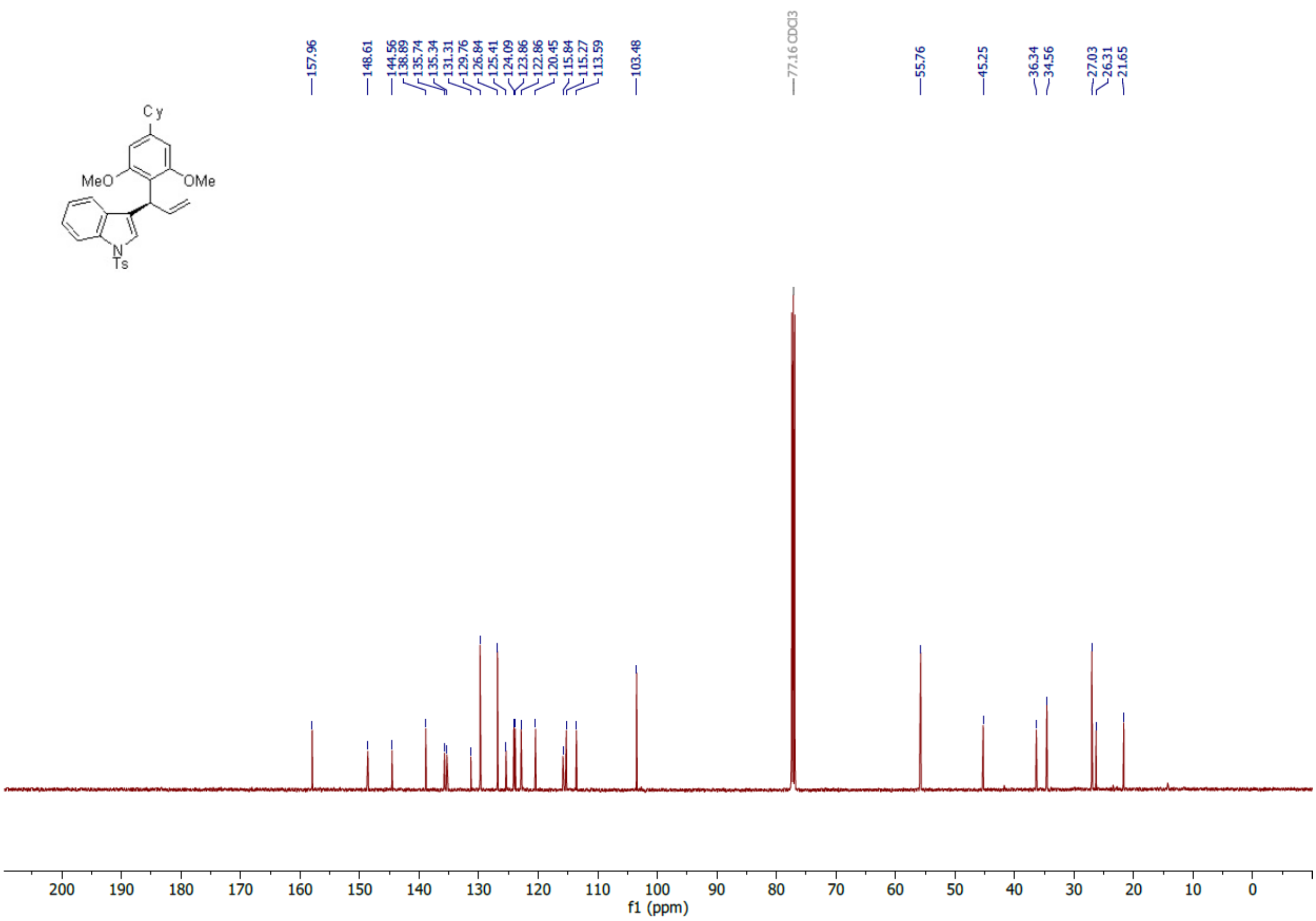

^{1}H NMR (500 MHz, $CDCl_3$) of **5m**

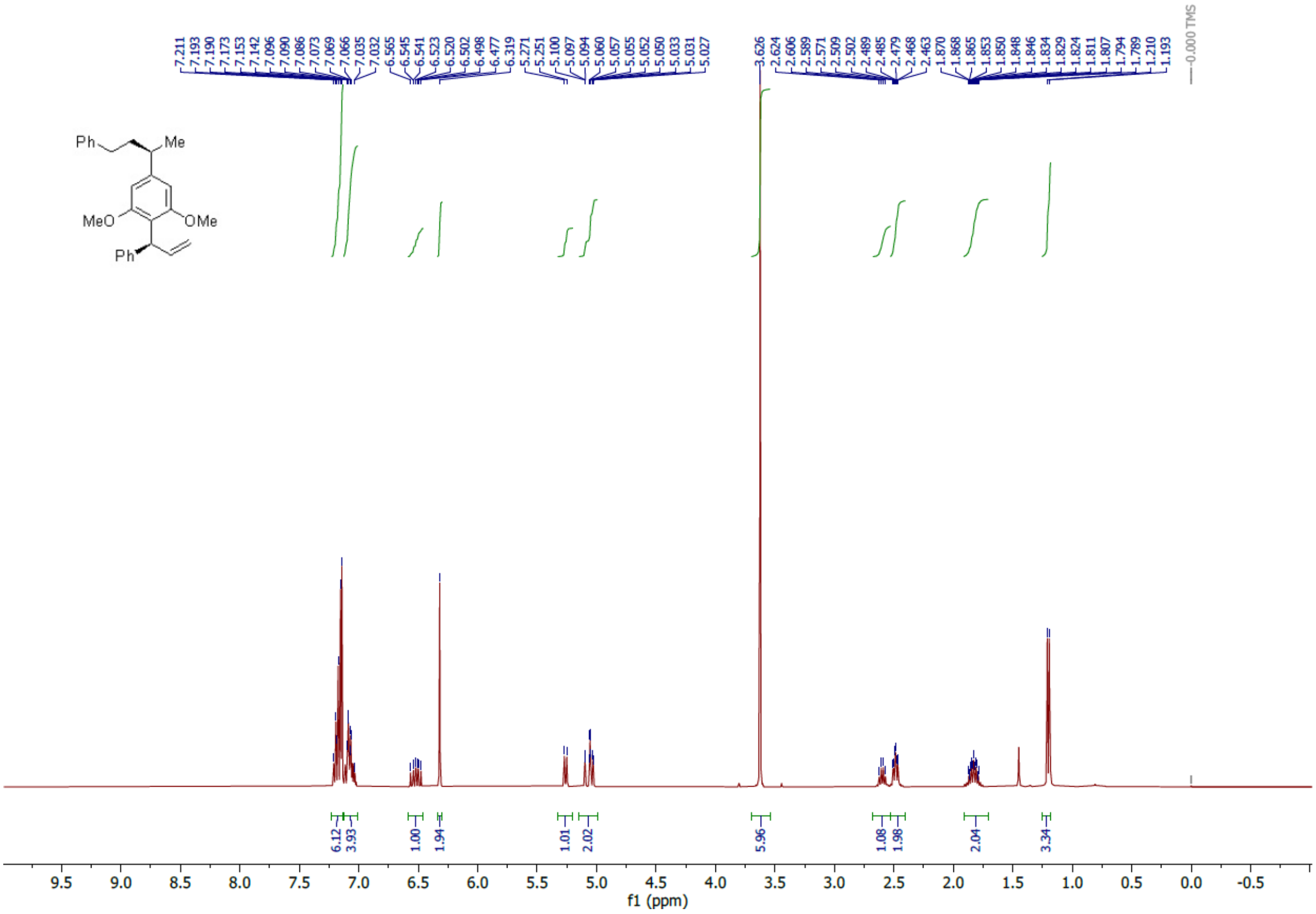

^{13}C NMR (101 MHz, $CDCl_3$) of **5m**

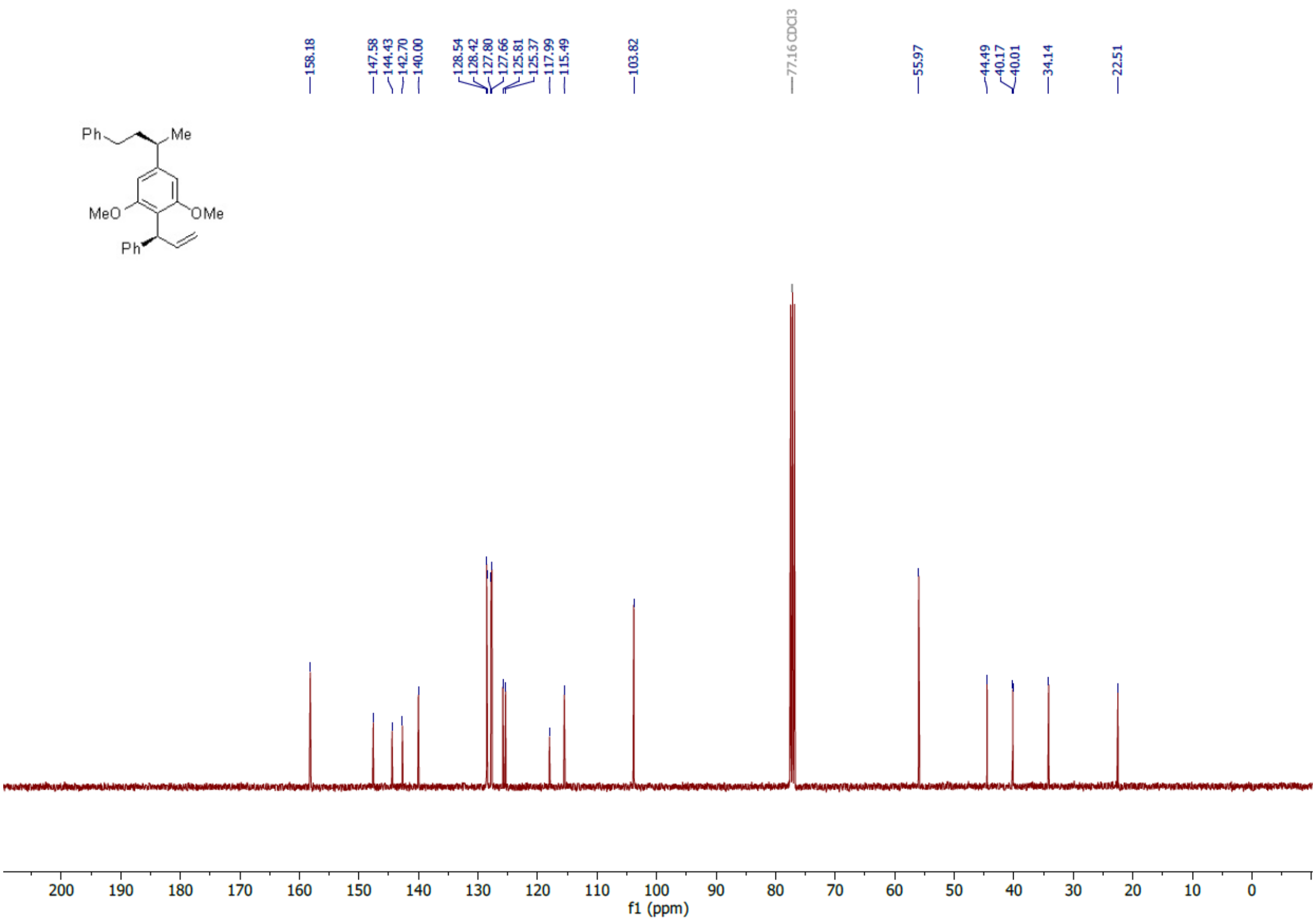

^{1}H NMR (400 MHz, $CDCl_3$) of **5q**

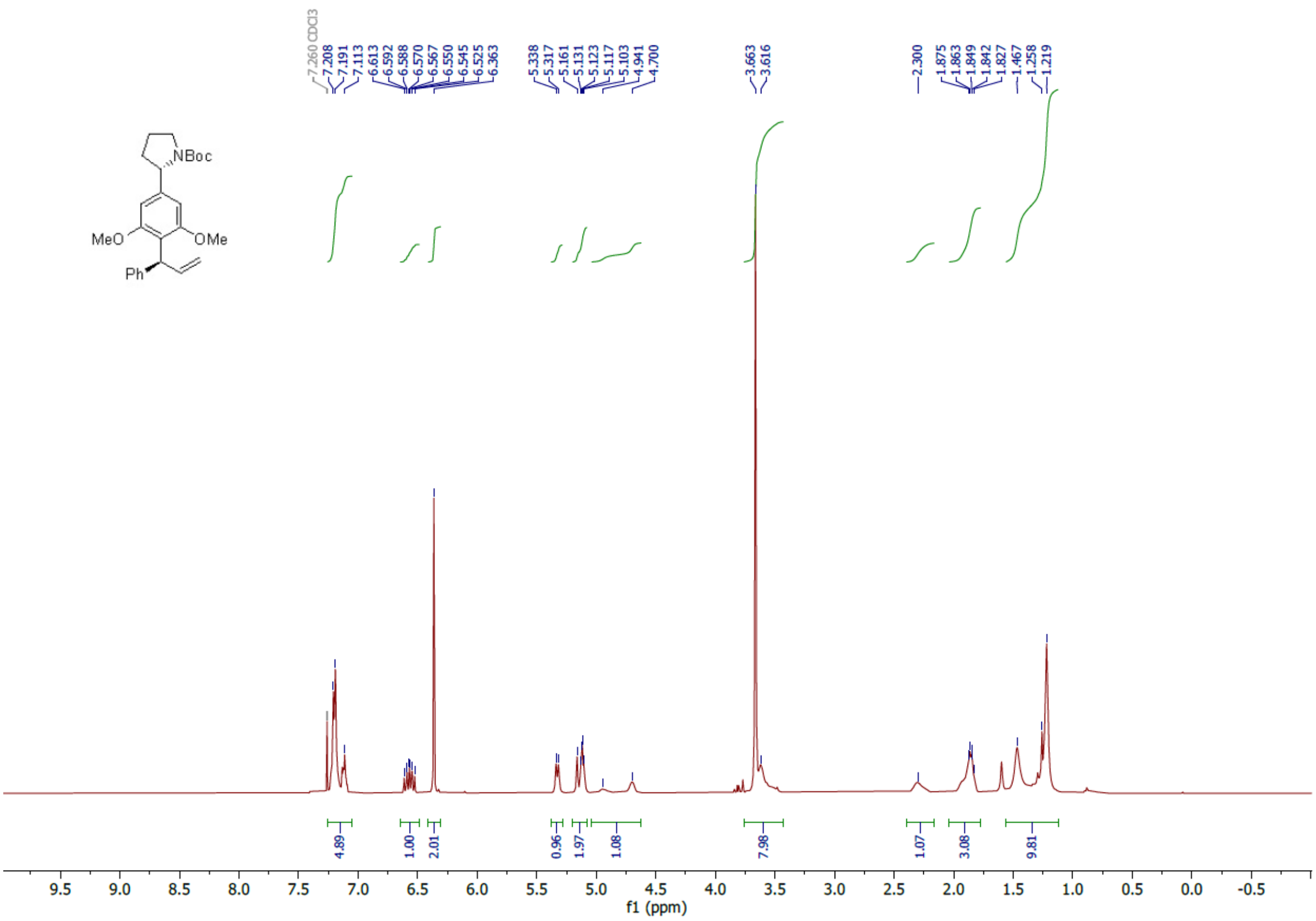

^{13}C NMR (101 MHz, $CDCl_3$) of **5q**

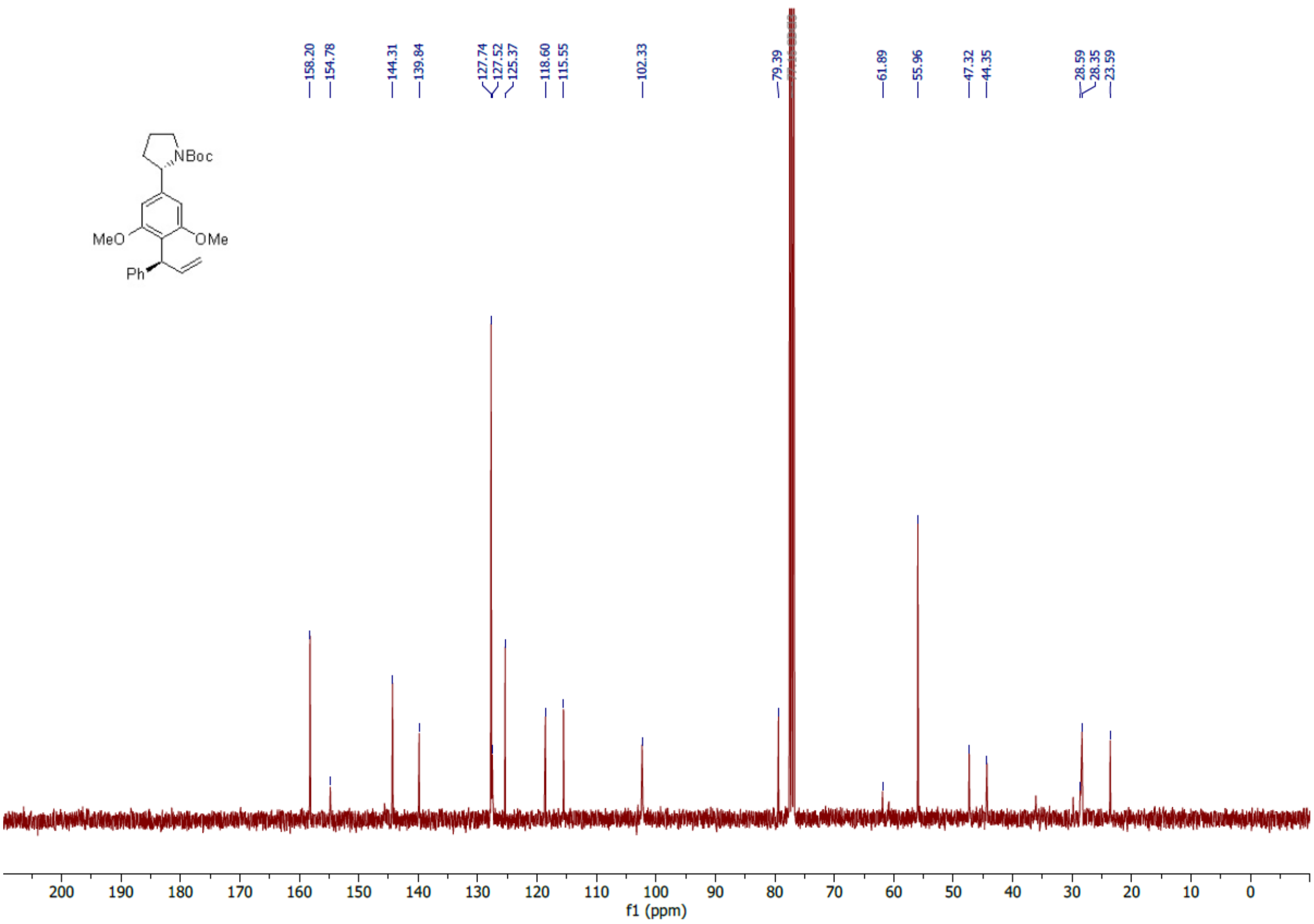

^{1}H NMR (500 MHz, $CDCl_3$) of **5r**

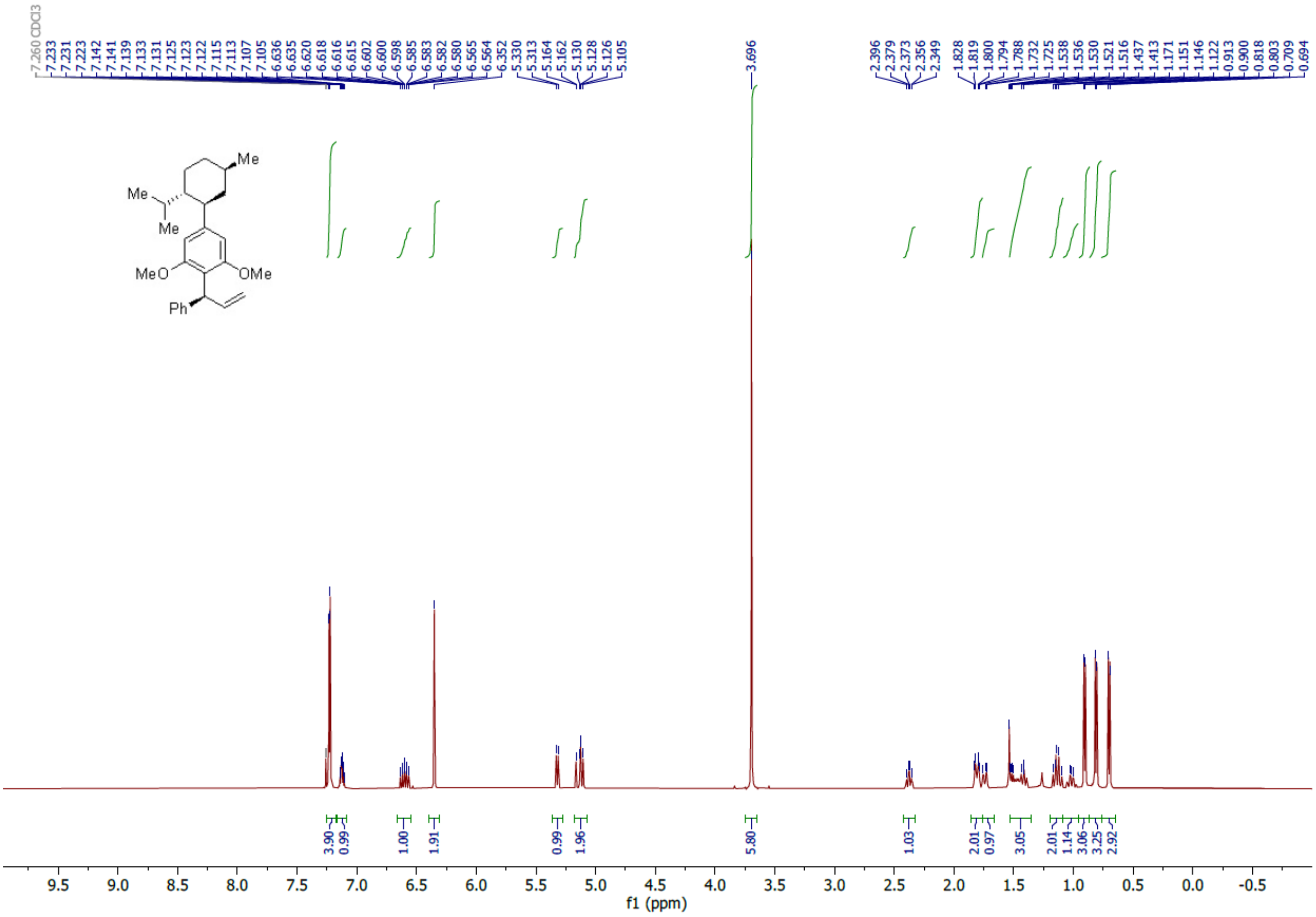

^{13}C NMR (126 MHz, $CDCl_3$) of **5r**

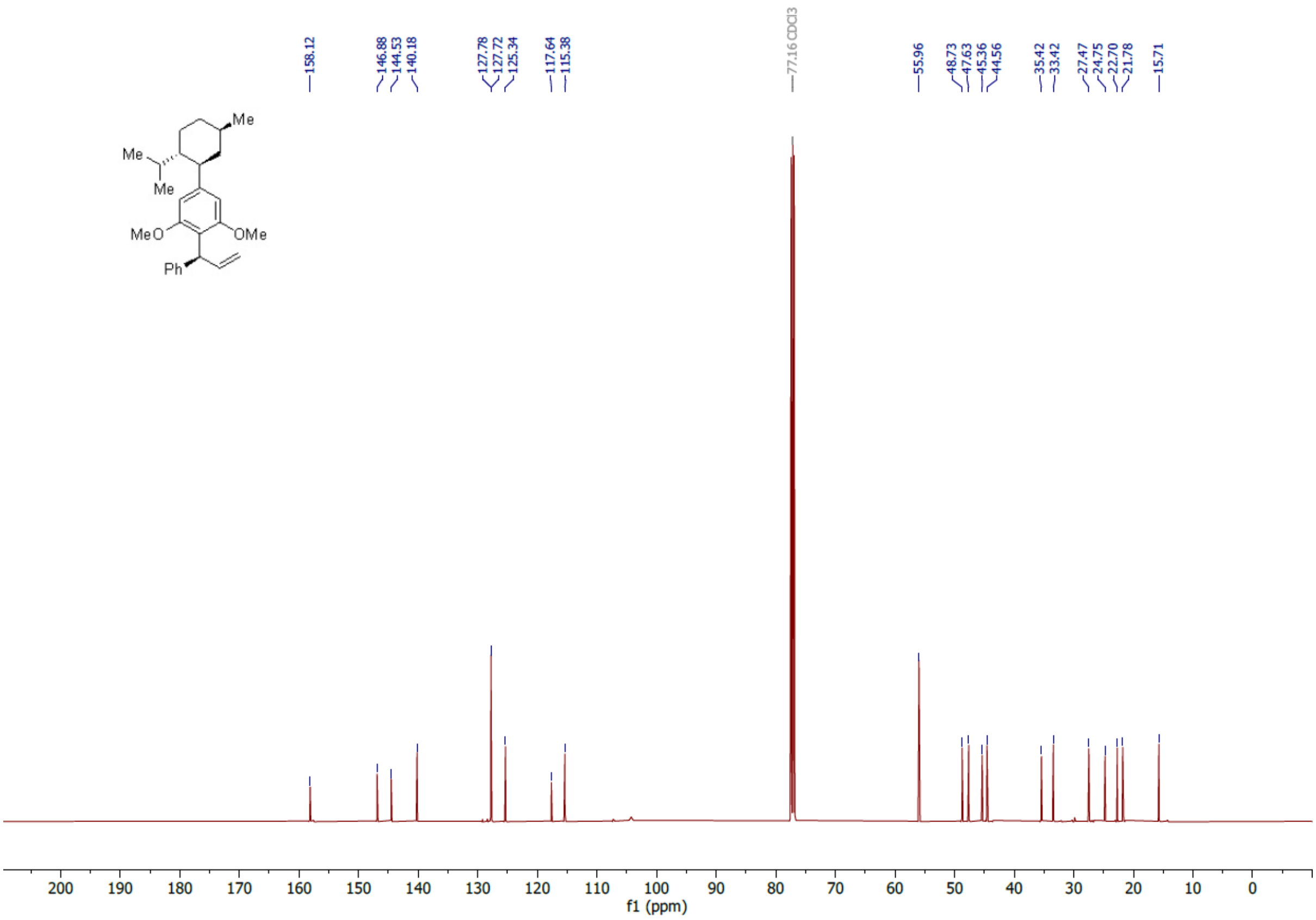

^{1}H NMR (500 MHz, $CDCl_3$) of **5s**

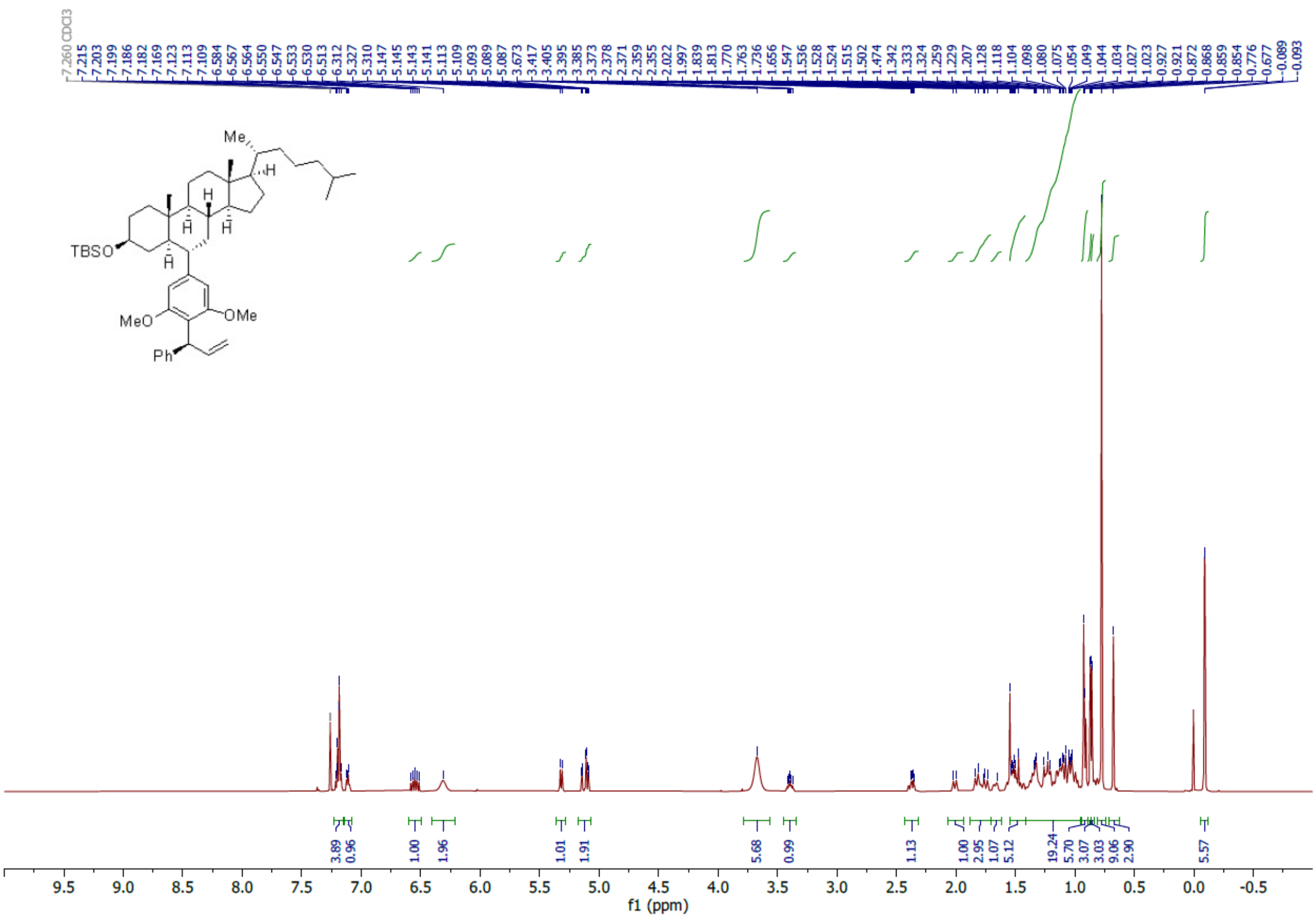

^{13}C NMR (126 MHz, $CDCl_3$) of **5s**

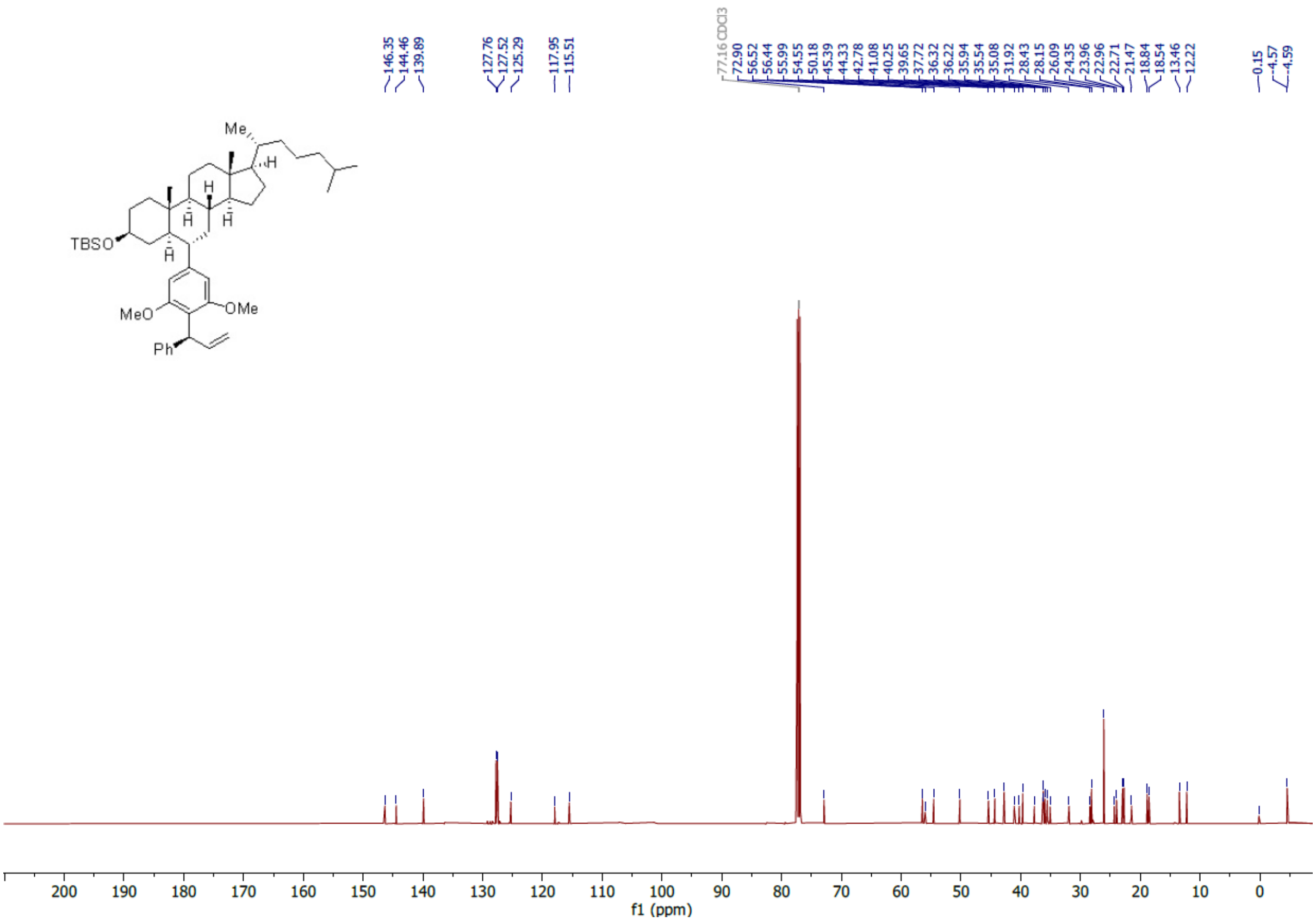

^{1}H NMR (500 MHz, $CDCl_3$) of **5t**

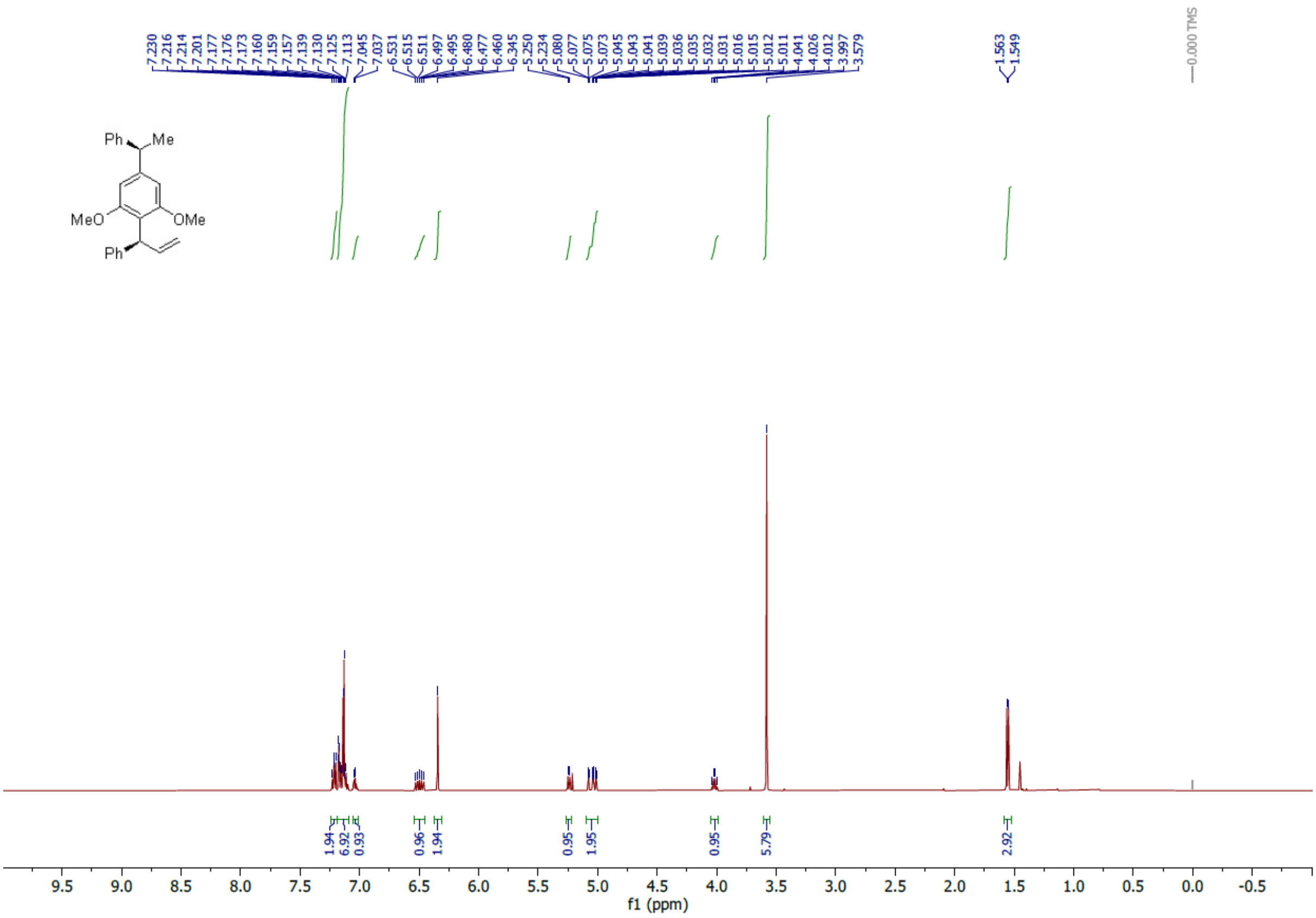

^{13}C NMR (126 MHz, $CDCl_3$) of **5t**

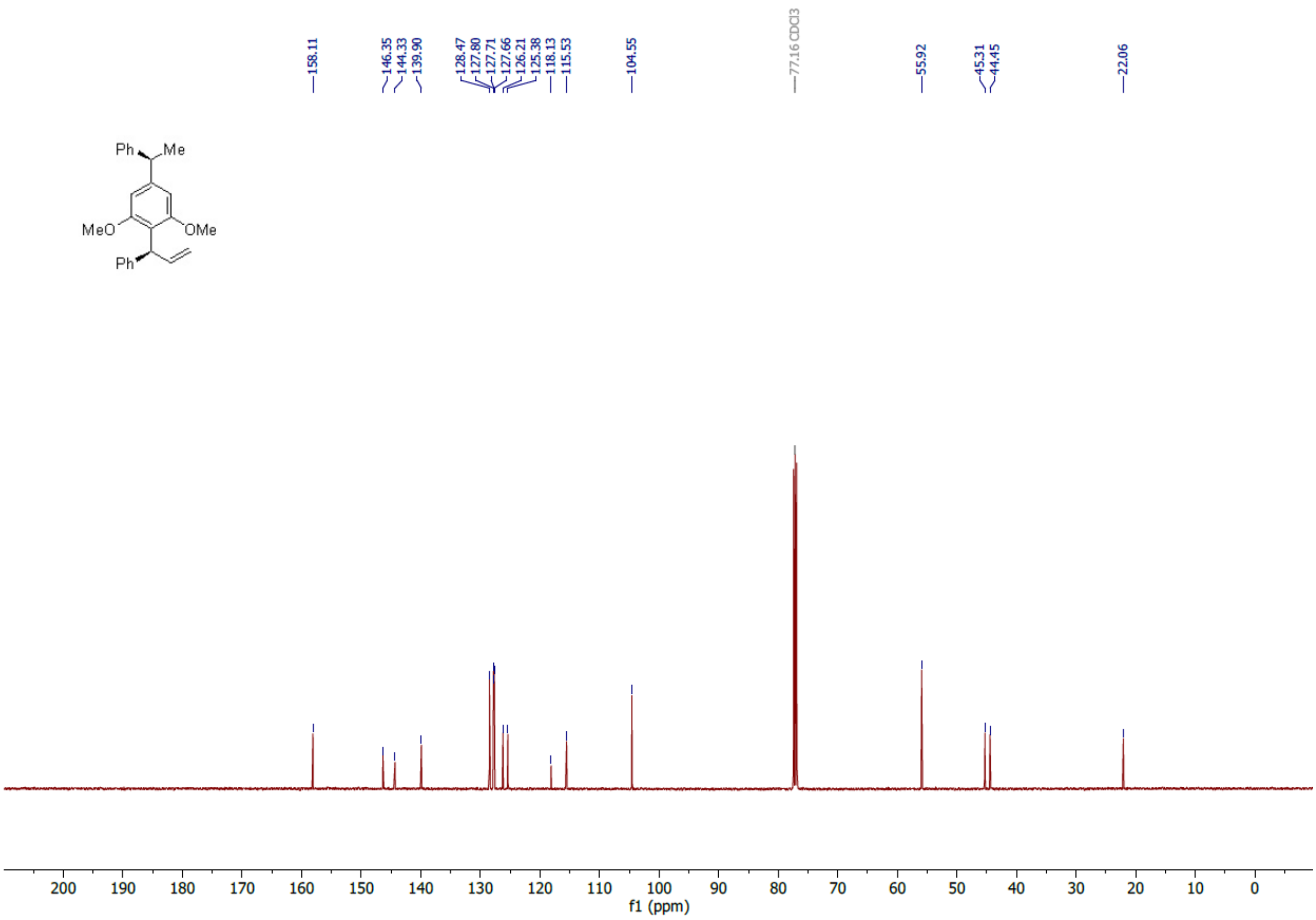

^{1}H NMR (500 MHz, $CDCl_3$) of **5t'**

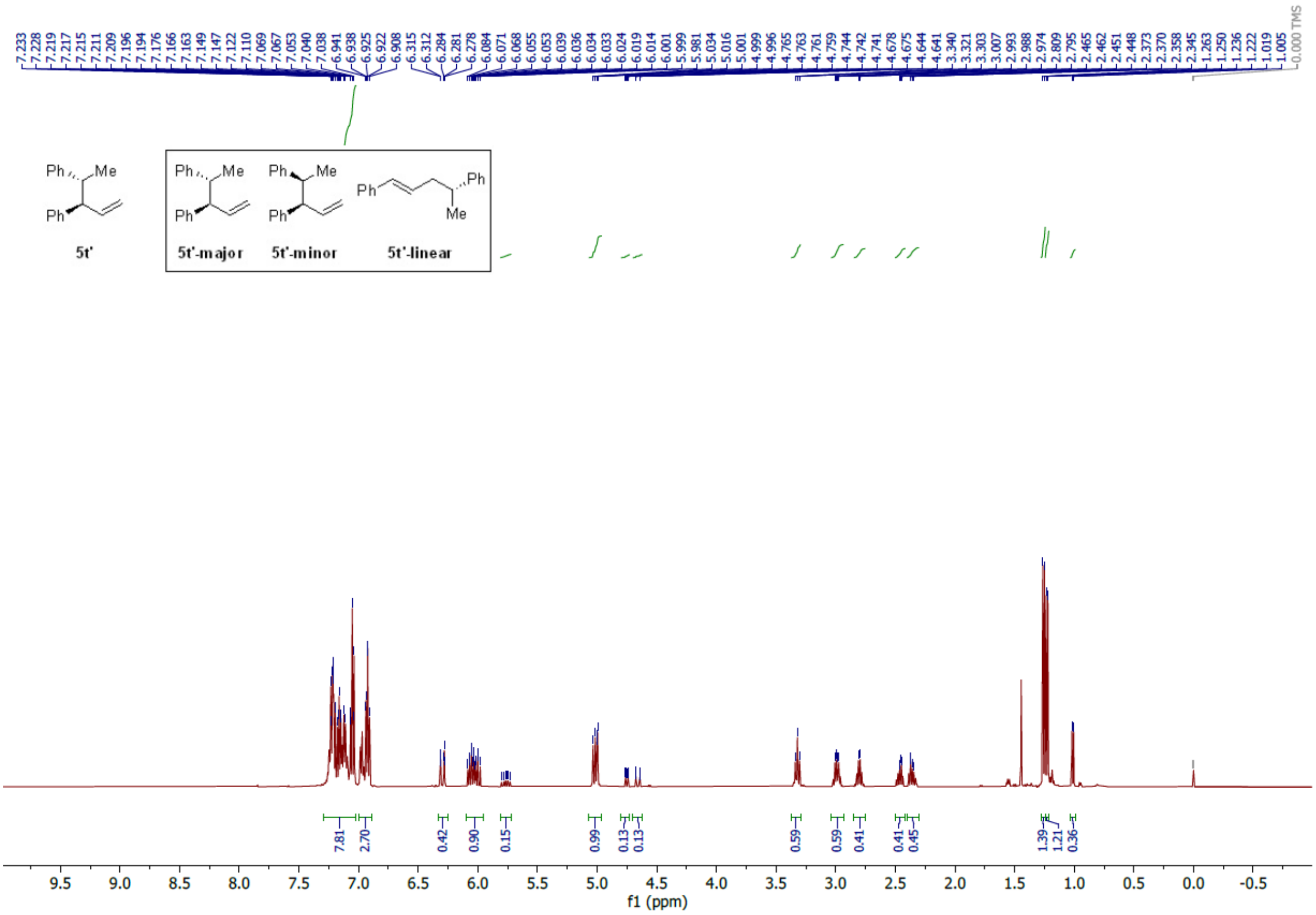

^{13}C NMR (126 MHz, $CDCl_3$) of **5t'**

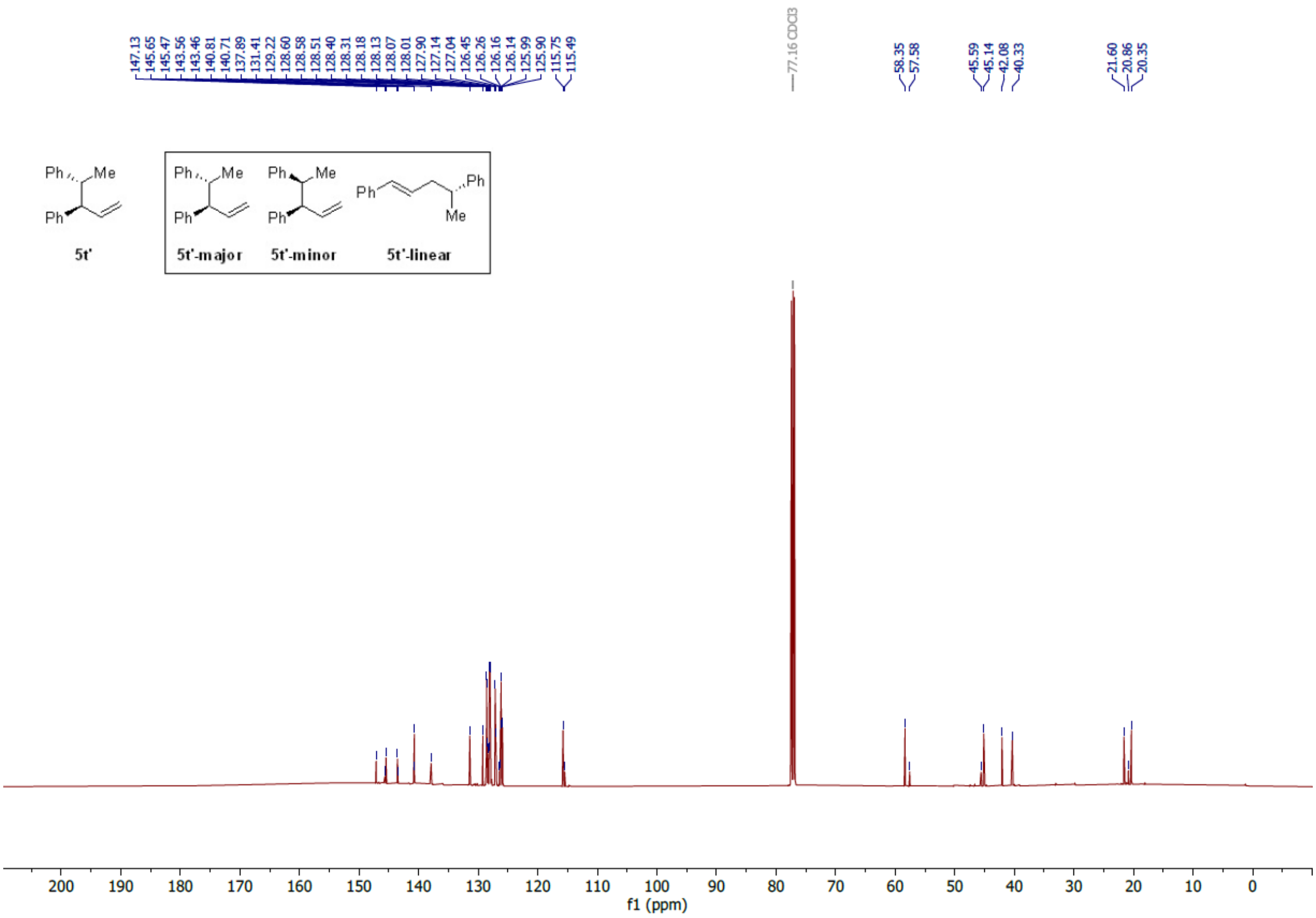

^{1}H NMR (400 MHz, $CDCl_3$) of **5u**

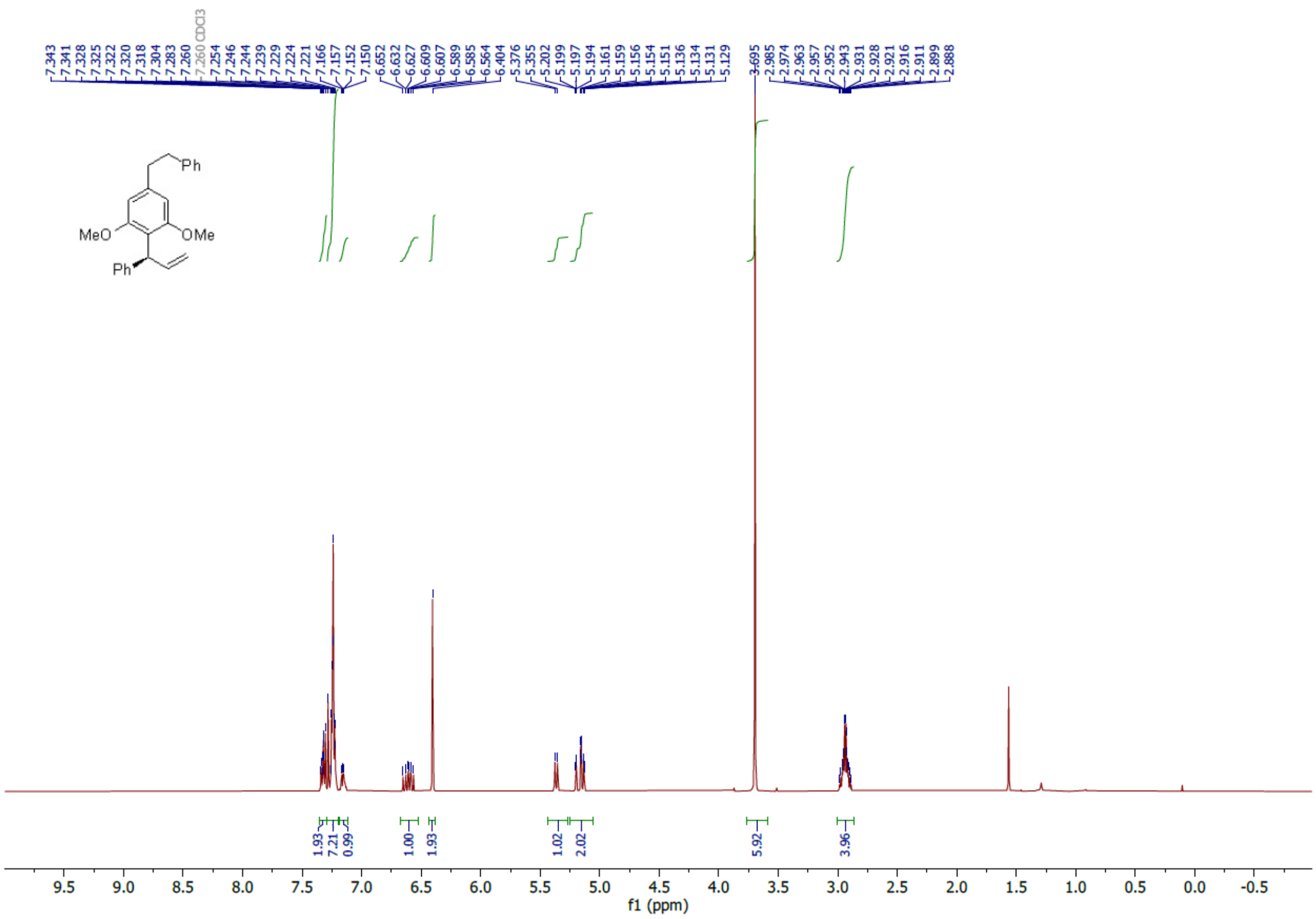

^{13}C NMR (101 MHz, $CDCl_3$) of **5u**

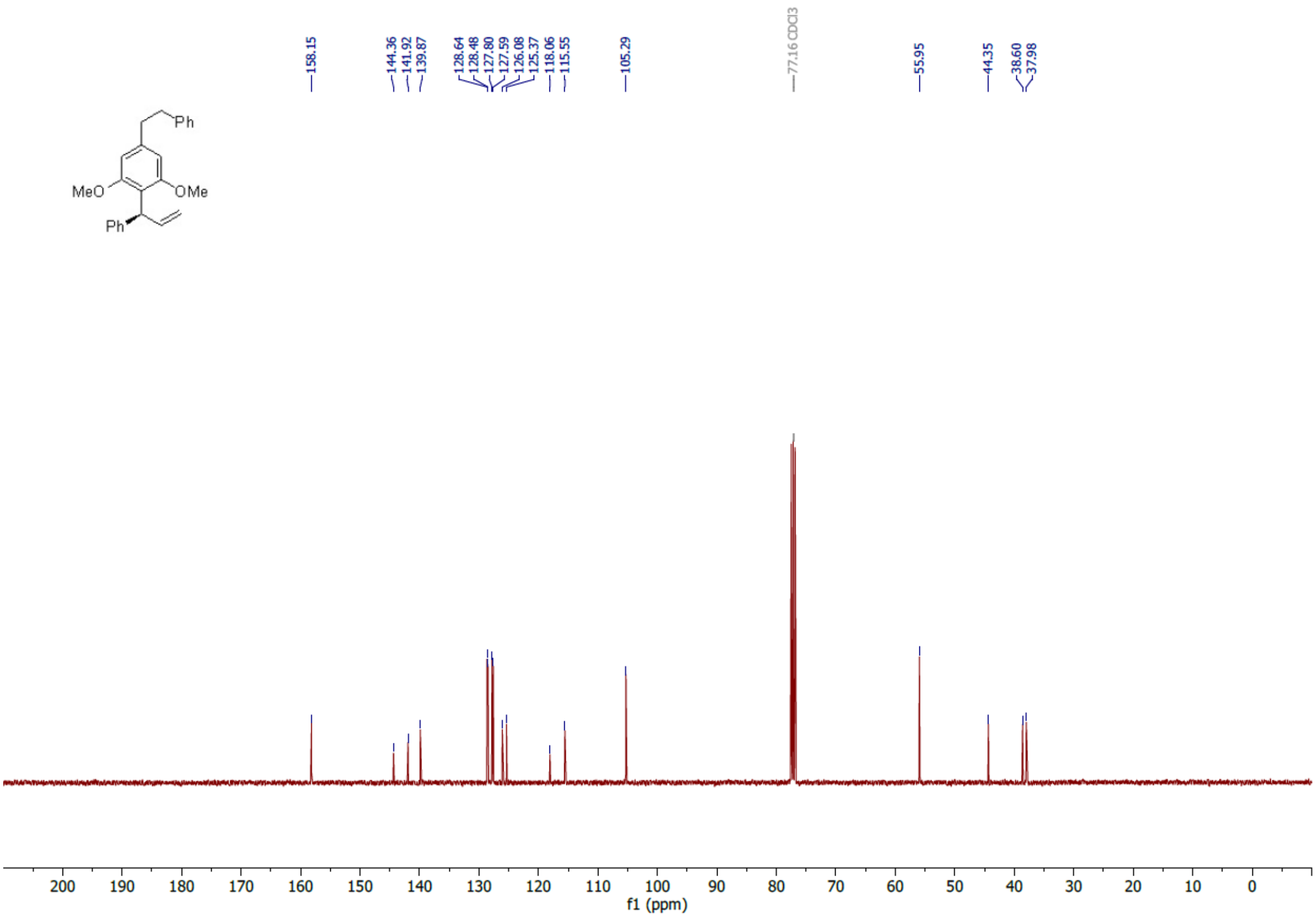

^{1}H NMR (500 MHz, $CDCl_3$) of **5v**

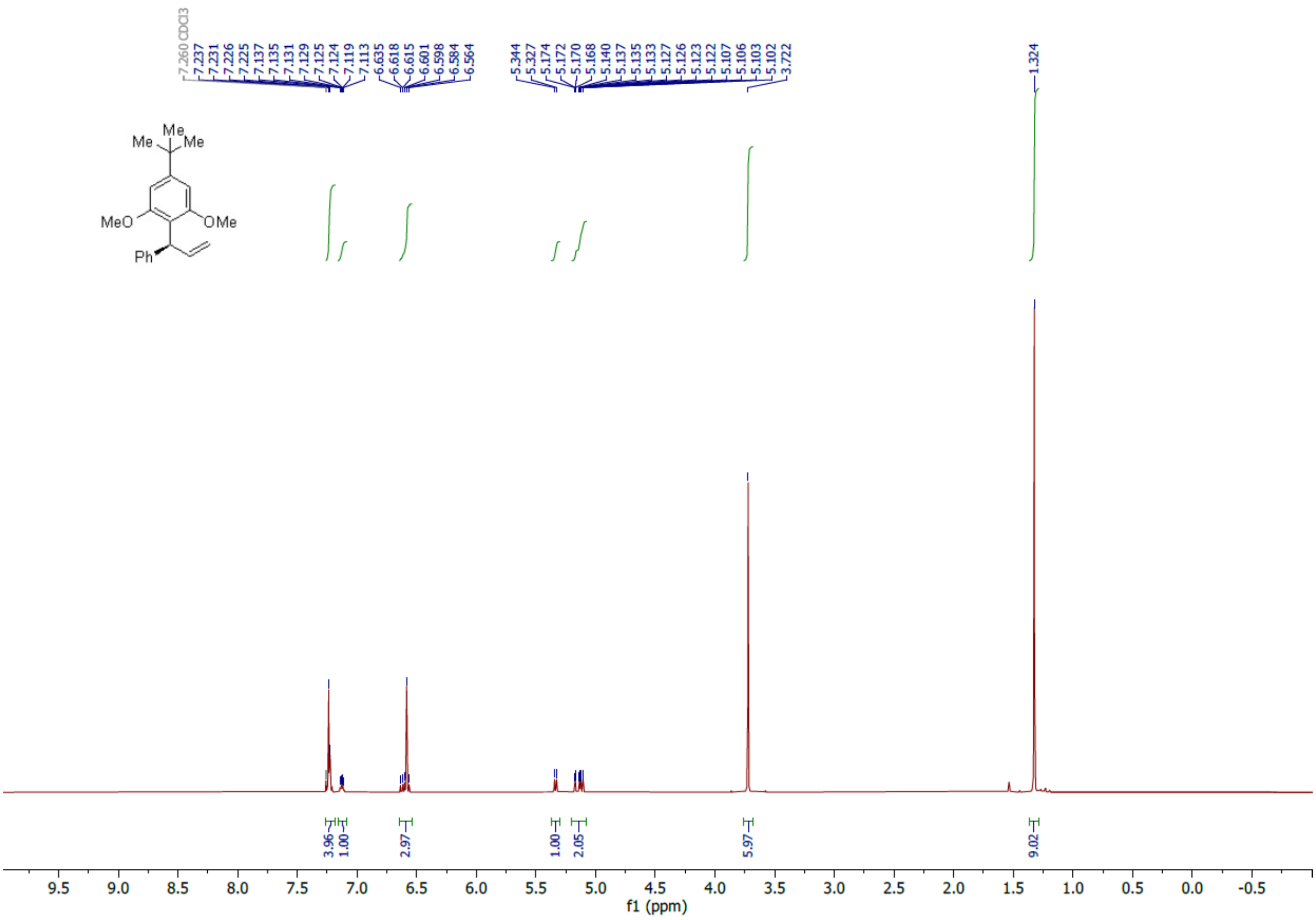

^{13}C NMR (126 MHz, $CDCl_3$) of **5v**

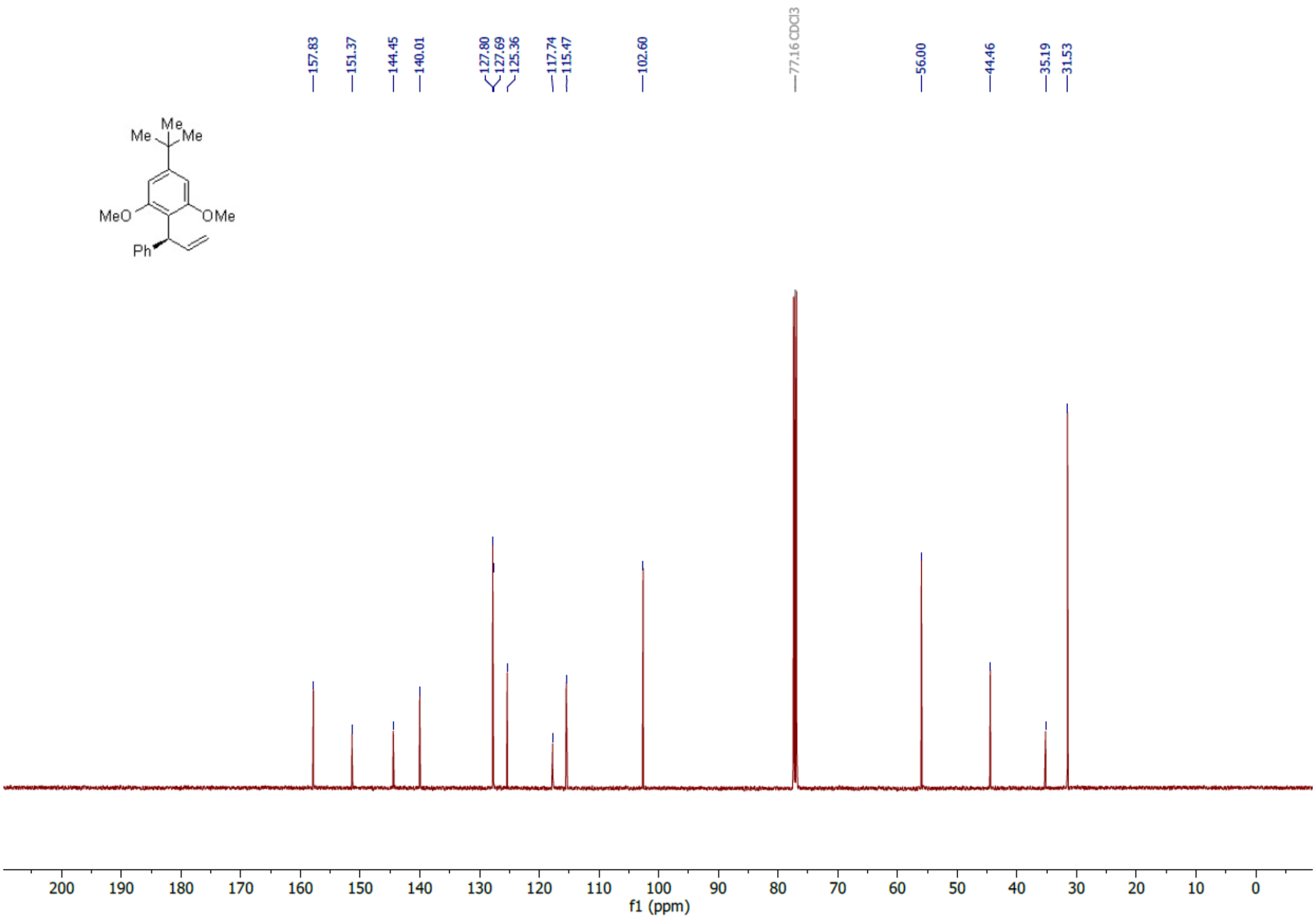

^{1}H NMR (500 MHz, $CDCl_3$) of **5w**

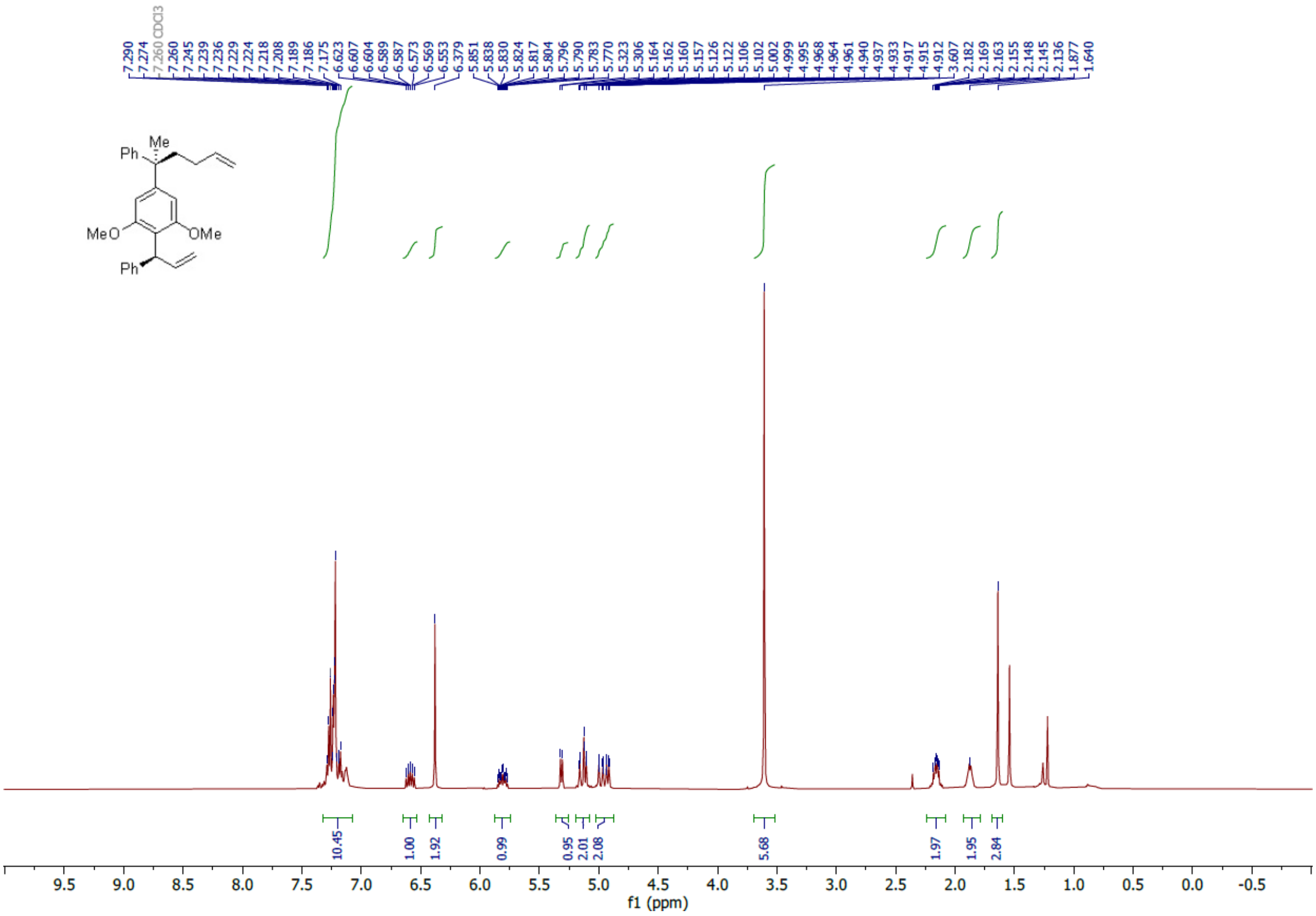

^{13}C NMR (126 MHz, $CDCl_3$) of **5w**

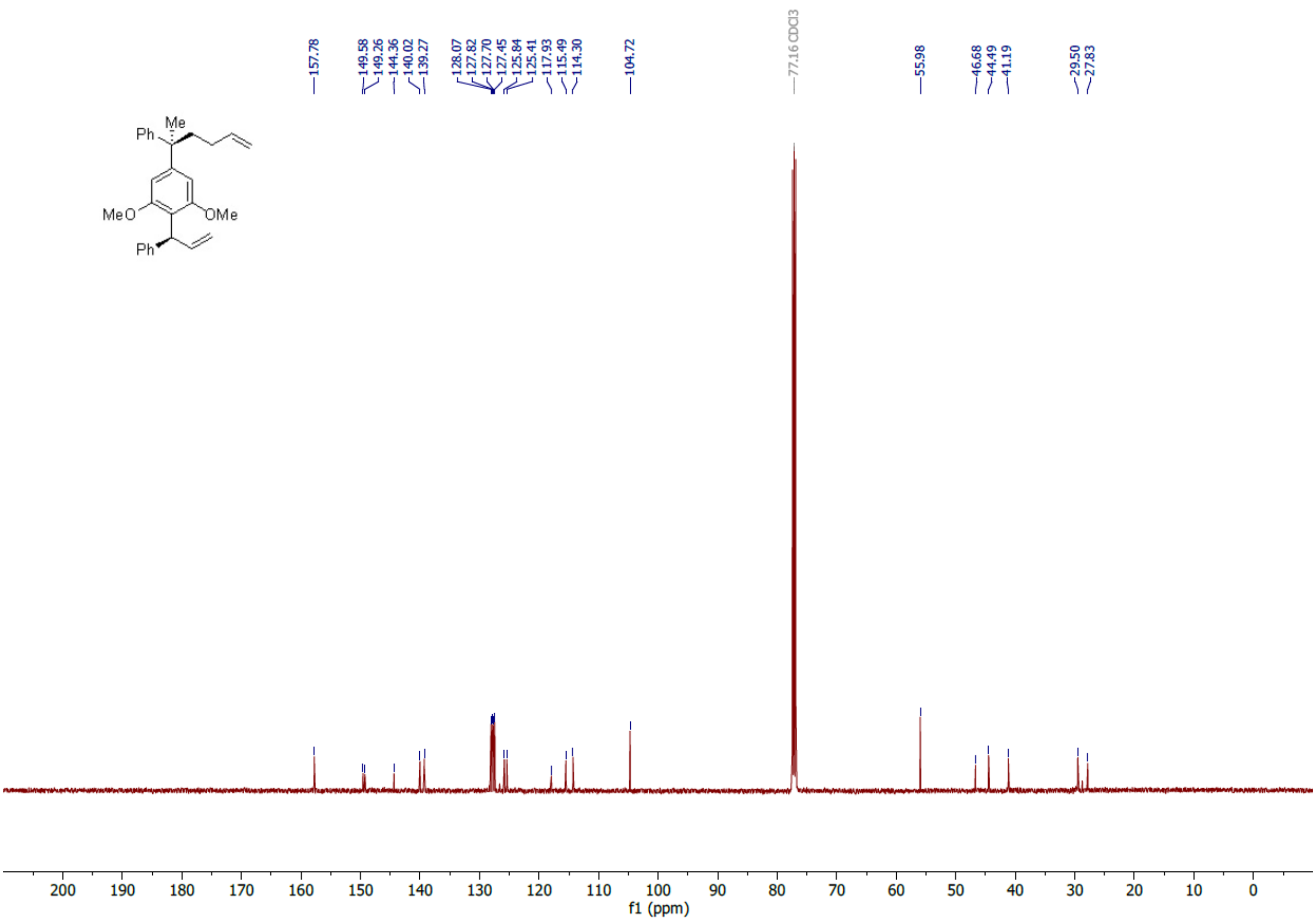

^{1}H NMR (400 MHz, $CDCl_3$) of **5y**

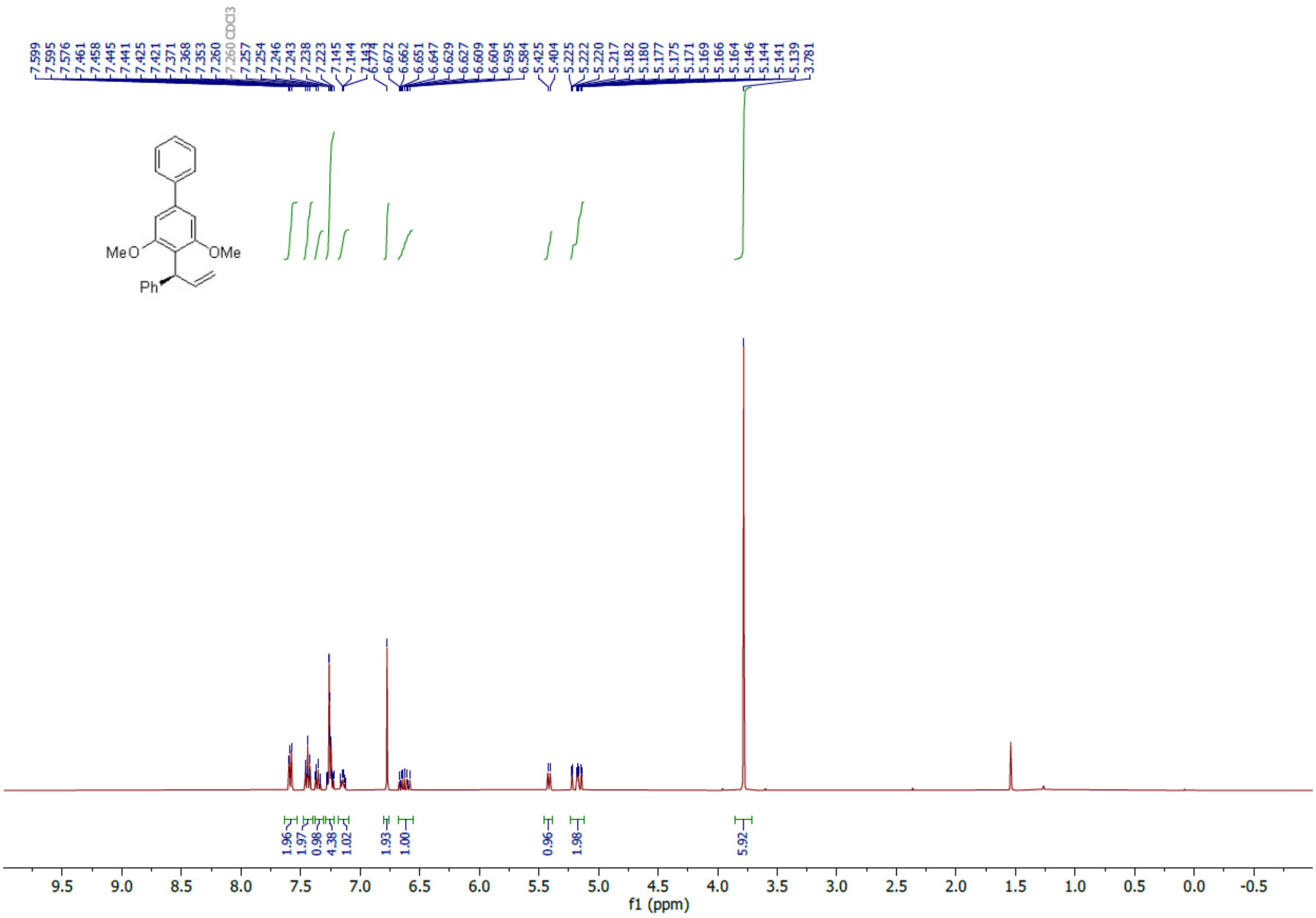

^{13}C NMR (101 MHz, $CDCl_3$) of **5y**

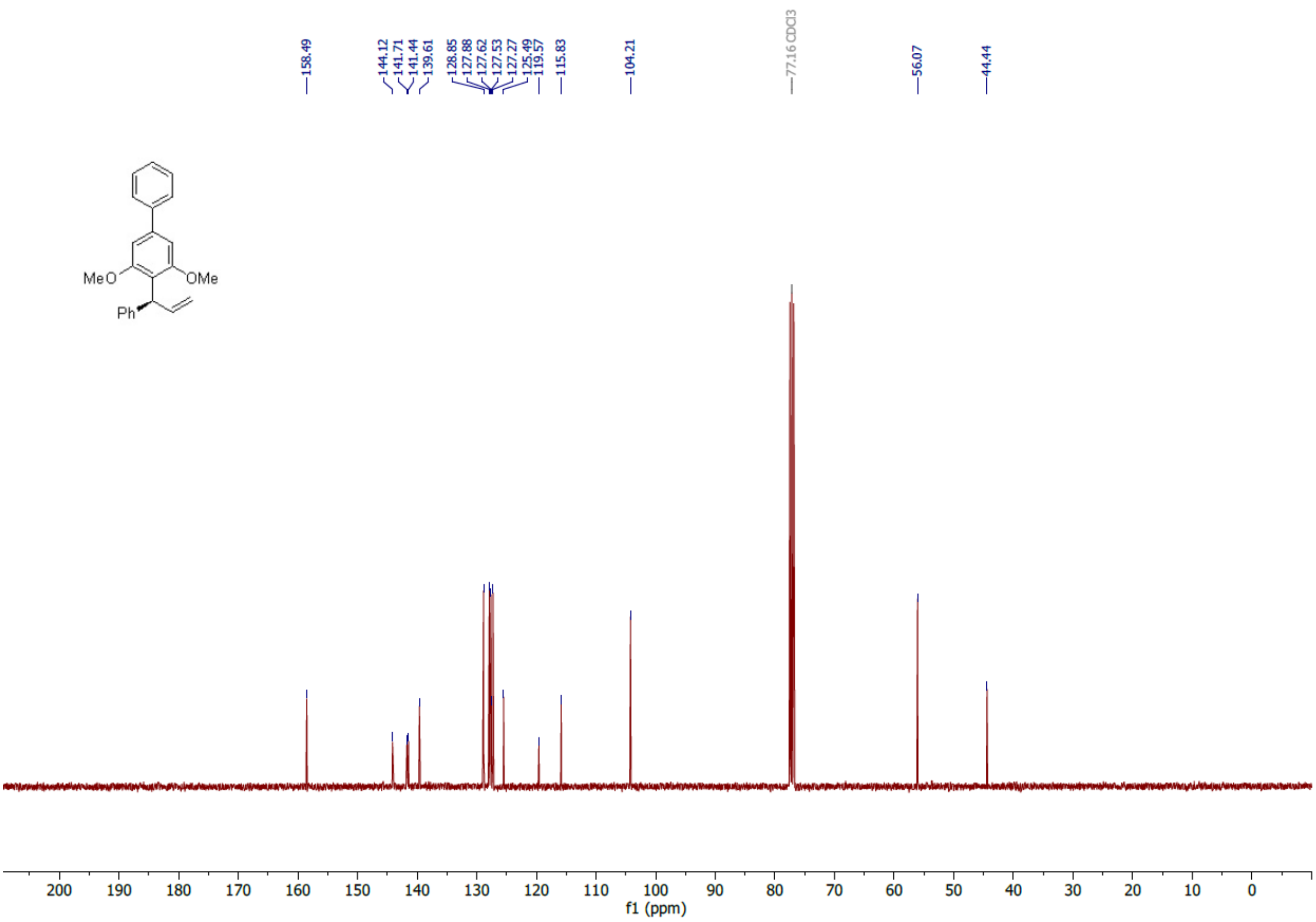

^{1}H NMR (400 MHz, $CDCl_3$) of **14**

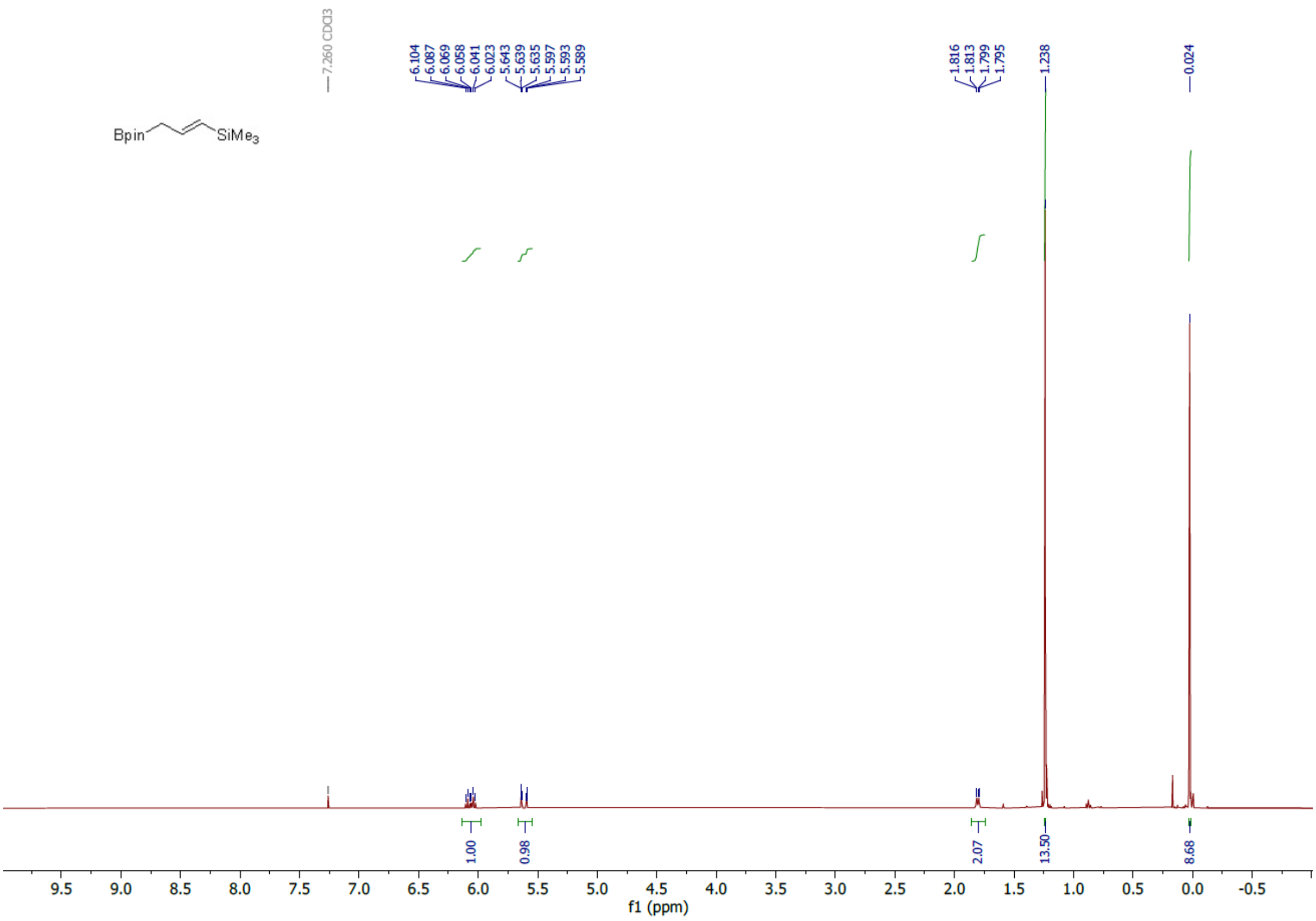

^{13}C NMR (101 MHz, $CDCl_3$) of **14**

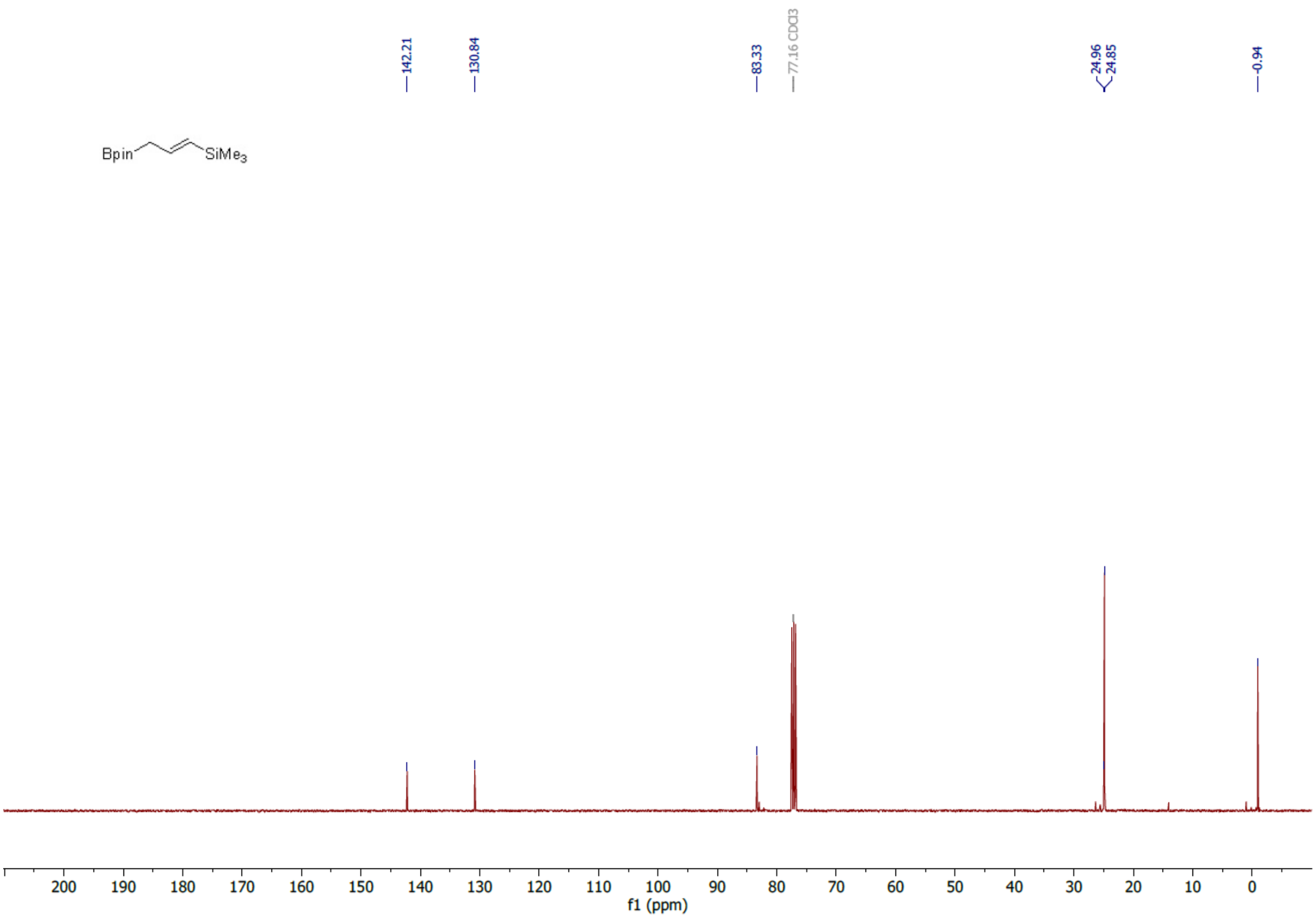

^{1}H NMR (400 MHz, $CDCl_3$) of **15**

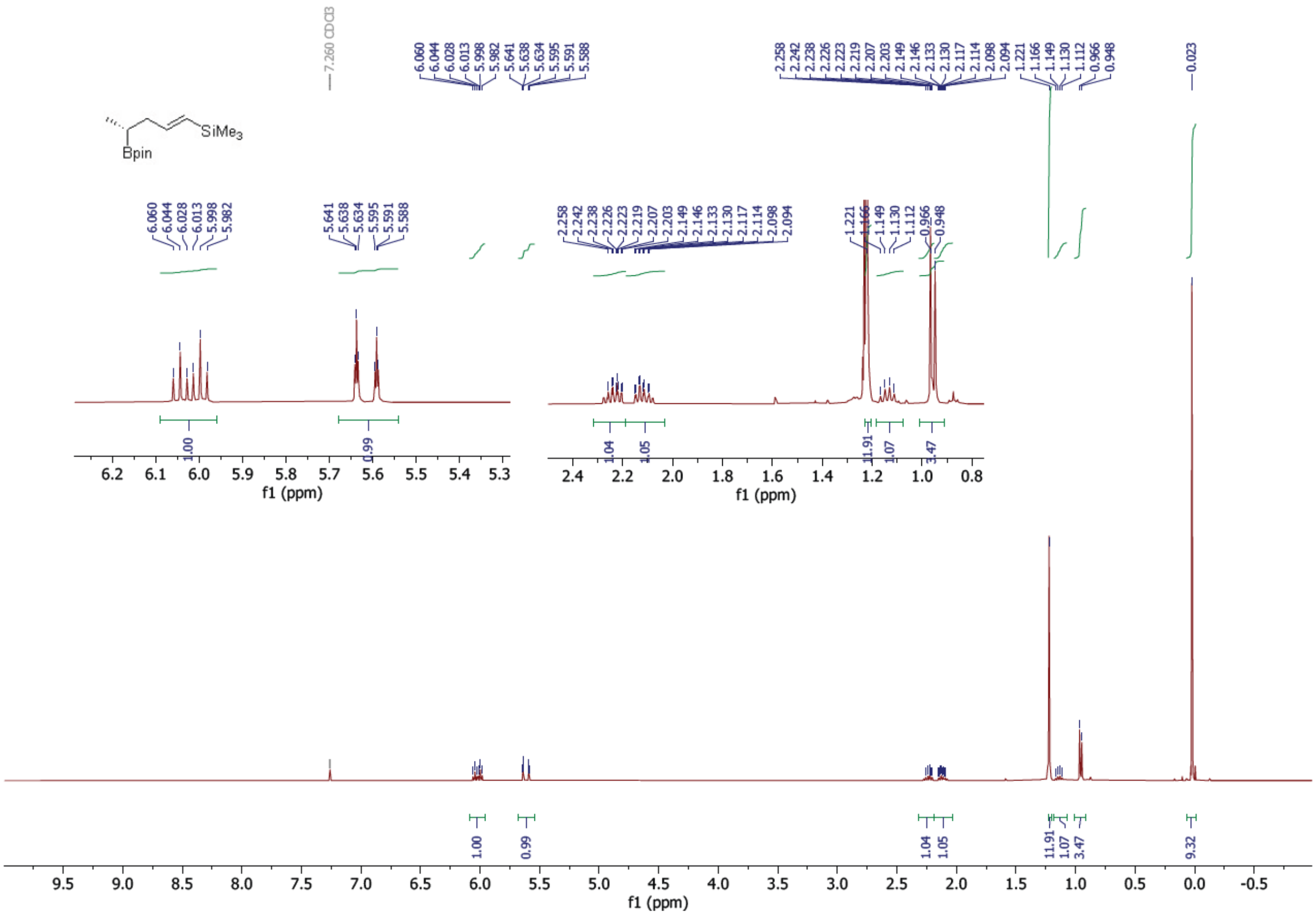

^{13}C NMR (101 MHz, $CDCl_3$) of **15**

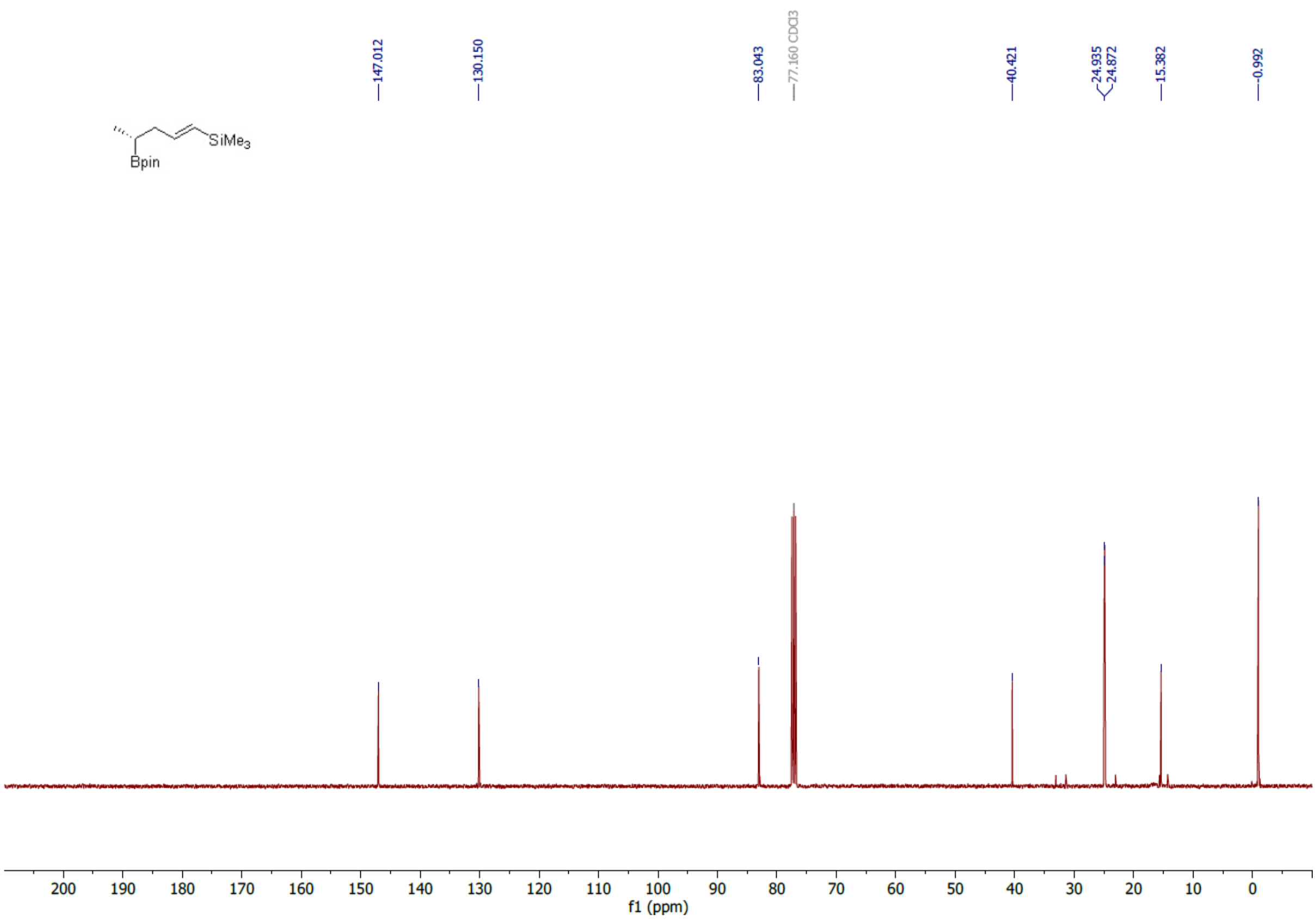

^{1}H NMR (500 MHz, $CDCl_3$) of **12c**

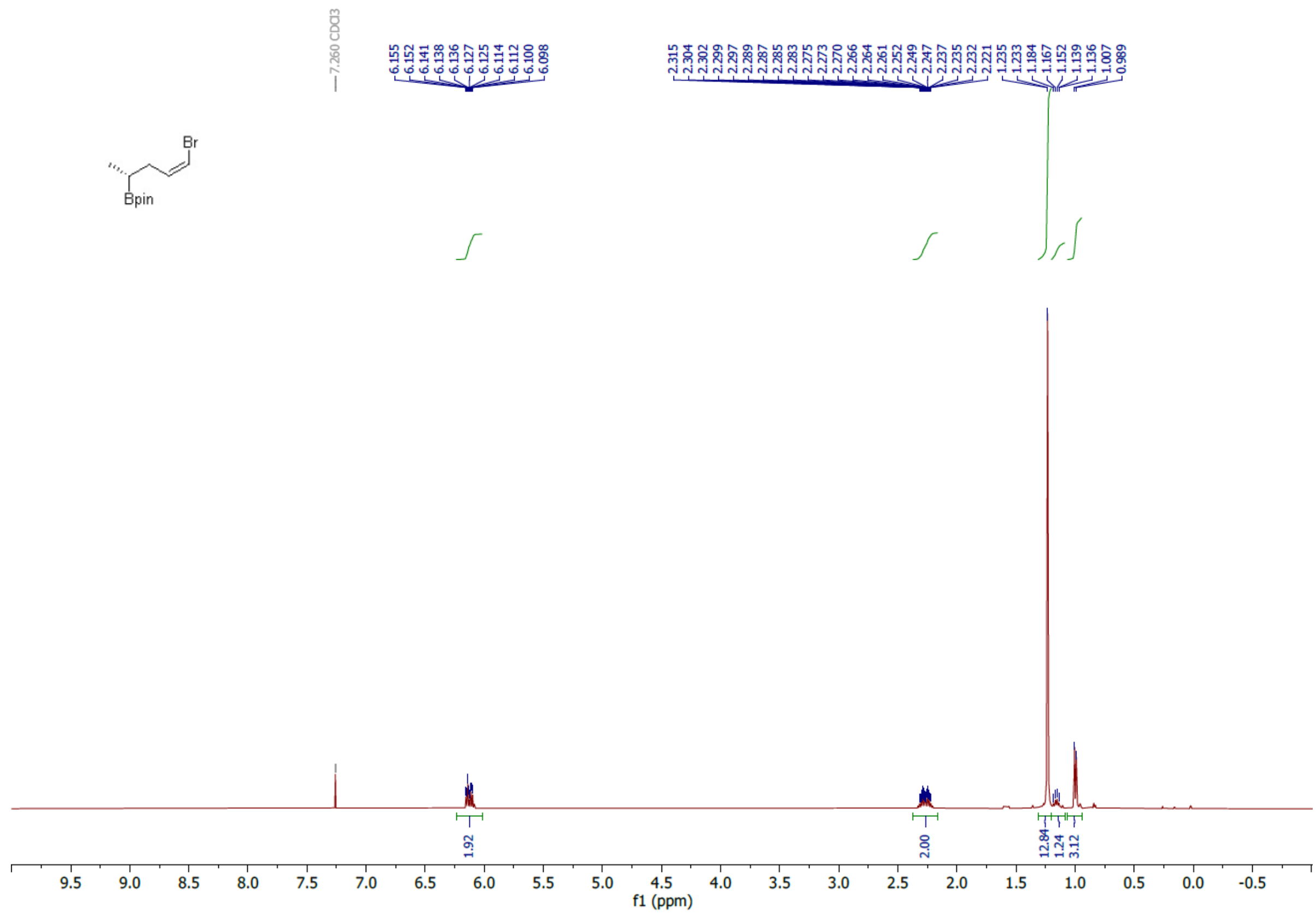

^{13}C NMR (126 MHz, $CDCl_3$) of **12c**

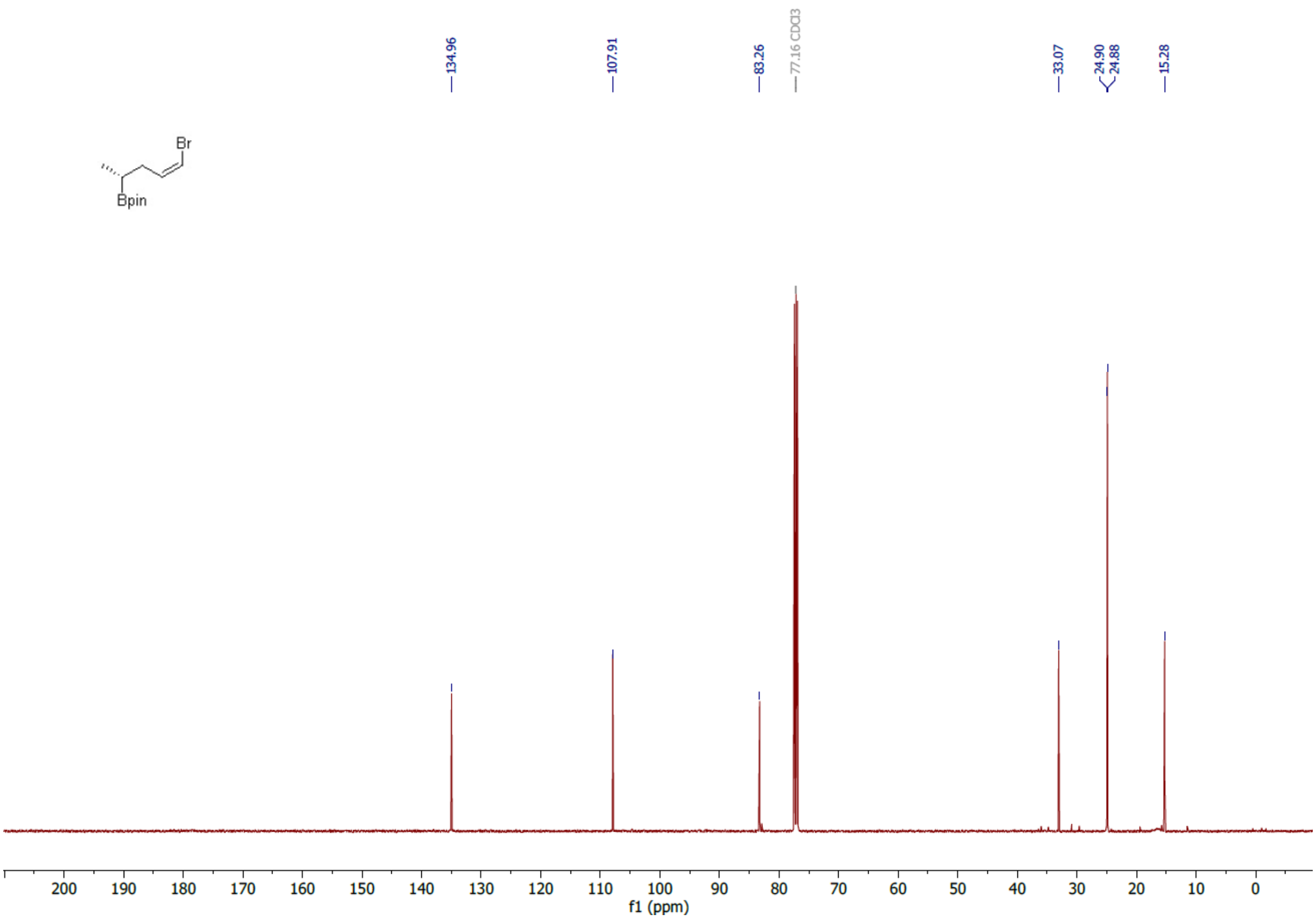

^{1}H NMR (500 MHz, $CDCl_3$) of **11c**

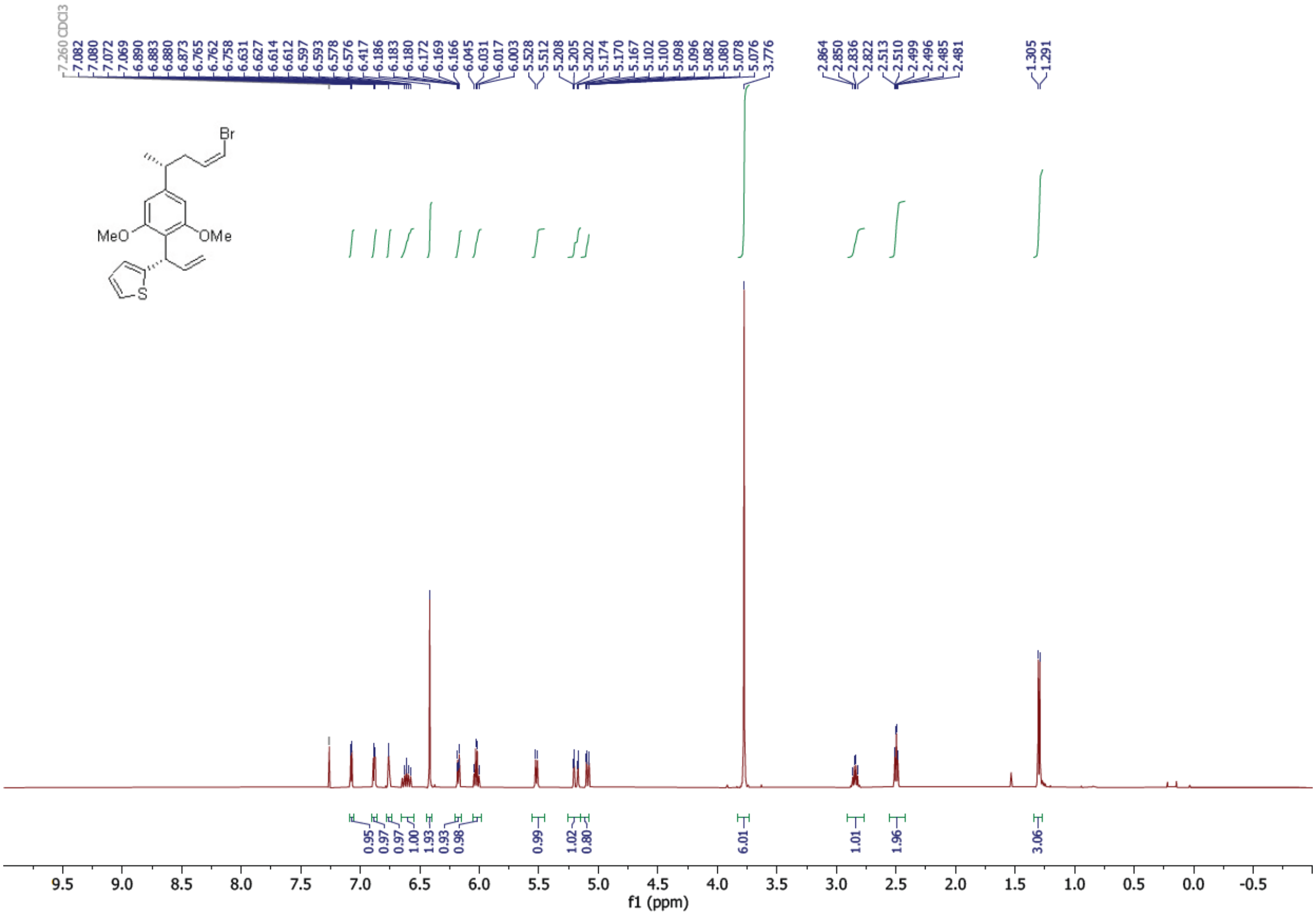

^{13}C NMR (126 MHz, $CDCl_3$) of **11c**

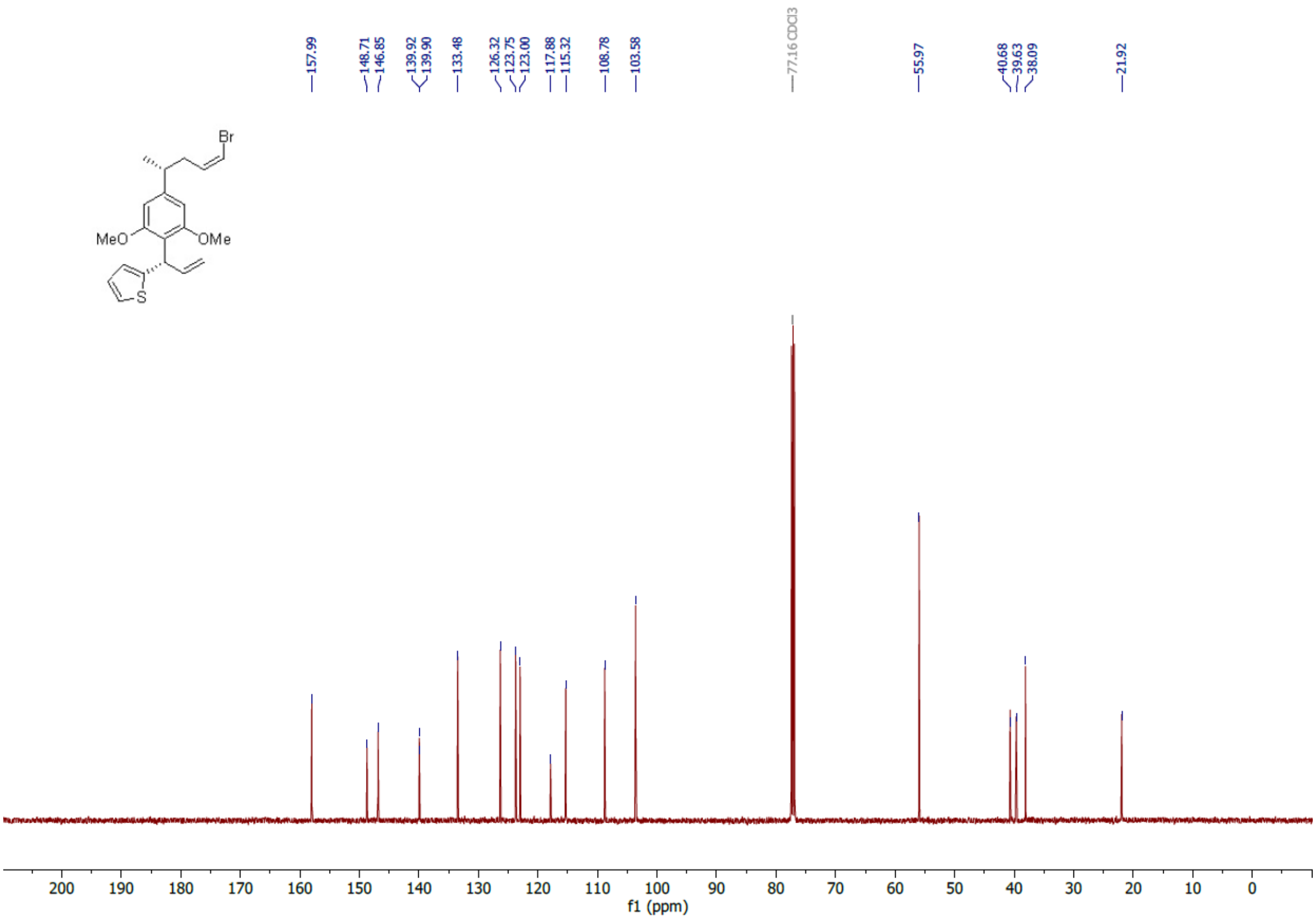

^{1}H NMR (500 MHz, $CDCl_3$) of **10**

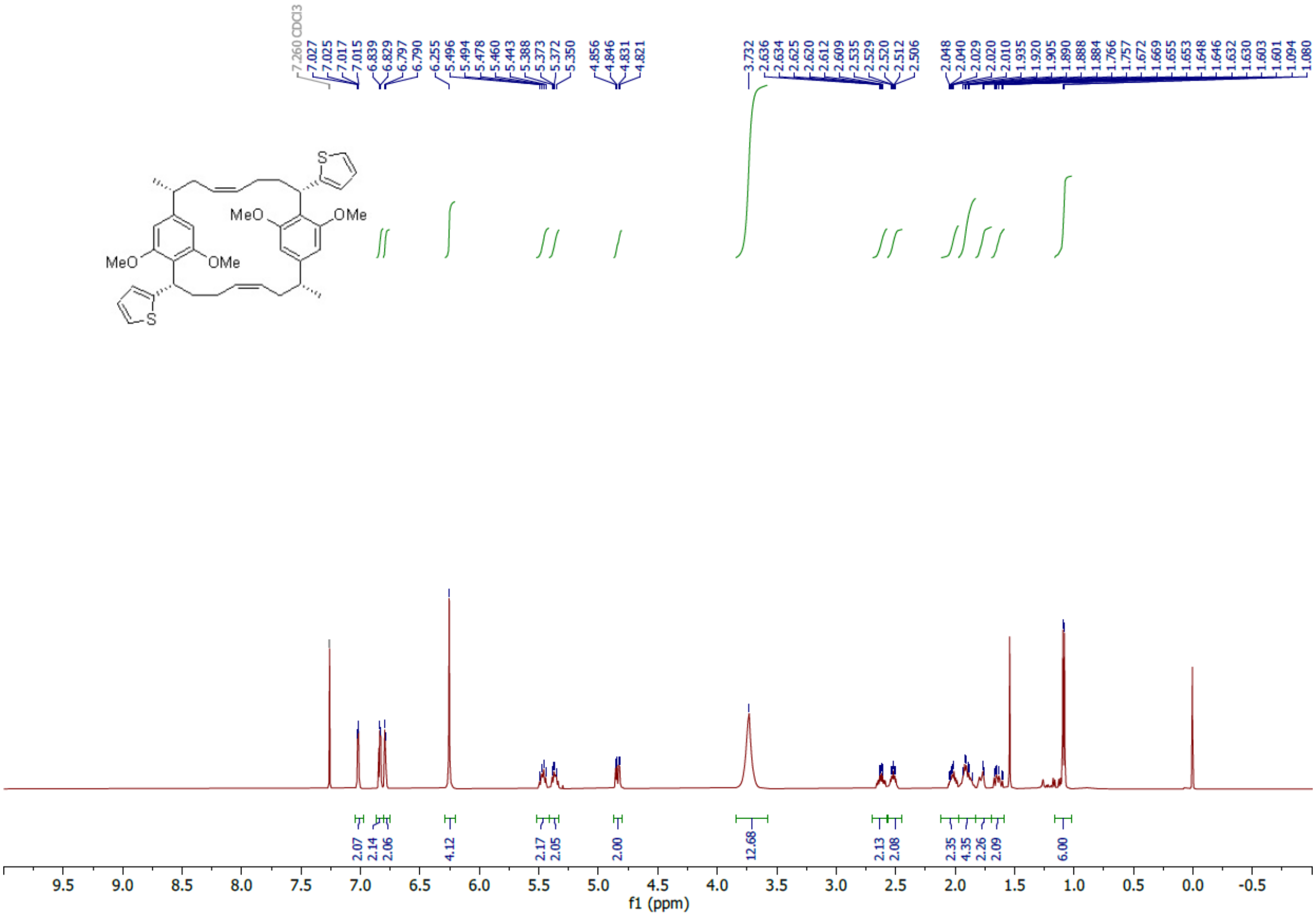

^{13}C NMR (126 MHz, $CDCl_3$) of **10**

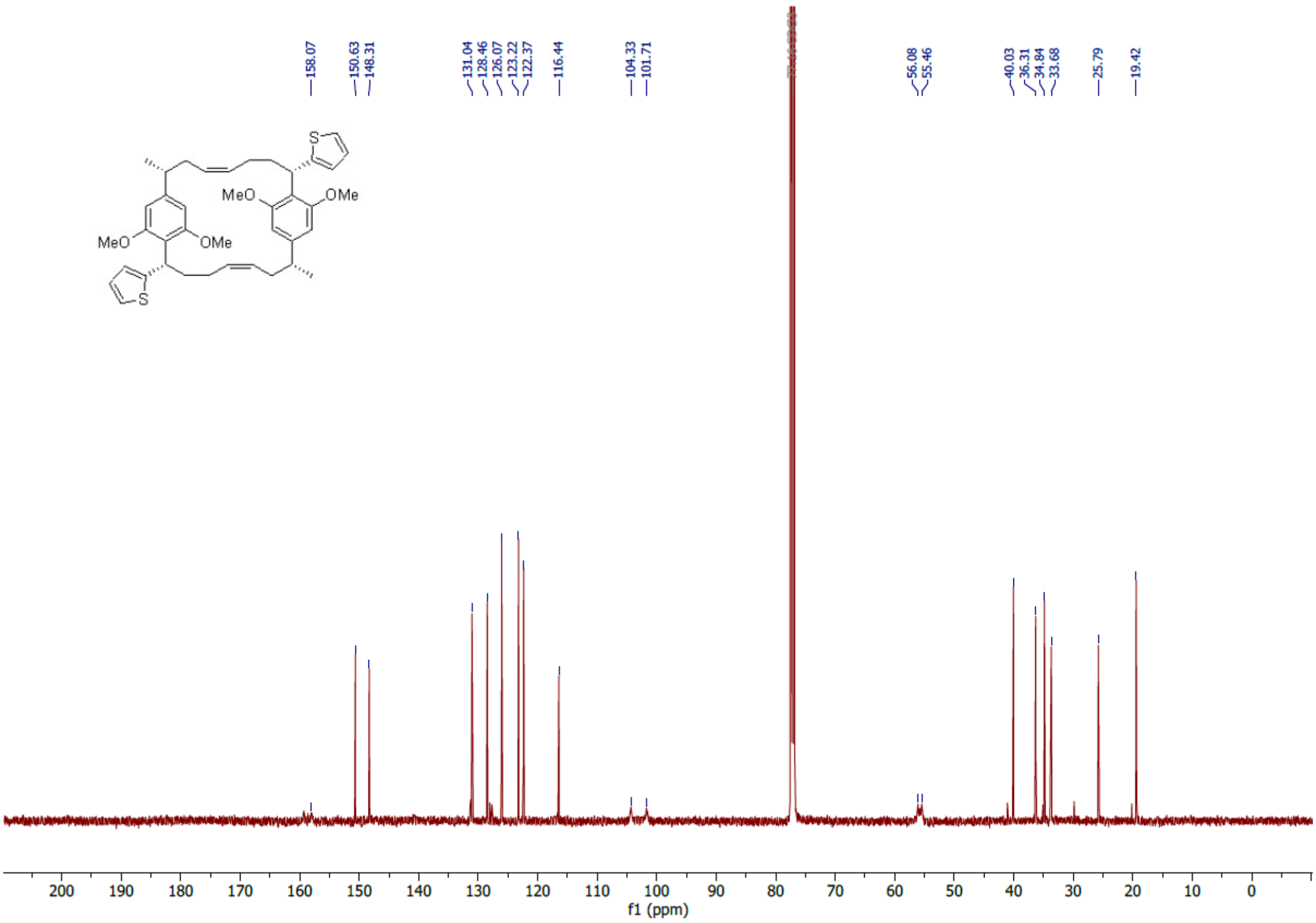

HSQC (500 MHz, $CDCl_3$) of **10**

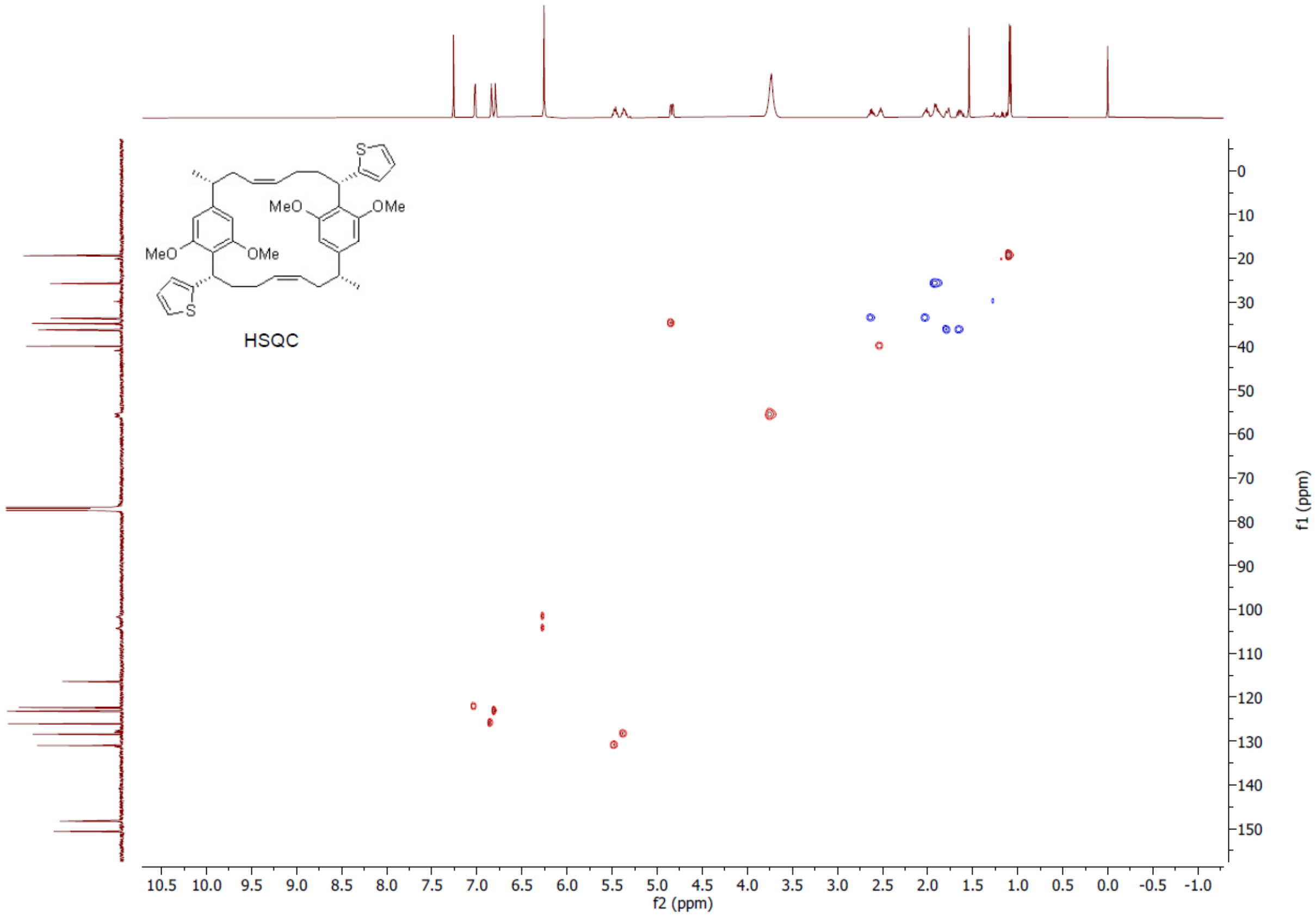

HMBC (500 MHz, $CDCl_3$) of **10**

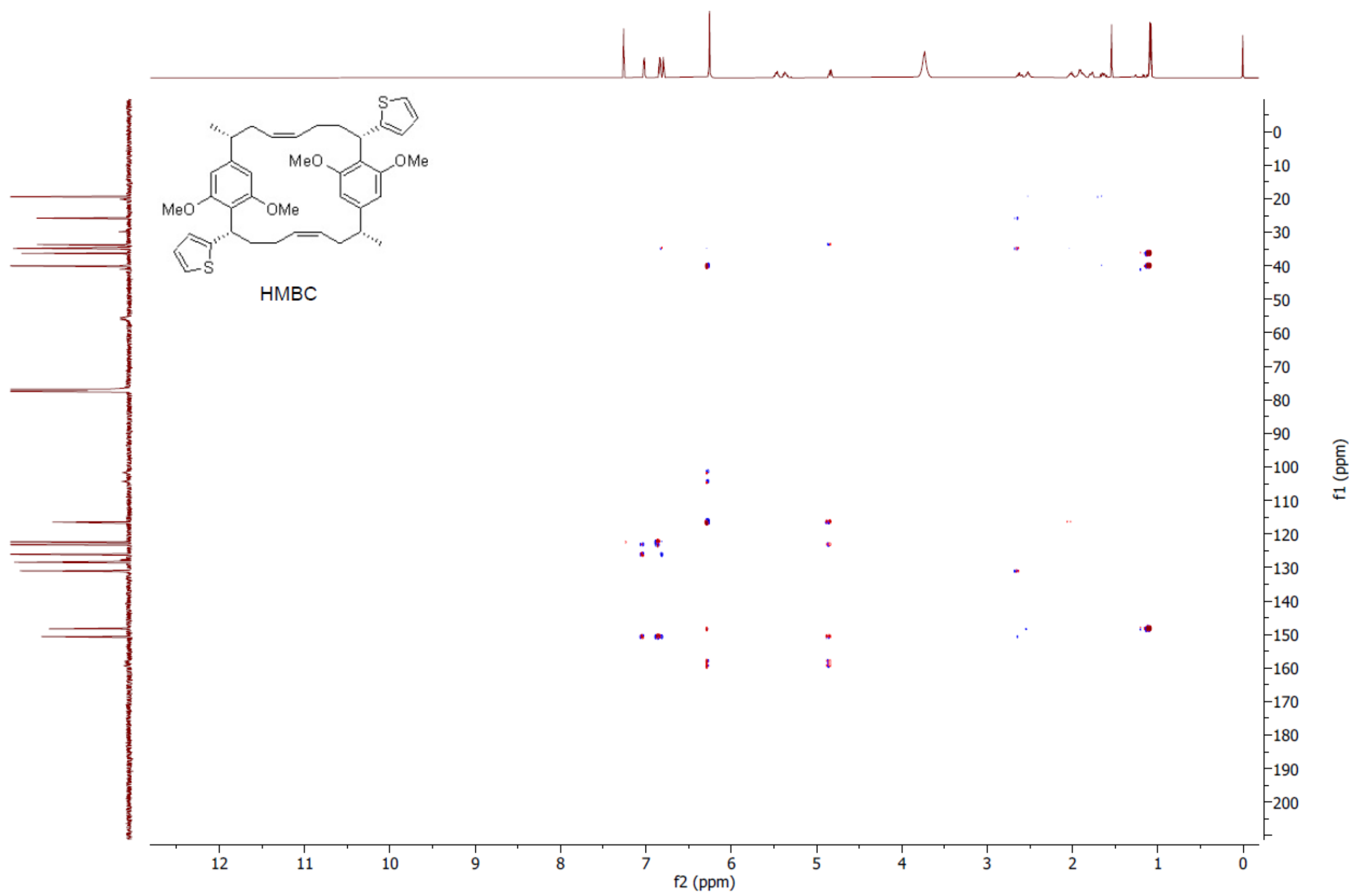

^{1}H NMR (500 MHz, CD_3OD) of **6** (merocyclophane A)

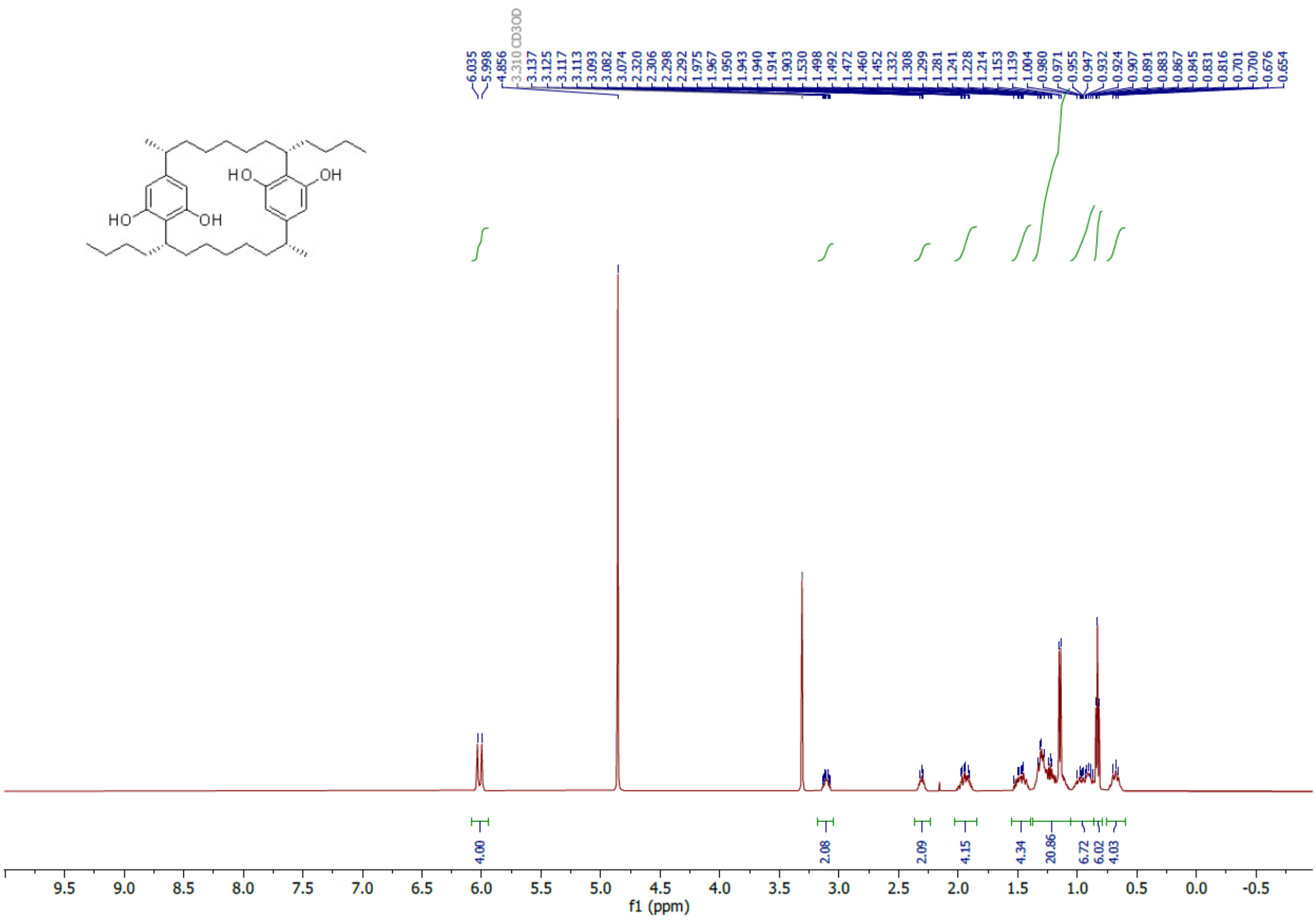

^{13}C NMR (126 MHz, CD_3OD) of **6**

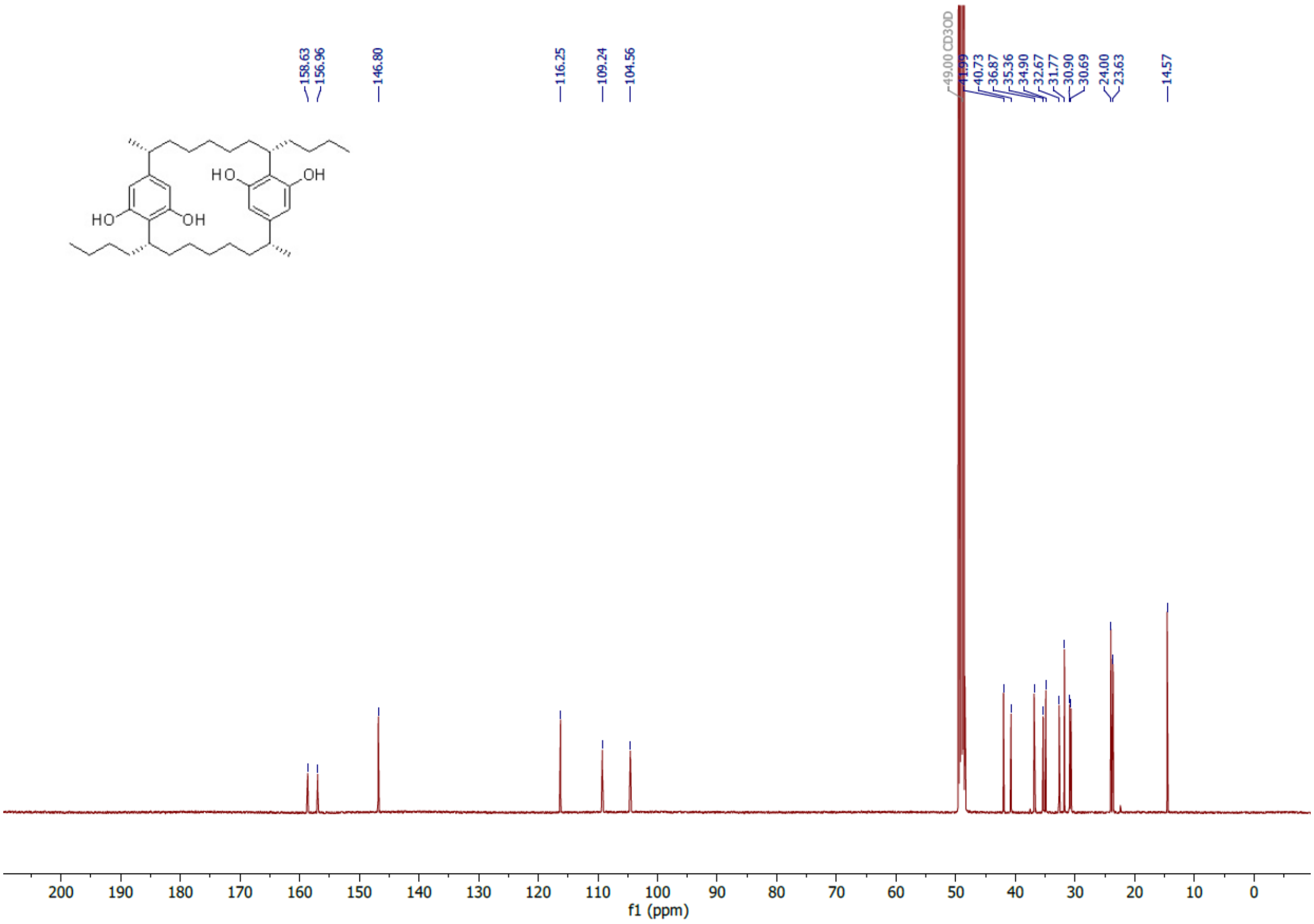

^{1}H NMR (500 MHz, $CDCl_3$) of **12a**

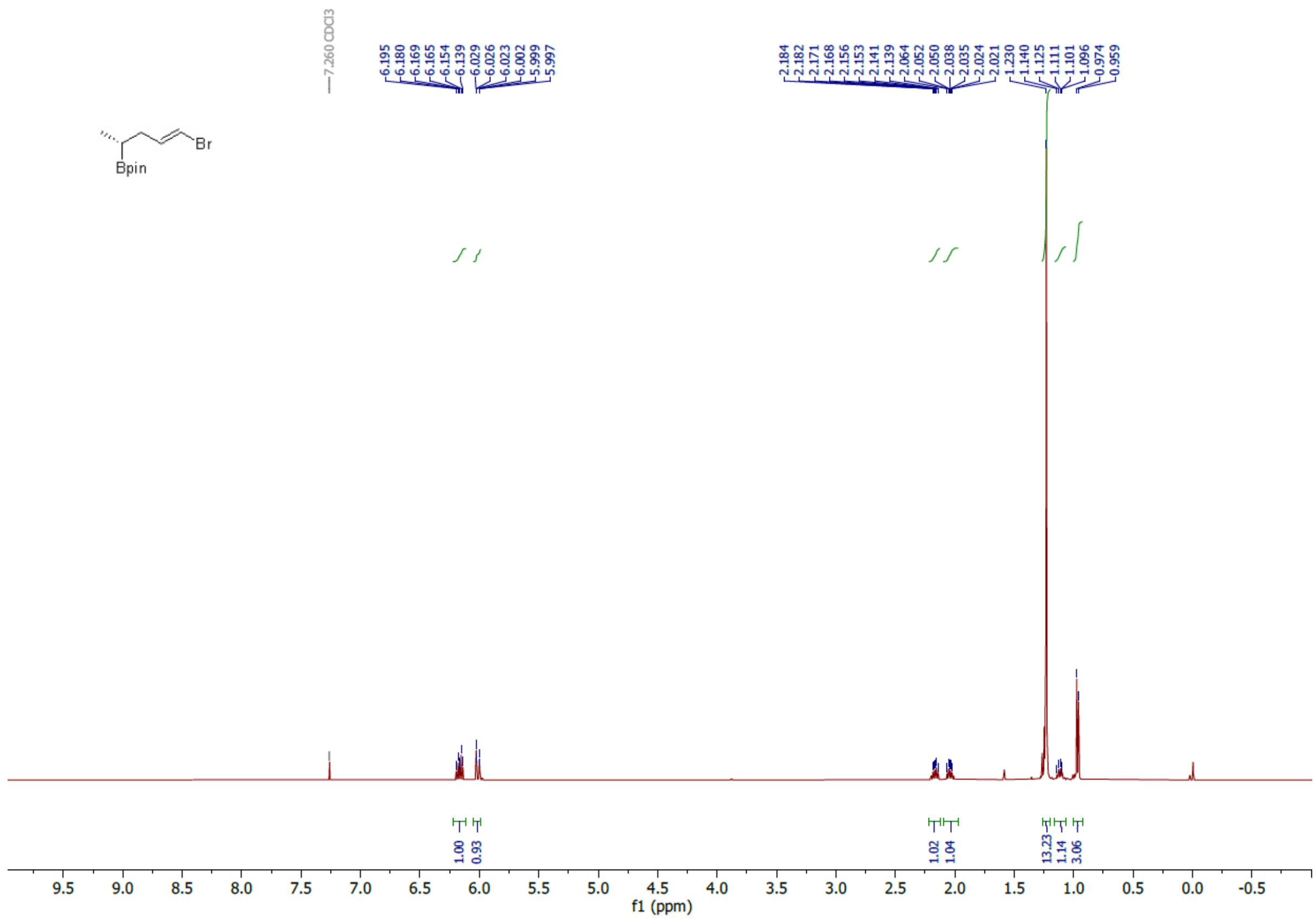

^{13}C NMR (126 MHz, $CDCl_3$) of **12a**

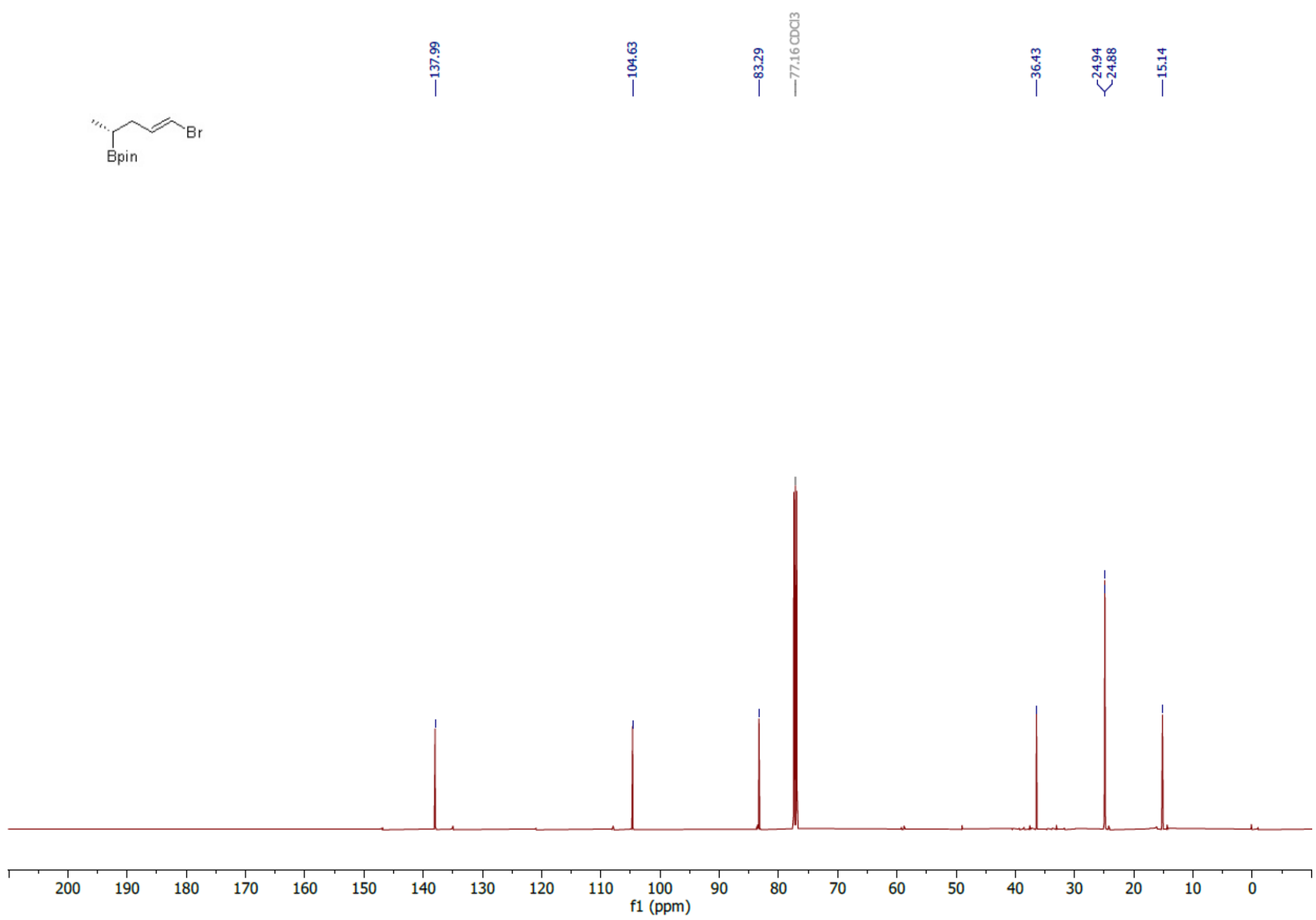

^{1}H NMR (500 MHz, $CDCl_3$) of **11a**

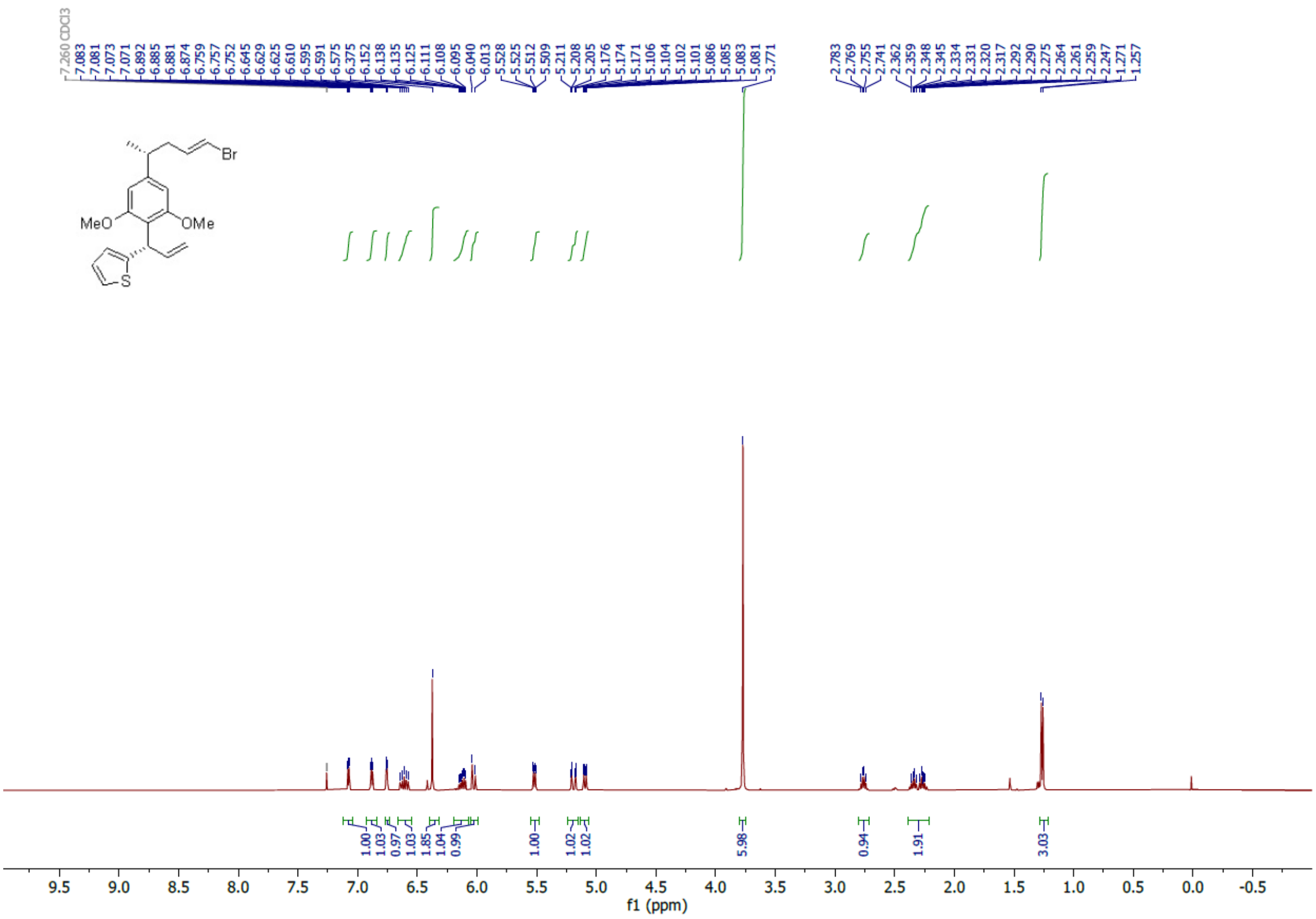

^{13}C NMR (126 MHz, $CDCl_3$) of **11a**

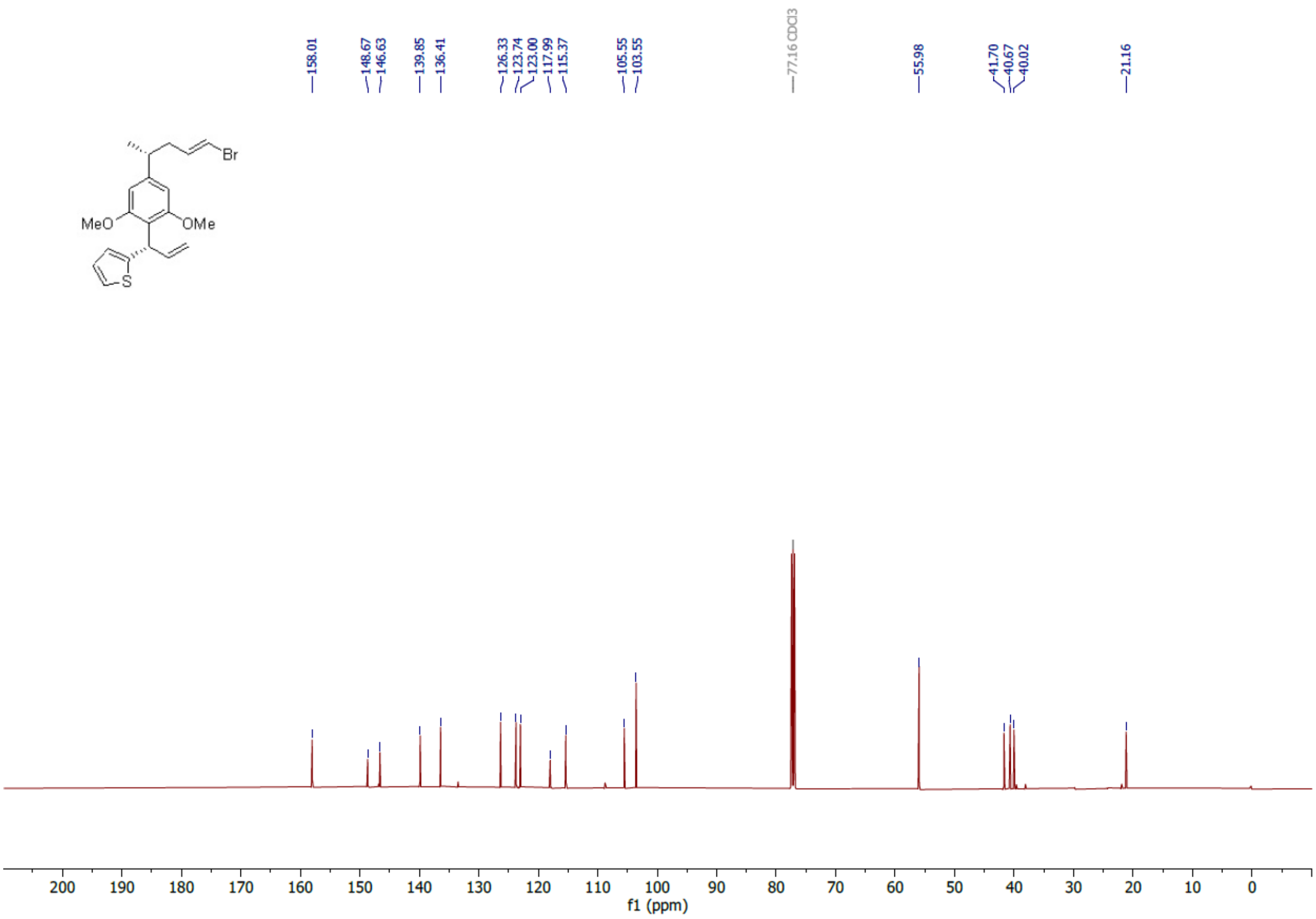

^{1}H NMR (500 MHz, $CDCl_3$) of **12b**

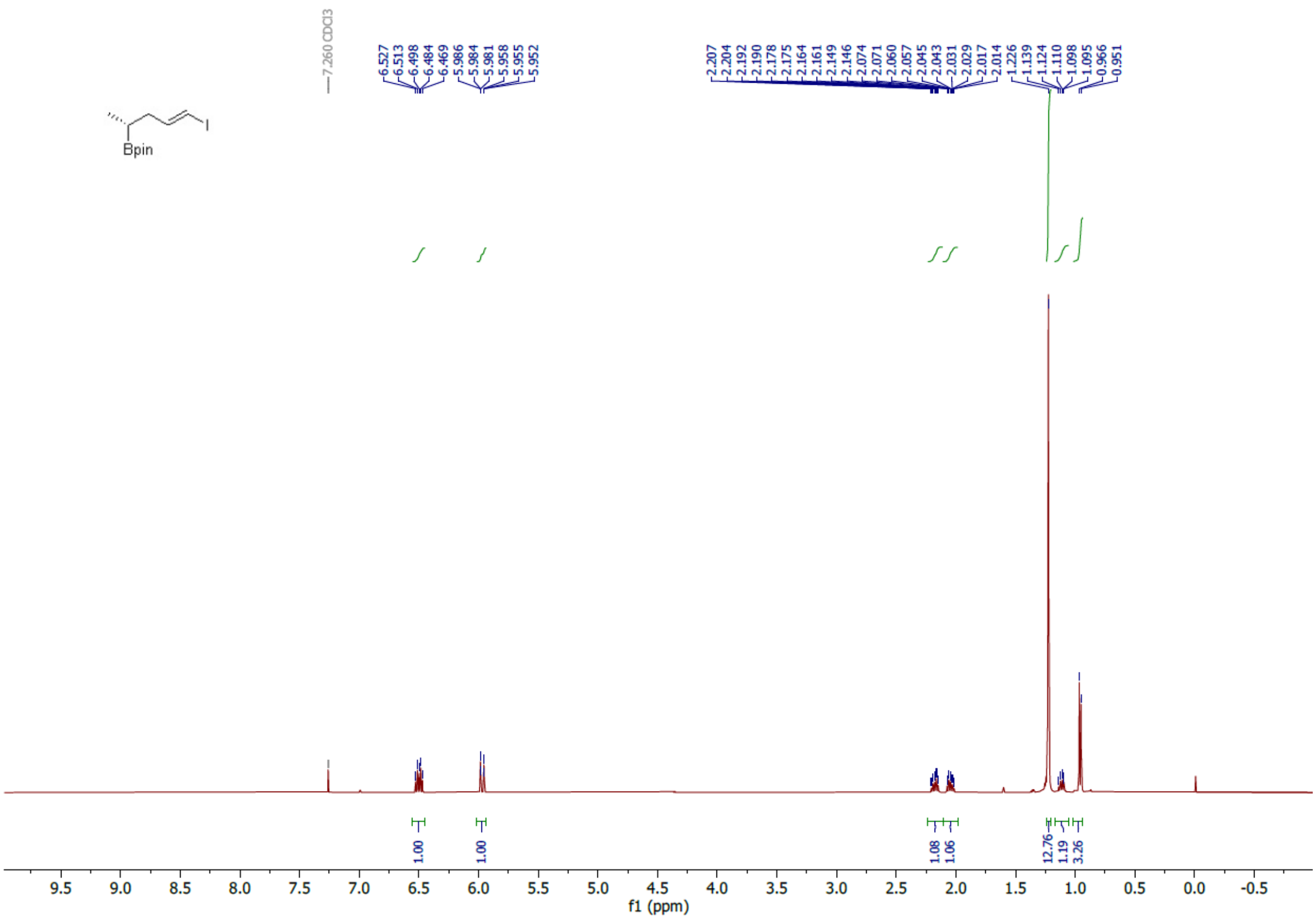

^{13}C NMR (126 MHz, $CDCl_3$) of **12b**

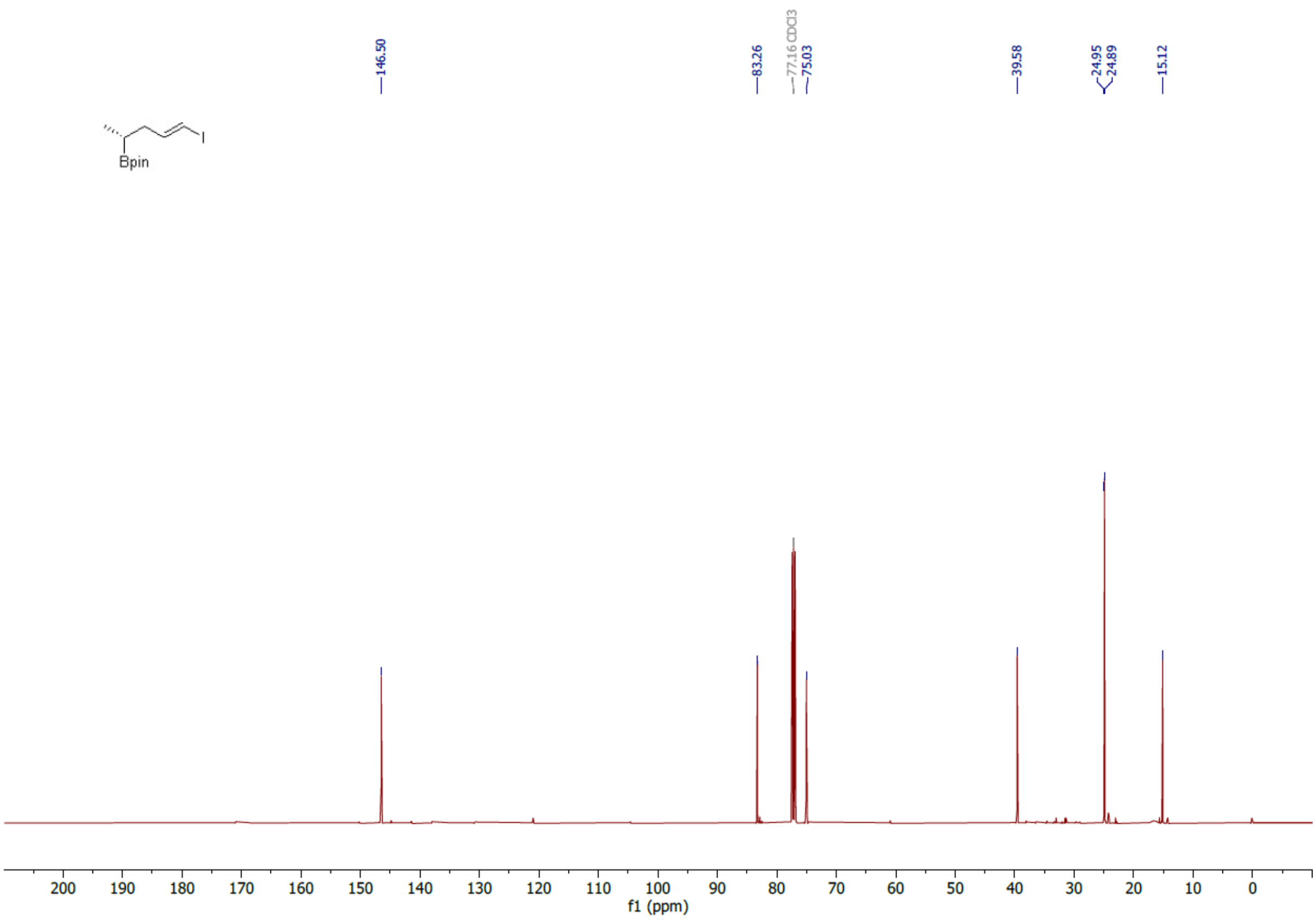

^{1}H NMR (500 MHz, $CDCl_3$) of **11b**

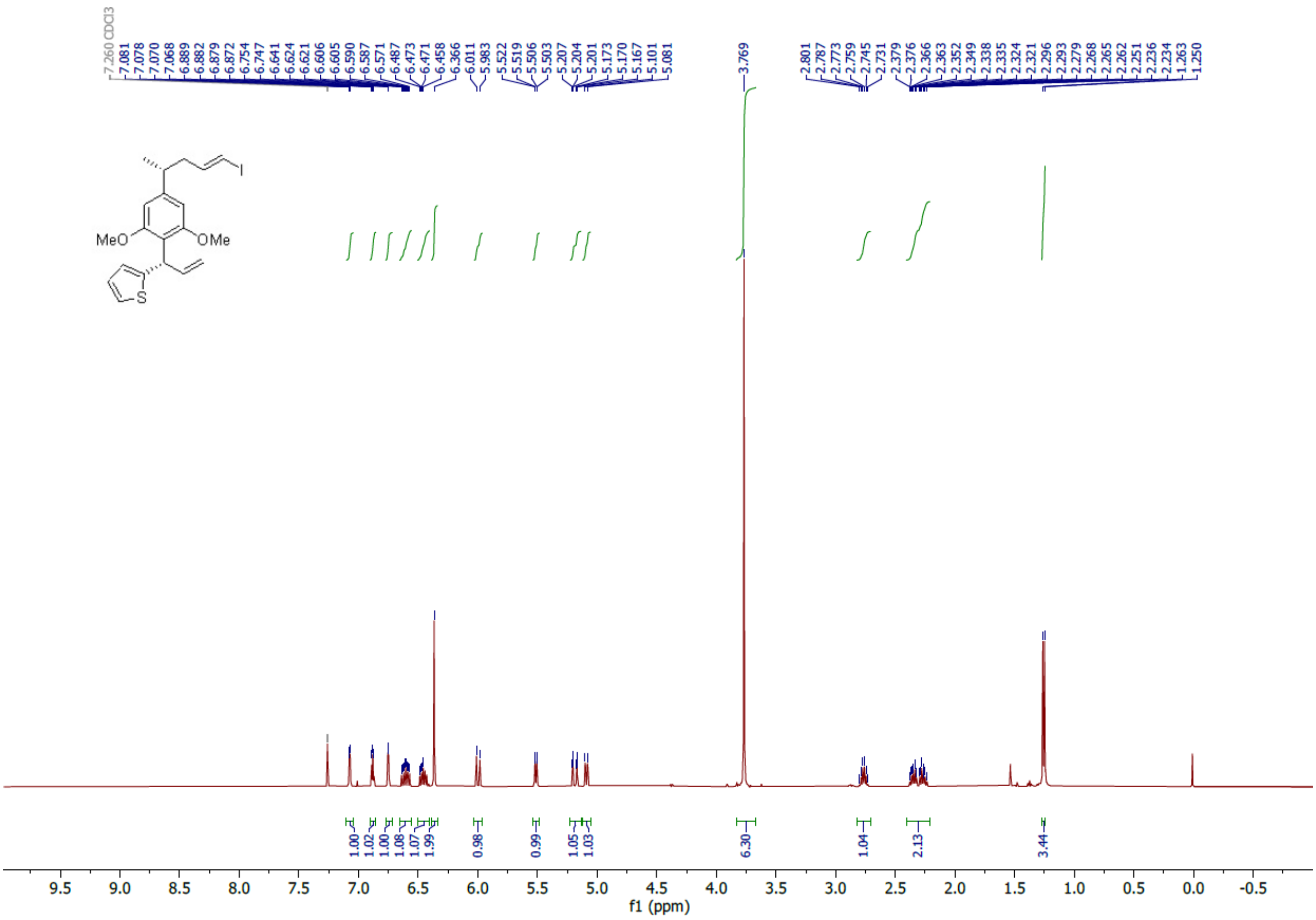

^{13}C NMR (126 MHz, $CDCl_3$) of **11b**

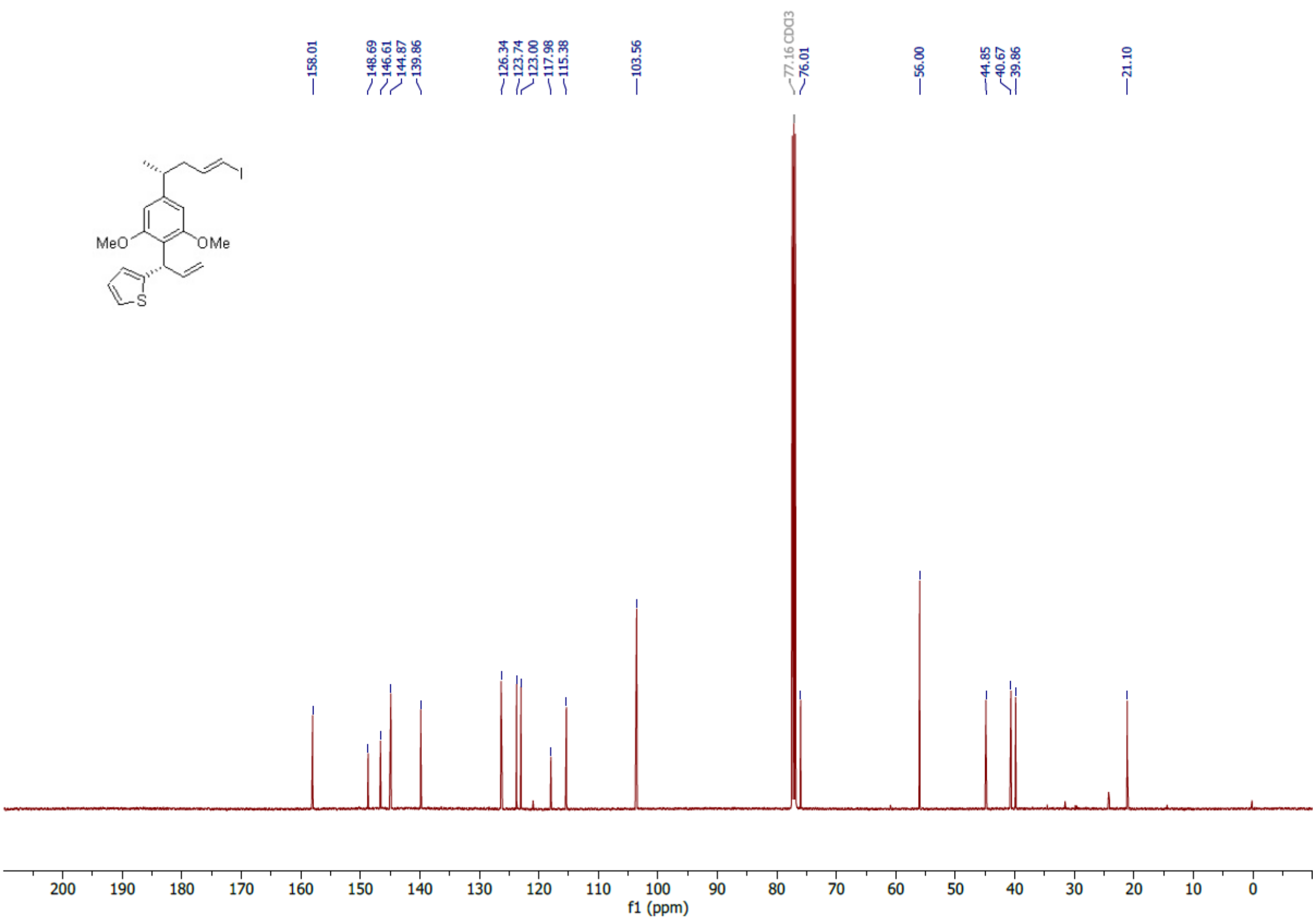

^{1}H NMR (500 MHz, $CDCl_3$) of **17**

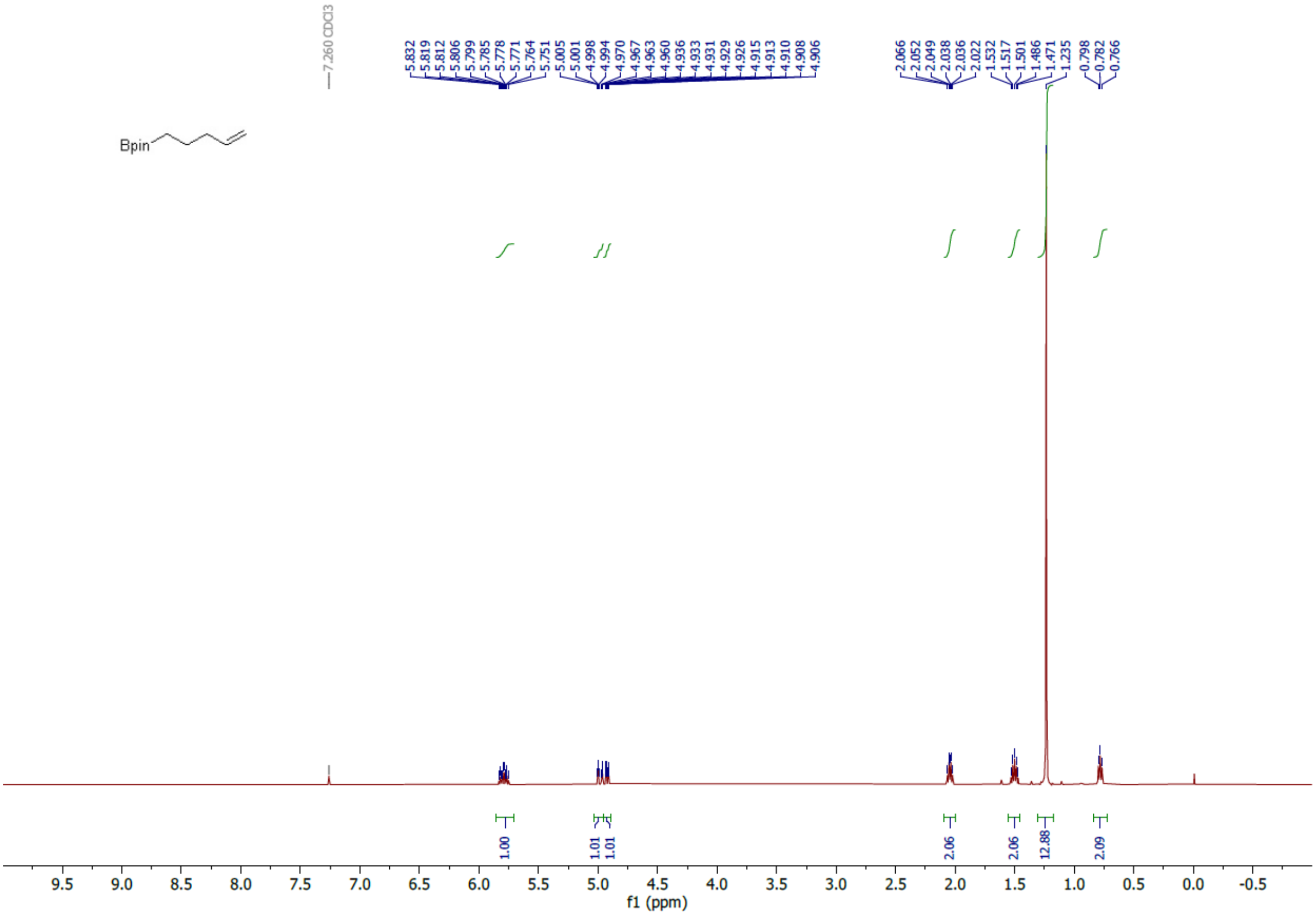

^{13}C NMR (126 MHz, $CDCl_3$) of **17**

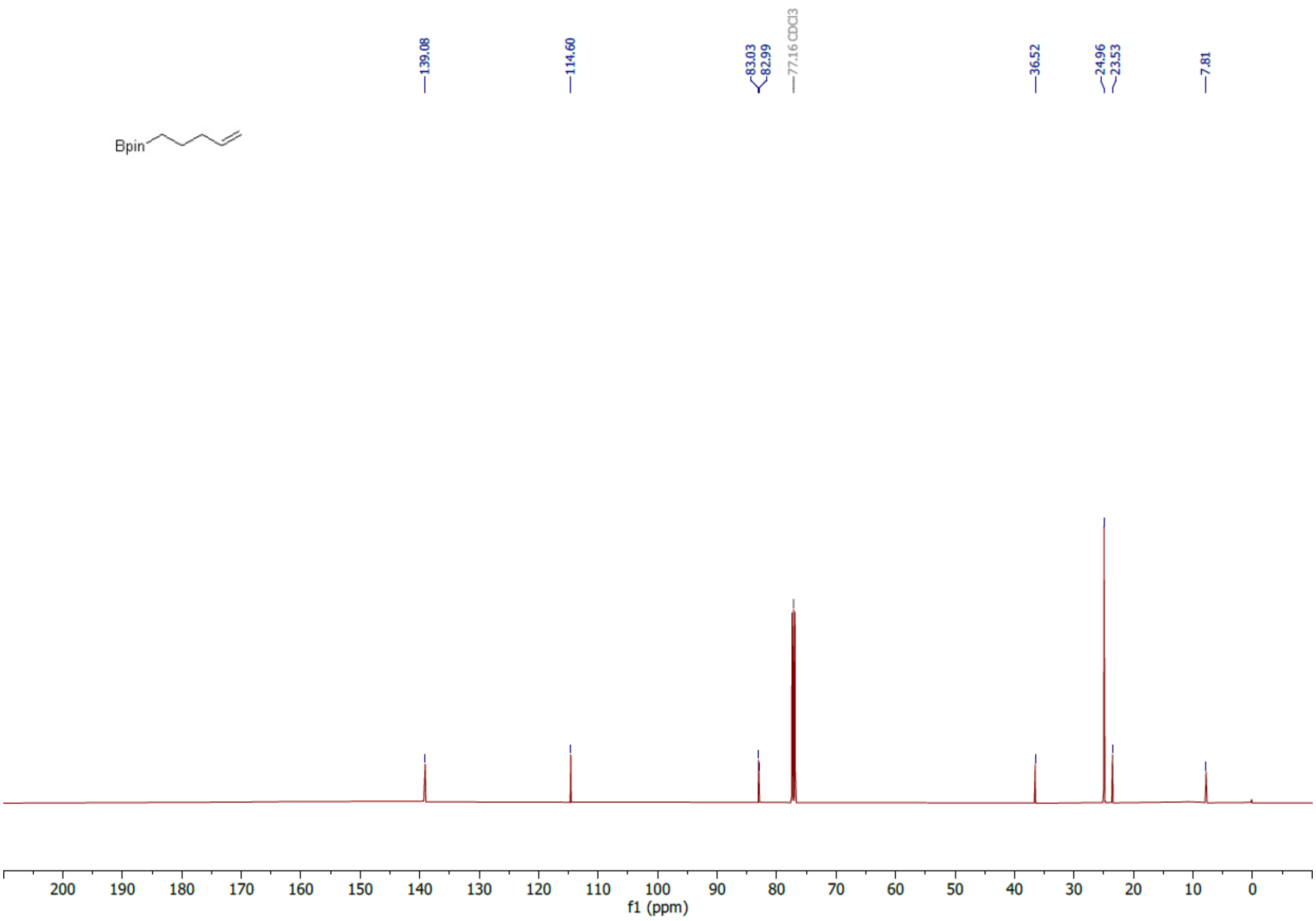

^{1}H NMR (400 MHz, $CDCl_3$) of **9**

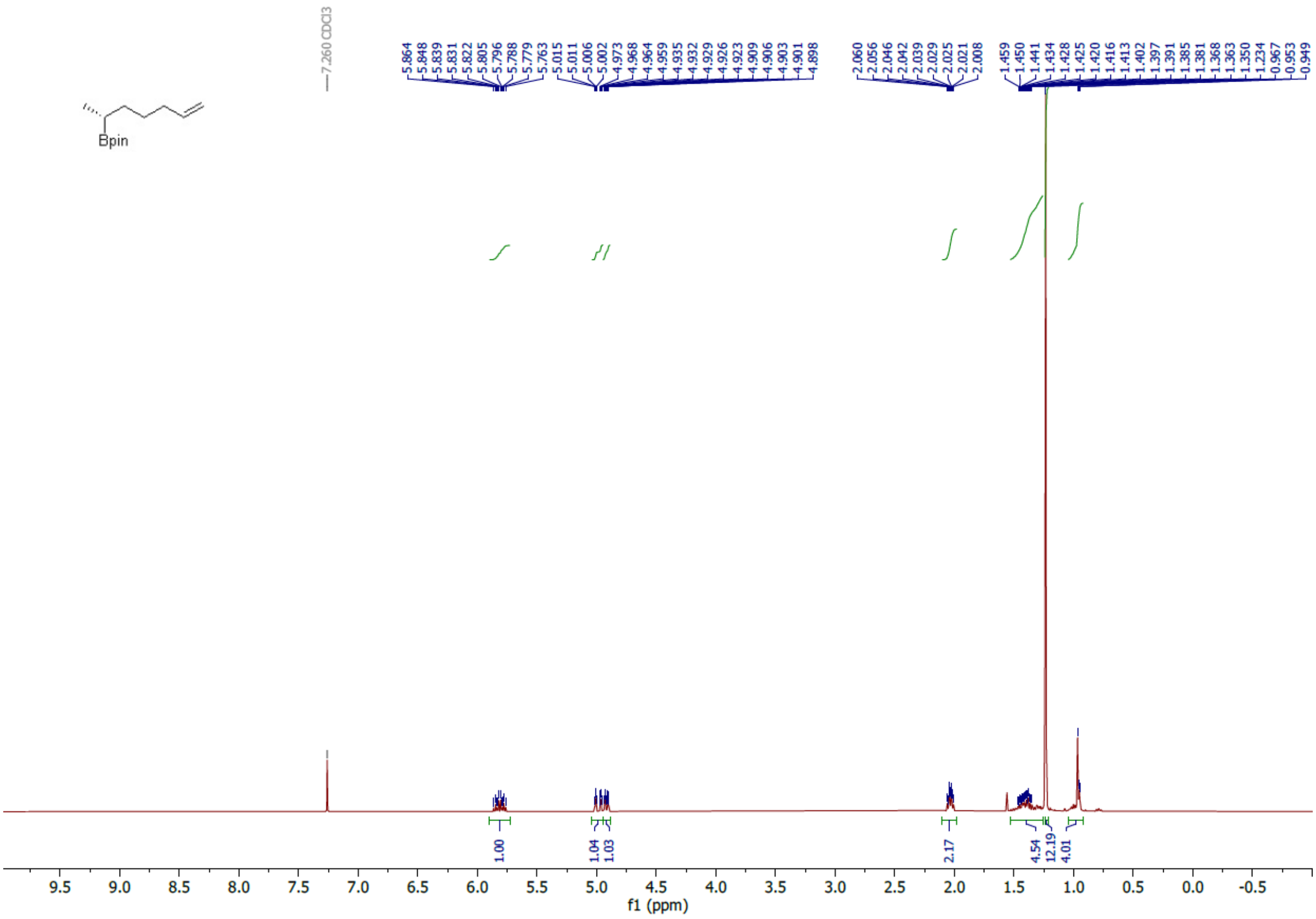

^{13}C NMR (126 MHz, $CDCl_3$) of **9**

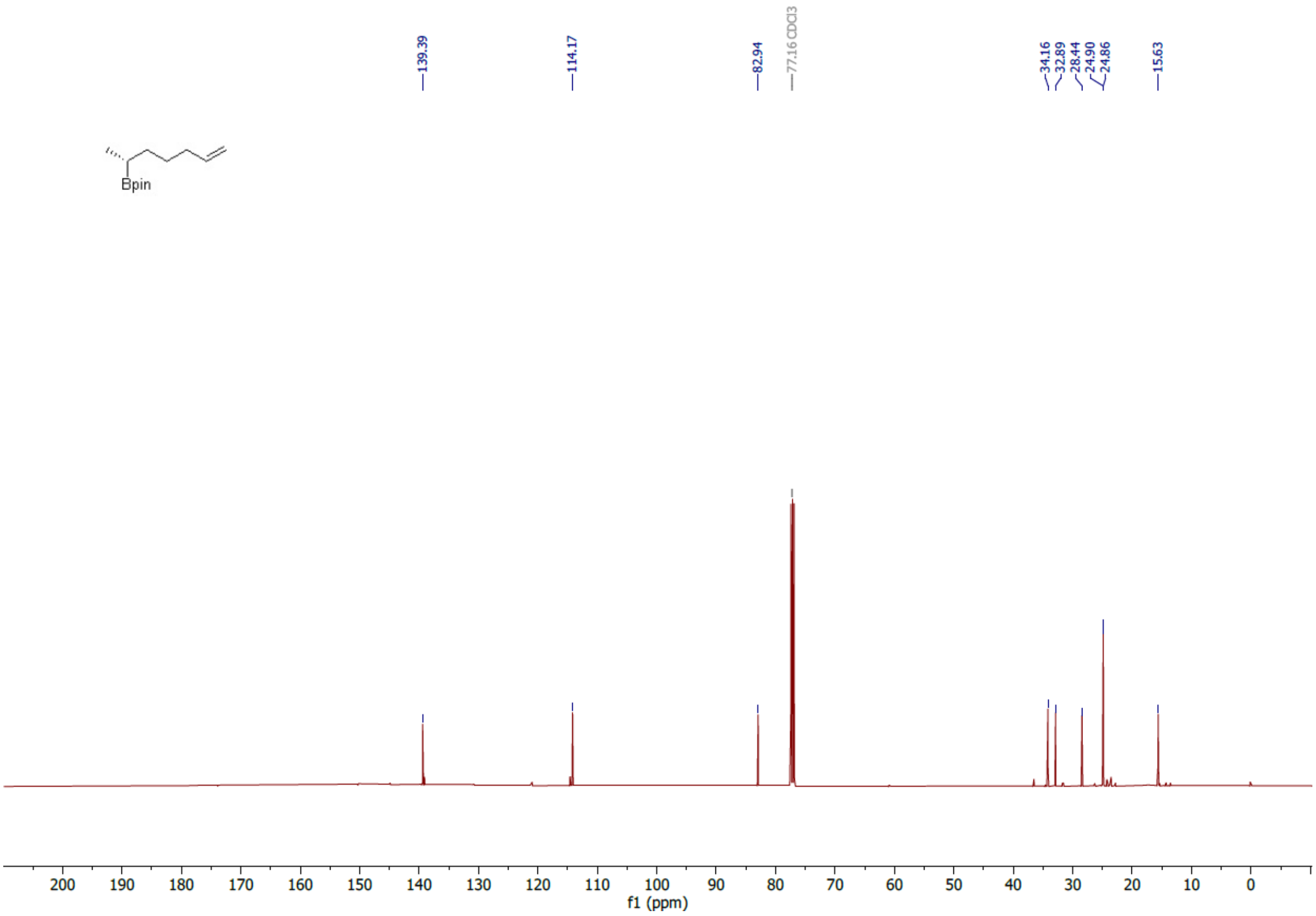

^{1}H NMR (500 MHz, $CDCl_3$) of **8**

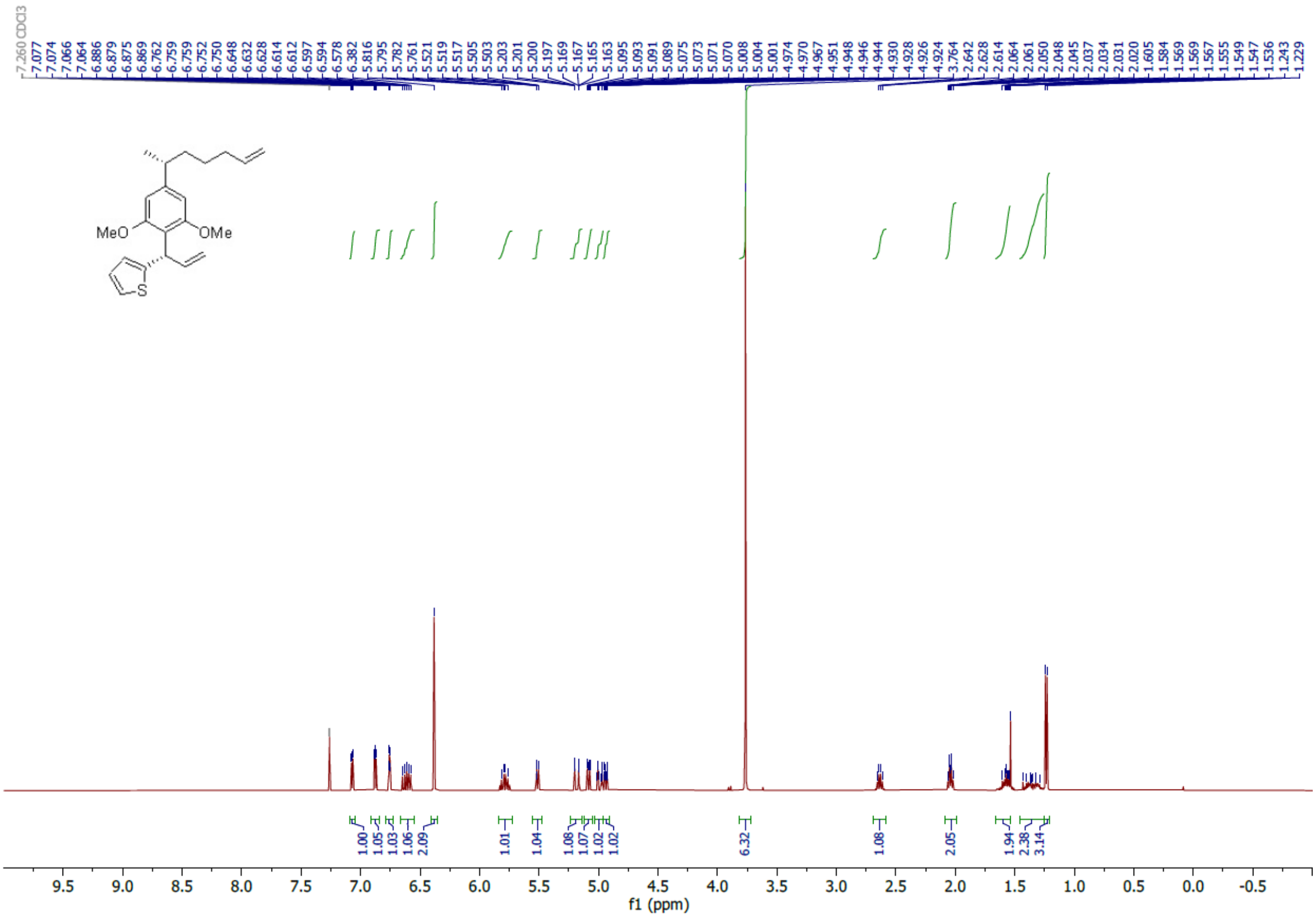

^{13}C NMR (126 MHz, $CDCl_3$) of **8**

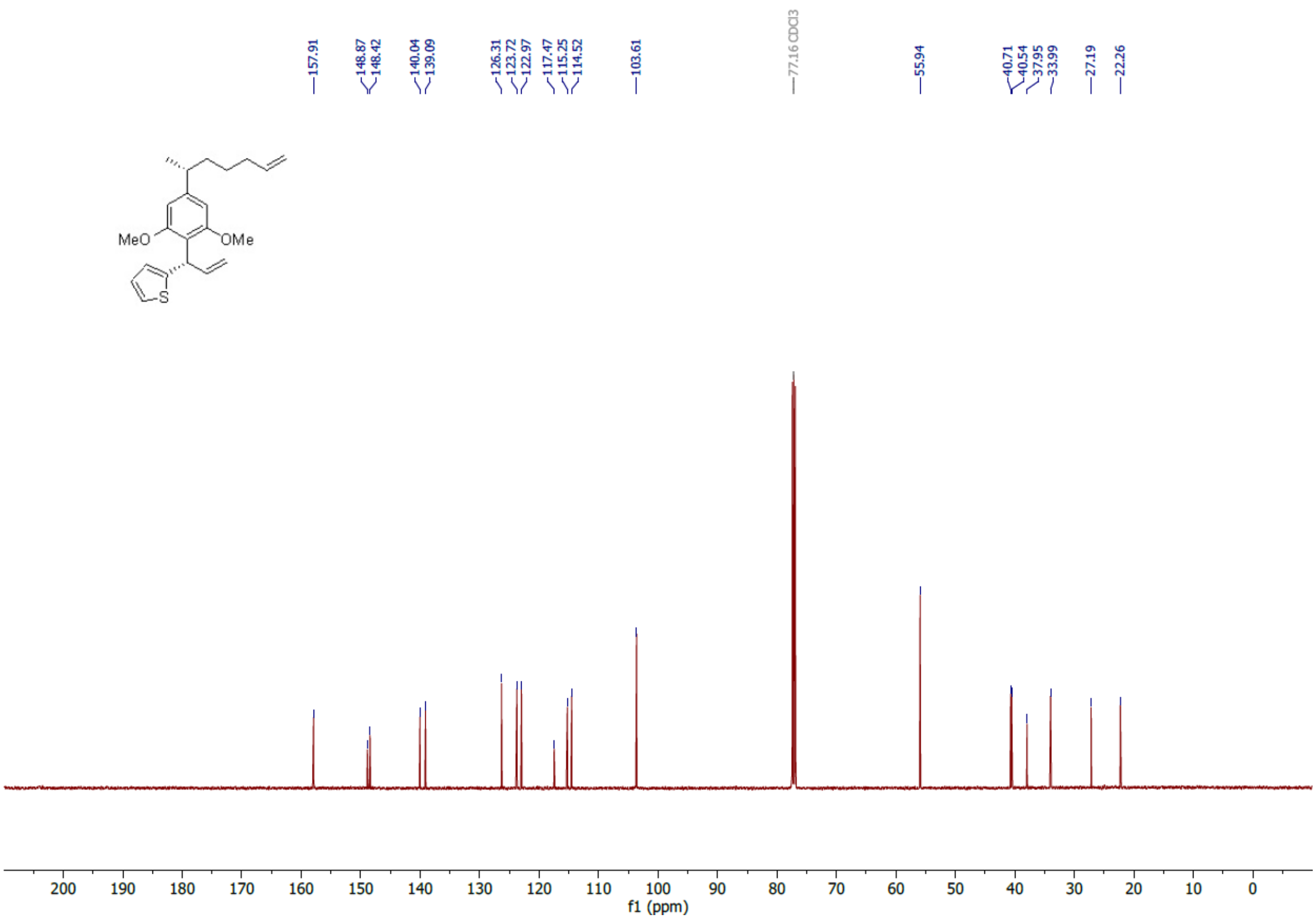

6. References

[1] Burchat, A. F.; Chong, J. M.; Nielsen, N. Titration of Alkyllithiums with a Simple Reagent to a Blue Endpoint. *J. Organomet. Chem.* **1997**, *542*, 281–283.

[2] (a) Xi, Y.; Hartwig, J. F. Diverse Asymmetric Hydrofunctionalization of Aliphatic Internal Alkenes through Catalytic Regioselective Hydroboration. *J. Am. Chem. Soc.* **2016**, *138*, 6703–6706; (b) Xu, N.; Liang, H.; Morken, J. P. Copper-Catalyzed Stereospecific Transformations of Alkylboronic Esters. *J. Am. Chem. Soc.* **2022**, *144*, 11546–11552; (c) Bonet, A.; Odachowski, M.; Leonori, D.; Essafi, S.; Aggarwal, V. K. Enantiospecific sp^2–sp^3 Coupling of Secondary and Tertiary Boronic Esters. *Nat. Chem.* **2014**, *6*, 584–589; (d) Varela, A.; Garve, L. K. B.; Leonori, D.; Aggarwal, V. K. Stereocontrolled Total Synthesis of (−)-Stemaphylline. *Angew. Chem. Int. Ed.* **2017**, *56*, 2127–2131; (e) Ito, H.; Kubota, K. Copper(I)-Catalyzed Boryl Substitution of Unactivated Alkyl Halides. *Org. Lett.* **2012**, *14*, 890–893; (f) Ros, A.; Aggarwal, V. K. Complete Stereoretention in the Rhodium-Catalyzed 1,2-Addition ff Chiral Secondary and Tertiary Alkyl Potassium Trifluoroborate Salts to Aldehydes. *Angew. Chem. Int. Ed.* **2009**, *48*, 6289–6289; (g) Bagutski, V.; French, R. M.; Aggarwal, V. K. Full Chirality Transfer in the Conversion of Secondary Alcohols into Tertiary Boronic Esters and Alcohols Using Lithiation–Borylation Reactions. *Angew. Chem. Int. Ed.* **2010**, *49*, 5142–5145.

[3] Davis, C. R.; Luvaga, I. K.; Ready, J. M. Enantioselective Allylation of Alkenyl Boronates Promotes a 1,2-Metalate Rearrangement with 1,3-Diastereocontrol. *J. Am. Chem. Soc.* **2021**, *143*, 4921−4927.

[4] Ruchti, J.; Carreira, E. M. Rh-Catalyzed Stereospecific Synthesis of Allenes from Propargylic Benzoates and Arylboronic Acids. *Org. Lett.* **2016**, *18*, 2174−2176.

[5] Shen, H.-C.; Wang, Z.-S.; Noble, A.; Aggarwal, V. K. Simultaneous Stereoinvertive and Stereoselective C(Sp3)–C(Sp3) Cross-Coupling of Boronic Esters and Allylic Carbonates. *J. Am. Chem. Soc.* **2024**, *146*, 13719–13726.

[6] Adam, J.-M.; Abel, M.; Jayaraman, S. Process for Preparation of Cyclosporin A Analogs Having a Terminal Diene Group. WO2004089960A2**.**

[7] Sato, K.; Ji, W.; Palmer, L. C.; Weber, B.; Barz, M.; Stupp, S. I. Programmable Assembly of Peptide Amphiphile via Noncovalent-to-Covalent Bond Conversion. *J. Am. Chem. Soc.* **2017**, *139*, 8995–9000.

[8] Aiken, S. G.; Bateman, J. M.; Liao, H.-H.; Fawcett, A.; Bootwicha, T.; Vincetti, P.; Myers, E. L.; Noble, A.; Aggarwal, V. K. Iterative Synthesis of 1, 3-Polyboronic Esters with High Stereocontrol and Application to the Synthesis of Bahamaolide A. *Nat. Chem.* **2022**, *15*, 248–256.

[9] Ogawa, N.; Katagiri, K.; Haimotoa, Y.; Kobayas,Y. Total Synthesis of Resolvin D3. *Org. Biomol. Chem.* **2022**, *20*, 4338–4341.

[10] Mu, Y.; Hartrampf, F. W. W.; Yu, E. C.; Lounsbury, K. E.; Schrock, R. R.; Romiti, F.; Hoveyda, A. H. *E*- and *Z*-Trisubstituted Macrocyclic Alkenes for Natural Product Synthesis and Skeletal Editing. *Nat. Chem.* **2022**, *14*, 640–649.

[11] Bosse, A. T.; Hunt, L. R.; Suarez, C. A.; Casselman, T. D.; Goldstein, E. L.; Wright, A. C.; Park, H.; Virgil, S. C.; Yu, J.-Q.; Stoltz, B. M.; Davies, H. M. L. Total Synthesis of (−)-Cylindrocyclophane A Facilitated by C−H Functionalization. *Science* **2024**, *386*, 641–646.

[12] Högberg, H.-E.; Hedenström, E.; Fägerhag, J. Bakers' Yeast Reduction of Thiophenepropaenals. Enantioselective Synthesis of (S)-2-Methyl-1-alkanols via Bakers' Yeast Mediated Reduction of 2-Methyl-3-(2-thiophene)propenals. *J. Org. Chem.* **1992**, *57*, 2052–2059.

[13] (a) Chen, K.-Y.; Wang, H.-Q.; Yuan, Y.; Mou, S.-B.; Xiang, Z. Chemoenzymatic Synthesis of Cylindrocyclophanes A and F and Merocyclophanes A and D. *Angew. Chem. Int. Ed.* **2023**, *62*, e202307602. (b) Kang, H.-S.; Santarsiero, B. D.; Kim, H.; Krunic, A.; Shen, Q.; Swanson, S. M.; Chai, H.; Kinghorn, A. D.; Orjala, J. Merocyclophanes A and B, Antiproliferative Cyclophanes from the Cultured Terrestrial Cyanobacterium *Nostoc* sp. *Phytochemistry* **2012**, *79*, 109-115.

[14] Ilardi, E. A.; Stivala, C. E.; Zakarian, A. Hexafluoroisopropanol as a Unique Solvent for Stereoselective Iododesilylation of Vinylsilanes. *Org. Lett.* **2008**, *10*, 1727–1730.

[15] Atack, T. C.; Cook, S. P. Manganese-Catalyzed Borylation of Unactivated Alkyl Chlorides. *J. Am. Chem. Soc.* **2016**, *138*, 6139–6142.

[16] Sheldrick, G. M. A Short History of ShelX. *Acta Cryst.* **2008**, *A64*, 339–341.

[17] Dolomanov, O. V.; Bourhis, L. J.; Gildea, R. J.; Howard, J. A. K.; Puschmann, H. Olex2: A Complete Structure Solution, Refinement and Analysis Program. *J. Appl. Cryst.* **2009**, *42*, 339–341.

[18] Sheldrick, G. M. Crystal Structure Refinement with ShelXL. *Acta Cryst.* **2015**, *C71*, 3–8.

[19] Sheldrick, G. M. SHELXS-2018, Programs for X-ray Crystal Structure Solution. University of Göttingen: Göttingen, Germany, **2018**.

[20] Sheldrick, G. M. ShelXT-Integrated Space-Group and Crystal-Structure Determination. *Acta Cryst.* **2015**, *A71*, 3–8.

[21] Software for the Integration of CCD Detector System Bruker Analytical X-ray Systems, Bruker axs, Madison, WI (after **2013**).

[22] CrysAlisPro Software System, Rigaku Oxford Diffraction, **2025**.
